# 4-Hydroxybenzoic Acid-Based Hydrazide–Hydrazones as Potent Growth Inhibition Agents of Laccase-Producing Phytopathogenic Fungi That Are Useful in the Protection of Oilseed Crops

**DOI:** 10.3390/molecules29102212

**Published:** 2024-05-08

**Authors:** Halina Maniak, Konrad Matyja, Elżbieta Pląskowska, Joanna Jarosz, Paulina Majewska, Joanna Wietrzyk, Hanna Gołębiowska, Anna Trusek, Mirosław Giurg

**Affiliations:** 1 Department of Micro, Nano, and Bioprocess Engineering, Faculty of Chemistry, Wroclaw University of Science and Technology, 4/6 Norwida Street, 50-373 Wroclaw, Poland; konrad.matyja@pwr.edu.pl (K.M.); anna.trusek@pwr.edu.pl (A.T.); 2 Division of Plant Pathology and Mycology, Department of Plant Protection, Wroclaw University of Environmental and Life Sciences, 24A Grunwald Square, 50-363 Wroclaw, Poland; elzbieta.plaskowska@upwr.edu.pl; 3 Laboratory of Experimental Anticancer Therapy, Hirszfeld Institute of Immunology and Experimental Therapy, Polish Academy of Sciences, 12 R. Weigla Street, 53-114 Wroclaw, Poland; joanna.jarosz@iitd.pan.wroc.pl (J.J.); wietrzyk@iitd.pan.wroc.pl (J.W.); 4 Institute of Technology and Life Sciences-National Research Institute, 3 Hrabska Avenue, 05-090 Raszyn, Poland; paulina_majewska7@tlen.pl; 5 Department of Weed Science and Tillage Systems, Institute of Soil Science and Plant Cultivation State Research Institute, 61 Orzechowa Street, 50-540 Wroclaw, Poland; hanna.golebiowska1@wp.pl; 6 Department of Organic and Medicinal Chemistry, Faculty of Chemistry, Wroclaw University of Science and Technology, 27 Wybrzeże Wyspiańskiego, 50-370 Wroclaw, Poland

**Keywords:** antifungal activity, benzaldehydes, benzohydrazides, cytotoxicity, oxidoreductase, phytotoxicity, salicylic aldehydes

## Abstract

The research on new compounds against plant pathogens is still socially and economically important. It results from the increasing resistance of pests to plant protection products and the need to maintain high yields of crops, particularly oilseed crops used to manufacture edible and industrial oils and biofuels. We tested thirty-five semi-synthetic hydrazide–hydrazones with aromatic fragments of natural origin against phytopathogenic laccase-producing fungi such as *Botrytis cinerea*, *Sclerotinia sclerotiorum*, and *Cerrena unicolor*. Among the investigated molecules previously identified as potent laccase inhibitors were also strong antifungal agents against the fungal species tested. The highest antifungal activity showed derivatives of 4-hydroxybenzoic acid and salicylic aldehydes with 3-*tert*-butyl, phenyl, or isopropyl substituents. *S. sclerotiorum* appeared to be the most susceptible to the tested compounds, with the lowest IC_50_ values between 0.5 and 1.8 µg/mL. We applied two variants of phytotoxicity tests for representative crop seeds and selected hydrazide–hydrazones. Most tested molecules show no or low phytotoxic effect for flax and sunflower seeds. Moreover, a positive impact on seed germination infected with fungi was observed. With the potential for application, the cytotoxicity of the hydrazide–hydrazones of choice toward MCF-10A and BALB/3T3 cell lines was lower than that of the azoxystrobin fungicide tested.

## 1. Introduction

In recent years, the number of active substances used as plant protection products has decreased as the ones with the highest toxicity and harmfulness have been withdrawn from the environment [1]. Furthermore, the fungicide resistance of phytopathogens is an increasingly severe problem worldwide [2]. Improper application of available preparations, which often work according to similar mechanisms of action, makes the pathogens immune to them. Therefore, plant protection strategies have evolved and changed in more complex activities, including nonchemical and chemical control methods [3]. Both approaches, when applied simultaneously, may decrease pathogen growth rates and the use of fungicides, minimizing selection for resistant strains. Nevertheless, chemical treatment is still a valuable and required tool for plant infection control and also reduces selection for pathogen strains with increased virulence against resistant cultivars [3,4]. Biologically active compounds for fungal disease control of plants could be divided into several classes as multisite mode-of-action fungicides, single site-of-action fungicides, plant activators, natural products and natural product-derived fungicides, biopesticides, chemical fumigants, and biofumigants [4]. Most have fungicidal or fungistatic effects, while others do not influence pathogens as they induce plant resistance to their attack.

In mild climates, many pathogens of plants can cause significant economic losses during crops’ cultivation and storage. One of the most difficult to limit is polyphagous fungi, such as *Botrytis cinerea* Pers. and *Sclerotinia sclerotiorum* (Lib.) de Bary, due to there being many host plants and the ability to create resting bodies (sclerotia) in plant debris in the soil that can last for many years. *Botrytis cinerea*, which causes gray mold disease, is a significant plant threat in the growing season. It is a cosmopolitan fungus noted throughout the world. It infects more than 240 different plant species [5]. The most significant losses result in temperate and tropical climate regions. It is a facultative parasite that can live a saprotrophic lifestyle in the form of mycelium and sclerotia on plant debris in the soil. Still, very often, it becomes a parasitic lifestyle under favorable conditions for its development. During the growing season, large amounts of conidial spores spread the disease on the plantation. The pathogen contributes to significant economic losses in horticultural crops (mainly strawberries and berry bushes), vegetable crops (e.g., cucumber), and ornamental plants [6,7]. The fungus produces toxins, which are derivatives of botrydial and botcinins, which are undesirable in strawberry fruit [8]. The result of infestation may be gangrene of seedlings, gangrene of flowers, and gray rot of shoots and fruit. *Sclerotinia sclerotiorum* causes white mold disease. The fungus is a polyphag that infects about 400 species of plants [9] and occurs on all continents. In mild climates, it has an adverse effect on the cultivation of rapeseed, sunflower, and vegetables. It causes yield losses of up to 60% in oilseed rape, as infected plants often result in massive seed drop. White mold causes considerable losses in the storage of root vegetables, especially carrots. The pathogen does not produce conidial spores, only ascospores. They form in fruiting bodies (apothecia). Under unfavorable conditions, the fungus produces sclerotia, in the form of which it overwinters in plant debris or stored roots. In the spring, sclerotia grows cupped apothecia on long legs, promoted by moist soil and higher temperatures [10,11].

*Cerrena unicolor* (Bull.) Murrill is a different type from the above-characterized mold fungi, as it is a decaying arboreal fungus belonging to the *Basidiomycetes* and the family *Polyporaceae*, classified as a wound parasite and saprotroph [12]. It is found in deciduous and mixed woodland and in parks and gardens. It attacks and colonizes living and weakened broadleaf trees, most commonly chestnut trees (*Aesculus* sp.), maples (*Acer* sp.), beech trees (*Fagus* sp.), and oaks (*Quercus* sp.). The fungus is widespread throughout the year in Europe, Africa, and South America but is rare. It is also commonly known as the mossy maze polypore but presents high similarity to the genus *Trametes* fungi [13]. It is among the fungi that cause white wood rot, characterized by the decomposition of all the components of plant biomass: cellulose, hemicellulose, and lignin. The fungi described above have the common trait of producing laccases–copper-dependent oxidoreductases. The enzymes from *B. cinerea* [14,15], *S. sclerotiorum* [16], and *C. unicolor* [17] have been isolated and described in the literature.

Laccases are catalytic proteins with particular substrate specificity toward a broad group of electron-rich arenes, mostly phenols, that are oxidized through a one-electron mechanism directly with molecular oxygen or in the presence of mediators. The laccase active center comprises four copper atoms (type Cu1, type Cu2, and two type Cu3s), in which Cu1 interacts with an electron-rich substrate, and the trinuclear-Cu cluster binds oxygen molecules. In nature, laccase conducts the polymerization and depolymerization reactions, which are related to the natural role of lignification and delignification processes in plants and fungi, respectively [14]. Laccases may be produced in different isoforms by fungi, usually in the presence of polyphenols and copper ions [18,19,20,21].

This research focuses on compounds containing fragments of naturally occurring molecules that may act as antifungal agents or plant resistance activators. In this context, phenolic compounds are an interesting group of low-molecular-weight species, including simple phenols, aldehydes, polyphenolic acids, flavonoids, coumarins, stilbenes, and tannins that are widely distributed in the plant kingdom [22]. Phenolic acids have essential physiological functions, such as defense against herbivores and pathogens, antioxidation, protection of cell structures from harmful solar radiation or free radicals, and other substructural functions [23]. Of the various phenolic compounds found in plants, hydroxybenzoic acids are among the simplest phenolic acids that act in response to environmental stresses. For instance, in cucumber leaves infected with *Pseudomonas syringae* van Hall, the synthesis and accumulation of both salicylic and 4-hydroxybenzoic acid were observed in the stems and petioles in response to a mobile signal from the inoculated leaf as part of systemic acquired resistance [24]. Furthermore, it was tested when applied exogenously to wheat cultivars to test crop tolerance to short-term drought and freezing, resulting in increased stress tolerance [25]. In other studies, 4-hydroxybenzoic acid was found to be synthesized de novo and covalently linked to cell wall polysaccharides in the carrot protoplast system upon activating the phenylpropanoid pathway induced through fungal elicitor from plant pathogen *Pythium aphanidermatum* (Edson) Fitzp. [26,27]. Plant material contains, among others, thymol, salicylic acid, and salicylic aldehyde, which were tested separately and showed total fungal inhibition growth of *Fusarium*, *Penicillium*, and *Aspergillus* species at a high concentration of 1 g/L [28]. Interestingly, thymol was effective against non-plant and plant pathogens such as *Cryptococcus neoformans* Vuill. and *Rhizopus* sp., respectively, and its isomer carvacrol against *Candida albicans* (CP Robin) Berkhout [29]. Among various phenolic compounds, 4-hydroxybenzoic acid is one of the simpler polyphenolic laccase mediators [30], and it is still within our area of interest.

**Figure 1:**
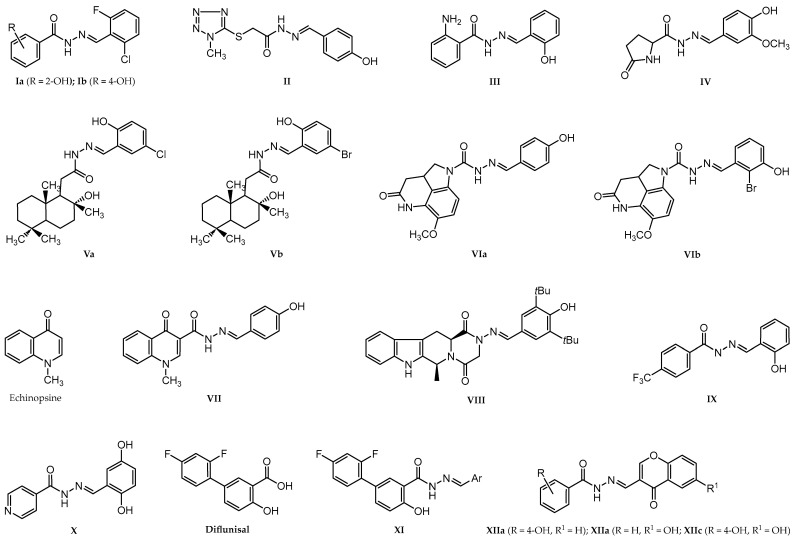
Structures of the aryl OH-substituted hydrazide–hydrazones **I**–**XII** with significant biological activities.

To date, many studies have been carried out on aryl OH-substituted hydrazide–hydrazones (see Figure 1), covering several biological aspects, such as antimicrobial [31,32,33,34,35], antiviral [33,36,37,38,39], anticancer [40,41,42,43], antiradical [31,44], enzyme inhibitors [40,44,45,46,47,48,49], cytotoxicity [36,50], and photophysical activity [51,52]. Recently, Mali et al. [33] and Popiołek [32] published exhaustive, comprehensive reviews on the biological activity, including antimicrobial activity, of hydrazides and their derivatives. Several articles described the examples of aryl-OH-substituted hydrazide–hydrazones in the context of the antibacterial, antifungal, and antiviral activities. For instance, the derivatives of 2-chloro-6-fluorobenzaldehyde with salicylic acid and 4-hydroxybenzoic acid hydrazides **Ia** and **Ib**, respectively, were active against the Gram-positive bacterium *Bacillus subtilis* [31]. In other studies, the 4-hydroxybenzaldehyde derivative of 2-[(1-methyl-1*H*-tetrazol-5-yl)thio)acetohydrazide (**II**) showed weak anticandidal activities (MIC 0.5–1.0 mg/mL) compared to ketoconazole (MIC 1–31 µg/mL) and promising anticancer activity against NIH3T3 cell line with IC_50_ = 0.632 mM [34]. On the other hand, the imine derivative of salicylic aldehyde and anthranilhydrazide (**III**) had very good antibacterial and antifungal effects with the most susceptible *Staphylococcus aureus* and *Candida albicans* strains [35]. A derivative of vanillin aldehyde and pyrrolidin-2-one (**IV**) had activity against the mold *Geotrichum candidum* comparable to hymexazol [53]. Interestingly, the 5-chloro-2-hydroxybenzaldehyde derivative of sclareolide **Va** drimane sesquiterpenoid from *Salvia sclarea* L. had thiadiazol copper (used as positive control) antibacterial activity on *Xanthomonas oryzae* pv. *oryzae* at the concentration of 50 µg/mL, but the 5-bromo-2-hydroxybenzaldehyde derivative **Vb** inactivated the TMV virus in vivo at 0.5 mg/mL concentration similar to ribavirin [37].

The 4-hydroxybenzaldehyde derivative of dehydrobufotenine analog **VIa** showed ribavirin activity at 500 µg/mL concentration and more promising ningnanmycin activity at 100 µg/mL toward plant tobacco mosaic virus (TMV) and 2-bromo-3-hydroxybenzaldehyde derivative **VIb** shown chlorothalonil fungicidal activities at concentration 50 µg/mL toward phytopathogenic *Alternaria solani*, *Fusarium graminearum*, *Sclerotinia sclerotiorum*, *Rhizoctonia solani*, and *Botrytis cinerea* [54]. An alkaloid from *Echinops sphaerocephalus* L.–echinopsine and their hydrazide–hydrazone derivative were examined as fungicidal compounds. The 4-hydroxybenzaldehyde derivative **VII** at a concentration of 50 µg/mL showed very good activity against phytopathogenic fungi: *Sclerotinia sclerotiorum* and *Magnaporthe grisea* [55]. The indole derivative diketopiperazine acylhydrazone and 3,5-di-*tert*-butyl-4-hydroxybenzaldehyde (**VIII**) showed high inhibitory activity on tobacco mosaic virus (TMV), comparable to the pesticide ningnanmycin, and moderate activity on diamondback moth (*Plutella xylostella*) and mosquito (*Culex pipiens pallens*) larvae [56].

Hydrazide–hydrazones, derivatives of 4-trifluoromethylhydrazide, showed inhibition of two important enzymes involved in the breakdown of choline neurotransmitters, important in the treatment of neurological diseases, including Alzheimer’s disease. As a result, the salicylaldehyde derivative **IX** was found to be a highly selective acetylcholinesterase (AChE) inhibitor in relation to butyrylacetylcholinesterase (BuChE) with a BuChE/AChE selectivity of 18.6. This was a promising result for the non-selective drug galantamine (BuChE/AChE = 1.8) [45]. Furthermore, studies have shown that this derivative is non-toxic in tests on liver cancer cell HepG2 (human hepatoblastoma cell line). In the article dealing with the same issue, derivatives of isoniazid with salicylic aldehydes or 4-hydroxybenzaldehydes were inactive against AChE, but the gentisaldehyde derivative **X** had activity comparable to isoniazid with an IC_50_ of 4.8 µM on MPO (myeloperoxidase), and the free radical scavenging efficiency was at the level of quercetin control [44]. The research [38] was conducted on a series of diflunisal (2′,4′-difluoro-4-hydroxybiphenyl-3-carboxylic acid) hydrazide–hydrazones to determine antiviral activity against hepatitis C virus (HCV) and cytotoxicity toward liver cancer cell lines (Huh7, HepG2, HepB3, Mahlavu). From the results of these authors, the imine derivative of diflunisal hydrazide with 2-pyridylaldehyde (**XI**) was identified as the most active against HCV with an EC_50_ of 3.9 µM and sequential on all launched lines, with the lowest EC_50_ of 4.74 µM for the Mahlavu line. In the work [49] on α-glucosidase inhibitors used in the treatment of type-2 diabetes, a series of chromones aldehydes derivatives with various substituted hydrazides were obtained. In general, hydroxylated derivatives of hydrazide–hydrazones and chromene aldehydes **XIIa-c** showed much higher activity compared to the typically used α-glucosidase inhibitor, acarbose drug.

The above examples demonstrate a broad spectrum of hydrazone activity in different biological areas. Interest in this class of compounds is still growing in plant fungicide development [53,54,55,56].

The hydrazide–hydrazones were tested as a part of our ongoing scientific program to discover low-molecular-weight compounds that are antimicrobial agents [57], the inhibitors of essential enzymes that are overproduced by microorganisms during disease development in plants [47,48] and humans [58,59,60], as well as those that act directly toward cell lines [61,62]. In our previous work, we discovered potent laccase inhibitors as a target, as this enzyme is secreted by phytopathogenic fungi, contributing to various plant diseases. Therefore, these potent laccase inhibitors can prevent or weaken pathogen attacks through chemical protection. Continuing our previous research on laccase inhibitors [47,48], we have addressed the effect of changing the structure of the acyl and phenylidene units in hydrazide–hydrazones, inspired by plant secondary metabolites such as 4-hydroxybenzoic acid and salicylic aldehydes, on the direct mycelium growth inhibition of selected phytopathogenic fungi.

## 2. Results and Discussion

### 2.1. Syntheses and Characterization

In general, hydrazide–hydrazones, which are the subjects of our studies, comprise aromatic aldehydes and benzoic acid fragments linked by hydrazine (Ar–CH=N–NH–(C=O)–Ar′). These are usually crystalline compounds synthesized in a condensation reaction of an equimolar amount of hydrazide, formed from benzoic acid esters and hydrazine hydrate with aldehyde molecules. The reactions are carried out in the classic conditions or with a mechano-chemical procedure. The first procedure is conducted in an organic solvent with hydrazine hydrate in the first step and an acetic acid additive in the second step to form a crystalline pure product directly isolated from the reaction mixture [47]. The second procedure covers the step of grinding substrates, esters with hydrazine hydrate, and later forming hydrazide and aldehyde with a few drops of acetic acid, followed by a recrystallization step from organic solvent [39]. Both classical and mechanical approaches generally provide pure products from good to quantitative yields. However, the first approach does not require the additional purification step and offers safer conditions for working with hydrazine hydrate and acetic acid.

**Scheme 1:**
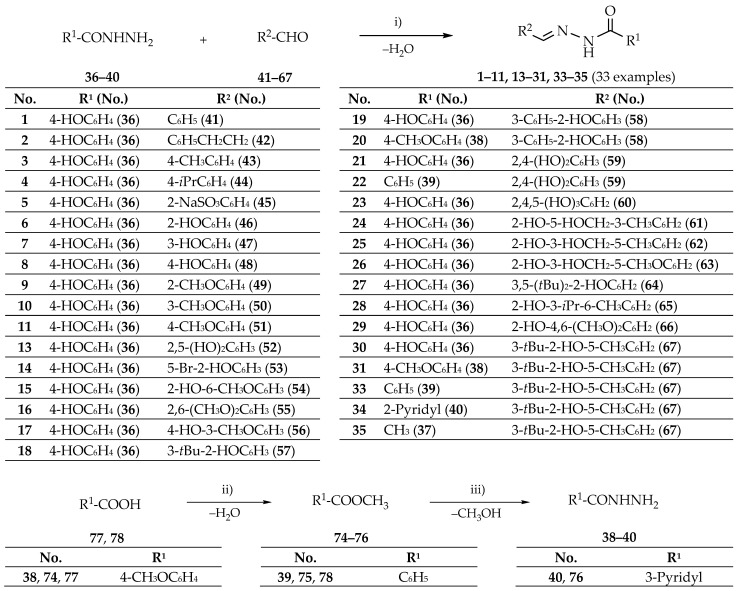
Hydrazide–hydrazones **1**–**11**, **13**–**31**, and **33**–**35**; hydrazides **38**–**40**; and methyl ester of benzoic acids **74** and **75** preparations, and the numbering of hydrazides, aldehydes, methyl ester of benzoic acid, and benzoic acids used. *Reagent and conditions*: (i) CH_3_OH, AcOH (cat.), reflux, 2–8 h [47,48]; (ii) CH_3_OH, SOCl_2_ or H_2_SO_4_ (cat.), reflux [47,48]; (iii) H_2_N-NH_2_ × H_2_O, CH_3_OH, reflux [47,48].

**Scheme 2:**
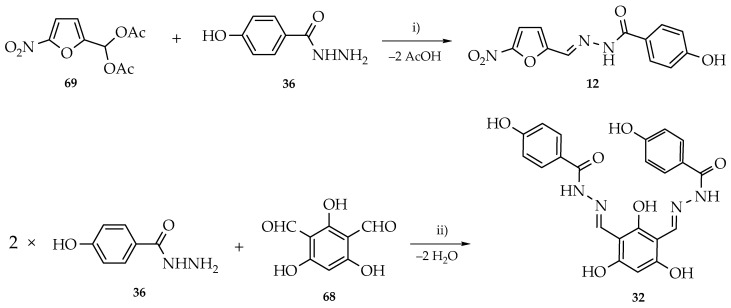
Nifuroxazide (**12**) and hydrazide–hydrazone **32** preparations. *Reagent and conditions*: (i) CH_3_OH, reflux, 20 h; (ii) CH_3_OH, AcOH (cat.), reflux, 20 h [47,48].

The target hydrazide–hydrazones **1**–**35** were synthesized by condensation of carboxylic acid hydrazides **36**–**40** [63] with aldehydes **41**–**68** or diacetylacetal **69** [64,65] by conventional heating in methanol with the literature procedure in [47], generally in the presence of a catalytic amount of acetic acid additive (Scheme 1 and Scheme 2).

In the synthesis of the nifuroxazide drug (**12**), the 4-hydroxybenzoic acid hydrazide (**36**) reacted with 5-nitro-2-furaldehyde diacetate (**69**) in the absence of the catalyst in agreement with the nature of the diacetate substrate **69** (Scheme 2).

The aldehydes **53**–**55**, **57**, **58**, **61**, **62**, and **65**–**67** were previously obtained in our laboratory [48]. The 2,4,5-trihydroxybenzaldehyde (**60**) was obtained by formylation of 1,2,4-trihydroxybenzene (**70**) with triethyl orthoformate mediated by AlCl_3_ in toluene [66].

The 2-hydroxy-3-hydroxymethyl-5-methoxybenzaldehyde (**63**) was obtained in a two-step literature procedure starting from 4-methoxyphenol (**71**) [67]. In the first step, phenol **71** underwent hydroxymethylation with an excess of paraformaldehyde in an alkaline methanol–water solution [68]. Finally, the obtained crude 2,6-dihydroxymethyl-4-methoxyphenol (**72**) [68] was directly oxidized with active manganese dioxide (MnO_2_) in chloroform in mild reaction conditions with a satisfactory 75% yield [67].

In the case of hydrazide–hydrazone **32**, the 2,4-diformylphloroglucinol (**68**) and two equivalents of 4-hydroxybenzoic acid hydrazide (**36**) were used in the standard catalytic reaction condition with prolonged reaction time to 20 h (Scheme 2). The 2,4-diformylphloroglucinol (**68**) was obtained by direct diformylation of phloroglucinol–1,3,5-trihydroxybenzene (**73**) in 1,4-dioxane with Vilsmeier reagent [69].

The 4-methoxybenzoic acid hydrazide (**38**), benzoic acid hydrazide (**39**), and nicotinic acid hydrazide (**40**) were prepared from appropriate benzoic acid methyl esters **74**–**76** and hydrazine monohydrate as previously reported [47,48]. The key esters **74** and **75** were obtained from the corresponding 4-methoxybenzoic acid (**77**) and benzoic acid (**78**), respectively [47,70], as presented in Scheme 1.

Thirty-four hydrazide–hydrazones **1**–**34** have *E* geometry on the azomethine group (CH=N). An exception is a salicylidenehydrazone **35** derived from acetic acid hydrazide, which was isolated as an *E* isomer predominantly with a ratio of 79:21. The twenty-eight products **1**–**3**, **5**–**11**, **14**–**21**, **24**, **25**, **27**–**31**, and **33**–**35** were characterized in our previous articles [47,48]; the remaining seven aroylhydrazone derived from benzoic and 4-hydroxybenzoic acid hydrazides **4**, **12**, **13**, **22**, **23**, **26**, and **32** were characterized in this work [71].

The results of spectroscopic analyses of new hydrazide–hydrazones and their precursors have typical characteristics for these groups of compounds [47,48,72]. Interestingly, on the HRMS analysis of both known and new hydrazide **25** and **26** derivatives of 5-methyl- and 5-methoxy-3-hydroxymethyl-salicylaldehydes (**62** and **63**), respectively, additional mono-dehydration ions were observed. On the contrary, only mono-dehydration ions were detected in the case of the salicylic aldehyde **62** and **63** substrates.

### 2.2. Biological Studies

All prepared hydrazide–hydrazones were used in a test for determination of their antifungal activity against *Botrytis cinerea*, *Sclerotinia sclerotiorum*, and *Cerrena unicolor* at basal 50 μg/mL concentration. Detailed antifungal tests were conducted for the most active compounds **18**, **19**, **27**, **28**, and **30** to determine the IC_50_ value in the 0–50 μg/mL range. We also investigated the phytotoxicity of seven representative compounds of choice **13**, **17**, **18**, **19**, **25**, **27**, and **30** using three oilseed dicot plant representatives as flax (*Linum usitatissimum* L.), sunflower (*Helianthus annuus* L.), and rapeseed (*Brassica napus* L. var. *napus*). We chose the representatives of dicots as they are the most frequently attacked by the *B. cinerea* and *S. sclerotiorum*, preferentially growing on aerial plant tissues rich in pectin [73]. For flax and sunflower, we also performed further experiments on the properties of tested compounds to induce the resistance for *B. cinerea* and *S. sclerotiorum* attack, respectively. Finally, the representative compounds of choice were evaluated on their cytotoxicity on the human and mouse cell lines.

#### 2.2.1. Antifungal Activities

Results of the screening activities of the hydrazide–hydrazones **1**–**35** at a concentration of 50 µg/mL are presented in Table 1, Table 2 and Table 3. The magnitude of mycelial growth inhibition (%) caused by the selected compound was related to a control sample with a fungus growing on a medium free of the tested compound. A value of 100% refers to complete inhibition of mycelial growth, and 0% refers to no effect on growth. The discussed thirty-five hydrazide–hydrazones were divided into three groups depending on the structure of the aldehyde fragment used. Twenty-nine compounds are derivatives of 4-hydroxybenzoic acid.

The simplest compounds are in the first group (Table 1), which consists of twelve molecules (**1**–**12**) containing unsubstituted phenyl **1**, phenyl linked with azomethine group through ethylene linker **2**, monosubstituted phenyl **3**–**11**, and the 5-nitro-2-furylaldehyde derivative **12** (Nifuroxazide). The substituents are 4-CH_3_, 4-isopropyl, 2-SO_3_Na, and differently localized OH and OCH_3_ groups. The more complex second group, shown in Table 2, contains ten disubstituted molecules (**13**–**22**), derivatives of vanillic aldehyde and salicylic aldehydes with OH, Br, C_6_H_4_, *t*Bu, and OCH_3_ substituents. Thirteen derivatives (**23**–**35**) with three or more substituents were assigned to the most complicated third group, which are formally salicylic aldehyde derivatives with OH, OCH_3_, CH_2_OH, CH_3_, isopropyl, and *t*Bu, as given in Table 3.

The determined direct antimicrobial activities were compared between groups 1 and 3. Further comparison concerned antifungal activity and laccase inhibition constants previously reported for compounds **1**–**3**, **6**–**10**, **17**–**19**, **21**, **27**, **30**–**31**, and **33** [47,48] and determined in this study for phloroglucinol derivative **32**. For the preliminary studies, the concentration of 50 μg/mL was admitted to the test.

The hydrazide–hydrazones from the first group are inactive toward tested microorganisms (Table 1). However, some residual activities against white-rot fungus *C. unicolor* were observed in the case of molecules **4**, **5**, and **9**, which inhibited mycelium growth in the range of 20.9–23.6% at the tested concentration. Generally, these molecules have larger *i*Pr or SO_3_Na and OCH_3_ substituents in benzylidene units localized at opposite or near imine linkers, respectively. In our test, nifuroxazide (**12**) showed no activity toward the molds, and to a small extent, it reduced the growth of *C*. *unicolor* by 19.5%. Nifuroxazide is typically used to cure bacterial infections against bacteria that cause travelers’ diarrhea and colitis [74]. The results of antimicrobial activity correlate with the results of laccase inhibition tested in our previous study [48] on the enzyme from white-rot fungi *Trametes versicolor*. Generally, these simple, unsubstituted, monosubstituted phenylidene fragments in hydrazide–hydrazones in the first group are weak inhibitors and antifungal agents.

In the second group of tested hydrazide–hydrazones, two molecules numbered **18** and **19** derived from 4-hydroxybenzoic acid showed the highest activity against tested microorganisms (Table 2). These have salicylaldehyde units having hindered *tert*-butyl or bulky phenyl substituents near the hydroxy group. Both compounds showed relatively strong antifungal activity against *B. cinerea* (82.1 and 73.5%) and *C. unicolor* (85.9 and 71.4%). Furthermore, the *tert*-butyl-salicylic aldehyde derivative was the most fungicidal and almost completely inhibited the growth of *S. sclerotiorum*. Such a significant reduction in the growth of tested microorganisms makes this compound a highly nonselective antifungal agent. In contrast, in the same group, the compound numbered **22** presented high activity toward solely *C. unicolor*. Interestingly, this compound is a derivative of 2,4-dihydroxybenzaldehyde and benzoic acid hydrazide and showed better selective activity when compared to 4-hydroxybenzoic acid derivative **21**. Furthermore, in this group, the hydrazide–hydrazones **14** and **15** derivatives of a salicylic aldehyde with bulky bromine opposite to the hydroxy group and methoxy group near imine linker, respectively, present a selectivity against Basidiomycetes fungus as *C. unicolor* acting as moderate antifungal agents. Among the tested compounds in the more complex second group, a high correlation between antifungal activity and the potency of inhibition of laccase activity is apparent. The most potent inhibitors with hindered 3-*tert*-butyl and bulky 3-phenyl groups, **18** and **19**, respectively, are also strong antifungal agents, and those that did not inhibit the enzyme are also weak or moderate antifungal compounds.

In the third group, the most active and simultaneously non-selective is compound **30**—a derivative of 3-*tert*-butylsalicylidene **18** with the presence of methyl, the smallest alkyl group in the 5th position of aldehyde fragment. Similarly, it almost completely inhibited the growth of *S. sclerotiorum* (96.8%), and it is the most promising antifungal agent against *C. unicolor*. Similar compounds derived from 4-methoxybenzoic acid **31**, benzoic acid **33**, and nicotinic acid **34** showed no antifungal activity against molds and residual activity against white-rot fungi. Interestingly, the total replacement of the acyl unit in **33** and **34** on the acetyl unit in **35** slightly improved activity against *C. unicolor*. Compounds **24**–**26**, having differently localized hydroxymethyl group and methyl or methoxy groups at the salicylidene unit, showed moderate and reasonable activity toward *C. unicolor*. Replacing methyl, the smallest alkyl group in compound **30**, on the second bulky *tert*-butyl group in compound **27** slightly decreased activity against *S. sclerotiorum*; nonetheless, it improved the selectivity among all tested fungi. Noteworthy, compound **28**, a derivative of thymol formylated near the hydroxy group, has potent activity against *S. sclerotiorum* (also moderately active toward *B. cinerea*) with a complete lack of inhibitory activity toward laccase from *Trametes versicolor* [48].

**Table 3:** In vitro fungicidal activities of target hydrazide–hydrazones **23**–**35** at a basal concentration of 50 µg/mL expressed as a fungal growth inhibition (%), mean (±SD) ^a^; n.o.—inhibition effect not observed.

No.	Structure	*B. cinerea*	*S. sclerotiorum*	*C. unicolor*	*K*_i_ [μM]	Ref. *K*_i_
**23**	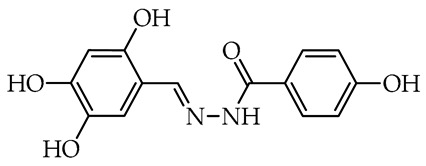	6.8 ± 2.5	2.7 ± 0.4	5.8 ± 2.7	– ^b^	–
**24**	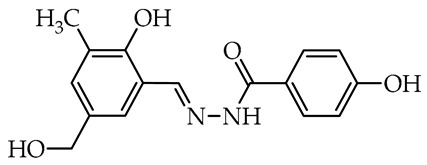	n.o.	n.o.	42.0 ± 2.1	≥1000	[48]
**25**	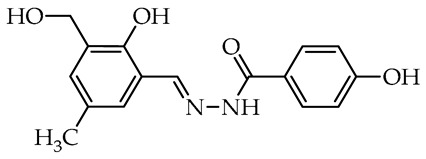	2.8 ± 1.9	n.o.	70.1 ± 1.8	≥1000	[48]
**26**	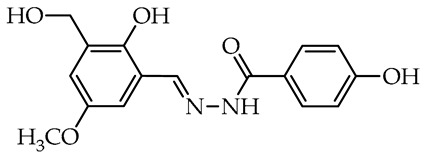	3.2 ± 0.5	n.o.	54.6 ± 3.1	– ^b^	–
**27**	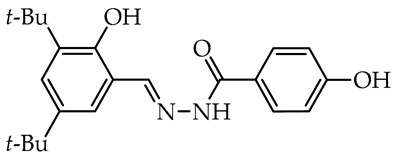	23.4 ± 2.7	**85.5 ± 3.2**	**42.8 ± 2.2**	17.9	[48]
**28**	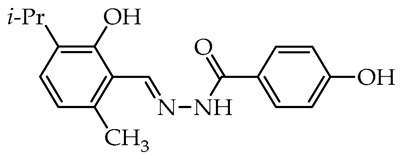	**50.8 ± 2.6**	**91.4 ± 2.2**	38.2 ± 2.3	≥1000	[48]
**29**	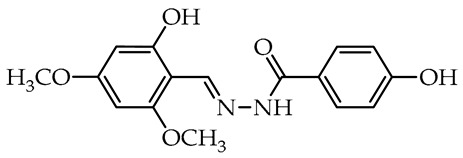	8.1 ± 0.6	n.o.	9.1 ± 1.9	≥1000	[48]
**30**	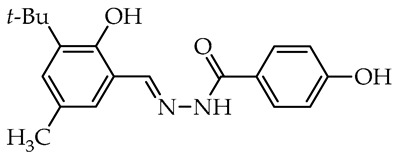	**76.2 ± 2.2**	**96.8 ± 2.2**	**89.8 ± 0.4**	26.4	[48]
**31**	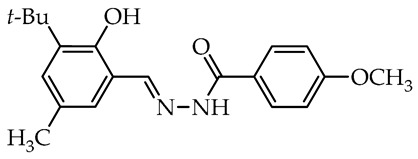	2.1 ± 0.9	n.o.	13.8 ± 3.9	25.8	[47]
**32**	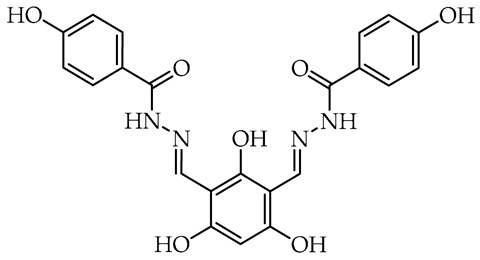	n.o.	n.o.	14.3 ± 2.7	194 ^c^	[75]
**33**	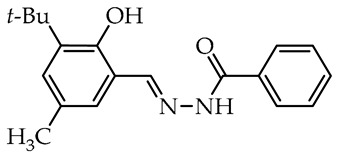	3.9 ± 0.6	n.o.	36.6 ± 1.2	82.0	[47]
**34**	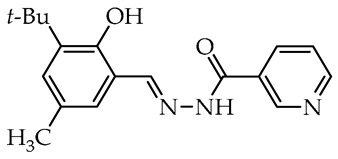	7.5 ± 0.6	n.o.	29.6 ± 2.7	– ^b^	[47]
**35**	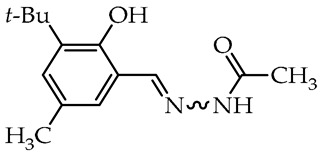	n.o.	n.o.	45.7 ± 0.5	≥1000	[47]
Fenhexamid	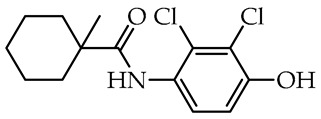	99.9 ± 0.4	100 ± 0.3	39.9 ± 0.7	– ^b^	–

^a^ Results were calculated as the percent of microorganism growth inhibition in the presence of 50 μg/mL of tested compounds in potato dextrose agar (PDA) medium compared to 0.5% (*v*/*v*) methanol-treated control; the result is expressed as the mean of three determinations. ^b^ not determined; ^c^ SD = 5.75 (%) for a test of laccase from *Trametes versicolor* inhibition on 2,4-diformylphloroglucinol derivative **32** *K*_i_ determination [75]. Bold values refer to compounds selected for detailed IC50 testing—the criteria are at least 40% inhibition of mycelial growth and such activity against at least two pathogens.

**Figure 2:**
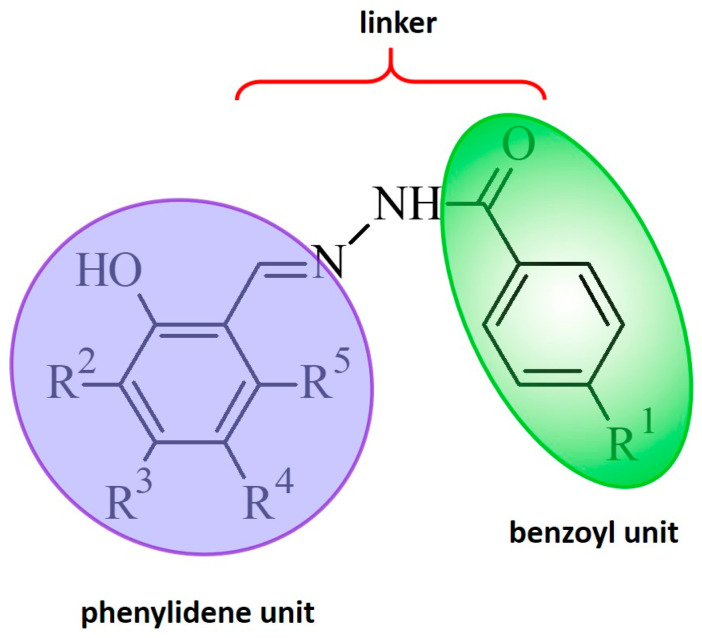
The general structure of the potent hydrazide–hydrazones acting on fungi. A fragment of the salicylidene unit (violet) and the benzoyl unit (green) are derived from benzoic acids, and the hydrazine linker (–C=N–NH–C(O)–) originates from hydrazine. R^1^ = H, OH; R^2^ = H, *i*Pr, *t*Bu, Ph, CH_2_OH; R^3^ = H, OH; R^4^ = H, CH_3_, *t*Bu, OCH_3_, Br; R^5^ = H, CH_3_.

We selected compounds for further testing based on the preliminary results of antifungal activity at a concentration of 50 µg/mL, with more than 40% growth inhibition for at least two microorganisms (see bolded values in Table 2 and Table 3). The IC_50_ values were determined by estimating parameter values of the log-logistic model along with goodness of fit (R^2^ and RMSE), as shown in Table 4. The IC_50_’s values were calculated only when the model predicted a decrease in growth rate by more than 50%. Of the hydrazide–hydrazones tested, five hydroxybenzoic acid derivatives, **18**, **19**, **27**, **28**, and **30**, were selected, and the results of their antifungal activity were compared with those obtained for the reference fenhexamid (fungicide). The most active compounds are those in the second and third groups, which include derivatives of 4-hydroxybenzoic acid and functionalized salicylic aldehydes, as presented in Figure 2. The benzoyl unit from benzoic acid hydrazides and salicylidene unit from salicylic aldehydes, used in the synthesis of the potent hydrazide–hydrazones toward fungi, were indicated in the green and violet frame, respectively.

The salicylic aldehyde fragment, in active molecules, always contains a larger substituent, such as isopropyl, *tert*-butyl, and phenyl, at position 3 (near the hydroxy group). Molecules **18**, **19**, **28**, and **30** have IC_50_ values in the range of 13.9–62.5 µg/mL against *B. cinerea*, which are two orders of magnitude lower when compared to the reference compound. Compounds **18**, **19**, and **30** show similar 13.5–20.3 µg/mL activity against *C. unicolor*. On the contrary, three of the five tested compounds, **18**, **27**, and **30** with bulky *tert*-butyl show the most promising 0.5–1.8 µg/mL activity against *S. sclerotiorum*, which is at least twice as high as fenhexamid fungicide. Of particular interest is hydrazide–hydrazone **18** with simple 3-*tert*-butylsalicylidene unit, which is active against all plant pathogens tested, while more substituted compounds **27** and **28** show high activity against the white mold fungus (Table 4).

The profiles of anti-*B. cinerea*, *-S. sclerotiorum*, and *-C. unicolor* activity for tested hydrazide–hydrazones derivatives of 4-hydroxybenzoic acid and fenhexamid (control fungicide) were illustrated in Supplementary Materials (see Figure S129a–c). Herein, we provide some insight into the relationship between the hydrazide–hydrazones concentrations applied and the inhibition potency of fungi growth in comparison to fenhexamid. Four concentrations of hydrazide–hydrazones, such as 6.25, 12.5, 25.0, and 50.0 µg/mL, were subjected to the estimation of the fungicidal profiles for **18**, **19**, **27**, **28**, **30**, and fenhexamid. After analyzing the data, it is apparent that, generally, the most susceptible microorganism for tested compounds was *S. sclerotiorum* (Table 4, Table S3 and Figure S129b). Even the lowest tested concentration (6.25 µg/mL) of the **18**, **27**, and **30** molecules brought relatively high antifungal activity between 70.6 and 87.6%, compared to 95.6% of fenhexamid. A two-fold increase in the concentration of the hydrazide–hydrazones **18** and **30** showed their activity similar to fenhexamid, 96.3, 98.7, and 96.3%, respectively, approaching almost total inhibition of *S. sclerotiorum* growth at 50 µg/mL. Although derivative **27** presented a high 82.9% of *S. sclerotiorum* inhibition at the lowest investigated concentration, it strengthened the further effect to 93.1% only at 50 µg/mL. All discussed compounds (**18**, **27**, and **30**) are derivatives of 3-*tert*-butyl-2-hydroxybenzaldehydes and 4-hydroxybenzoic acid hydrazide. In that context, the presence of the *tert*-butyl group in the 3 position seems important for anti-*S. sclerotiorum* activity. Far less susceptible on tested hydrazide–hydrazones was *C. unicolor* (Table 4 and Table S3, Figure S129c). The smallest concentration inhibited the fungus in the range of 9.2–21.5%. A two-fold increase in the dose enhanced the effect to 21.7–42.2%. At 25 µg/mL, the molecules **18**, **19**, and **30** showed the highest activity, which reached 76.5, 70.5, and 90.5% of *C. unicolor* inhibition at maximal concentration. But again, the hydrazide–hydrazones with 3-*tert*-butyl-5-methyl-2-hydroxybenzaldehyde fragment showed the highest antifungal activity. The highly active mold fungicide, fenhexamid, was weakly active on the fungus, with 32.1% in mycelium inhibition at 50 µg/mL. The least susceptible to tested compounds was *B. cinerea* (see Table 4 and Table S3, Figure S129b). None of the hydrazide–hydrazones reached the activity of fenhexamid, which presented constant very high activity in the whole range of tested concentrations (98.5% inhibition at 6.25% to 100% inhibition at 50 µg/mL). In contrast to previous fungi, the most active compound against *B. cinerea* was a derivative of 3-phenyl-salicylic aldehyde and 4-hydroxybenzoic acid hydrazide **19**. In fact, for this compound, the antifungal activity profile covers the systematic increase of inhibition from 30.1 to 70.1% of fungal development from 6.25 to 25.0 µg/mL, but at 50 µg/mL, only 74.1% was observed. A similar course of the profile could be attributed to molecule **18** but with slightly weaker activity corresponding to 20.2, 56.4, and 64.7%. The remaining compounds showed rather low activity, less than 50% of *B. cinerea* inhibition.

Comparing the discussed results of the antimicrobial activity on the laccase-producing strains—*B. cinerea*, *S. sclerotiorum*, and *C. unicolor*—presented in this work to the results of the enzymatic studies performed on laccase from *Trametes versicolor* published in our previous work ([48], Table 1, Table 2 and Table 3), it is shown that potent laccase inhibitors **18**, **19**, **27**, and **30** are also potent fungicides to the microorganisms producing these oxidoreductases. The presented results also showed some exceptions that do not meet this rule. These concern compounds **31, 33**, and **28**. Compounds **31** and **33** inhibit laccase but do not inhibit the growth of the tested microorganisms. In contrast, the thymol derivative **28** does not inhibit laccase (*K*_i_ > 1000 µM) but selectively inhibits the growth of *S. sclerotiorum* IC_50_ = 9.8 µg/mL. Therefore, further research is needed to clarify these phenomena. These compounds will be the subject of future experimental studies to determine the mechanism of action of this class of compounds. Concerning the last hydrazide–hydrazone (**28**), it is worth focusing on the thymol molecule. So far, unsubstituted thymol has been the subject of research due to its advantageous properties used in the food industry and traditional medicine and its activity against bacteria and fungi [76]. In studies of unsubstituted thymol on *Fusarium*, *Penicillium*, and *Aspergillus* species, it inhibits mycelium growth at only high concentration (1 mg/mL) [28]. In later detailed studies, the determined antifungal activity (MIC—*minimum inhibitory concentration*, µg/mL) of thymol on plant pathogens were 300 µg/mL for *B. cinerea* and *Fusarium oxysporum* Schltdl., 400 µg/mL for *Alternaria alternata* (Fr.) Keissl, and 450 µg/mL for *Rhizopus oryzae* [77]. Recent studies on the antifungal activity of thymol have shown that it is a promising chemosensing agent for various fungi, and combined treatment with commercial fungicide allowed for a lower dosage of chemical treatment [78]. Thus, thymol derivatives represent an interesting and noteworthy research area for discovering new fungicides.

The search for new compounds active against plant pathogens is still socially and economically necessary. This phenomenon results from increasing resistance to the use of plant protection products and the need to maintain high yields of crops, the quality of ornamental plants, and the protection of plants forming forest ecosystems, parks, etc. One group of compounds under recent investigation as antifungal agents against plant pathogens producing laccase is hydrazide–hydrazones with various molecular architectures and potency against fungal phytopathogens, including quinoline, triazole, tetrahydro-*β*-carbolino-3-carbohydrazide, echinospine, indole diketopiperazine, or rosin-based hydrazide–hydrazones [79]. Although there is no leading motif in the design of fungicides, research indicates that hydrazide linker is essential for biological activity. The literature review indicates that most research has only screening characters performed at elevated compound concentrations, 25, 50, or 100 µg/mL, in which the basal concentration of 50 µg/mL is the most common. Quinoline acylhydrazones containing 4-ethylbenzaldehyde fragment at 100 µg/mL showed weak antifungal activity of 72.49% and 39.57% growth inhibition for *S. sclerotiorum* and *B. cinerea*, respectively [80]. The ((1-(2-chlorophenyl)-1*H*-1,2,3-triazol-4-yl)methylene)benzohydrazide tested against *S. sclerotiorum* was inactive at 25 µg/mL [81]. The derivatives of 2-bromobenzaldehyde and 1,2,4-triazole-acyl-hydrazone containing the quinazoline-4-one showed more than 60% of *B. cinerea* inhibition growth [82]. For tetrahydro-*β*-carbolino-3-carbohydrazide, two derivatives, *N*′-(4-*tert*-butyl-benzylidene)-(3*S*)-1-methyl-2,3,4,9-tetra-hydro-1*H*-pyrido [3,4-b]indole-3-carbohydrazide and *N*′-(3,4-dichloro-benzylidene)-(3*S*)-1-methyl-2,3,4,9-tetrahydro-1*H*-pyrido[3,4-*b*]indole-3-carbohydrazide, showed more than 80% of growth inhibition of *S. sclerotiorum* and *B. cinerea* [83]. The *N*′-(4-{[(4-methylbenzoyl)oxy]imino}cyclohexa-2,5-dien-1-ylidene)benzohydrazide tested on *B. cinerea* showed weak activity restricting mycelium growth of 35% [84]. Some antifungal properties were found for molecules bearing naturally occurring echinospine alkaloid, 1-methyl-4-oxo-1,4-dihydroquinoline-3-carbohydrazide which was combined with substituted phenyl or heterocyclic aldehyde fragments. Two molecules, namely (*E*)-*N*′-(3,4-dimethoxybenzylidene)-1-methyl-4-oxo-1,4-dihydroquinoline-3-carbohydrazide and (*E*)-*N*′-((1*H*-indol-2-yl)methylene)-1-methyl-4-oxo-1,4-dihydroquinoline-3-carbohydrazide, caused more than 80% of *S. sclerotiorum* growth inhibition [55]. In the screening study, the derivatives of indole diketopiperazine with acyl hydrazones containing 4-substituted trifluoromethylbeznaldehyde and *tert*-butylbenzaldehyde fragment as well as 3-chloro and 2,3-dichlorobenzaldehyde fragments exhibited more than 80% and 50% of antifungal activity against *S. sclerotiorum* and *B. cinerea*, respectively [54]. The rosin hydrazide–hydrazones presented weak activity against *S. sclerotiorum* and *B. cinerea*. The most active derivatives showed 25.32% and 32.20% of inhibition growth. Still, the hydrazone derivatives of thiophene aldehydes presented significant activity against another phytopathogenic laccase-producing fungus, *Rhizoctonia solani*, with EC_50_ 0.981 µg/mL [85]. Comparing the results obtained in the above reports for hydrazide–hydrazones applied against fungi causing white (*S. sclerotiorum*) and grey mold (*B. cinerea*), it can be concluded that the hydrazide–hydrazones of naturally occurring 4-hydroxybenzoic acid link with methionine linker with salicylic aldehydes compose scaffolds for designing antifungal compounds. There is no leading motif in the design of fungicides, but research indicates that hydrazide linker is essential for biological activity.

#### 2.2.2. Dicotyledonous Plant Germination Tests

**Figure 3:**
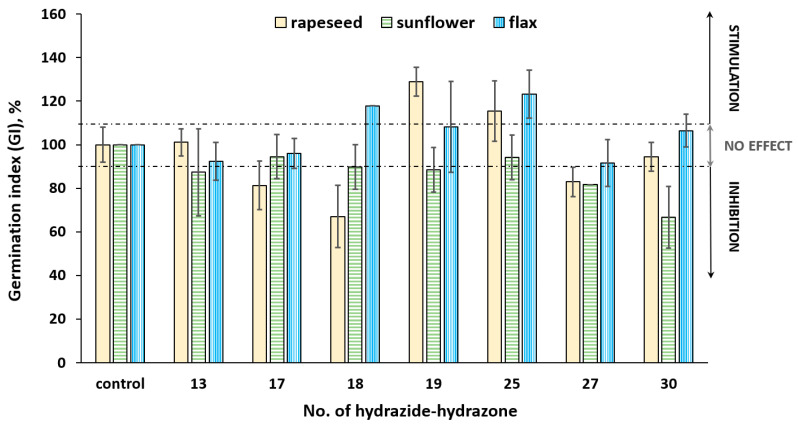
Test 1—Germination index (GI, %) determined for the uncoated seeds of dicot plants: rapeseed (yellow), sunflower (green), and flax (blue). Phytotoxicity tests carried out for hydrazide–hydrazone **13**, **17**, **18**, **19**, **25**, **27**, and **30** with 50 µg/mL concentration; control—the seeds grew on water–ethanol solution 0.5% (*v*/*v*). The results are presented as a mean of three independent repetitions; standard deviation is expressed as error bars.

Among the tested hydrazide–hydrazones derived from 4-hydroxybenzoic acid, we chose seven derivatives of 2- and 4-hydroxybenzaldehydes **13**, **17**, **18**, **19**, **25**, **27**, and **30**. Four of them, **18**, **19**, **27**, and **30**, with a salicylidene framework, are simultaneously the most potent laccase inhibitors and strong antifungal agents. They have the salicylic aldehyde fragment modified with hindered *tert*-butyl or large phenyl substituents in 3-position. The hydrazide–hydrazone **25** has a hydroxymethyl substituent next to the hydroxy group of salicylic aldehyde fragment. This molecule represented the potential against *C. unicolor* without activity toward the laccase enzyme. The last two molecules, **13** and **17**, are derivatives of gentisaldehyde and vanillin aldehyde, respectively. These natural components of both molecules are ubiquitous in the plant kingdom. Therefore, we use them to compare their influence on seed germination and induction of pathogen resistance with the highly potent antimicrobial potential application of our compounds in agriculture and horticulture. The grey and white molds that were studied usually attacked dicotyledons [73]. Thus, we have selected three representatives of temperate climate plants whose seeds produce valuable oils, both for food and industrial purposes. The crops of choice are *Linum usitatissimum* (flax), *Helianthus annuus* (sunflower), and *Brassica napus* var. *napus* (rapeseed) for the test of phytotoxicity (test 1). Moreover, the flax and sunflower seeds were used to evaluate their resistance to *B. cinerea* and *S. sclerotiorum*, respectively (test 2). The results of test 1 were presented in Figure 3 and discussed based on plant germination index (GI, %, for methodology, see Section 3.2.4). Both tests were performed by using 50 µg/mL hydrazide–hydrazones solutions.

According to the adopted methodology [86,87], a germination index (GI, %) ranging from 90 to 110% means no effect of the tested substance on germination and root growth, below 90% means an adverse effect, and above 110% means a favorable effect on germination and root growth. The phytotoxic effect showed six out of seven compounds for at least one kind of seed. Among them, derivative **27** has a GI of 83% and 82% for rapeseed and sunflower, respectively, which are the single compounds that negatively influence two plants. In single cases, the germination index was reduced to 67%, the lowest GI value calculated for rapeseed and sunflower for hydrazide–hydrazone **18** and **30**, respectively, having one *tert*-butyl group. The sensitivity of the dicotyledonous crop seeds tested to the tested hydrazide–hydrazones can be presented as follows: sunflower > rape > flax, where the listed seeds were inhibited by four or three compounds, and for flax seeds, no inhibition effect was observed. No general trend describes the influence of hydrazide–hydrazones tested, but some rules could be applied for at least two kinds of seeds. Surprisingly, hydrazide–hydrazones **13** and **17** with a fragment of naturally occurring aldehydes, syringaldehyde, and salicylaldehyde, respectively, had little negative effect on sunflower and rapeseed with GIs of 87 and 81% (Figure 3). The positive, stimulating effect was observed for **18**, **19**, and **25** molecules. However, derivatives **18** and **19** showed different effects on the seed tested, having no effect on germination and root growth for sunflower and stimulating germination and growth for flax and rape, respectively. Hydrazide–hydrazone **19** is a derivative of 2-hydroxybiphenyl (*ortho*-phenylphenol). The *ortho*-phenylphenol itself is usually used in harvesting fruits to prevent fungal growth. An unexpected result was observed for hydrazide–hydrazone **25**, with the aspect of *C. unicolor* grown inhibition, which was non-toxic for all seeds tested and stimulated the growth of rapeseed and flax.

**Figure 4:**
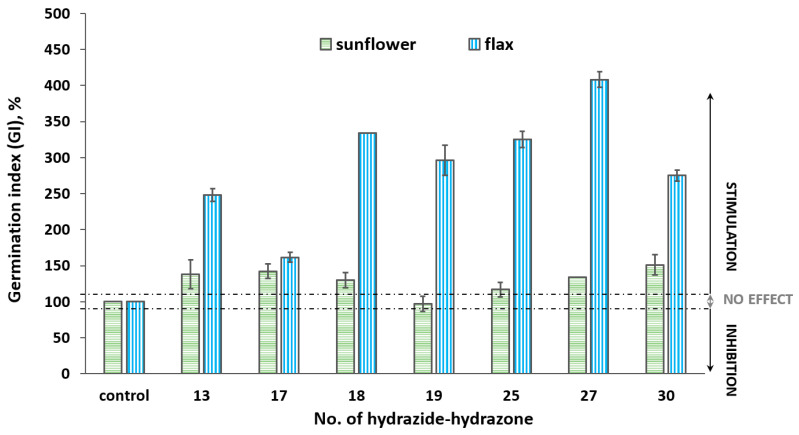
Test 2—Germination index (GI, %) determined for the uncoated seeds of dicot plants: sunflower (green) and flax (blue) infected with *S. sclerotiorum* and *B. cinerea*, respectively. Phytotoxicity tests were performed for hydrazide–hydrazone **13**, **17**, **18**, **19**, **25**, **27**, and **30** with 50 µg/mL concentration; control—the sunflower and flax seeds infected with mycelium of *S. sclerotiorum* and *B. cinerea* grew on water–ethanol solution 0.5% (*v*/*v*). The results are presented as a mean of three independent repetitions.

The second test (test 2) assessed the seeds’ germination and root growth ability infected with the mycelium of the pathogen on soil with a filter in which the corresponding test compound was present. For the test, we selected pairs of plants–pathogens such as sunflower–*S. sclerotiorum* and flax–*B. cinerea*. The results were compared with a control sample that contained soil with a water–ethanol solution (0.5%, *v*/*v*) and seeds infected with a particular mold. The results of test 2 were also expressed in terms of the germination index (%), as shown in Figure 4.

**Figure 5:**
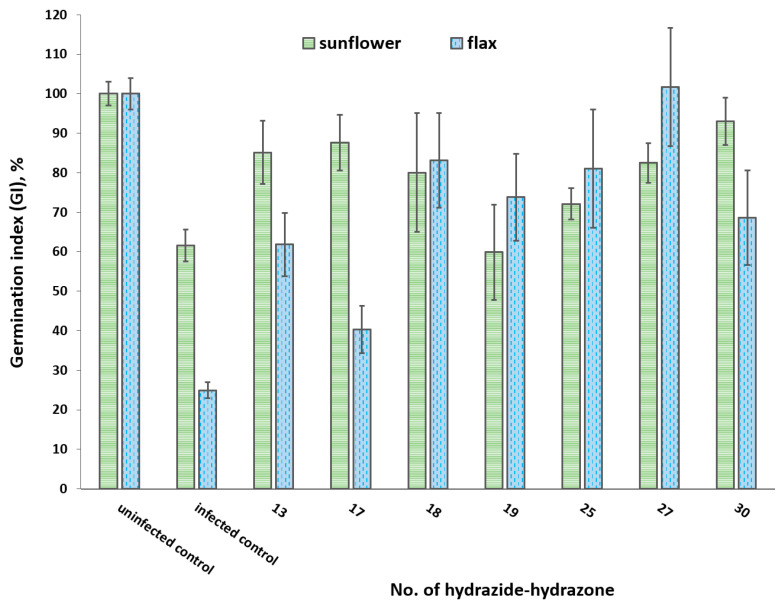
Germination index (GI, %) determined for the uncoated seeds of dicot plants: sunflower (green) and flax (blue); uninfected control—GI determined for uninfected seeds, assumed as 100%; infected control—GI determined for the sunflower and flax seeds infected with mycelium of *S. sclerotiorum* and *B. cinerea*, respectively, grew on water–ethanol solution 0.5% (*v*/*v*); tests performed for sunflower and flax seeds treated with **13**, **17**, **18**, **19**, **25**, **27**, and **30** with 50 µg/mL concentration and infected with *S. sclerotiorum* and *B. cinerea*, respectively. The results are presented as a mean of three independent repetitions.

Almost all compounds showed positive effects on GI for sunflower and flax compared to the infected seeds control. The GI for sunflower was between 97 and 151%; meanwhile, for flax, the effect was more pronounced, which is reflected in GI values between 162 and 408%. Furthermore, to see the positive effect on GI for seeds infected with a fungal pathogen, we compare this parameter for uninfected and infected control samples with infected samples treated with a particular hydrazide–hydrazone solution, as shown in Figure 5. In comparing the control sample uninfected with fungal pathogens to the infected control samples, a significant reduction in the germination index of 62 and 25% was apparent for sunflower and flax, respectively. In all cases tested, hydrazide–hydrazones eliminated the negative effect of the pathogen. In the case of 3,5-di-*tert*-butylsalicylidene derivative **27** and 5-methyl-3-*tert*-butylsalicylidene derivative **30** on sunflower and flax, no negative effect of the pathogen was observed (compounds eliminated the negative effect of the pathogen).

#### 2.2.3. Cytotoxicity Studies

The cytotoxicity studies were conducted on normal human breast epithelial MCF-10A and mouse fibroblasts Balb/3T3 cell lines using *cis*-platin and azoxystrobin as positive controls. The calculated IC_50_ values are presented in Table 5, and the illustrations of cytotoxicity profiles are provided in the Supplementary Materials (see Table S4 and Figure S130). To our surprise, despite the presence of a hydrazine toxicophore in the molecules **13** and **17** consisting of unmodified naturally occurred gentisaldehyde (**52**) and vanillin aldehyde (**56**) units, respectively, they did not show cytotoxicity on tested lines. In addition to the salicylic aldehyde fragment, the CH_2_OH and CH_3_ substituents at positions 3 and 5, respectively, in compound **25**, lead to a 3-fold reduction in cytotoxicity compared to *cis*-platin. The naturally occurring gallic acid (**3**,**4**,**5-hba**), 4-hydroxybenzoic acid (**4-hba**), and its hydrazide **36** were non-toxic. All highly fungicidal molecules tested, i.e., **18**, **19**, **27**, and **30**, have the same cytotoxicity as *cis*-platin but at least one order of magnitude less cytotoxic on the mouse cell line than azoxystrobin, a commonly used fungicide in plant protection. Azoxystrobin is semisynthetic, a well-known, widely used pesticide of natural origin, characterized by a broad spectrum of action against plant fungal diseases, and has already been used for almost three decades [88]. Therefore, we assume that all compounds with lower cytotoxicity could be classified for future studies concerning the determination of the mechanism of action and further research on plants in the context of future applications in agriculture.

Summarizing the biological studies, our results showed that a specific group of hydrazide–hydrazones, derivatives of naturally occurring salicylic aldehyde and 4-hydroxybenzoic acid, namely **18**, **19**, **27**, **28**, and **30**, showed promising antifungal activity against phytopathogens. The most susceptible to the tested compounds was *S. sclerotiorum*, for which hydrazones **18** and **30** were shown to exhibit activity close to fenhexamid at 12.5 μg/mL. In phytotoxicity tests, derivative **18** showed no effect on the germination index (GI), while derivative **30** had a negative effect on GI. On the other hand, in a phytotoxicity test on seeds infected with *S. sclerotiorum* mycelium, a positive effect on GI was evident for all tested compounds, as well as the effect of eliminating the pathogen’s negative effect on germination and root growth in the case of derivative **30**. Moreover, all of the aforementioned compounds showed at least an order of magnitude lower cytotoxicity compared to the commonly used fungicide azoxystrobin. With future plans in mind, we believe that compounds with the highest activity against *S. sclerotiorum* are candidates for further research aimed at application in the agricultural sector, mainly in the context of protecting oilseed crops from which edible and industrial oils are currently obtained. It will be crucial to elucidate their mechanism of action and determine the effects of the tested compounds in dose–response assays on plants, as well as further modification of the substituents to improve antifungal activity and lower phyto- and cytotoxicity.

## 3. Materials and Methods

### 3.1. Reagents and Materials

All commercially available chemicals were purchased as pure for synthesis or analytical grade reagents (Sigma-Aldrich, St. Louis, MO, USA; ARMAR, Muligasse, Switzerland; Fluka Hamburg, Germany; Loba Feinchemie AG, Fischamend Austria; POCh Gliwice, Poland) and solvents were primarily used without further purification. In particular, 4-hydroxybenzoic acid hydrazide (**36**), acetic acid hydrazide (**37**), benzaldehyde (**41**), 3-phenylpropionaldehyde (**42**), 4-methylbenzaldehyde (**43**), 4-*iso*-propylbenzaldehyde (**44**), benzaldehyde-2-sodiumsulfonate (**45**), salicylic aldehyde (**46**), 3-hydroxy-benzaldehyde (**47**), 4-hydroxybenzaldehyde (**48**), 2-methoxybenzaldehyde (**49**), 3-methoxy-benzaldehyde (**50**), anisaldehyde (**51**), gentisaldehyde (**52**), vanillic aldehyde (**56**) (LobaFeinchemie), 3-*tert*-butyl-salicylic aldehyde (**57**), 2,4-dihydroxy-benzaldehyde (**59**), 3,5-di-*tert*-butyl-salicylic aldehyde (**64**), nicotinic acid methyl ester (**76**), 4-methoxybenzoic acid (**77**), and benzoic acid (**78**), syringaldazine (SNG, 4-hydroxy-3,5-dimethoxybenzaldehyde azine), guaiacol (2-Methoxyphenol), dimethyl sulfoxide for plant cell culture, laccase from *Trametes versicolor* in lyophilized powder were bought from Sigma-Aldrich. Vanillic aldehyde (**56**) was purchased from Loba Feinchemie. The 5-nitro-2-furfural diacetyl acetal (**69**) and phloroglucinol (**73**) were purchased from Fluka. Inorganics, citric acid monohydrate, and sodium phosphate dibasic dodecahydrate were purchased from POCh. The 99.8 atom % D solvents for NMR spectroscopy, chloroform-*d*_1_ (CDCl_3_), and dimethyl sulfoxide-*d*_6_ (DMSO-*d*_6_) and ethanol (95%) for biological tests were purchased from ARMAR (Muligasse, Switzerland) and were used without further purification.

Methanol was distilled prior to the condensation reaction from Mg element shavings in the presence of I_2_. Analytical TLC was performed on PET foils precoated with silica gel (Merck silica gel, 60 F254) and was made visual under UV light (λ_max_ = 254 nm) or by staining with iodine vapor. Melting points were determined on an Electrothermal IA 91100 digital melting-point apparatus (Sigma-Aldrich, Saint Louis, MO, USA) using the standard open capillary method. FT-IR spectra (4000–400 cm^−1^) were recorded on a 2000 FT-IR (Perkin–Elmer, Manchester, UK) or VERTEX 70V spectrometer (Bruker, Ettlingen, Germany) using a diamond ATR accessory. Absorption maxima are reported in wavenumbers (cm^–1^). ^1^H-NMR and ^13^C-NMR spectra were recorded on Jeol 400yh (Tokyo, Japan) (399.78 MHz for ^1^H and 100.52 MHz for ^13^C) at 295 K. Chemical shifts (δ) are given in parts per million (ppm) downfield relative to TMS, and coupling constants (*J*) are in Hz. Residual solvent central signals were recorded as follows: DMSO-*d*_6_, δ_H_ = 2.500 ppm, δ_C_ = 39.43 ppm; CDCl_3_, δ_H_ = 7.263 ppm, δ_C_ = 77.00 ppm. When measured, DEPT and ATP experiments signals are referred to as (+) or (–). High-resolution mass spectra (HRMS) were recorded on a LCD Premier XE instrument (Waters, Manchester United, UK), and only the [M + H]^+^ or [M + Na]^+^ molecular species were reported.

The literature procedure was adapted for the preparation of hydrazide–hydrazones **1**–**3**, **5**–**11**, **14**–**25**, **27**–**30** [48], **32** [71], **31**, and **33**–**35** [47]; hydrazides **38**–**40** [47]; aldehydes **53**–**55**, **58**, **61**, **62** [48,89], **60** [66], **63** [67], **65** [48,90], **66**, **67 [48]**, and **68** [69]; and phenol **72** [68].

Purity and homogeneity of known compounds were confirmed by measuring their m.p. for **1**–**3**, **5**–**11**, **14**–**21**, **24**, **25**, **27**–**30** [48], **12 [91]**, **31**, **33**–**35** [47], **32** [71], **38** [48], **39** [92,93], **40** [47,93], **60** [94], **63** [67], and **68** [69]; boiling points for **65** [48] and **68** [67]; FT-IR spectra for **3** [48], **22** [95], **25** [48], **38**, **39** [52], **40** [47], and **68** [69]; ^1^H-NMR spectra for **3** [48], **4** [96], **12** [97], **13** [98], **22** [99], **23** [41], **25**, **28** [48], **38**, **39** [52], **40** [47], **60** [66], **65** [48], and **68** [69]; ^13^C-NMR spectra for **3** [48], **4** [96], **22** [95], **25**, **28** [48], **38**, **39** [52], **40** [47], **65** [48], and **68** [69]; and/or HRMS for **3** [48], **4** [96], **22** [95], **25**, and **28** [48] and comparing them with literature data. Two new aroylhydrazide–hydrazones, **26** and **32,** were fully characterized. The position of hydrogen and carbon atoms in the NMR data was determined by supporting the standard dept-135 experiment and by 2D map analysis of Heteronuclear Multiple Quantum Correlation (HMQC), Heteronuclear Single Quantum Coherence (HSQC), Heteronuclear Multiple Bond Correlation (HMBC), and Nuclear Overhauser Enhancement Spectroscopy (NOESY) experiments, if measured. The spectra images of FT-IR and NMR for the following compounds are placed in the Supplementary Materials.

The cultures of *Botrytis cinerea* strain FBc05 and *Sclerotinia sclerotiorum* strain FSc10 were obtained from the collection of the Division of Plant Pathology and Mycology at the Department of Plant Protection of Wroclaw University of Environmental and Life Sciences (Poland). *Cerrena unicolor* (Bull.ex.Fr.) Murr, strain no. 139, originated from the culture collection of the Department of Biochemistry, University of Lublin (Poland). The stock cultures were maintained on potato dextrose agar at +4 °C and periodically transferred to a fresh medium.

### 3.2. Synthesis

#### 3.2.1. General Procedure for the Synthesis of Hydrazide–Hydrazones **1**–**35** [47]

To a mixture of aldehyde **41**–**68** or diacetylacetal **69** (2.0 mmol) and carboxylic acid hydrazide **36**–**40** (2.0–4.0 mmol) in dry CH_3_OH (2.0–100 mL), AcOH (0–200 µL) was added at room temperature (RT) with 2.5–20 h. Then, the resulting solution was gently refluxed during stirring. The reaction progress was monitored by TLC. Then, the reaction was finished after slow cooling to room temperature (RT) and cooled to ca. +4 °C; the reaction mixture was left in a refrigerator (−24 °C) overnight. The crystals formed were collected by filtration with suction, and the filter cake was washed with a frozen mixture of methanol and water (3:2, *v*/*v*) and dried to obtain pure products **1**–**35**. These compounds were fully characterized by melting point and NMR, IR, and HRMS spectra in our previous articles [47,48] or in the present work (compounds no. **4**, **12**, **13**, **22**, **23**, **26**, **28**, **32**). The known compound was identified by comparison of its melting points and FT-IR and/or NMR spectra with literature data. The new products **26** and **32** were fully characterized. For all products, HRMS analyses were measured (see the Supplementary Materials).

*4-Hydroxy-N′-[(E)-(2-hydroxy-3-hydroxymethyl-5-methoxyphenyl)methylidene]benzohydrazide* (**26**). The general procedure starting from 2-hydroxy-3-hydroxymethyl-5-methoxy-benzaldehyde (**63**) (128 mg, 0.70 mmol) [67], 4-hydroxybenzohydrazide (**36**) (106 mg, 0.70 mmol), CH_3_OH (3.5 mL), and AcOH (70 µL) was employed with a 5 h reaction time and left to crystallize in an open vessel to obtain the 4-hydroxy-*N*′-[(*E*)-(2-hydroxy-3-hydroxymethyl-5-methoxyphenyl)methylidene]benzohydrazide (**26**). A pale yellow powder; 215 mg, 0.68 mmol, 97% yield; m.p. 219–220 °C (from CH_3_OH); selected FT-IR (ATR): ν_max_ 3540 (O-H), 3223 (br, N-H, O-H), 3045 (C_Ar_-H), 2987 (C-H), 2834 (C-H), 1626 (C=O), 1602 (N-H), 1582 (CH=N), 1556 (C_Ar_-H), 1509, 1457 (C_Ar_-H), 1305, 1254 (C_Ar_-O), 1176, 1144 (CH_2_-O), 1060, 1035 (CH_3_-O), 955 (N-N), 890, 847, 763, 660, 621, 526 cm^–1^; ^1^H-NMR (400 MHz, DMSO-*d*_6_): δ 12.02 (s, 1H, NH), 11.53 (s, 1H, Ar-2-OH), 10.21 (s, 1H, 4-OH), 8.51 (s, 1H, CH=N), 7.83 (d, ^3^*J* = 8.6 Hz, 2H, H-2,6), 7.04 (d, ^4^*J* = 2.9 Hz, 1H, ArH-4), 6.89 (d, ^4^*J* = 2.9 Hz, 1H, ArH-6), 6.89 (d, ^3^*J* = 8.6 Hz, 2H, H-3,5), 5.15 (t, ^3^*J* = 5.5 Hz, 1H, CH_2_OH), 4.55 (d, ^3^*J* = 5.5 Hz, 2H, CH_2_), 3.74 (s, 3H, OCH_3_) ppm; ^13^C-NMR (101 MHz, DMSO-*d*_6_): δ 162.41 (C=O), 160.97 (C-4), 151.83 (ArC-5), 148.66 (CH=N), 148.48 (ArC-2), 131.06 (ArC-3), 129.77 (C-2,6), 122.98 (C-1), 117.32 (ArC-1), 115.48 (ArC-4), 115.13 (C-3,5), 112.09 (ArC-6), 57.60 (CH_2_), 55.46 (OCH_3_) ppm; HRMS (TOF, MS, ESI): *m*/*z* for C_16_H_16_N_2_O_5_–H_2_O + H^+^ calculated: 299.1026; found: 299.1037; *m*/*z* for C_16_H_16_N_2_O_5_ + H^+^ calculated: 317.1132; found: 317.1141; for C_16_H_16_N_2_O_5_–H_2_O + Na^+^ calculated: 321.0846; found: 321.0847; *m*/*z* for C_16_H_16_N_2_O_5_ + Na^+^ calculated: 339.0951; found: 339.0951.

*N*′,*N″*-[(2,4,6-trihydroxy-1,3-phenylene)di-(*E*)-methanylylidene]bis(4-hydroxybenzohydrazide) (**32**) [71]. The hydrazide–hydrazone **32** was prepared using a modified literature procedure [71]. The general procedure starting from 1,3-diformylo-2,4,6-trihydroxybenzene (**68**) (0.18 g, 1.0 mmol) [69], 4-hydroxybenzohydrazide (**36**) (304 mg, 2.0 mmol), CH_3_OH (10 mL), and AcOH (0.10 mL) was employed with a 20 h reaction time, with formation carmine red mixture to obtain the *N*′,*N*″-[(2,4,6-trihydroxy-1,3-phenylene)di-(*E*)-methanylylidene]bis(4-hydroxybenzohydrazide) (**32**). A brown red powder; 428 mg, 0.95 mmol, 95% yield; m.p. 223–226 °C (from CH_3_OH) with decomposition (m.p. 223–226 °C with decomposition [71]); selected FT-IR (ATR): ν_max_ 3173 (br, OH, NH), 3074 (C_Ar_-H), 3024 (C_Ar_-H), 1604 (br, C=O, C=N), 1502, 1443, 1325, 1238 (br, C-O), 1171, 1056, 843, 756, 611, 515, 473 cm^−1^; ^1^H-NMR (400 MHz, DMSO-*d*_6_): δ 13.33 (s, 1H, ArC-2-OH), 11.87 (s, 4H, ArC-4,6-OH, 2 × CONH), 10.17 (s, 2H, C-4,4′-OH), 8.84 (s, 2H, CH=N), 7.82 (d, ^3^*J* = 8.7 Hz, 4H, H-2,2′,6,6′), 6.88 (d, ^3^*J* = 8.7 Hz, 4H, H-3,3′,5,5′), 5.99 (s, 1H, ArH-5) ppm; ^13^C-NMR (101 MHz, DMSO-*d*_6_): δ 162.03 (2 × C=O), 161.25 (2 × C–ArC-4,6), 160.86 (2 × C–C-4,4′), 159.89 (C–ArC-2), 145.01 (2 × CH=N), 129.63 (4 × CH–C-2,2′,6,6′), 123.12 (2 × C–C-1,1′), 115.16 (4 × CH–C-3,3′,5,5′), 99.21 (2 × C–ArC-1,3), 94.46 (CH) ppm; HRMS (TOF, MS, ESI) *m*/*z* for C_22_H_18_N_4_O_7_ + H^+^ calculated: 451.1248; found: 451.1261.

#### 3.2.2. In Vitro Antifungal Activity

Prior to the fungicidal activity assay of hydrazide–hydrazones **1**–**35**, we considered ethanol and DMSO as potential dissolution media for tested compounds. Most of the hydrazide–hydrazones have poor solubility in water. Furthermore, a very important aspect of biological testing is the careful selection of organic solvents. These are typically used, e.g., methanol, ethanol, acetone, and DMSO, and may be highly toxic for microorganisms in even very low concentrations, such as 1% (*v*/*v*) [100]. Ethanol and DMSO with a final concentration between 0.1 and 1.0% (*v*/*v*) in a PDA medium were used and applied to evaluate the inhibition growth of *B. cinerea*, *S. sclerotiorum*, and *C. unicolor* (see Supplementary Materials). The following procedure was applied to the microorganism testing. The assays of fungi inhibition growth were performed at a basal concentration of 50 µg/mL for all compounds, and detailed experiments were extended for testing between 0 and 50 µg/mL (0, 6.25, 12.5, 25.0, and 50.0 µg/mL) for selected compounds. The tests were carried out on Petri dishes (diameter 60 mm) with PDA medium containing appropriate compound, co-solvent control, and hydrazide–hydrazone dissolved in co-solvent assay. A piece of mycelium with a 5 mm edge taken from the outer edge of the growing fungi on the PDA plate with co-solvent was transferred to a central part of the PDA plate with the examined compound. The plates thus inoculated were incubated at 22 °C in the dark for a time when the control plate containing only the co-solvent was covered entirely with the tested microorganism. For *B. cinerea* and *S. sclerotiorum*, the test took 3 days, and for *C. unicolor*, it took 7 days. Each assay was performed at least in triplicate. The dose–response relationship between the concentration of tested chemicals and the growth of three chosen fungi species was described by log-logistic Equation (1) [101,102]:

(1) $$A=\left(c+\frac{d-c}{1+exp(b(\ln\left(x\right)-ln(e)))}\right)\times 100\%$$

where A is the area of the culture medium overgrown by a given species of fungus expressed as a percentage of area overgrown by control [%], x is the mean (three determinations) tested substance concentration in the medium [μg/mL] or [%] in case of ethanol and DMSO, e is the inflection point [μg/mL] or [%], b is the threshold parameter [–], and c and d are the lower and upper bound of A, respectively [%]. Model parameters c and e were estimated using the nonlinear least-squares method. Parameters d and b (with two exceptions) were fixed and assumed to be equal. Based on estimated parameters, the values of IC_50_ were determined. When the dose did not reach 50% of microorganism growth inhibition, the IC_50_ value was not defined (n.d.). Estimated parameter values of the log-logistic model, along with calculated IC_50_ and goodness of fit, are given in Table 4. The IC_50_ values were calculated only in cases when the model predicted a decrease in growth rate by more than 50%. The low value of R^2^, e.g., 0.13 for **27** and *S. sclerotiorum*, indicated a small difference between the regression line and the simple average, which was noted when increasing concatenations of chemicals hardly influenced the toxic effect, and therefore, the data points were arranged close to the horizontal line reflecting their average value. The model parameters were not estimated for ethanol influence on *S*. *sclerotiorum* growth because no signs of inhibition were observed, even in the highest ethanol concentrations (see Supplementary Materials, Tables S2 and S3).

#### 3.2.3. Kinetic Study for **32**

The inhibition constant for **32** was determined according to procedures and calculations reported previously [47,48] using commercially available laccase from *Trametes versicolor* (Sigma-Aldrich).

#### 3.2.4. Phytotests

In the experiments with plants, two aspects were taken under consideration: (1) phytotoxicity, which determined the toxicity of the hydrazide–hydrazones on seed germination and root growth, and (2) study on the behavior of seeds in the presence of the tested hydrazide–hydrazones and pathogens producing laccase (*B. cinerea* and *S. sclerotiorum*). The non-coated seeds of Linum usitatissimum, Helianthus annuus, and Brassica napus var. napus were provided by the Institute of Soil Science and Plant Cultivation, National Research Institute in Pulawy, Department of Weed Science and Tillage Systems, and Institute of Technology and Life Sciences–National Research Institute Falenty (Poland). Both phytotests were performed based on direct-contact microbiotests following the protocol recommended by the manufacturer (PhytotestkitTM, MicroBio Tests, Mariakerke, Belgium) with the authors’ modifications.

The main phytotest equipment (Tigret, Warsaw, Poland) and general methods are described as follows. Test plates (21 × 15.5 × 0.8 cm) provide optimal moisture and enable observation of seed germination and data reading at the end of the test. A single plate is made of polyvinylchloride and composed of both a transparent bottom and a covering part that are stuck together by a set of press buttons. The bottom part is separated horizontally with a middle ridge into two equal shallow rectangular areas, in which the lower area was filled with 90 mL of roasted sand and 30 mL of the ethanol solution of the tested compound. In tests (1) and (2), the selected hydrazide–hydrazones were dissolved to 50 µM in the water–ethanol solution 0.5% (*v*/*v*) and distributed uniformly into the sand. Such prepared wet support was covered with a black filter paper that soaked the solution and was applied for further testing.

In the assay of phytotoxicity (1), the seeds of three dicota plants were used, namely ten seeds of flax or rapeseed and six seeds of sunflower. The seeds were transferred directly on the filter surface by placing them along the line at approximately 1 cm from the ridge at equal distances. Then, the seeds on the filter surface were covered with a covering part and placed in a holder.

In assay (2), the direct interaction between seed-microorganism-tested hydrazide–hydrazone was tested. Before the seeds were transferred on the surface of wet support, they were covered with mycelium of *B. cinerea* or *S. sclerotiorum* for flax or sunflower, respectively. For that purpose, 100 or 50 seeds of flax or sunflower, respectively, were transferred to the Petri dish (90 mm diameter) with a 7-day-old culture and gently shaken. Then, the seeds were placed on the prepared soaked support with the black filter and covered with the upper part of the plate. The choice of type of fungi and seeds was arbitrary since both molds attack both types of plant.

All test plates were put vertically in a special incubator to allow free germination and growth of roots at 25 °C through 5 days in the dark. The test was performed in triplicate for each test plant. The results were expressed as the germination index values (GI, Equation (2)) concerning the plant growth controls in water–ethanol solution for (1). The results obtained for (2) were also related to the control obtained in (1) test. The GI was calculated from the formula and categorized as an inhibition for GI values < 90%, as stimulation > 110%, and between 90 and 110% as non-toxic/no effect as proposed in the literature [86,87].

(2) $$GI,\%=\frac{Gs\times Ls}{Gc\times Lc}\times 100\%$$

where Gs is seed germination (%), Ls is root elongation (mm) for the test plate, and Gc and Lc are corresponding values for the control plates. Gc was calculated as the proportion of germinated seeds in the test in relation to the number of germinated seeds in the control. The control was the representative number of seeds that germinated on a medium with 0.5% (*v*/*v*) of ethanol.

#### 3.2.5. Cytotoxicity

##### Cell Lines

Antiproliferative tests were performed on a normal human breast cell line, MCF-10A, and a mouse embryonic fibroblast: Balb/3T3. Both cell lines were obtained from the American Type Culture Collection (Rockville, MD, USA). The cell lines were maintained in the Institute of Immunology and Experimental Therapy, Wroclaw, Poland. MCF-10A cells were cultured in the F-12 nutrient mixture (Gibco, Scotland, UK), supplemented with 5% horse serum (Gibco, Scotland, UK), 10 μg/mL of cholera toxin (*Vibrio cholerae* Pacini), 10 μg/mL of hydrocortisone and 20 ng/mL of human epidermal growth factor (all from Sigma-Aldrich, Chemie GmbH, Steinheim, Germany). Balb/3T3 cells were cultured in Dulbecco medium (Gibco, Darmstadt, Germany) supplemented with 10% fetal bovine serum (Thermo Fisher Scientific, Waltham, MA, USA) and 2 mM l-glutamine (Sigma-Aldrich, St. Louis, MO, USA). All culture media contained antibiotics: 100 U/mL penicillin (Sigma-Aldrich, St. Louis, MO, USA) and 100 µg/mL streptomycin (Polfa-Tarchomin, Warsaw, Poland). Both cell lines were cultured during the entire experiment in a humid atmosphere at 37 °C and 5% CO_2_. Amistar 250 SC (fungicide) with active compound azoxystrobin was provided by the Institute of Soil Science and Plant Cultivation, National Research Institute in Pulawy, Department of Weed Science and Tillage Systems in Wroclaw, Poland in a humid.

##### Antiproliferative Assay In Vitro

Twenty-four hours prior to the applications of the tested compounds, MCF-10A and Balb/3T3 cells were plated in 96-well plates at a density of 10^4^ cells per well and cultured at 37 °C in a humid atmosphere saturated with 5% CO_2_ for 24 h. Stock solutions of the compounds were prepared *ex tempore* for each test by dissolving in DMSO. The cells were also exposed to the reference drugs: *cis*-platin (Accord, London, UK) (10–0.01 µg/mL) and DMSO (Sigma-Aldrich) (at the concentrations corresponding to those in the tested agent dilutions). The solutions were then diluted in culture medium RPMI 1640 + Opti-MEM (1:1) (both from Gibco, Scotland, UK), supplemented with 2 mM l-glutamine, 5% fetal bovine serum (all from Sigma-Aldrich Chemie GmbH, Steinheim, Germany). An assay was performed after 72 h of exposure to the tested compounds **13**, **17**, **18**, **19**, **25**, **27**, **30**, **4-hba**, **4-hbah**, and **3**,**4**,**5-hba** (0.5, 5.0, 50, and to 500 µM) and azoxystrobin fungicide (0.05, 0.5, 5.0 and 50 µM). The antiproliferative effect in vitro was determined by the SRB method [103] (sulforhodamine B colorimetric assay). The optical densities of the samples were measured on the Synergy H4 photometer (BioTek Instruments, Winooski, VT, USA) at 540 nm. The results were calculated as 50% inhibitory concentration (IC_50_), namely the dose of the tested compound inhibiting proliferation of the tested cells by 50% as compared to the untreated control cells, in the Prolab-3 system based on Cheburator 0.4 software using the two-point method [104]. The mean values of ID_50_ for each experiment ± SD are presented in Table 5. Each test was repeated 3–5 times.

## 4. Conclusions

In the present article, we presented the results of the biological evaluation of hydrazide–hydrazones, derivatives of carboxylic acid hydrazides, and aldehydes, which previously were tested as laccase inhibitors. We postulated that the laccase might be the target enzyme produced by the phytopathogenic fungi, and inhibition may prevent or slow pathogen development in infected plants. We performed screening tests of all hydrazide–hydrazones at 50 µg/mL on laccase-producing fungi, *Botrytis cinerea*, *Sclerotinia sclerotiorum*, and *Cerrena unicolor* and further detailed tests to determine IC_50_ parameters. Among the tested molecules, derivatives of 4-hydroxybenzoic acid and salicylic aldehydes, namely 3-*tert*-butyl-, 3-Ph-, 3,5-di-*tert*-butyl, and 3-*tert*-butyl-5-methylsalicylic aldehyde (**18**, **19**, **27**, and **30**, respectively), showed high antifungal activity, which corresponded to their high laccase inhibition potency [48]. The most susceptible phytopathogen among tested laccase-producing fungi was *S. sclerotiorum*, for which the determined IC_50_ was up to 0.5 µg/mL. Good results were also obtained for the thymol derivative **28**, which was not active against laccase but showed high potency on *S. sclerotiorum* with IC_50_ 9.8 µg/mL. We also performed tests of phytotoxicity and cytotoxicity on selected hydrazide–hydrazones to determine their potential future application in the plant protection sector. Most of the tested molecules showed low or no phytotoxicity for tested dicotyledonous plants, and for two compounds, we observed no pathogen effect on the germination index of sunflower and flax seeds. Cytotoxicity results show that our compounds are non-toxic or less toxic than the azoxystrobin fungicide commonly used in agriculture. Therefore, we assumed that the derivatives of salicylic aldehydes and 4-hydroxybenzoic acid, namely **18**, **19**, **27**, **28**, and **30**, are promising antifungal agents that can be further optimized as fungicidal candidates for application in the agriculture of dicot plant produced of oilseed crops used in manufacturing edible and industrial oils, especially for biofuels.

## Figures and Tables

**Table 1:** In vitro fungicidal activities of target hydrazide–hydrazones **1**–**12** at a basal concentration of 50 µg/mL expressed as a fungal growth inhibition (%), mean (±SD) ^a^; n.o.—inhibition effect not observed.

No.	Structure	*B. cinerea*	*S. sclerotiorum*	*C. unicolor*	*K*_i_ [μM]	Ref. *K*_i_
**1**	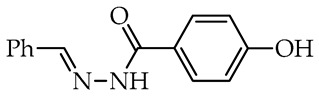	2.6 ± 0.2	n.o.	10.6 ± 1.1	1468	[48]
**2**	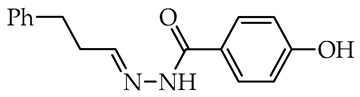	n.o.	n.o.	18.0 ± 3.2	1919	[48]
**3**	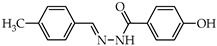	3.3 ± 1.1	n.o.	n.o.	251	[48]
**4**	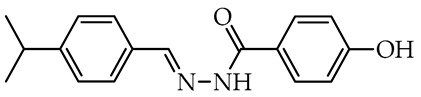	12.0 ± 2.6	n.o.	20.9 ± 2.8	– ^b^	–
**5**	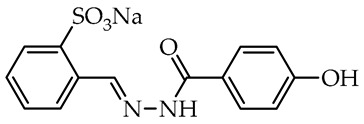	n.o.	n.o.	23.6 ± 4.2	≥2400	[48]
**6**	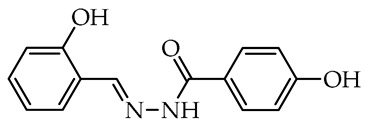	4.7 ± 1.1	n.o.	14.7 ± 0.3	674	[48]
**7**	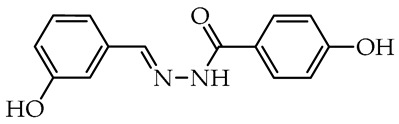	3.7 ± 1.8	n.o.	12.6 ± 4.1	2396	[48]
**8**	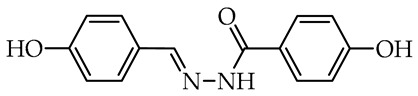	4.2 ± 1.5	n.o.	14.3 ± 1.7	638	[48]
**9**	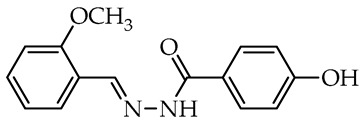	3.3 ± 1.2	n.o.	22.3 ± 2.6	1064	[48]
**10**	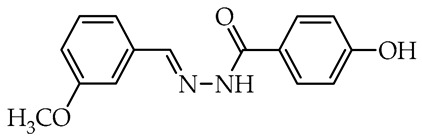	3.9 ± 2.4	n.o.	n.o.	416	[48]
**11**	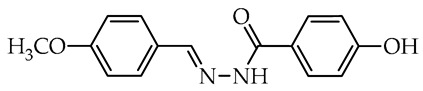	4.4 ± 0.9	n.o.	11.7 ± 3.5	≥2400	[48]
**12**	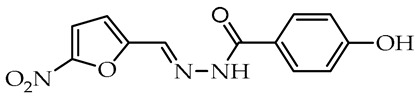	3.3 ± 1.2	n.o.	19.5 ± 2.8	– ^b^	[48]
Fenhexamid	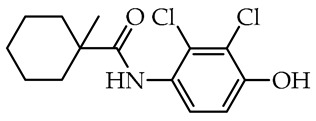	99.9 ± 0.4	100 ± 0.3	39.9 ± 0.7	– ^b^	–

^a^ Results were calculated as the percent of microorganism growth inhibition in the presence of 50 μg/mL of tested compounds in potato dextrose agar (PDA) medium compared to 0.5% (*v*/*v*) methanol-treated control; the result is expressed as the mean of three determinations. ^b^ not determined.

**Table 2:** In vitro fungicidal activities of target hydrazide–hydrazones **13**–**22** at a basal concentration of 50 µg/mL expressed as a fungal growth inhibition (%), mean (±SD) ^a^; n.o.—inhibition effect not observed.

No.	Structure	*B. cinerea*	*S. sclerotiorum*	*C. unicolor*	*K*_i_ [μM]	Ref. *K*_i_
**13**	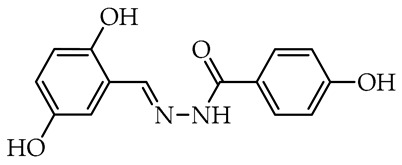	n.o.	n.o.	n.o.	– ^b^	[48]
**14**	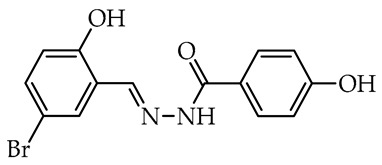	n.o.	n.o.	56.8 ± 8.4	≥1000	[48]
**15**	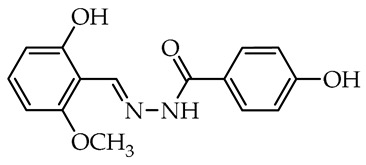	n.o.	n.o.	43.1 ± 3.0	≥1000	[48]
**16**	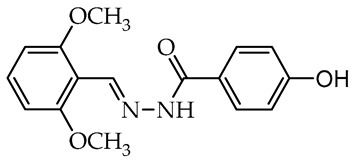	n.o.	n.o.	32.1 ± 3.1	≥1000	[48]
**17**	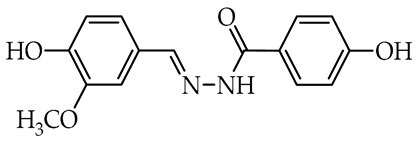	n.o.	7.3 ± 2.1	11.9 ± 0.8	251	[48]
**18**	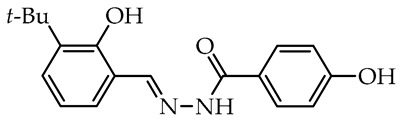	**82.1 ± 3.1**	**97.1 ± 1.1**	**85.9 ± 2.1**	**24.0**	[48]
**19**	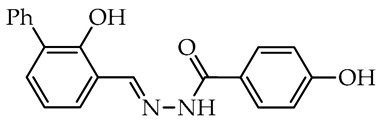	**73.5 ± 3.3**	**50.6 ± 5.5**	**71.4 ± 2.1**	**25.3**	[48]
**20**	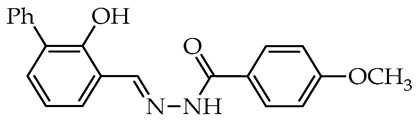	3.3 ± 1.2	n.o.	35.4 ± 2.4	≥1000	[48]
**21**	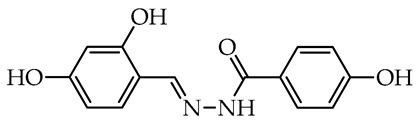	n.o.	n.o.	30.1 ± 4.3	939	[48]
**22**	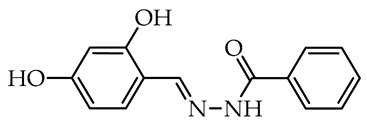	32.0 ± 2.9	n.o.	75.4 ± 6.2	– ^b^	–
Fenhexamid	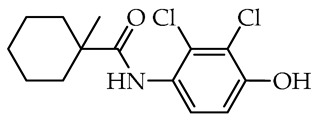	99.9 ± 0.4	100 ± 0.3	39.9 ± 0.7	– ^b^	–

^a^ Results were calculated as the percent of microorganism growth inhibition in the presence of 50 μg/mL of tested compounds in potato dextrose agar (PDA) medium compared to 0.5% (*v*/*v*) methanol-treated control; the result is expressed as the mean of three determinations. ^b^ not determined. Bold values refer to compounds selected for detailed IC_50_ testing—the criteria are at least 40% inhibition of mycelial growth and such activity against at least two pathogens.

**Table 4:** Log-logistic model parameter values for in vitro fungicidal activities, its goodness of fit, and IC_50_s for tested selected hydrazide–hydrazones, derivatives of 4-hydroxybenzoic acid against *Botrytis cinerea* (*Bc*), *Sclerotinia sclerotiorum* (*Ss*), and *Cerrena unicolor* (*Cu*).

No.	Fungus Species	Log-Logistic Model Parameters				Statistical Parameters ^b^		IC_50_^a^	Laccase Inhibition, *K*_i_^c^
		d	b	c	e	R^2^	RMSE	µg/mL	µM
**18**	*Bc*	1	1	14.0 × 10^–1^	14.28	0.94	5.65	20.0	24.0
	*Ss*	1	1	1.66 × 10^–12^	1.80	0.74	6.80	1.8	
	*Cu*	1	1	9.76 × 10^–9^	18.25	0.84	12.92	18.3	
**19**	*Bc*	1	1	2.39 × 10^–2^	13.20	0.96	5.30	13.9	25.3
	*Ss*	1	1	2.74 × 10^–8^	69.86	0.82	12.62	69.9	
	*Cu*	1	1	3.11 × 10^–10^	20.26	0.96	4.54	20.3	
**27**	*Bc*	1	1	7.74 × 10^–1^	0.70	0.13	2.72	n.d. ^d^	17.9
	*Ss*	1	1	1.04 × 10^–1^	0.42	0.43	3.13	0.5	
	*Cu*	1	1	4.98 × 10^–1^	9.51	0.91	3.84	2194	
**28**	*Bc*	1	1	4.59 × 10^–10^	52.68	0.84	9.80	52.7	≥1000
	*Ss*	1	1	2.34 × 10^–10^	9.82	0.92	6.97	9.8	
	*Cu*	1	1	5.82 × 10^–1^	5.85	0.99	0.69	n.d. ^d^	
**30**	*Bc*	1	1	1.50 × 10^–9^	62.48	0.89	6.24	62.5	26.4
	*Ss*	1	1	1.14 × 10^–13^	0.81	0.87	1.72	0.8	
	*Cu*	1	1	3.12 × 10^–10^	13.47	0.85	12.50	13.5	
Control ^e^	*Bc*	1	1	2.31 × 10^–14^	0.14	0.95	0.73	0.1	–
	*Ss*	1	5.108 ^f^	3.00 × 10^–2^	4.19	1.00	3.35	4.2	
	*Cu*	1	1	5.95 × 10^–1^	14.22	0.96	2.47	n.d. ^d^	

^a^ IC_50_ [µg/mL]—a compound concentration that inhibits the growth of fungi by 50% when compared to the reference test without hydrazide–hydrazone. ^b^ R^2^—coefficient of determination; RMSE—root mean squared error. ^c^ Inhibition kinetic constants of laccase from *Trametes versicolor* [48]; ^d^ n.d.—not defined, the dose does not reach 50% of microorganism growth inhibition. ^e^ Control–fenhexamid (fungicide). ^f^ Estimated value.

**Table 5:** Average IC_50_ [µM] ± SD values for selected hydrazide–hydrazone tested on the MCF-10A and BALB/3T3 cell lines.

No.	Structure	MCF-10A Line	Balb/3T3 Line
**13**	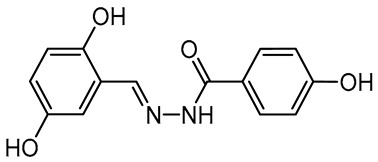	213.26 ± 34.06	132.48 ± 9.22
**17**	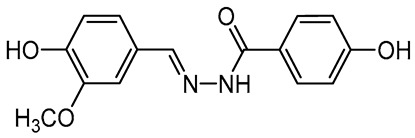	275.12 ± 41.46	216.51 ± 61.25
**18**	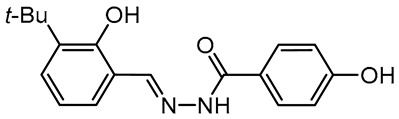	6.41 ± 2.05	11.39 ± 2.54
**19**	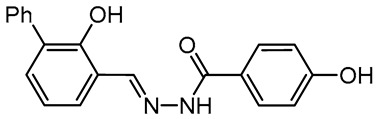	3.44 ± 0.83	3.97 ± 0.15
**25**	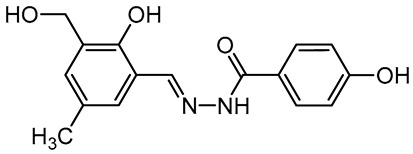	27.48 ± 1.83	27.24 ± 4.30
**27**	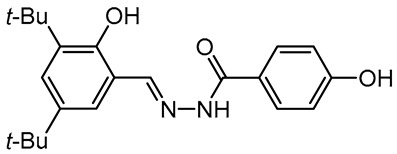	7.73 ± 1.08	11.65 ± 0.69
**30**	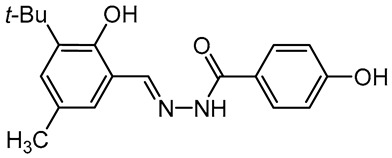	8.85 ± 5.47	23.11 ± 3.87
**4-hba** ^a^	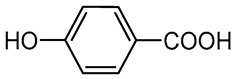	N/A ^b^	N/A ^b^
**36** **(4-hbah)** ^c^	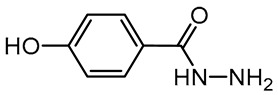	N/A ^b^	N/A ^b^
**3**,**4**,**5-hba** ^d^	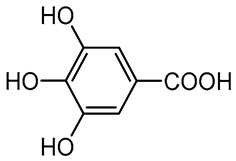	174.11 ± 14.33	163.17 ± 1.95
Azoxystrobin		N/D ^e^	0.10 ± 0.03
*Cis*-platin		9.27 ± 1.63	9.80 ± 1.93

^a^ **4-hba**–4-hydroxybenzoic acid; ^b^ N/A—not active at tested concentration up to 500 µM; ^c^ **4-hbah**–4-hydroxybenzoic acid hydrazide, ^d^ **3**,**4**,**5-hba**–gallic acid; ^e^ N/D—not determined IC_50_.

## Data Availability

The original contributions presented in the study are included in the article/Supplementary Material, further inquiries can be directed to the corresponding authors.

## Supplementary Materials

The following supporting information can be downloaded at https://www.mdpi.com/article/10.3390/molecules29102212/s1. General Methods are in S3–S4; Table S1: Characteristics of the hydrazide–hydrazones **1**–**35** in S5; procedures and spectral data in Figures S1–S128: FT-IR, ^1^H NMR, and ^13^C NMR spectra, dept-135 and 2D experiments of selected hydrazide–hydrazones; **4**, **12** (Nifuroxazide), **13**, **22**, **23**, **25**, **26**, **28**, and **32** and aldehydes **60** (4-hydroxy-gentisaldehyde), **62** (2-hydroxy-3-(hydroxymethyl)-5-methylbenzaldehyde), **63** [2-hydroxy-3-(hydroxymethyl)-5-methoxybenzaldehyde], **65** (3-*iso*-propyl-6-methylsalicylic aldehyde), and **68** (2,4-diformylphloroglucinol) in S7–S74; procedures of hydrazide **38**–**40** preparation in S75. Fungicidal profiles for DMSO and ethanol are in Table S2, and for hydrazide-hydrazones **18**, **19**, **27**, **28**, **30** and fenhexamid are in Table S3 and Figure S129. Cytotoxicity profiles for **13**, **17**, **18**, **19**, **25**, **27**, **30**, **4-hba**, **4-hbah**, gallic acid (**3**,**4**,**5-hba**), azoxystrobin and *cis*-platin are in S79–S83. References [41,47,48,52,66,67,68,69,71,89,90,91,92,93,94,95,96,97,98,99,101,102] are cited in the supplementary materials.
