# Supplementary material for: 4-Hydroxybenzoic Acid-Based Hydrazide–Hydrazones as Potent Growth Inhibition Agents of Laccase-Producing Phytopathogenic Fungi That Are Useful in the Protection of Oilseed Crops
Source: Molecules. 2024 May 8;29(10):2212. doi: 10.3390/molecules29102212 (PMC11124341; doi:10.3390/molecules29102212)
Supplement: Supplementary file 1 [file molecules-29-02212-s001.zip › molecules-2965833-supplementary materials.pdf]

SUPPORTING INFORMATION: Molecules **2024**, *29*,

Title: 4-Hydroxybenzoic Acid-Based Hydrazide-Hydrazones as Potent Growth Inhibition Agents of Laccase-Producing Phytopathogenic Fungi That Are Usefull in the Protection Oilseed Crops

Author(s): Halina Maniak [1]*, Konrad Matyja [1], Elżbieta Pląskowska [2], Joanna Jarosz [3], Paulina Majewska [4], Joanna Wietrzyk [3], Hanna Gołębiowska [5], Anna Trusek [1] and Mirosław Giurg [6]*

[1] Department of Micro, Nano, and Bioprocess Engineering, Faculty of Chemistry, Wroclaw University of Science and Technology, 4/6 Norwida, 50-373 Wroclaw, Poland; konrad.matyja@pwr.edu.pl (K.M.); anna.trusek@pwr.edu.pl (A.T.)

[2] Division of Plant Pathology and Mycology, Department of Plant Protection, Wroclaw University of Environmental and Life Sciences, 24A Grunwald Square, 50-363 Wroclaw, Poland; elzbieta.plaskowska@upwr.edu.pl (E.P.)

[3] Laboratory of Experimental Anticancer Therapy, Hirszfeld Institute of Immunology and Experimental Therapy, Polish Academy of Sciences, 12 R. Weigla Street, 53-114 Wroclaw, Poland; joanna.jarosz@iitd.pan.wroc.pl (J.J.); wietrzyk@iitd.pan.wroc.pl (J.W.)

[4] Institute of Technology and Life Sciences-National Research Institute, 3 Hrabska Avenue, 05-090 Raszyn, Poland; paulina_majewska7@tlen.pl (P.M.)

[5] Department of Weed Science and Tillage Systems, Institute of Soil Science and Plant Cultivation State Research Institute, 61 Orzechowa Street, 50-540 Wroclaw, Poland; hanna.golebiowska1@wp.pl (H.G.)

[6] Department of Organic and Medicinal Chemistry, Faculty of Chemistry, Wroclaw University of Science and Technology, 27 Wybrzeże Wyspiańskiego, 50-370 Wroclaw, Poland

***** Correspondence: halina.maniak@pwr.edu.pl (H.M.); miroslaw.giurg@pwr.edu.pl (M.G.); Tel.: +48713203314 (H.M.); +48713203616 (M.G.)

Table of the content: S2

General Methods	S3-S4
Table S1 – characteristics of the hydrazide-hydrazones **1–35**	S5
General procedure for synthesis of the hydrazide-hydrazones **1–35**	S6
4-Hydroxy-*N'*-[(*E*)-(4-methylphenyl)methylidene]benzohydrazide monometanolate (**3**)	S6
Preparation of hydrazide-hydrazone **4** and its spectral data	S7-S12
Preparation of Nifuroxazide (**12**) and its spectral data	S13-S18
Preparation of hydrazide-hydrazone **13** and its spectral data	S19-S24
Preparation of hydrazide-hydrazone **22** and its spectral data	S25-S31
Preparation of hydrazide-hydrazone **23** and its spectral data	S32-S37
The hydrazide-hydrazone **25** preparation	S38
Preparation of hydrazide-hydrazone **26** and its spectroscopic data	S39-S44
Preparation of hydrazide-hydrazone **28** and its FT-IR spectrum	S45
Preparation of hydrazide-hydrazone **32** and its spectral data	S46-S52
Preparation of 4-hydroxy-gentisaldehyde (**60**), and its spectral data	S53-S58
Preparation of 2-hydroxy-3-(hydroxymethyl)-5-methylbenzaldehyde (**62**)	S59
Preparation of 2-hydroxy-3-(hydroxymethyl)-5-methoxybenzaldehyde (**63**), and its spectral data	S60-S65
Preparation of 3-isopropyl-6-methylsalicylic aldehyde (**65**), and its spectral data	S66-S70
Preparation of 2,4-diformylphloroglucinol (**68**), and its spectral data	S71-S74
General procedure for synthesis of hydrazides **38–40**	S75
p-Anisic acid hydrazide (**38**) preparation	S75
Benzohydrazide (**39**) preparation	S75
Nicotinic acid hydrazide (**40**) preparation	S75
In vitro antifungal activity	S76-S78
Cytotoxicity profiles	S79-S83
References	S84-S85

General Methods

All commercially available chemicals were purchased as pure for synthesis or analytical grade reagents (Sigma-Aldrich St. Louis, MO, USA; ARMAR, Muligasse, Switzerland; Fluka Hamburg, Germany; Loba Feinchemie AG, Fischamend Austria; POCh Gliwice, Poland) and solvents were primarily used without further purification. In particular 4-hydroxybenzoic acid hydrazide (**36**), acetic acid hydrazide (**37**), benzaldehyde (**41**), 3-phenylpropionaldehyde (**42**), 4-methylbenzaldehyde (**43**), 4-*iso*-propylbenzaldehyde (**44**), benzaldehyde-2-sodiumsulfonate (**45**), salicylic aldehyde (**46**), 3-hydroxybenzaldehyde (**47**), 4-hydroxybenzaldehyde (**48**), 2-methoxybenzaldehyde (**49**), 3-methoxy-benzaldehyde (**50**), anisaldehyde (**51**), gentisaldehyde (**52**), 3-*tert*-butyl-salicylic aldehyde (**57**), 2,4-dihydroxybenzaldehyde (**59**), 3,5-di-*tert*-butyl-salicylic aldehyde (**64**), nicotinic acid methyl ester (**76**), 4-methoxybenzoic acid (**77**), and benzoic acid (**78**), syringaldazine (SNG, 4-hydroxy-3,5-dimethoxy-benzaldehyde azine), guaiacol (2-methoxyphenol), dimethyl sulfoxide (DMSO) for plant cell culture, laccase from *Trametes versicolor* in lyophilized powder, 4-methyl-2,6-bis(hydroxymethyl)phenol and hydrazine monohydrate ($H_2NNH_2 \times H_2O$) were bought in Sigma-Aldrich. Vanillic aldehyde (**56**) was purchased in Loba Feinchemie. The 5-nitro-2-furfural diacetyl acetal (**69**) and phloroglucinol (**73**) were purchased in Fluka. 4-Methoxyphenol (**71**), formaldehyde, acetic acid, and inorganics, magnesium (Mg) and iodine (I_2) elements, NaOH, citric acid monohydrate and sodium phosphate dibasic dodecahydrate were purchased in POCh. The 99.8 atom % D solvents for NMR spectroscopy, chloroform-d_1 ($CDCl_3$), and dimethyl sulfoxide-d_6 (DMSO-d_6), and ethanol (95%) for biological tests were purchased in ARMAR and were used without further purification.

Methanol (ARMAR) was distilled prior to the condensation reaction from Mg element shavings in the presence of I_2.

Analytical TLC was performed on PET foils precoated with silica gel (Merck silica gel, 60 F254), and were made visual under UV light (λ_{max} = 254 nm), or by staining with iodine vapor. Melting points were determined on an Electrothermal IA 91100 digital melting-point apparatus (Sigma-Aldrich, Saint Louis, MO, USA) using the standard open capillary method. FT-IR spectra (4000–400 cm^{-1}) were recorded on a Perkin–Elmer 2000 FT-IR or Bruker VERTEX 70V spectrometer using a diamond ATR accessory. Absorption maxima are reported in wavenumbers (cm^{-1}). ^{1}H-NMR and ^{13}C-NMR spectra were recorded on Jeol 400yh (Jeol, Tokyo, Japan) (399.78 MHz for ^{1}H and 100.52 MHz for ^{13}C) or a Bruker Avance II 600 Spectrometer (Bruker, Poznan, Poland) (600.58 MHz for ^{1}H) at 295 K. Chemical shifts (δ) are given in parts per million (ppm) downfield relative to TMS, and coupling constants (*J*) are in hertz (Hz). Residual solvent central signals were recorded as follows: DMSO-d_6, δ_H = 2.500 ppm, δ_C = 39.43 ppm; $CDCl_3$, δ_H = 7.263 ppm, δ_C = 77.00 ppm. Proton coupling patterns were described as singlet (s), doublet (d), triplet (t), and multiplet (m). When measured, DEPT and ATP experiments signals are referred to as (+) or (–). High-resolution mass spectra (HRMS) were recorded on a Waters LCD Premier XE instrument (Manchester United, UK), and the $[M + H]^+$ or $[M + Na]^+$ molecular species are reported, or water elimination species.

The literature procedure was adapted for the preparation of hydrazide-hydrazones **1–3**, **5–11**, **14–25**, **27–30** [1], **32** [2], **31**, **33–35** [3], hydrazides **38–40** [3], aldehydes **53–55**, **58**, **61**, **62** [1,4], **65** [1,5], **66**, **67** [1], **60** [6], **63** [7], **68** [8] and phenol **72** [9]. Purity and homogeneity of known compounds were confirmed by measuring their melting point for **1–3**, **5–11**, **14–21**, **24**, **25**, **27–30** [1], **12** [10], **31**, **33–35** [3], **32** [2], **38** [1], **39** [11,12], **40** [3,12], **60** [13], **63** [7], **68** [8]; boiling point for **65** [1], **68** [8]; FT-IR spectra **3** [1], **22** [14], **25** [1], **38**, **39** [15], **40** [3], **68** [8]; ^{1}H-NMR spectra for **3** [1], **4** [16], **12** [17], **13** [18], **22** [19], **23** [20], **25**, **28** [1], **38**, **39** [15], **40** [3], **60** [6], **65**

[1], **68** [8]; ^{13}C-NMR spectra for **3** [1], **4** [16], **22** [14], **25**, **28** [1], **38**, **39** [15], **40** [3], **65** [1], **68** [8]; and/or HRMS for **3** [1], **4** [16], **25**, **28** [1], and comparing them with literature data. Two new aroyl hydrazide-hydrazones **26**, and **32** were fully characterized. The position of hydrogen and carbon atoms in the NMR data was determined by supporting the standard dept-135 experiment and by 2D map analysis of Heteronuclear Multiple-Quantum Correlation (HMQC), Heteronuclear Single Quantum Coherence (HSQC), Heteronuclear Multiple Bond Correlation (HMBC), Nuclear Overhauser Enhancement Spectroscopy (NOESY) experiments, if measured. The spectra images of FT-IR and NMR for the following compounds are placed in Supplementary Materials as follows: Figure S1-S128.

Table S1. Characteristics of the hydrazide-hydrazones **1–35**.

No.	R^1	R^2	Time [h]	Yield [%]	M.p. [°C]	M.p. lit. [°C]	Lit.
**1**	4-HOC_6H_4	C_6H_5	2	96	242–244	242–244	[1]
**2**	4-HOC_6H_4	$C_6H_5CH_2CH_2$	6	72	243–244	243.5–244.0	[1]
**3**	4-HOC_6H_4	4-$CH_3C_6H_4$	2	79[a]	246–248	246–247	[1]
**4**	4-HOC_6H_4	4-*i*PrC_6H_4	2.5	93	203–205	–[b]	[16]
**5**	4-HOC_6H_4	4-$NaSO_3C_6H_4$	2	97	250.5[c]	251[c]	[1]
**6**	4-HOC_6H_4	2-HOC_6H_4	2	86	265–266	265.0–266.5	[1]
**7**	4-HOC_6H_4	3-HOC_6H_4	2	93[d]	255[c]	254[c]	[1]
**8**	4-HOC_6H_4	4-HOC_6H_4	2	95	263[c]	261[c]	[1]
**9**	4-HOC_6H_4	2-$CH_3OC_6H_4$	4	91	235–236	236–237	[1]
**10**	4-HOC_6H_4	3-$CH_3OC_6H_4$	5	81	208–210	210–212	[1]
**11**	4-HOC_6H_4	4-$CH_3OC_6H_4$	5	85	224–225	223–224	[1]
**12**	4-HOC_6H_4	5-NO_2-2-furyl	20	78	284[c]	282	[10]
**13**	4-HOC_6H_4	2,5-$(HO)_2C_6H_3$	4	87	304[c]	–[b]	[18]
**14**	4-HOC_6H_4	5-Br-2-HOC_6H_3	3	92	277–279	276–278	[1]
**15**	4-HOC_6H_4	2-HO-6-$CH_3OC_6H_3$	2	76	256–257	256.5–257.5	[1]
**16**	4-HOC_6H_4	2,6-$(CH_3O)_2C_6H_3$	6	95	214–215	214.5–216.0	[1]
**17**	4-HOC_6H_4	4-HO-3-$CH_3OC_6H_3$	3	99	218–220	218.5–220.0	[1]
**18**	4-HOC_6H_4	3-*t*Bu-2-HOC_6H_3	6	95	241–243	240–242	[1]
**19**	4-HOC_6H_4	3-C_6H_5-2-HOC_6H_3	6	88[a]	216–218	215–218	[1]
**20**	4-$CH_3OC_6H_4$	3-C_6H_5-2-HOC_6H_3	3	85	240–242	240.0–242.5	[1]
**21**	4-HOC_6H_4	2,4-$(HO)_2C_6H_3$	2	92	293[c]	294[c]	[1]
**22**	C_6H_5	2,4-$(HO)_2C_6H_3$	3.5	89	255–256[c]	249–250	[14]
**23**	4-HOC_6H_4	2,4,5-$(HO)_3C_6H_2$	5	92	253[c]	–[b]	[20]
**24**	4-HOC_6H_4	2-HO-5-$HOCH_2$-3-$CH_3C_6H_2$	3	85	266[c]	266[c]	[1]
**25**	4-HOC_6H_4	2-HO-3-$HOCH_2$-5-$CH_3C_6H_2$	3	80	228–229	229–230	[1]
**26**	4-HOC_6H_4	2-HO-3-$HOCH_2$-5-$CH_3OC_6H_2$	10	97	219–220	–	–
**27**	4-HOC_6H_4	3,5-(*t*Bu)$_2$-2-HOC_6H_2	6	95	274–275	274.5–275.5	[1]
**28**	4-HOC_6H_4	2-HO-3-*i*Pr-6-$CH_3C_6H_2$	6	95	258–260	258.5–260.5	[1]
**29**	4-HOC_6H_4	2-HO-4,6-$(CH_3O)_2C_6H_2$	5	98	231–234	231.5–234.5	[1]
**30**	4-HOC_6H_4	3-*t*Bu-2-HO-5-$CH_3C_6H_2$	2	75	261–262	261–263	[1]
**31**	4-$CH_3OC_6H_4$	3-*t*Bu-2-HO-5-$CH_3C_6H_2$	2	81	243–244	243–244	[3]
**32**	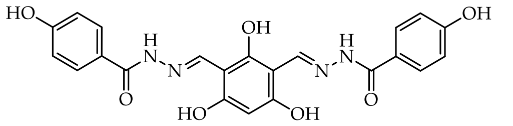		20	95	223–226[c]	223–226[c]	[2]
**33**	C_6H_5	3-*t*Bu-2-HO-5-$CH_3C_6H_2$	2	96	246–248	246–248	[3]
**34**	3-pyridyl	3-*t*Bu-2-HO-5-$CH_3C_6H_2$	1.5	92	231–232	230–231	[3]
**35**	CH_3	3-*t*Bu-2-HO-5-$CH_3C_6H_2$	2.5	84	232–234	231–233	[3]

[a] Crystallize with one molecule of CH_3OH; [b] Not reported; [c] Melts with decomposition; [d] Monohydrate was isolated.

Synthesis

General procedure for the synthesis of hydrazide-hydrazones 1–35 [1,3]**.**

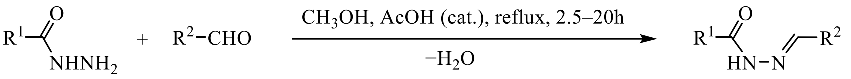

38–40 **41–67** **1–11, 13–35**

To a mixture of aldehyde **41–68** or diacetylacetal **69** (2.0 mmol) and carboxylic acid hydrazide **36–40** (2.0–4.0 mmol) in dry CH_3OH (1.2–140 mL), AcOH (0–200 µL) was added, and then the resulting solution was gently refluxed during stirring with 1.5–20 hours. The reaction progress was monitored by TLC. Then the reaction was finished, after slow cooling to room temperature (RT) and cooled to ca. +4 °C, the reaction mixture was left in a refrigerator (−24 °C) overnight. The crystals formed were collected by filtration with suction, the filter cake was washed with frozen mixture of methanol and water (3:2, *v*/*v*) and dried to obtain pure products **1–35** which were fully characterized by melting point, and NMR, IR, and HRMS spectra. The known compound was identified by comparison of its melting points (Table S2) and FT-IR and/or NMR spectra with literature data. The new products **26** and **32** were fully characterized. For all products, HRMS analyses were measured.

4-Hydroxy-*N*'-[(*E*)-(4-methylphenyl)methylidene]benzohydrazide monomethanolate (3) [1]**.**

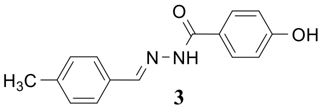

3

The general procedure starting from 4-methylbenzaldehyde (**43**) (240 mg, 2.0 mmol), 4-hydroxybenzohydrazide (**36**) (304 mg, 2.0 mmol), CH_3OH (15 mL), and AcOH (0.10 mL) was employed with a 2 hours reaction time, to obtain the 4-hydroxy-*N*'-[(*E*)-(4-methylphenyl)methylidene]benzohydrazide (**3**) (455 mg, 1.59 mmol) as a colorless powder which crystallize with one molecule of CH_3OH with 79% yield, which melt at 246–248 °C (from CH_3OH) (m.p. 246–247 °C [1]).

The FT-IR, ^{1}H-NMR, ^{13}C-NMR spectra and HRMS analyze are consistent with literature values [1].

4-Hydroxy-*N*'-[(*E*)-(4-*iso*-propylphenyl)methylidene]benzohydrazide (4) [16]**.**

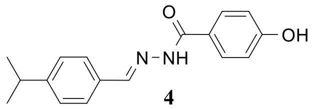

4

The general procedure starting from 4-isopropylbenzaldehyde (**44**) (296 mg, 2.0 mmol), 4-hydroxybenzohydrazide (**36**) (304 mg, 2.0 mmol), CH_3OH (4.5 mL), and AcOH (0.10 mL) was employed with a 2.5 hours reaction time, add water (0.30 mL) and left to crystallization in open vessel at RT to obtain the 4-hydroxy-*N*'-[(*E*)-(4-*iso*-propylphenyl)methylidene]benzohydrazide (**4**) (526 mg, 1.86 mmol) as a colorless powder with 93% yield, which melt at 203–205 °C (from CH_3OH); selected FT-IR (ATR): ν_{max} 3552 (N-H, O-H), 3250 (O-H, N-H), 3151 (br, N-H), 3034 (C_{Ar}-H), 2957 (CH), 1641 (C=O), 1607 (C=N), 1541 (N-H), 1507 (C_{Ar}-H), 1370, 1271 (C_{Ar}-O), 1237, 1174, 1052, 972 (N-N), 828, 763, 622, 549 cm^{-1}; ^{1}H-NMR (400 MHz, DMSO-d_6) [16]: δ 11.59 (s, 1H, NH), 10.13 (s, 1H, OH), 8.40 (s, 1H, CH=N), 7.81 (d, ^3J = 8.6 Hz, 2H, H-2,6), 7.63 (d, ^3J = 8.1 Hz, 2H, ArH-2,6), 7.32 (d, ^3J = 8.1 Hz, 2H, ArH-3,5), 6.86 (d, ^3J = 8.6 Hz, 2H, H-3,5), 2.91 (m, ^3J = 6.9 Hz, 1H, CH), 1.21 (d, ^3J = 6.9 Hz, 6H, 2 × CH_3) ppm; ^{13}C-NMR (101 MHz, DMSO-d_6) [16]: δ 162.66 (C=O), 160.59 (C-4), 150.40 (ArC-4), 146.85 (CH=N), 132.16 (ArC-1), 129.59 (C-2,6), 127.00 (ArC-2,6), 126.73 (ArC-3,5), 123.87 (C-1), 114.96 (C-3,5), 33.32 (CH), 23.63 (2 × CH_3) ppm; HRMS (TOF, MS, ESI): *m*/*z* for $C_{17}H_{18}N_2O_2 + H^+$ calculated: 283.1441; found: 283.1436.

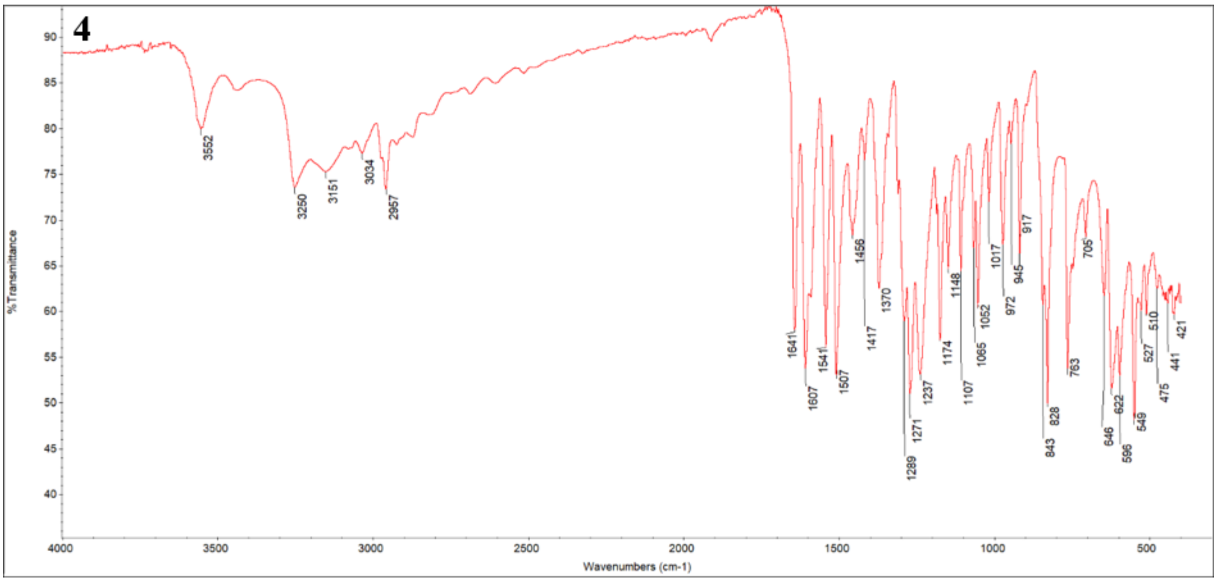

Figure S1. FT-IR (ATR) (4000–400 cm^{-1}) spectrum of 4-hydroxy-*N*'-[(*E*)-(4-*iso*-propylphenyl)methylidene]benzohydrazide (**4**).

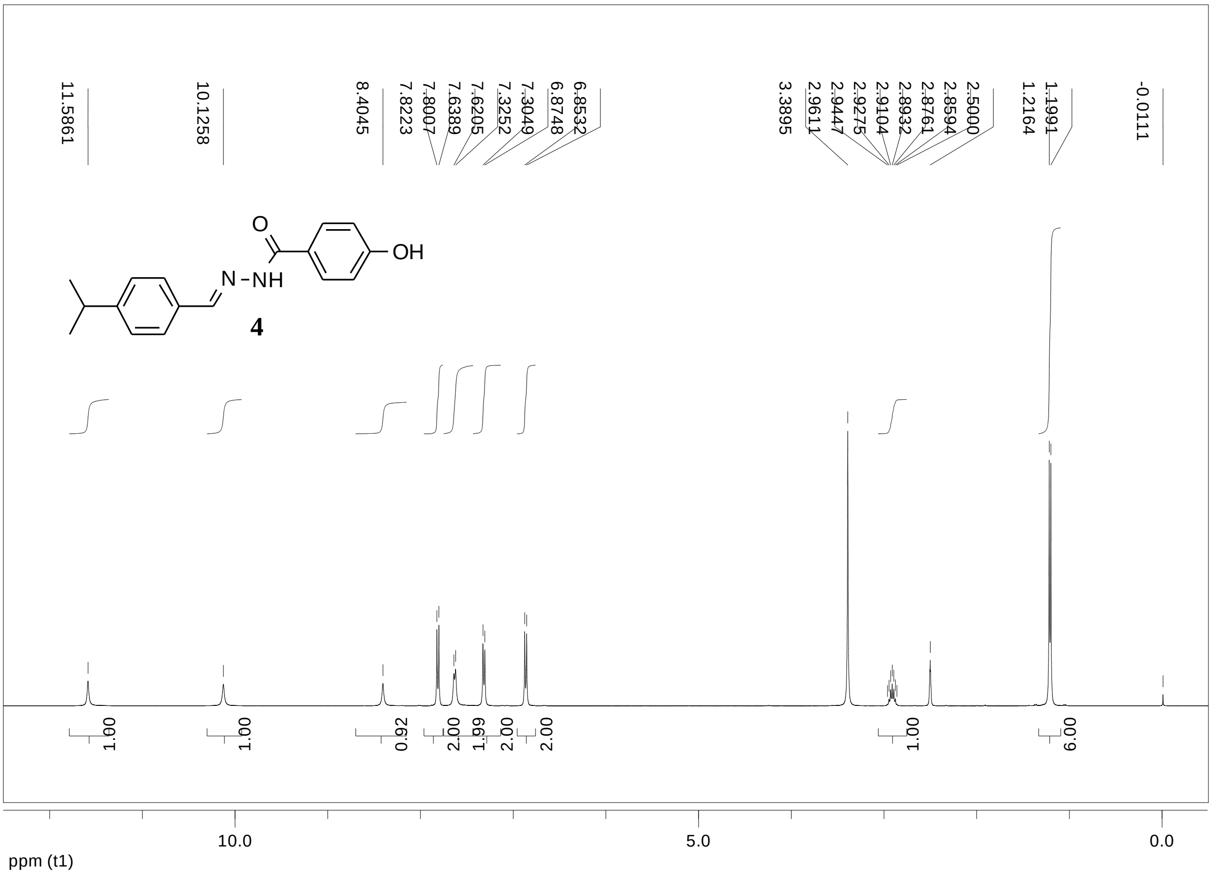

Figure S2. ^{1}H-NMR (400MHz, DMSO-d_6) spectrum of compound **4**.

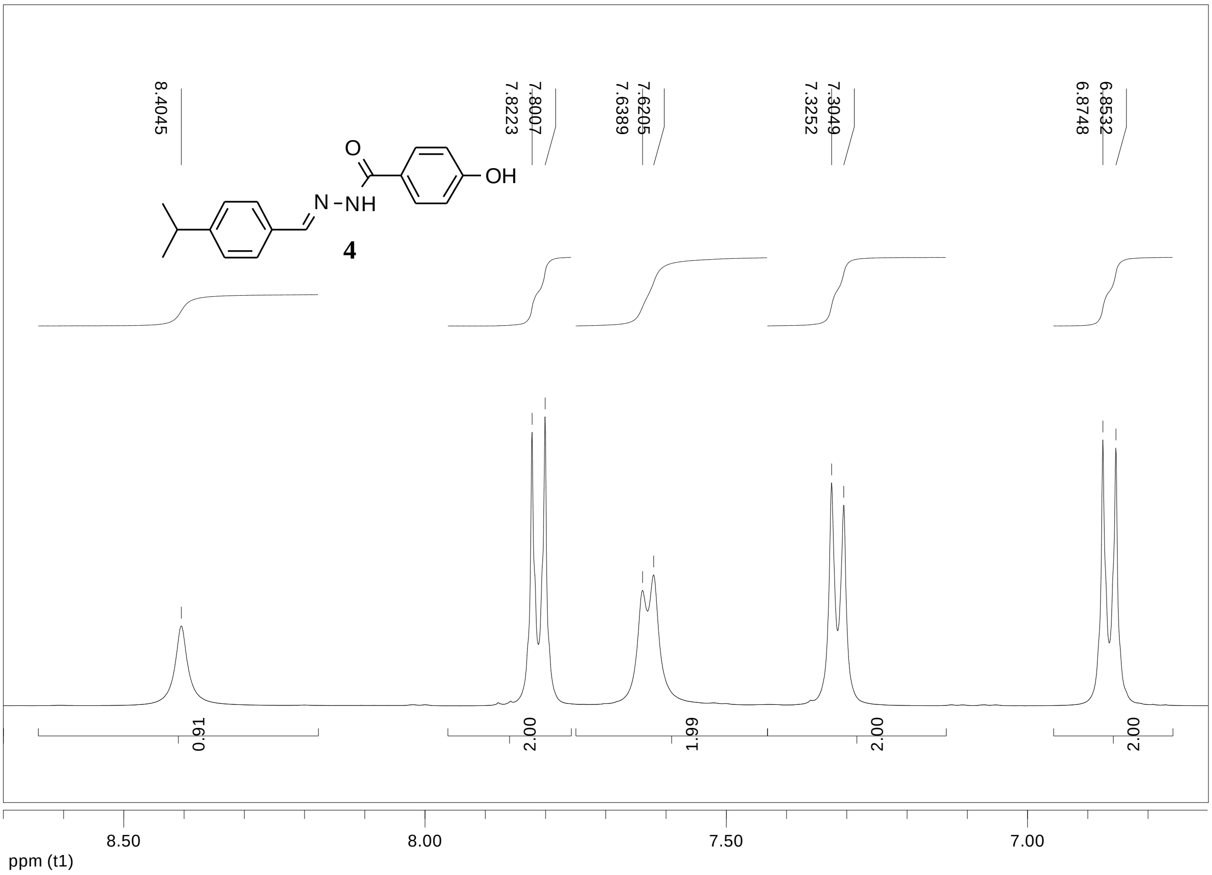

Figure S3. Expansion ^{1}H-NMR (400MHz, DMSO-d_6) spectrum of compound **4**.

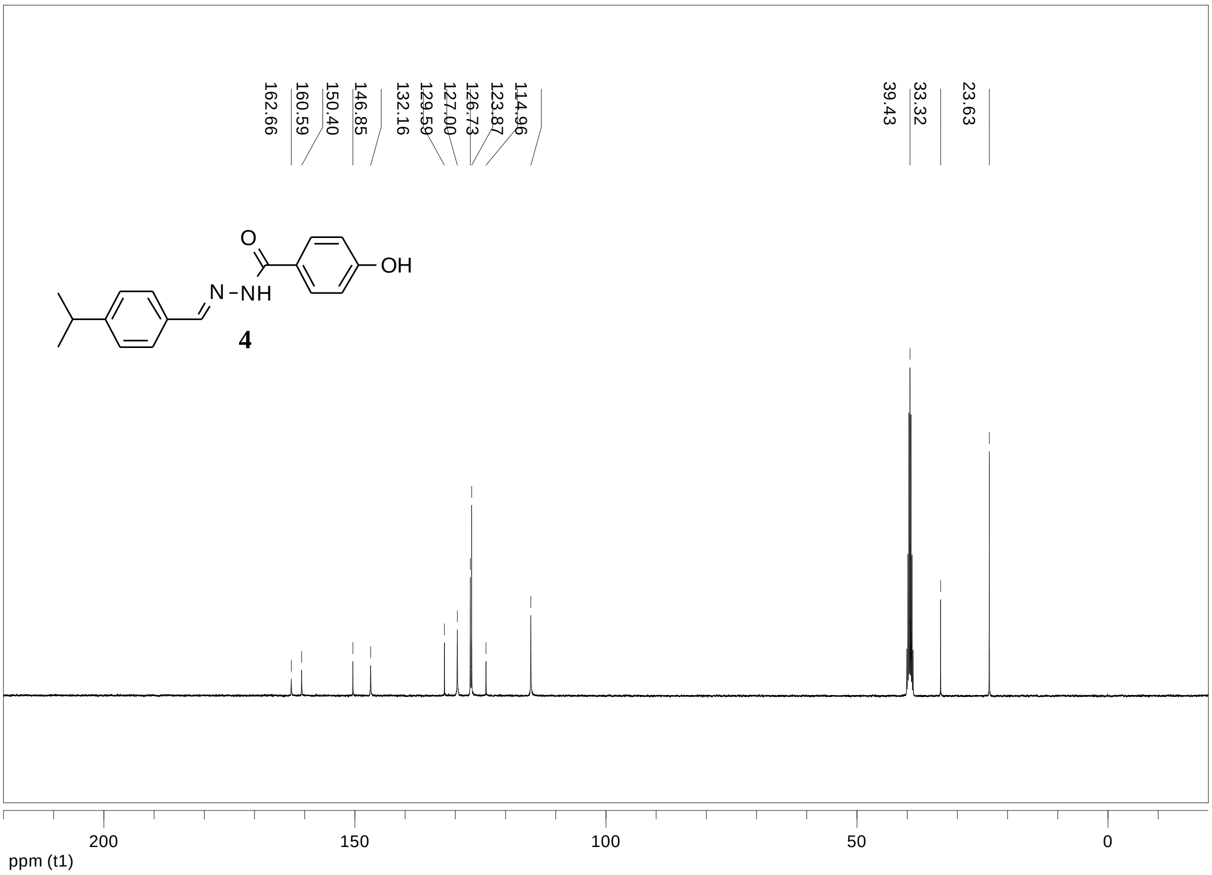

Figure S4. ^{13}C-NMR (101 MHz, DMSO-d_6) spectrum of compound **4**.

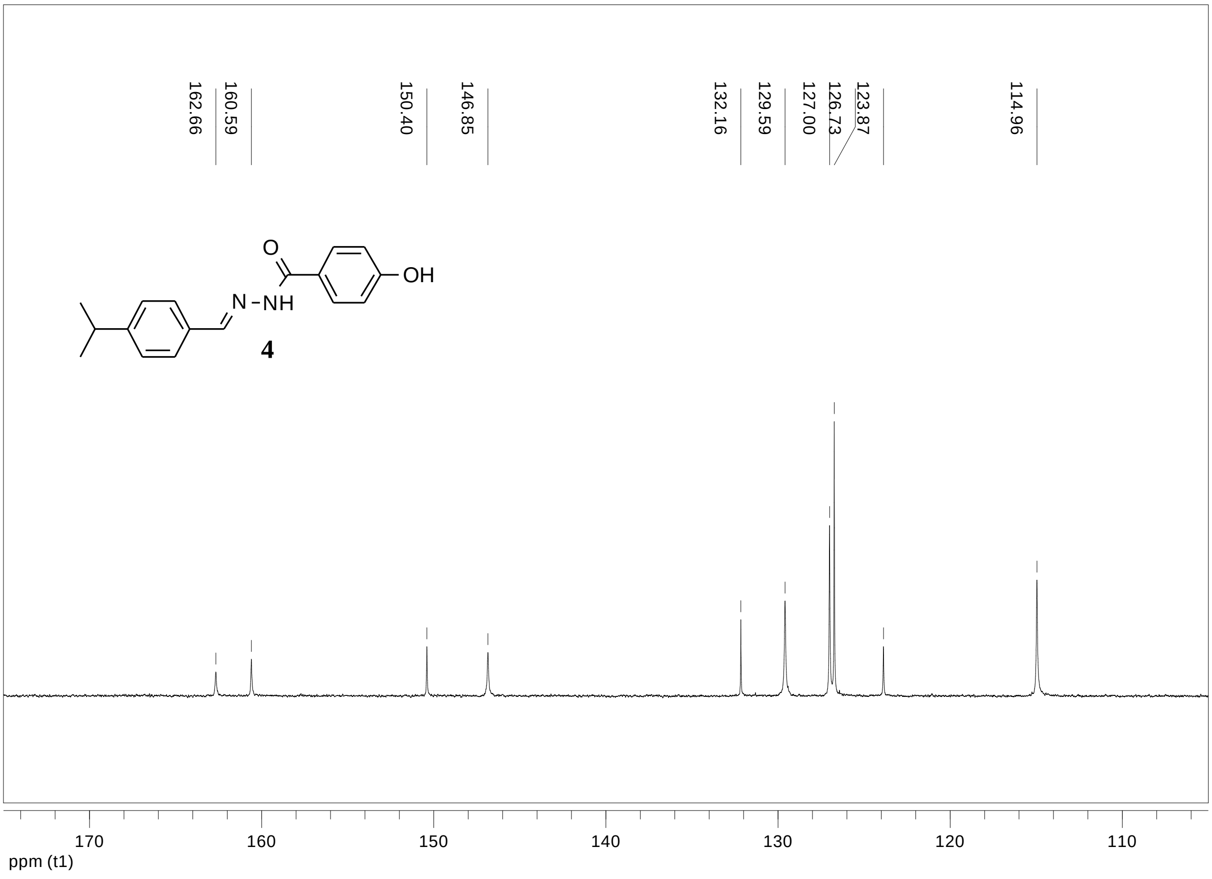

Figure S5. Expansion ^{13}C-NMR (101 MHz, DMSO-d_6) spectrum of compound **4**.

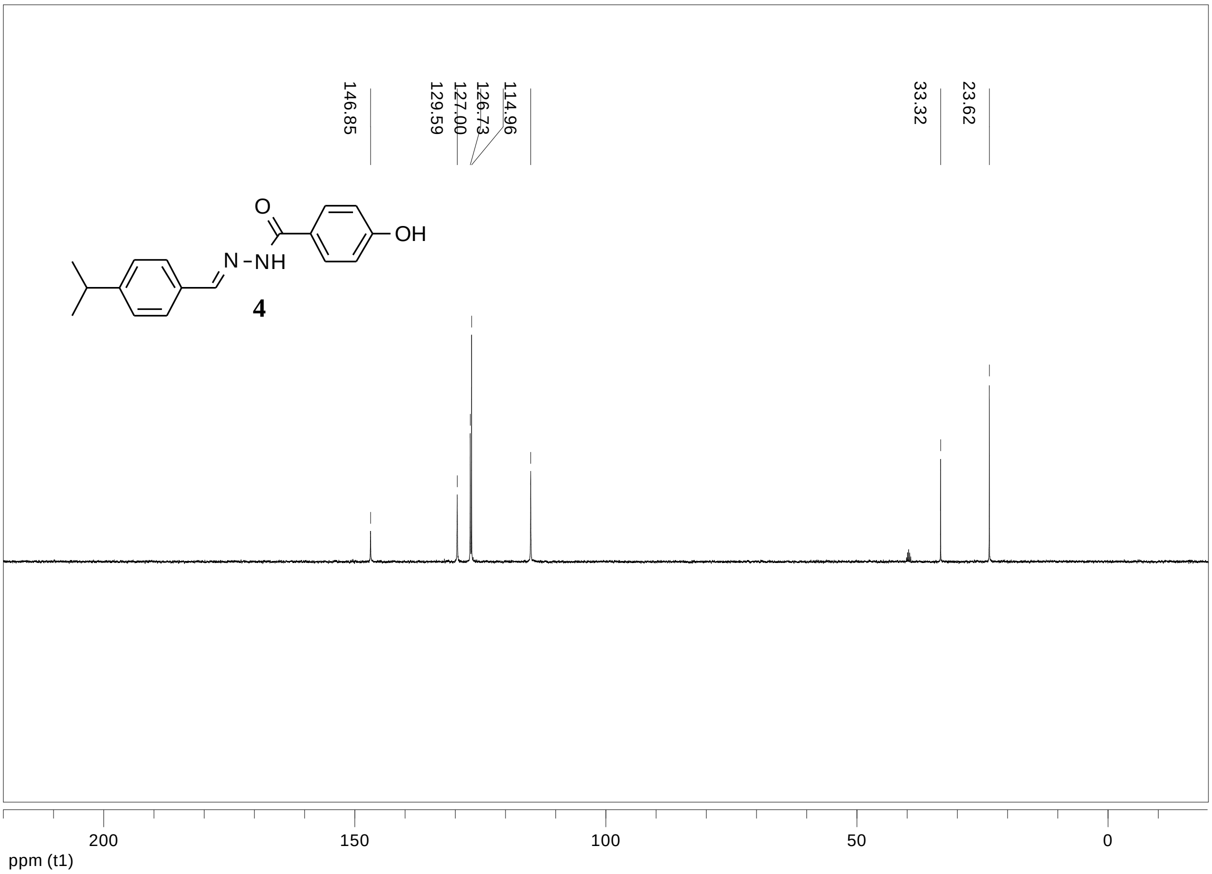

Figure S6. ^{13}C-NMR (101 MHz, DMSO-d_6) dept-135 experiment of compound **4**.

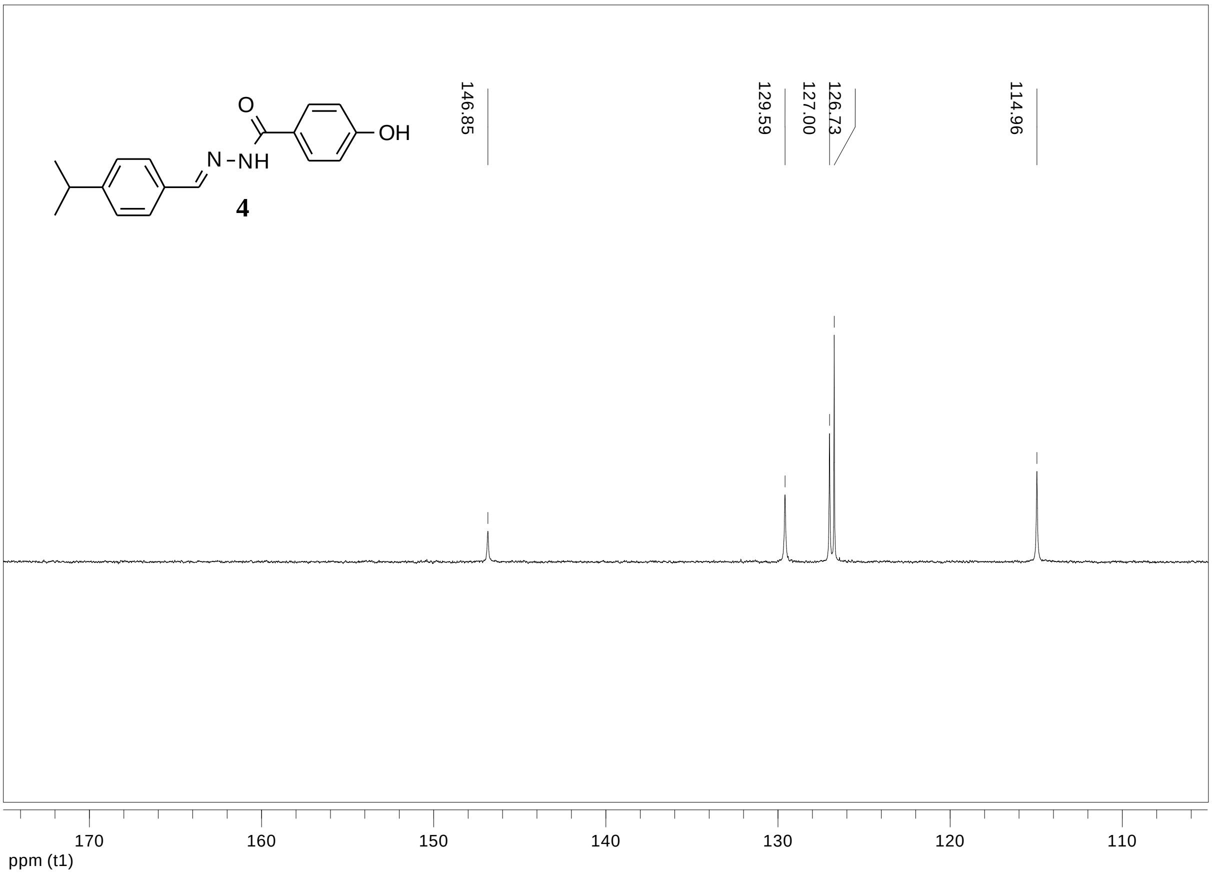

Figure S7. Expansion of ^{13}C-NMR (101 MHz, DMSO-d_6) dept-135 experiment of compound **4**.

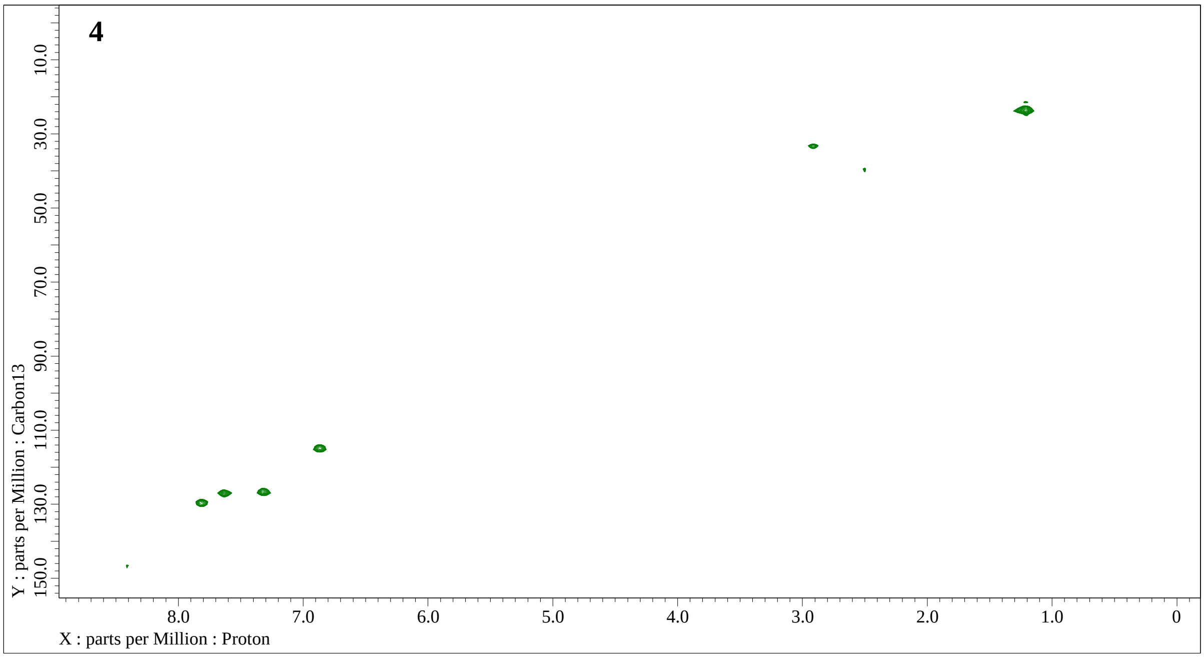

Figure S8. 2D-NMR (400 MHz, DMSO-d_6) HSQC experiment of 4-hydroxy-*N*'-[(*E*)-(4-*iso*-propylphenyl)methylidene]benzohydrazide (**4**).

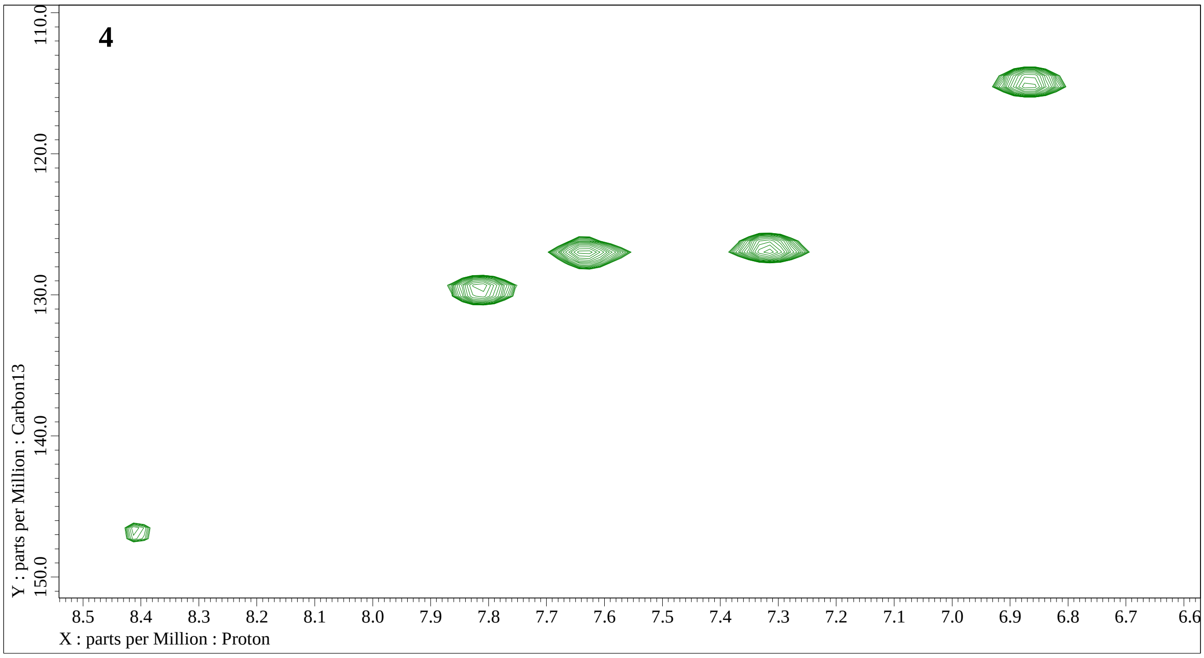

Figure S9. Expansion 2D-NMR (400 MHz, DMSO-d_6) HSQC experiment of 4-hydroxy-*N*'-[(*E*)-(4-*iso*-propylphenyl)methylidene]benzohydrazide (**4**).

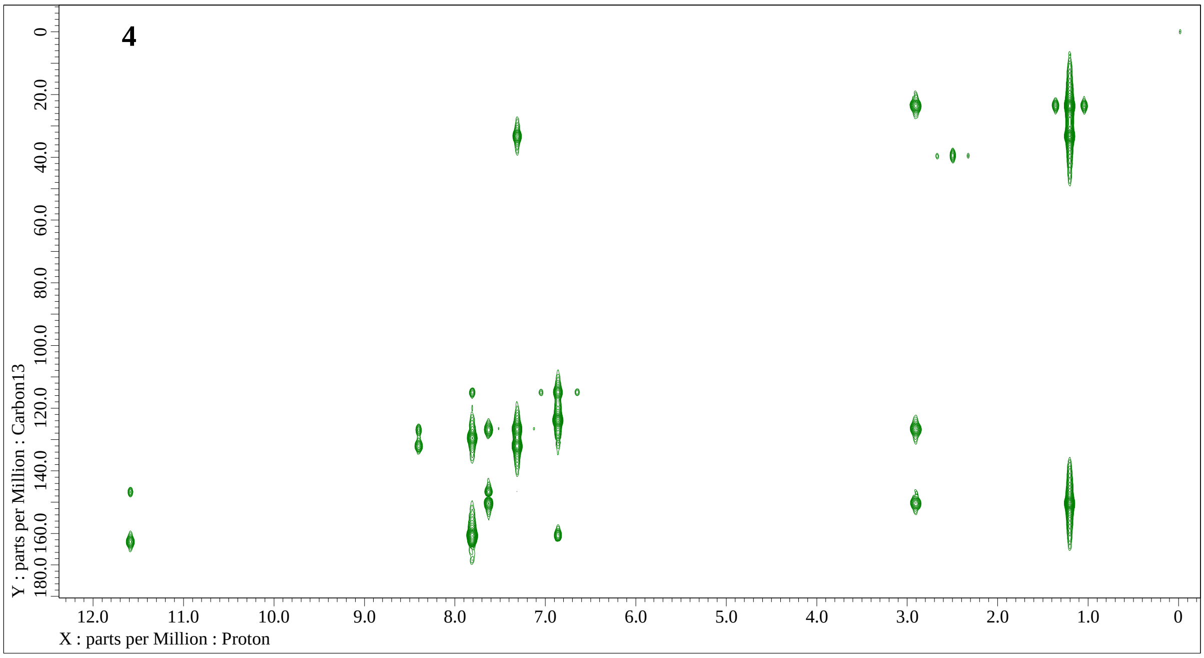

Figure S10. 2D-NMR (400 MHz, DMSO-d_6) HMBC experiment of 4-hydroxy-*N*'-[(*E*)-(4-*iso*-propylphenyl)methylidene]benzohydrazide (**4**).

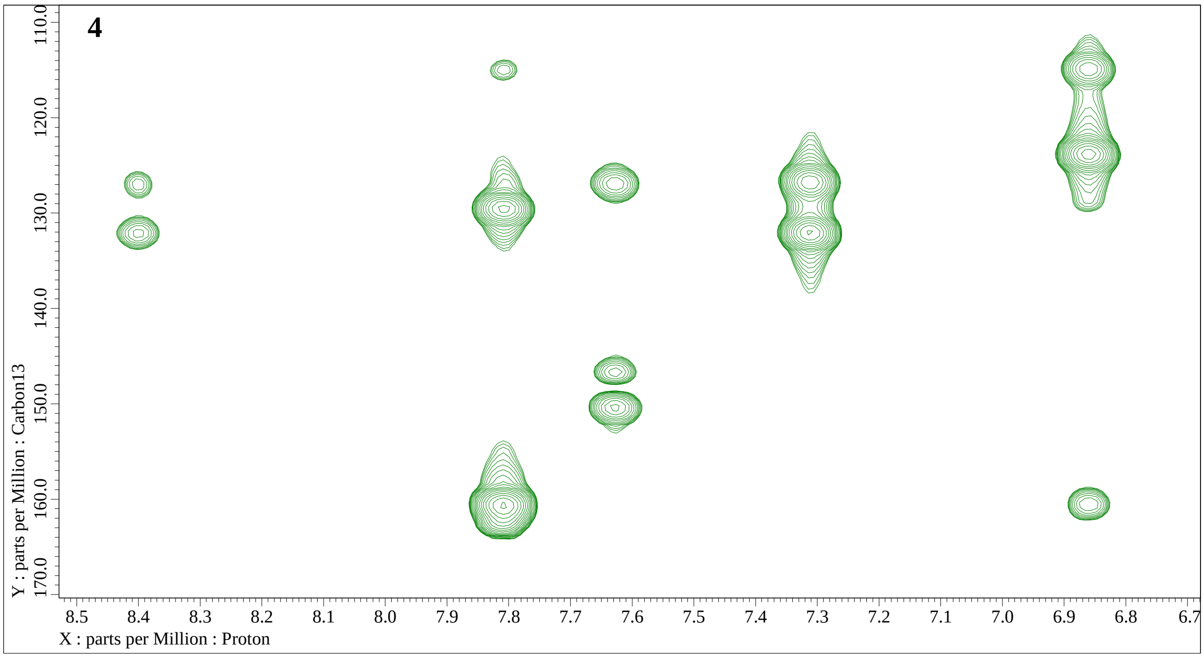

Figure S11. Expansion of 2D-NMR (400 MHz, DMSO-d_6) HMBC experiment of 4-hydroxy-*N*'-[(*E*)-(4-*iso*-propylphenyl)methylidene]benzohydrazide (**4**).

4-Hydroxy-*N*'-[(*E*)-(5-nitrofuran-2-yl)methylidene]benzohydrazide (12) [17].

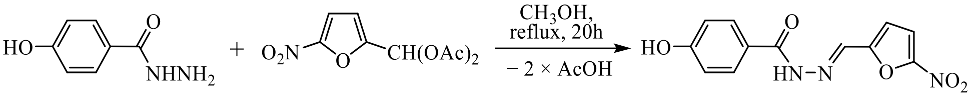

36 **69** Nifuroxide (**12**)

The general procedure starting from 5-nitro-2-furfural diacetyl acetal (**69**) (1.22g, 5.0 mmol), 4-hydroxybenzohydrazide (**36**) (837 mg, 5.5 mmol), CH_3OH (10 mL), at 55 °C was employed with a 20 hours reaction time to obtain the 4-hydroxy-*N*'-[(*E*)-(5-nitrofuran-2-yl)methylidene]benzohydrazide – Nifuroxazide (**12**). Yellow powder; 1.07g, 3.88 mmol, 78% yield. The analytical pure sample was obtained by recrystallization from 95% ethanol (5 mg/mL), which melts at 284 °C with decomposition (from ethanol) (m.p. 282 °C [10]); selected FT-IR (ATR): ν_{max} 3364 (O-H), 3234 (br, N-H), 3129, 1669 (C=O), 1606 (C_{Ar}-H), 1583, 1552 (C=N), 1506 (NO_2), 1360 (NO_2), 1255 (br, C_{Ar}-O), 1187, 1117, 1025, 963 (N-N), 933, 851, 796, 717, 623, 545 (br) cm^{-1}; ^{1}H-NMR (400 MHz, DMSO-d_6): δ 12.04 (s, 1H, NH), 10.23 (s, 1H, OH), 8.37 (s, 1H, CH=N), 7.81 (d, ^3J = 8.7 Hz, 2H, H-2,6), 7.78 (d, ^3J = 3.9 Hz, 1H, FurH-4), 7.23 (d, ^3J = 3.9 Hz, 1H, FurH-3), 6.88 (d, ^3J = 8.7 Hz, 2H, H-3,5) ppm; ^{13}C-NMR (101 MHz, DMSO-d_6): δ 162.97 (C=O), 161.06 (C-4), 152.00 (ArC-2), 151.77 (ArC-5), 134.42 (CH=N), 129.94 (C-2,6), 123.11 (C-1), 115.10 (C-3,5), 114.82 (FurC-3), 114.69 (FurC-4) ppm; HRMS (TOF, MS, ESI): *m/z* for $C_{12}H_9N_3O_5 + H^+$ calculated: 276.0615; found: 276.0615.

^{1}H-NMR spectrum is consistent with the literature value [17].

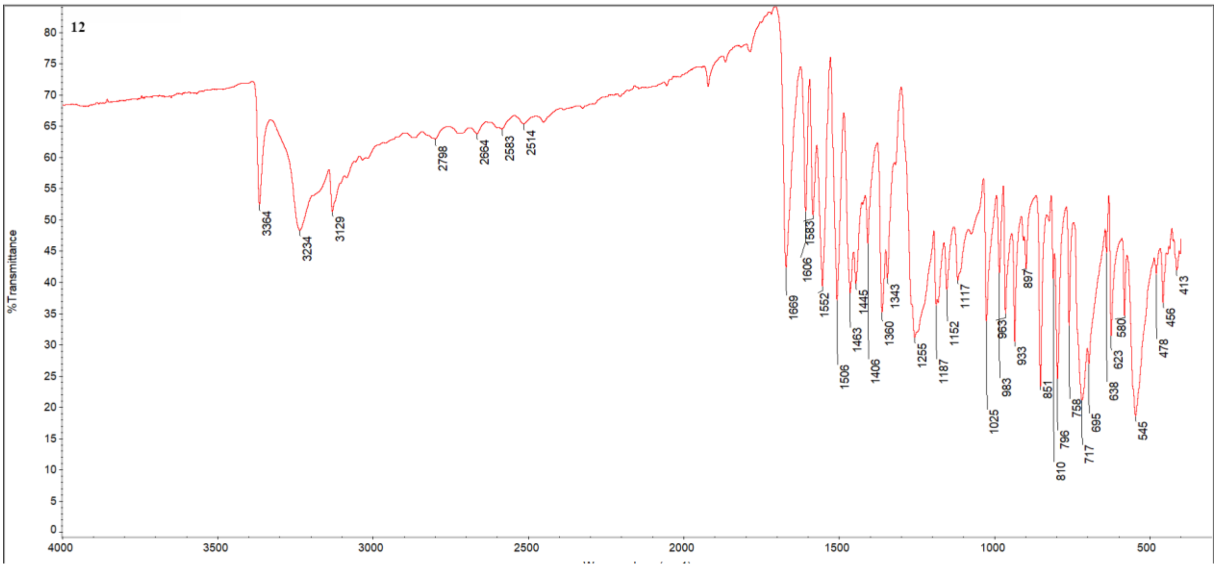

Figure S12. FT-IR (ATR) (4000–400 cm^{-1}) spectrum of Nifuroxazide – 4-hydroxy-*N*'-[(*E*)-(5-nitrofuran-2-yl)methylidene]benzohydrazide (**12**).

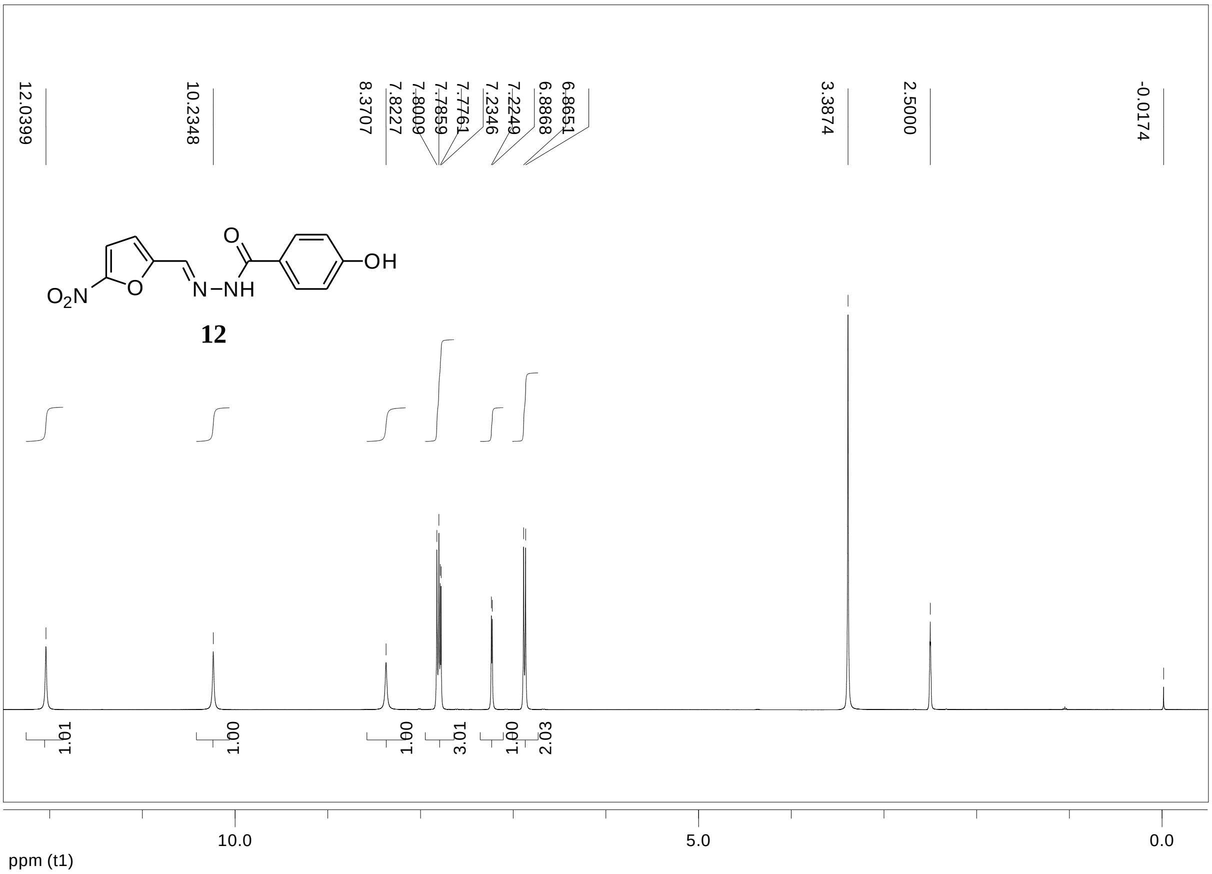

Figure S13. ^{1}H-NMR (400 MHz, DMSO-d_6) spectrum of Nifuroxazide (**12**).

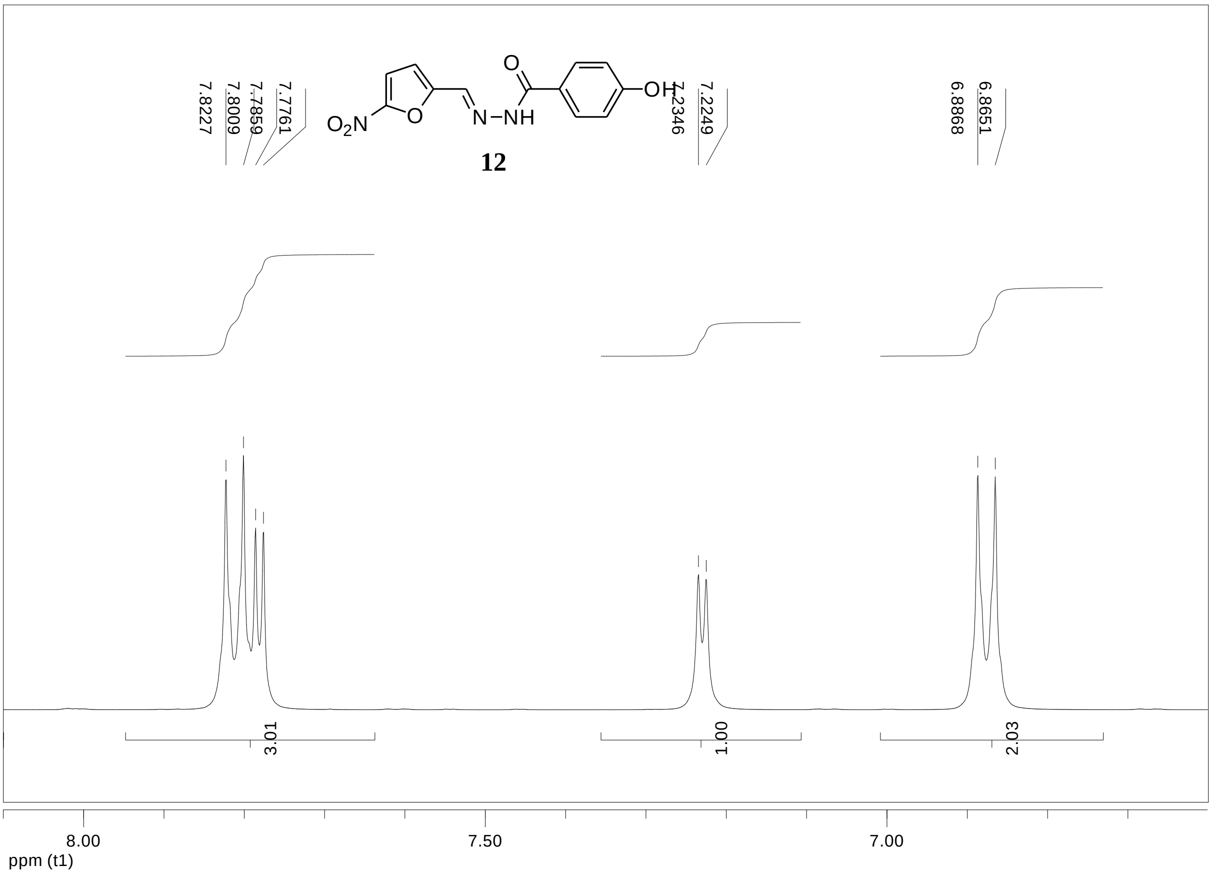

Figure S14. Expansion of ^{1}H-NMR (400 MHz, DMSO-d_6) spectrum of Nifuroxazide (**12**).

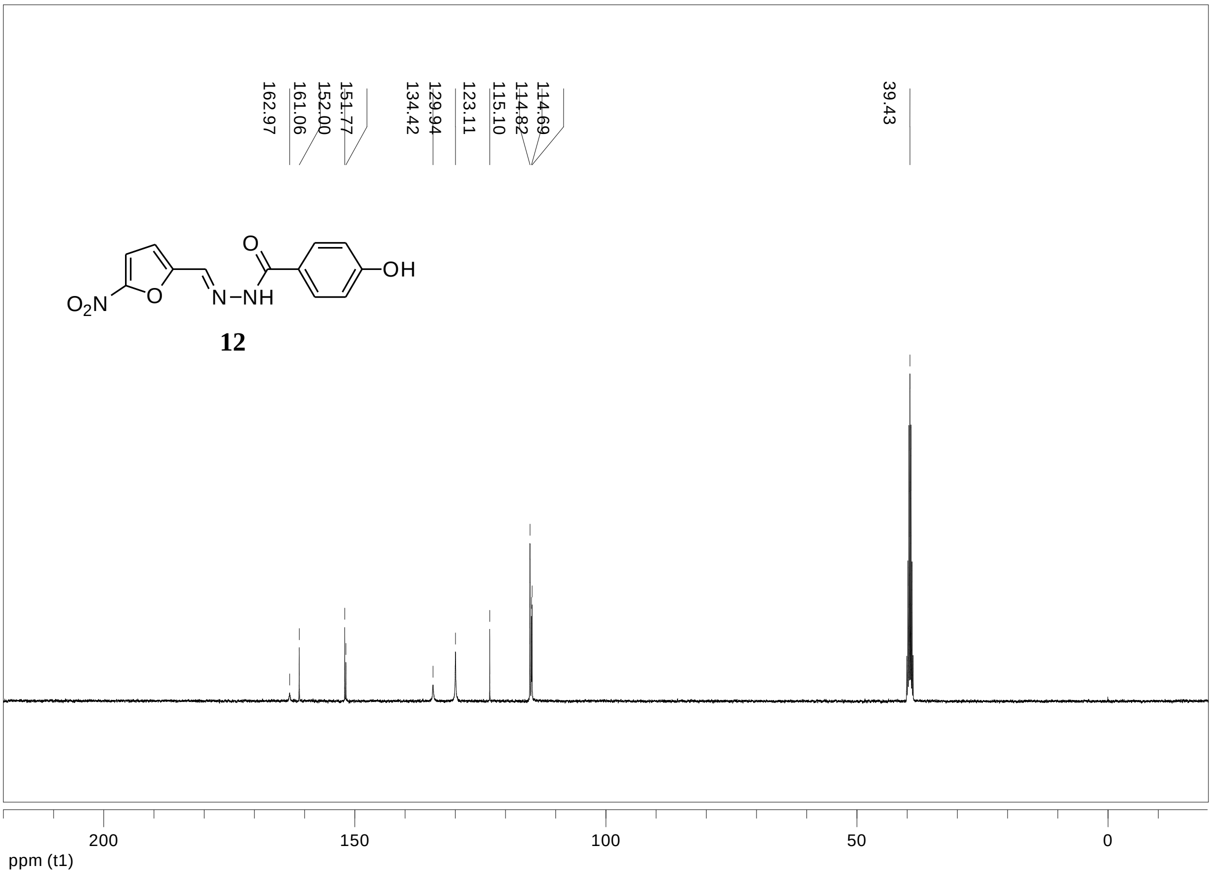

Figure S15. ^{13}C-NMR (101 MHz, DMSO-d_6) spectrum of Nifuroxazide (**12**).

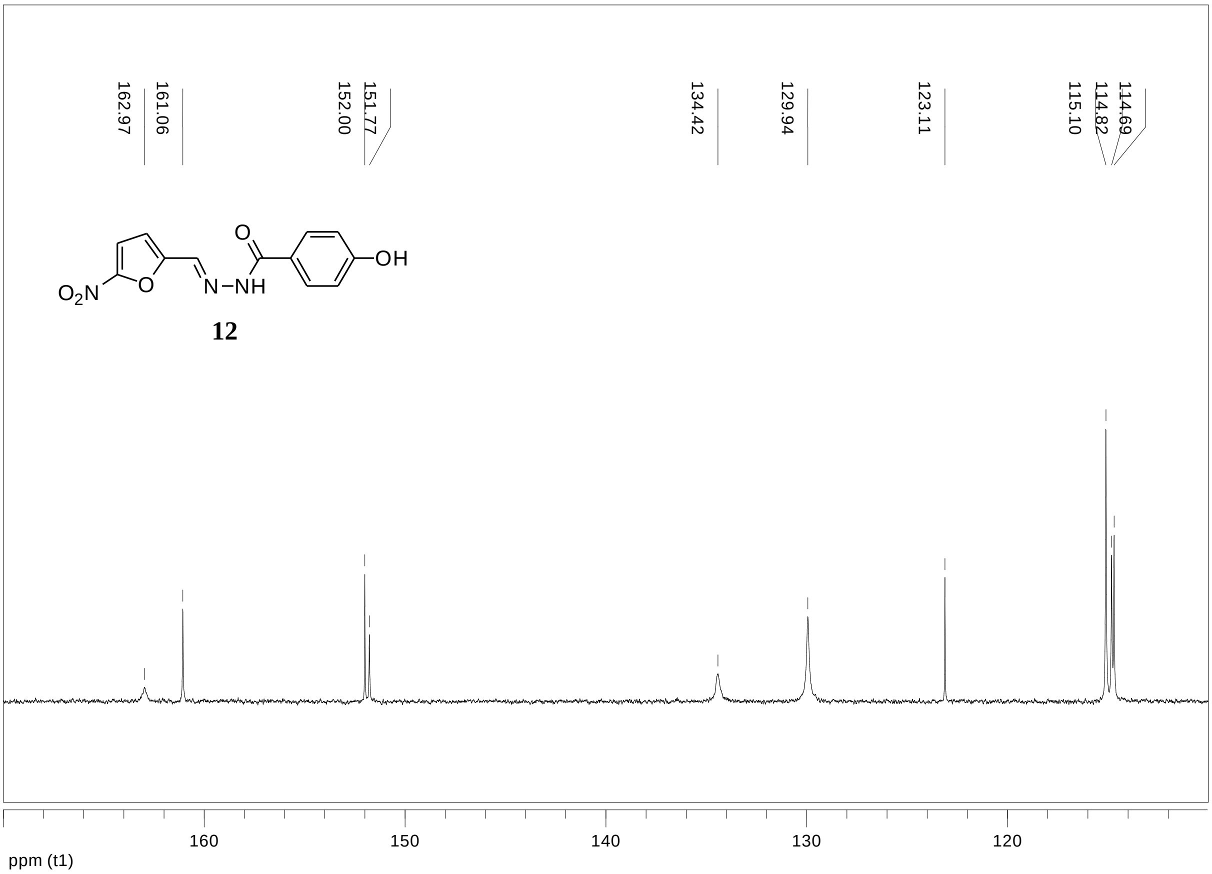

Figure S16. Expansion of ^{13}C-NMR (101 MHz, DMSO-d_6) spectrum of Nifuroxazide (**12**).

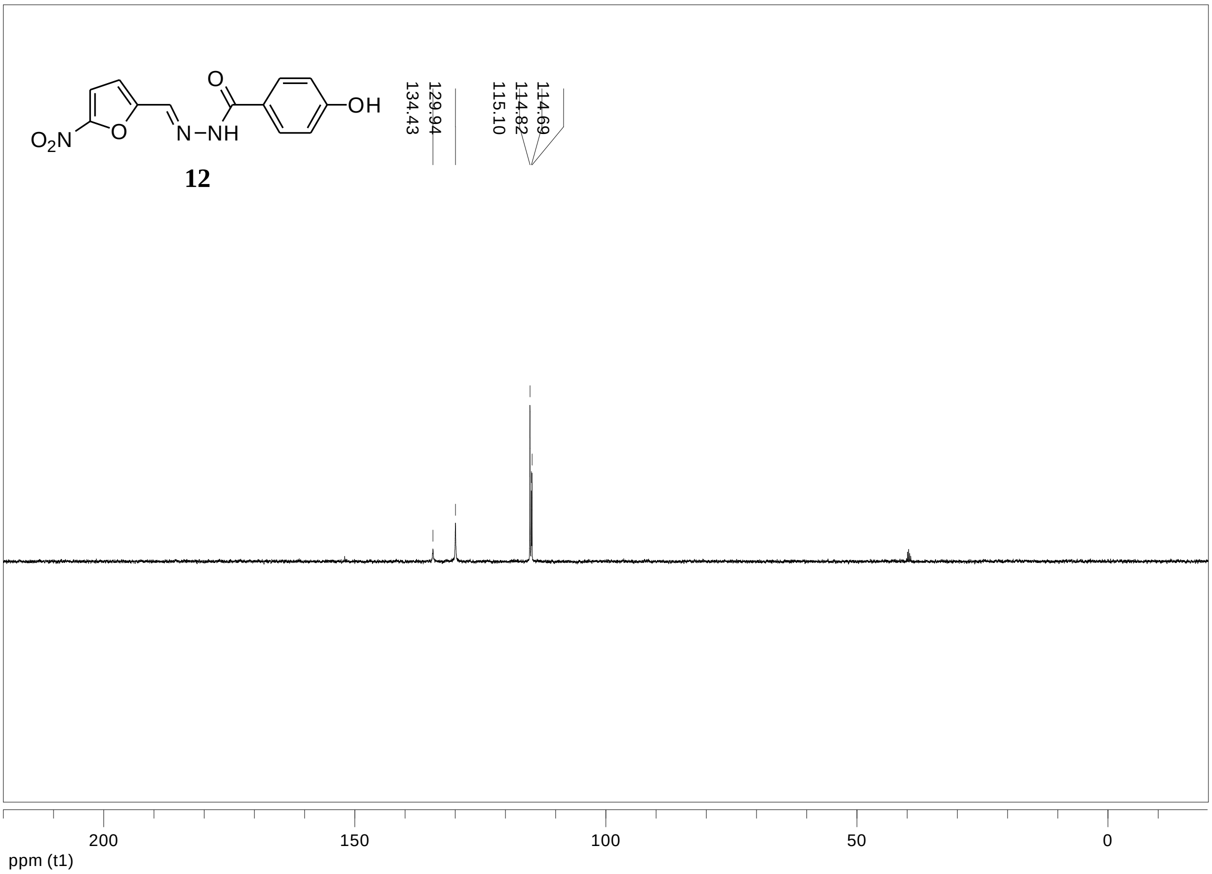

Figure S17. ^{13}C-NMR (101 MHz, DMSO-d_6) dept-135 experiment of Nifuroxazide (**12**).

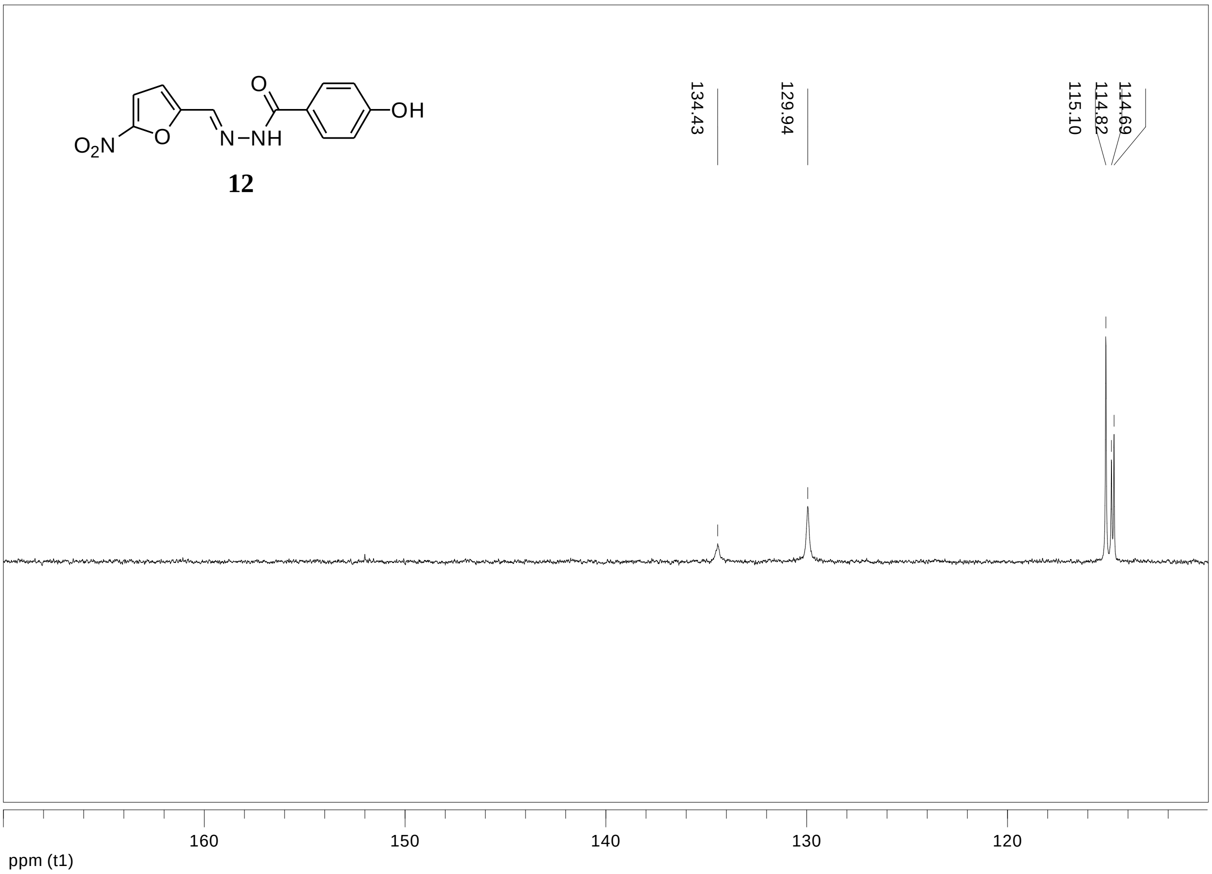

Figure S18. Expansion of ^{13}C-NMR (101 MHz, DMSO-d_6) dept-135 experiment of Nifuroxazide (**12**).

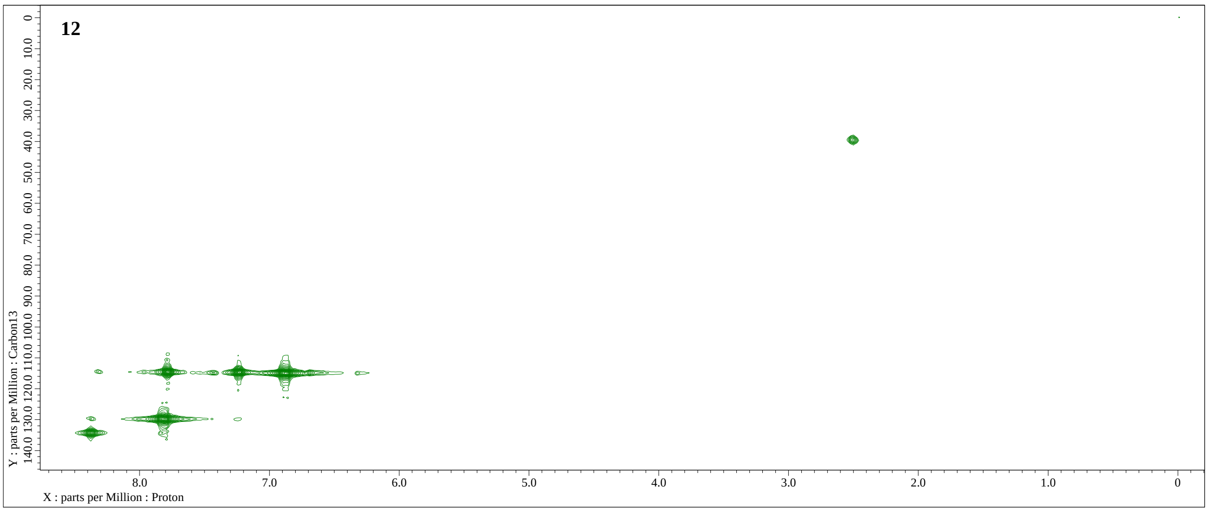

Figure S19. 2D-NMR (400 MHz, DMSO-d_6) HMQC experiment of Nifuroxazide – 4-hydroxy-*N*'-[(*E*)-(5-nitrofuran-2-yl)methylidene]benzohydrazide (**12**).

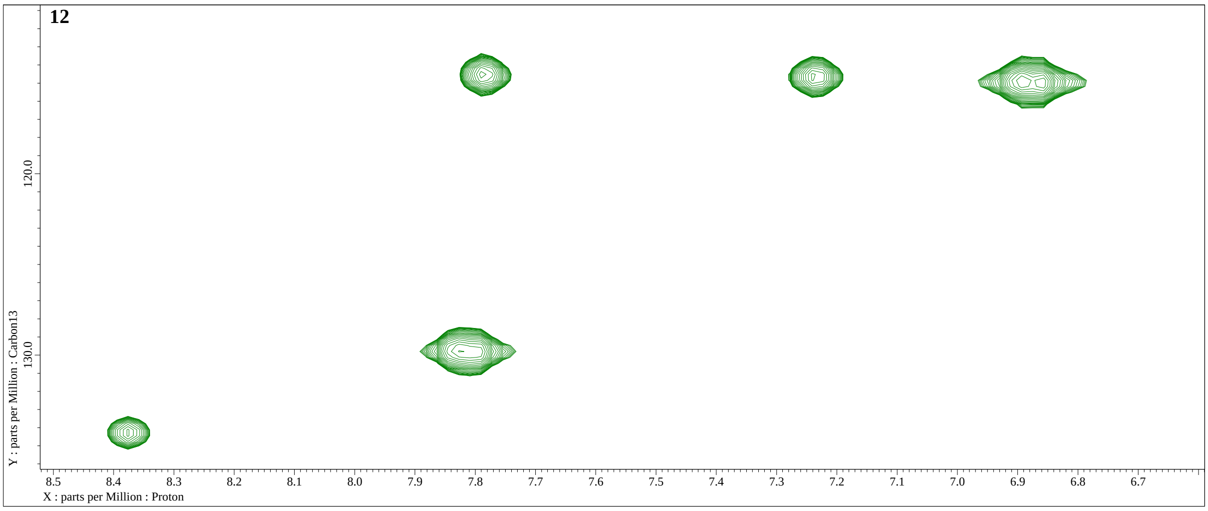

Figure S20. Expansion of 2D-NMR (400 MHz, DMSO-d_6) HMQC experiment of Nifuroxazide – 4-hydroxy-*N*'-[(*E*)-(5-nitrofuran-2-yl)methylidene]benzohydrazide (**12**).

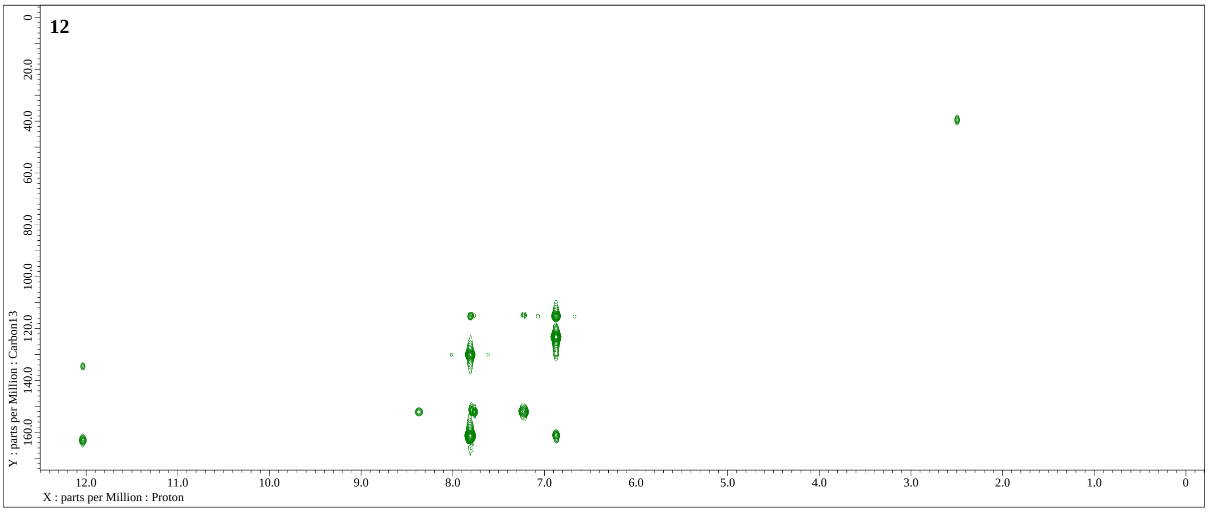

Figure S21. 2D-NMR (400 MHz, DMSO-d_6) HMBC experiment of Nifuroxazide – 4-hydroxy-*N*'-[(*E*)-(5-nitrofuran-2-yl)methylidene]benzohydrazide (**12**).

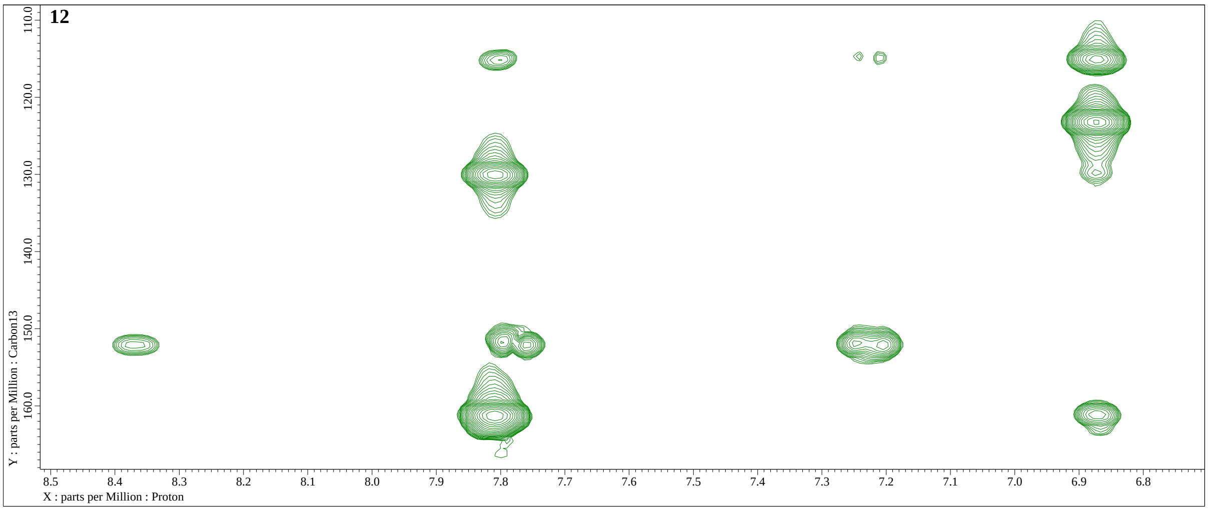

Figure S22. Expansion of 2D-NMR (400 MHz, DMSO-d_6) HMBC experiment of Nifuroxazide – 4-hydroxy-*N*'-[(*E*)-(5-nitrofuran-2-yl)methylidene]benzohydrazide (**12**).

4-Hydroxy-*N*'-[(*E*)-(2,5-dihydroxyphenyl)methylidene]benzohydrazide (13).

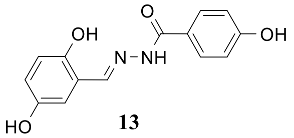

13

The general procedure starting from 2,5-dihydroxybenzaldehyde (**52**) (276 mg, 2.0 mmol), 4-hydroxybenzohydrazide (**36**) (304 mg, 2.0 mmol), CH_3OH (70 mL), and AcOH (0.20 mL) was employed with a 4 hours reaction time, decolorized with charcoal and concentrated before crystallization to obtain the 4-hydroxy-*N*'-[(*E*)-(2,5-dihydroxyphenyl)methylidene]benzohydrazide (**13**). Yellow powder; 472 mg, 1.73 mmol, 87% yield; m.p. 304 °C with decomposition; selected FT-IR (ATR): ν_{max} 3266 (N-H, O-H), 3133 (br, O-H, N-H), 3068 (C_{Ar}-H), 3041 (C_{Ar}-H), 1635 (C=C), 1611 (C=O), 1583 (N-H), 1544 (CH=N), 1510, 1500, 1438, 1379, 1292 (C_{Ar}-O), 1285, 1260, 1228 (br, C_{Ar}-O), 1174 (C_{Ar}-O), 1149, 1110, 951, 845, 771, 762, 707, 673, 579, 478 cm^{-1}; ^{1}H-NMR (601 MHz, DMSO-d_6): δ 11.81 (s, 1H, NH), 10.52 (s, 1H, OH), 10.17 (s, 1H, OH), 8.98 (s, 1H, OH), 8.52 (s, 1H, CH=N), 7.82 (d, ^3J = 8.6 Hz, 2H, H-2,6), 6.93 (d, ^4J = 2.7 Hz, 1H, ArH-6), 6.87 (d, ^3J = 8.6 Hz, 2H, H-3,5), 6.75 (d, ^3J = 8.7 Hz, 1H, ArH-3), 6.72 (dd, ^3J = 8.7 Hz, ^4J = 2.7 Hz, 1H, ArH-4) ppm; ^{13}C-NMR (101 MHz, DMSO-d_6): δ 162.38 (C=O), 160.81 (C-4), 150.14 (ArC-2), 149.79 (ArC-5), 146.97 (CH=N), 129.67 (C-2,6), 123.31 (C-1), 118.98 (ArC-1), 118.62 (ArC-3), 116.99 (ArC-4), 115.07 (C-3,5), 113.99 (ArC-5) ppm; HRMS (TOF, MS, ESI): *m/z* for $C_{14}H_{12}N_2O_4 + H^+$ calculated: 273.0870; found: 273.0867.

^{1}H-NMR (400 MHz, DMSO-d_6) spectra are partially consistent with the literature [18]. The discrepancies are in the number and positions of protons observed in the spectrum. Particularly, we observed a singlet at 8.98 ppm of the OH group and only three signals at the 6.80–7.00 ppm region.

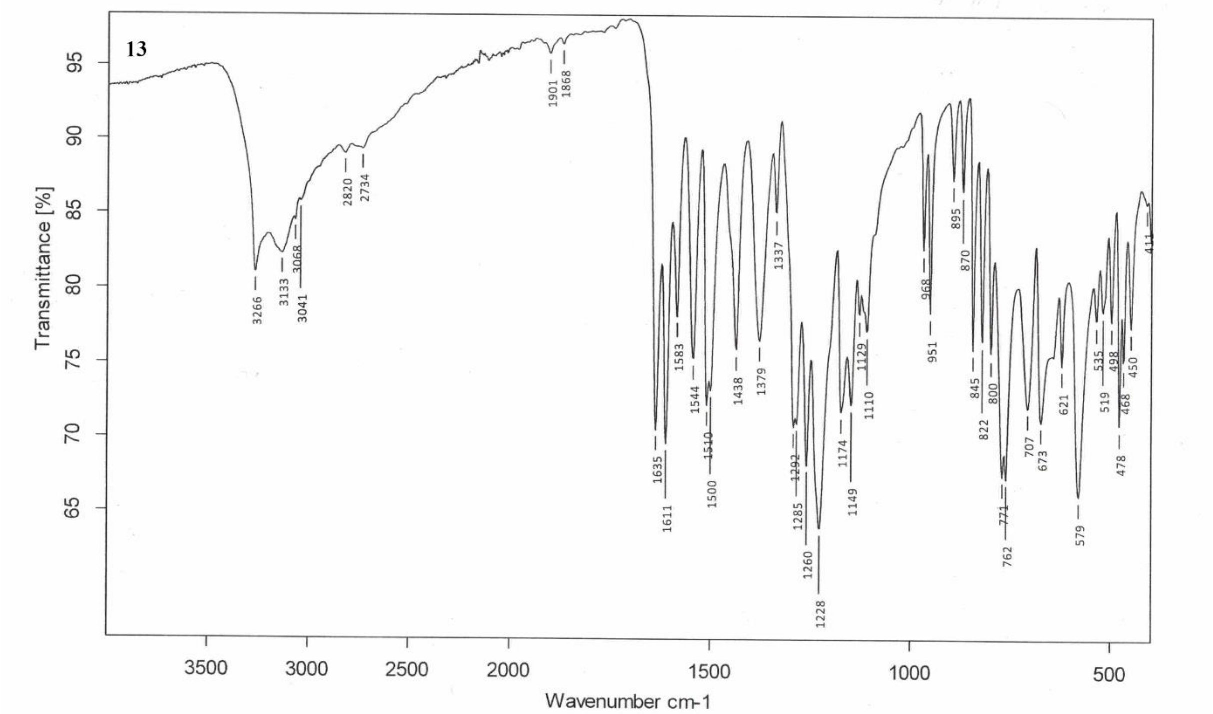

Figure S23. FT-IR (ATR) (4000–400 cm^{-1}) spectrum of gentisaldehyde derivative – 4-hydroxy-*N*'-[(*E*)-(2,5-dihydroxyphenyl)methylidene]benzohydrazide (**13**).

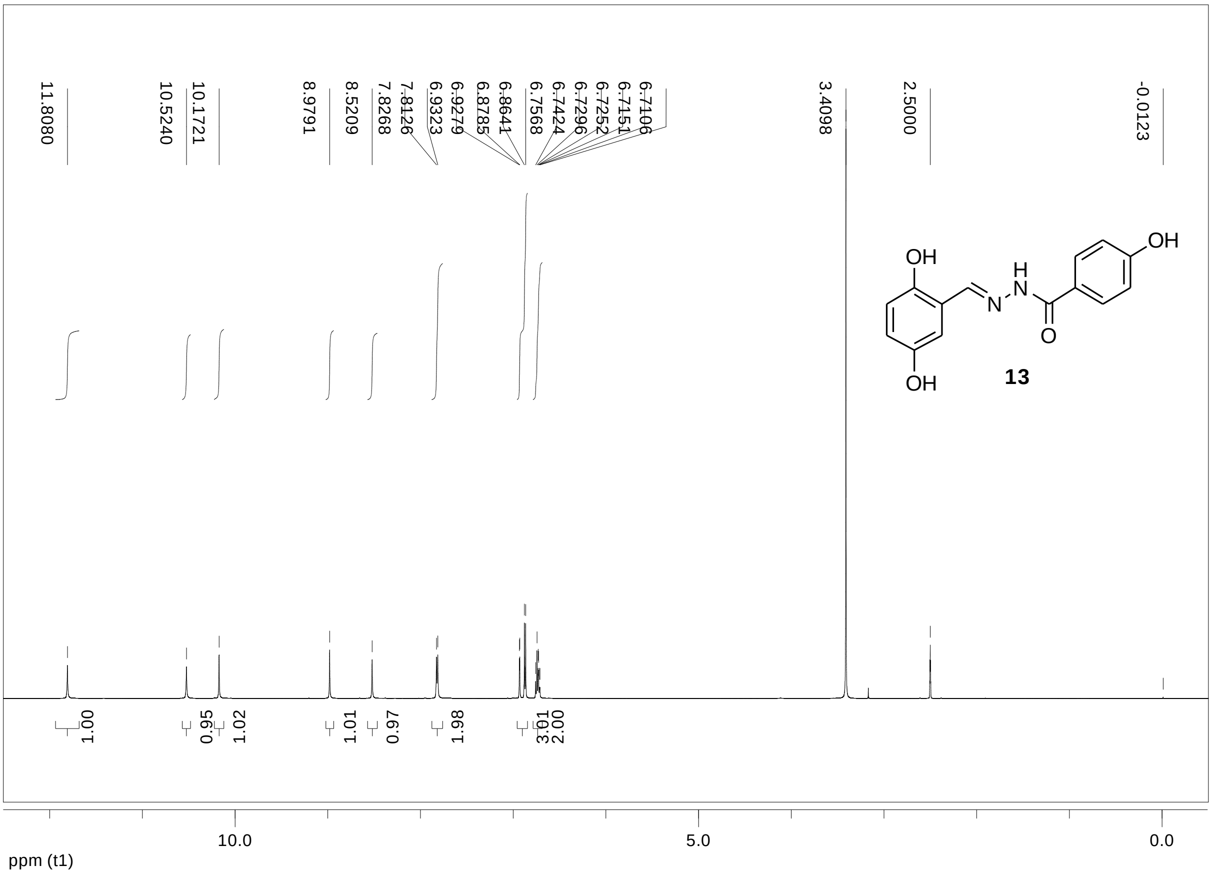

Figure S24. ^{1}H-NMR (601 MHz, DMSO-d_6) spectrum of gentisaldehyde derivative **13**.

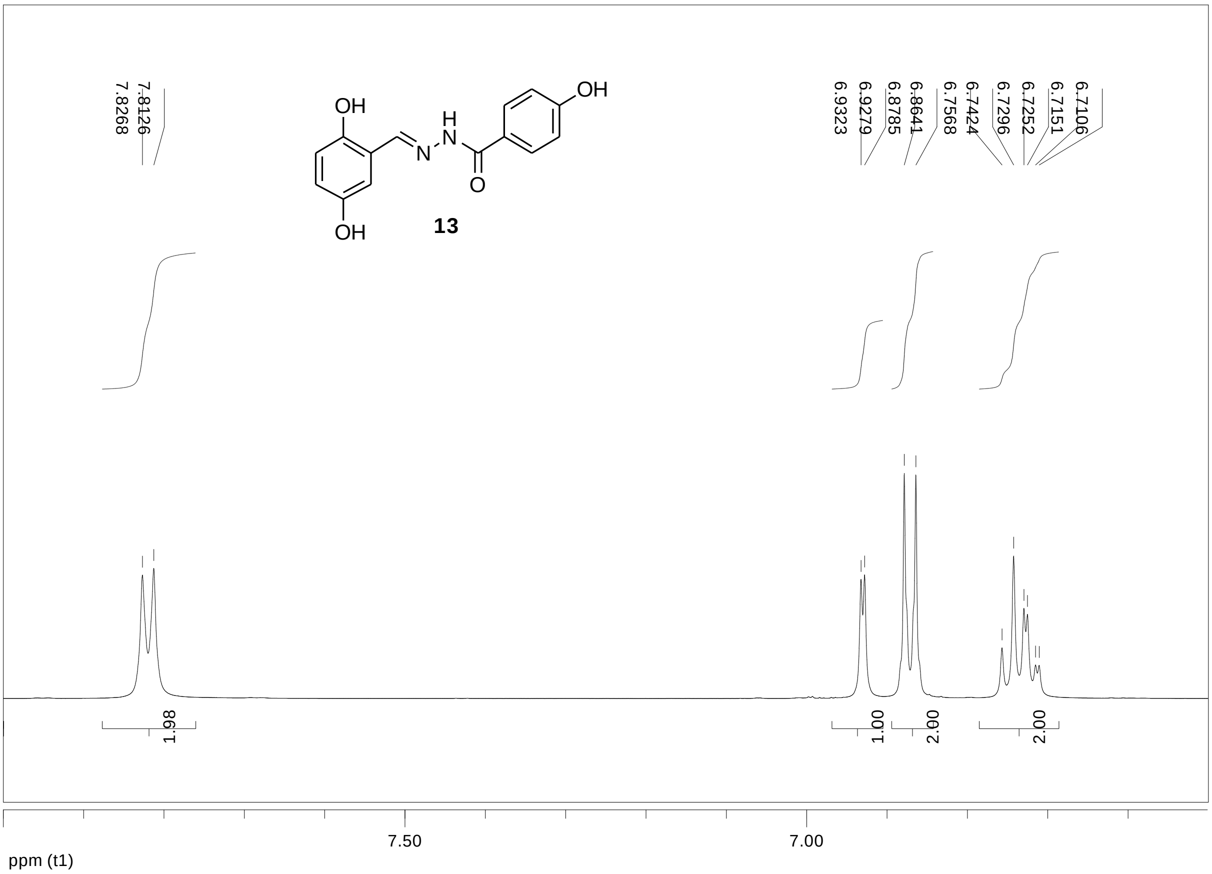

Figure S25. Expansion of ^{1}H-NMR (601 MHz, DMSO-d_6) spectrum of gentisaldehyde derivative **13**.

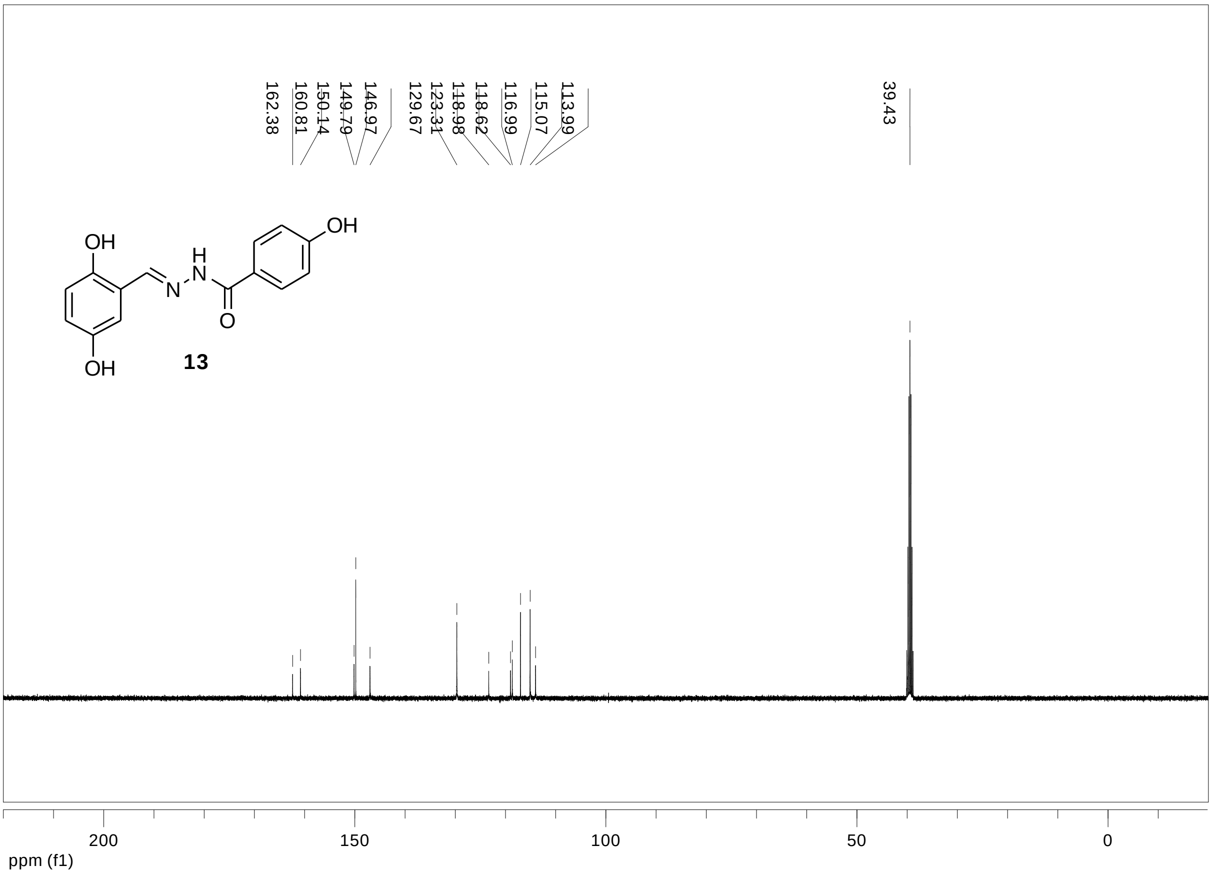

Figure S26. ^{13}C-NMR (101 MHz, DMSO-d_6) spectrum of gentisaldehyde derivative **13**.

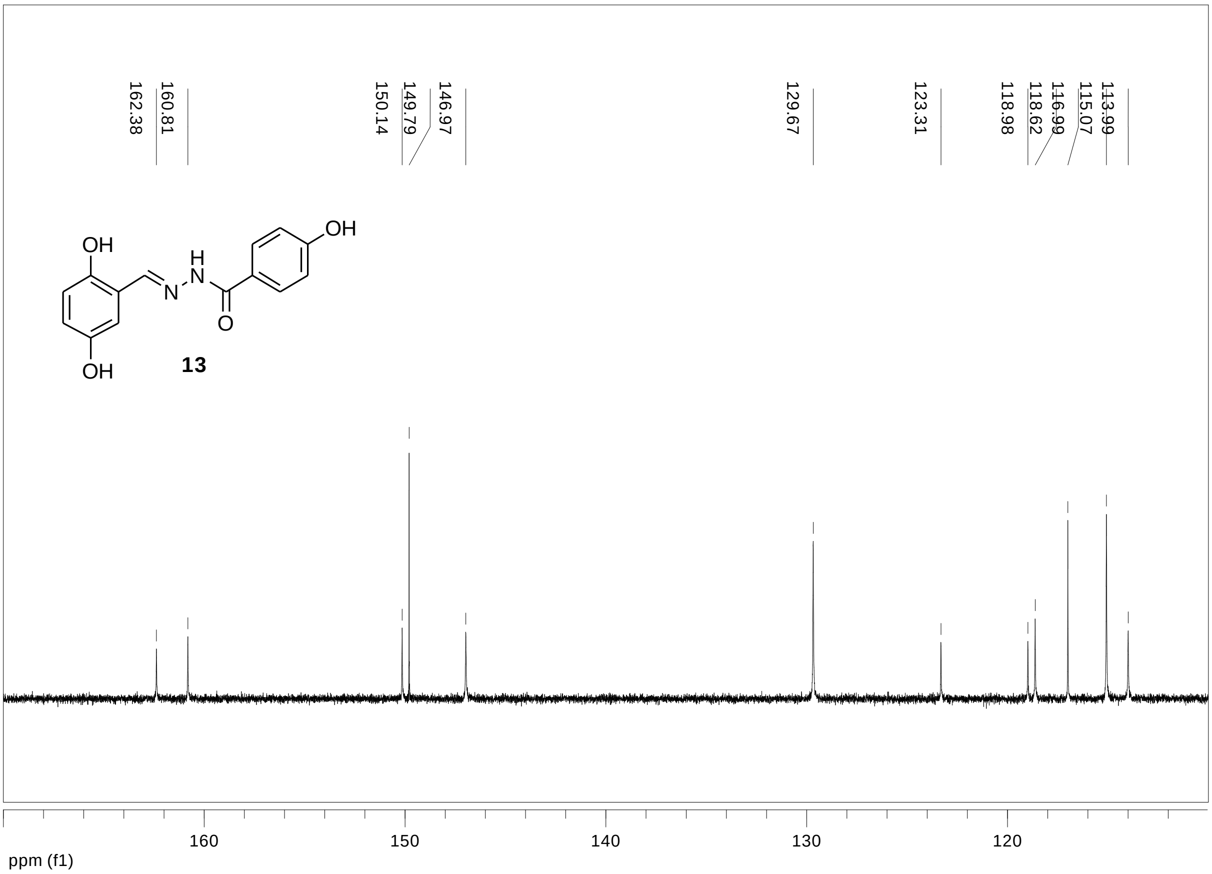

Figure S27. Expansion of ^{13}C-NMR (101 MHz, DMSO-d_6) spectrum of gentisaldehyde derivative **13**.

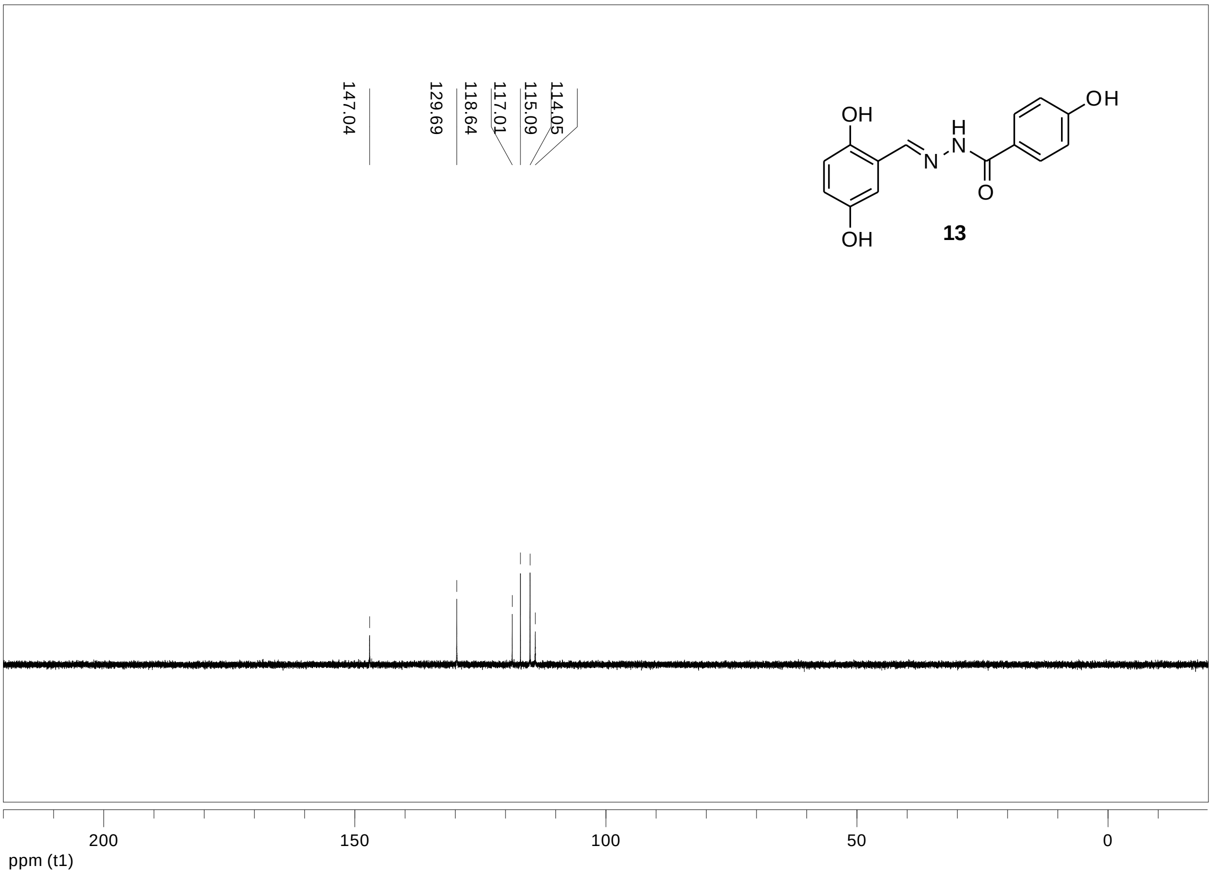

Figure S28. ^{13}C-NMR (101 MHz, DMSO-d_6) dept-135 experiment of gentisaldehyde derivative **13**.

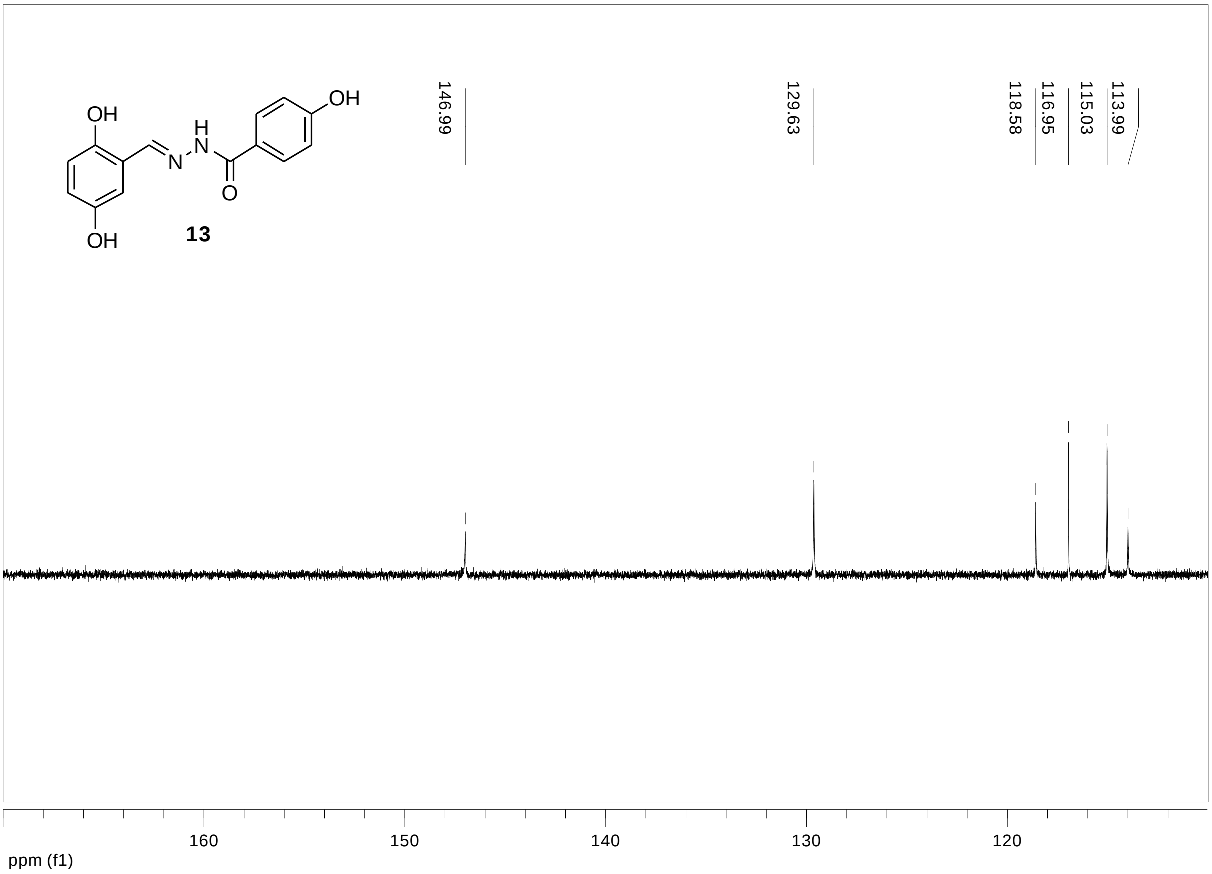

Figure S29. Expansion of ^{13}C-NMR (101 MHz, DMSO-d_6) dept-135 experiment of derivative **13**.

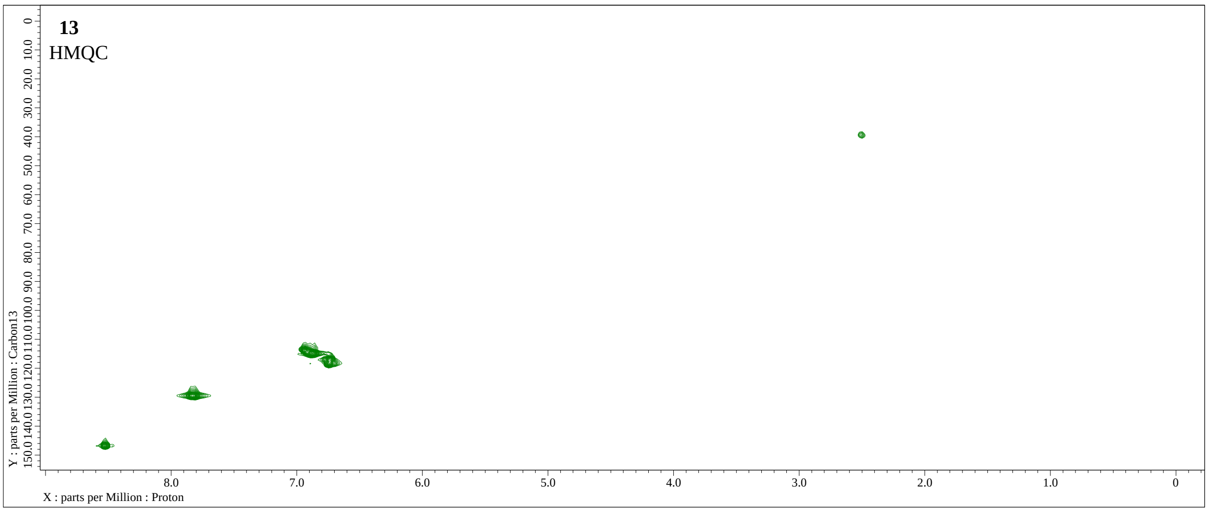

Figure S30. 2D-NMR (400 MHz, DMSO-d_6) HMQC experiment of gentisaldehyde derivative – 4-hydroxy-*N*'-[(*E*)-(2,5-dihydroxyphenyl)methylidene]benzohydrazide (**13**).

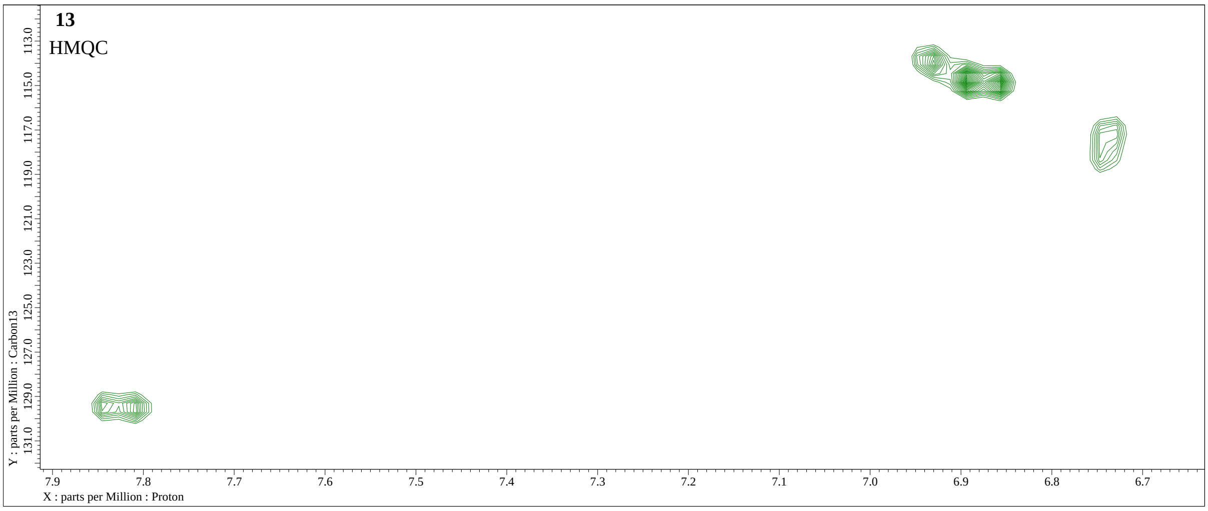

Figure S31. Expansion of 2D-NMR (400 MHz, DMSO-d_6) HMQC experiment of gentisaldehyde derivative – 4-hydroxy-*N*'-[(*E*)-(2,5-dihydroxyphenyl)methylidene]benzohydrazide (**13**).

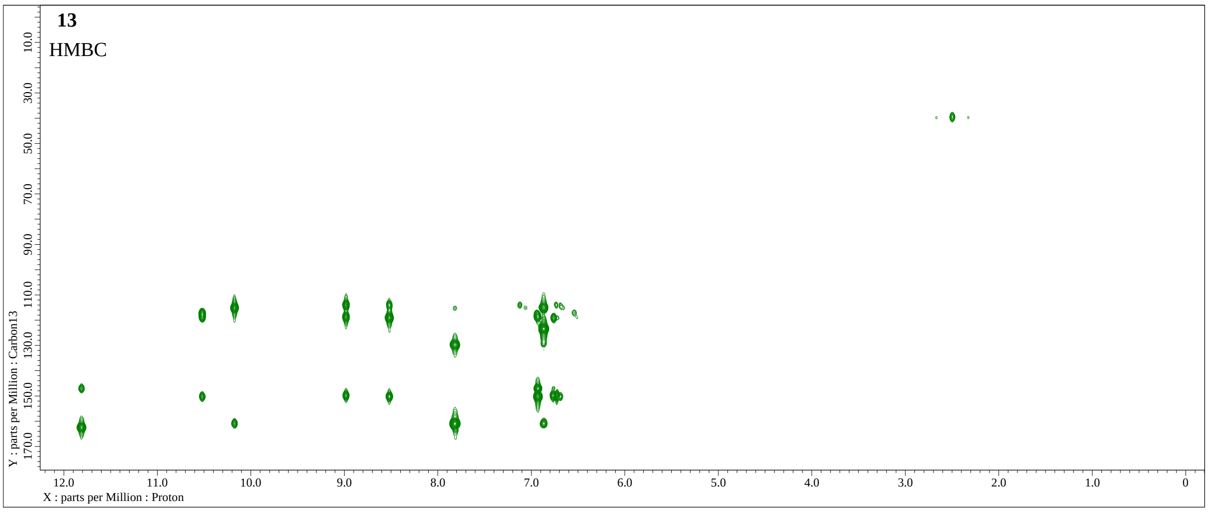

Figure S32. 2D-NMR (400 MHz, DMSO-d_6) HMBC of gentisaldehyde derivative – 4-hydroxy-*N*'-[(*E*)-(2,5-dihydroxyphenyl)methylidene]benzohydrazide (**13**).

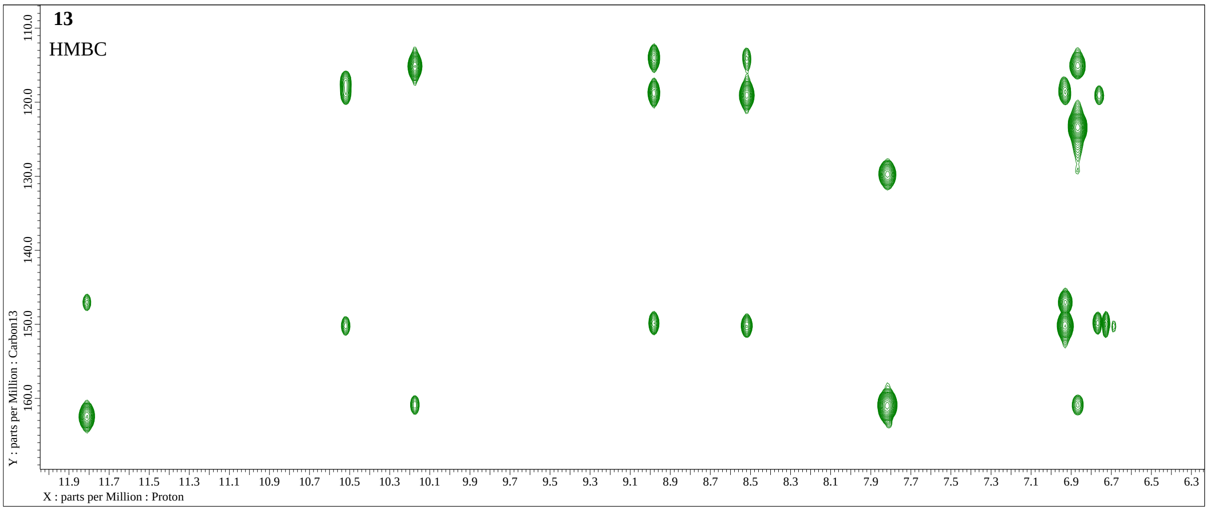

Figure S33. Expansion of 2D-NMR (400 MHz, DMSO-d_6) HMBC experiment of gentisaldehyde derivative – 4-hydroxy-*N*'-[(*E*)-(2,5-dihydroxyphenyl)methylidene]benzohydrazide (**13**).

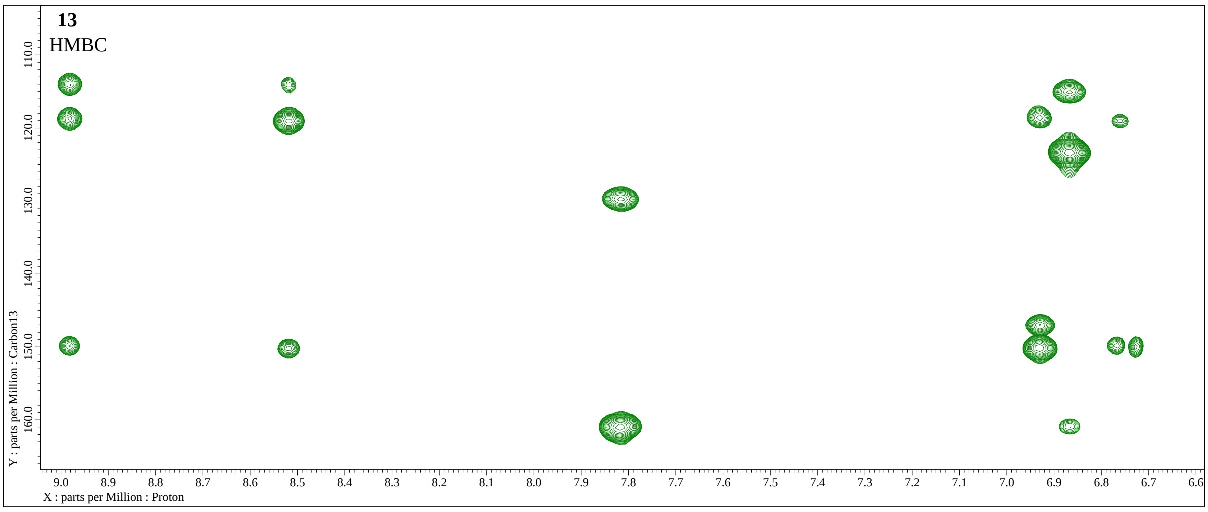

Figure S34. Expansion of 2D-NMR (400 MHz, DMSO-d_6) HMBC experiment of gentisaldehyde derivative – 4-hydroxy-*N*'-[(*E*)-(2,5-dihydroxyphenyl)methylidene]benzohydrazide (**13**).

N'-[(E)-(2,4-dihydroxyphenyl)methylidene]benzohydrazide (22).

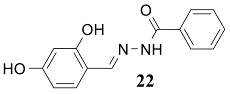

The general procedure starting from 2,4-dihydroxybenzaldehyde (**59**) (276 mg, 2.0 mmol), benzohydrazide (**39**) (272 mg, 2.0 mmol), CH_3OH (20 mL), and AcOH (0.20 mL) was employed with a 3.5 hours reaction time. The reaction mixture was concentrated before crystallization by distillation, and the solid formed was washed with a frozen solution of methanol – water (3 × 1.5 mL, 3:2, *v*/*v*) to obtain the N'-[(E)-(2,4-dihydroxy-phenyl)methylidene]benzohydrazide (**22**). A beige fine crystalline; 456 mg, 1.8 mmol, 89% yield; m.p. 255–256 °C with decomposition (from CH_3OH) (m.p. 249–250 °C [14]); selected FT-IR (ATR): ν_{max} 3253 (O-H), 3056 (vbr, N-H, O-H), 1650 (C=C), 1630 (C=O), 1603 (N-H), 1552 (CH=N), 1518, 1462, 1447, 1328, 1280 (C_{Ar}-H), 1258, 1242, 1219 (C_{Ar}-O), 1182, 1116, 1079, 984, 969 (N-N), 910, 851, 713, 682, 640, 522, 408 cm^{-1}; ^{1}H-NMR (601 MHz, DMSO-d_6) [19]: δ 11.92 (s, 1H, NH), 11.49 (s, 1H, Ar-2-OH), 9.96 (s, 1H, Ar-4-OH), 8.51 (s, 1H, CH=N), 7.92 (dd, ^3J = 7.8 Hz, ^4J = 1.4 Hz, 2H, H-2,6), 7.60 (tt, ^3J = 7.3 Hz, ^4J = 1.4 Hz, 1H, H-4), 7.53 (dd, ^3J = 7.8 Hz, ^3J = 7.3 Hz, 2H, H-3,5), 7.31 (d, ^3J = 8.4 Hz, 1H, ArH-6), 6.37 (dd, ^3J = 8.4 Hz, ^4J = 2.3 Hz, 1H, ArH-5), 6.32 (d, ^4J = 2.3 Hz, 1H, ArH-3) ppm; ^{13}C-NMR (101 MHz, DMSO-d_6): δ 162.46 (C=O), 160.68 (ArC-4), 159.46 (ArC-2), 149.17 (CH=N), 132.93 (C-1), 131.75 (C-4), 131.33 (ArC-6), 128.45 (C-3,5), 127.49 (C-2,6), 110.47 (ArC-1), 107.65 (ArC-5), 102.62 (ArC-3) ppm; HRMS (TOF, MS, ESI): *m*/*z* for $C_{14}H_{12}N_2O_3$ + Na^+ calculated: 279.0740; found: 279.0739.

The ^{1}H-NMR spectrum is consistent with the literature value [19], and the FT-IR and ^{13}C-NMR spectroscopic data are consistent with the literature values [14].

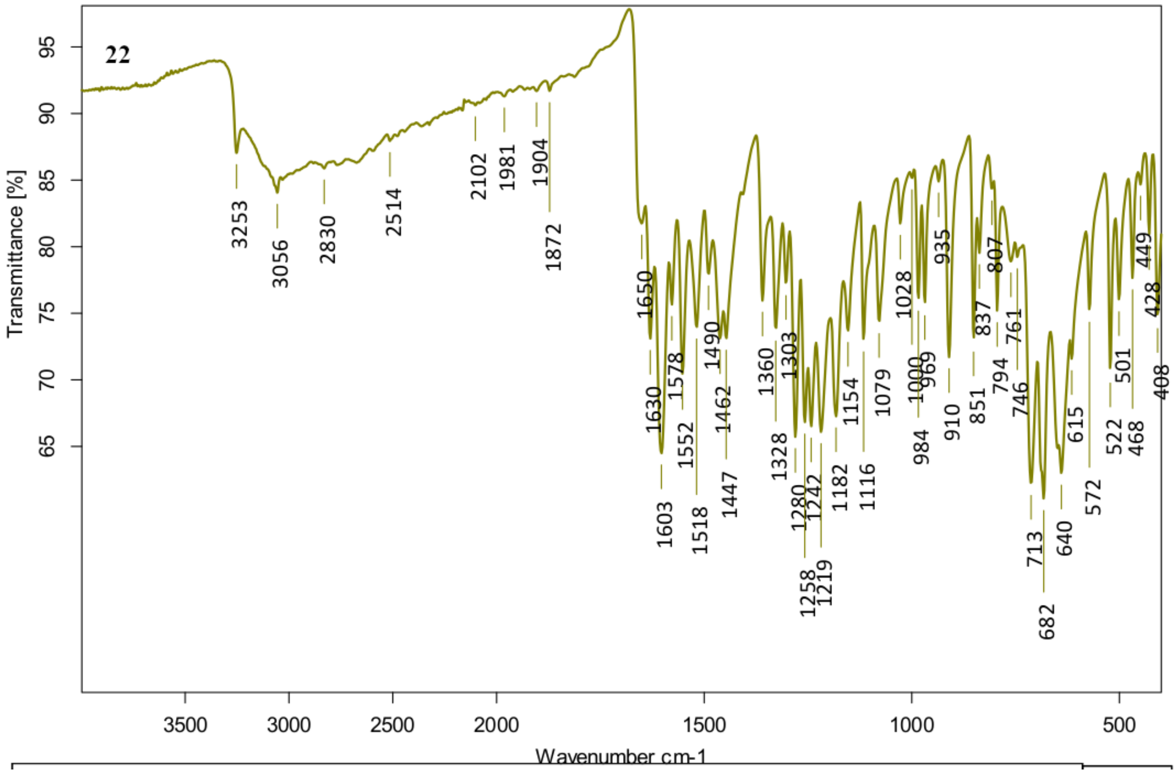

Figure S35. FT-IR (ATR) (4000–400 cm^{-1}) spectrum of N'-[(E)-(2,4-dihydroxy-phenyl)methylidene]benzohydrazide (**22**).

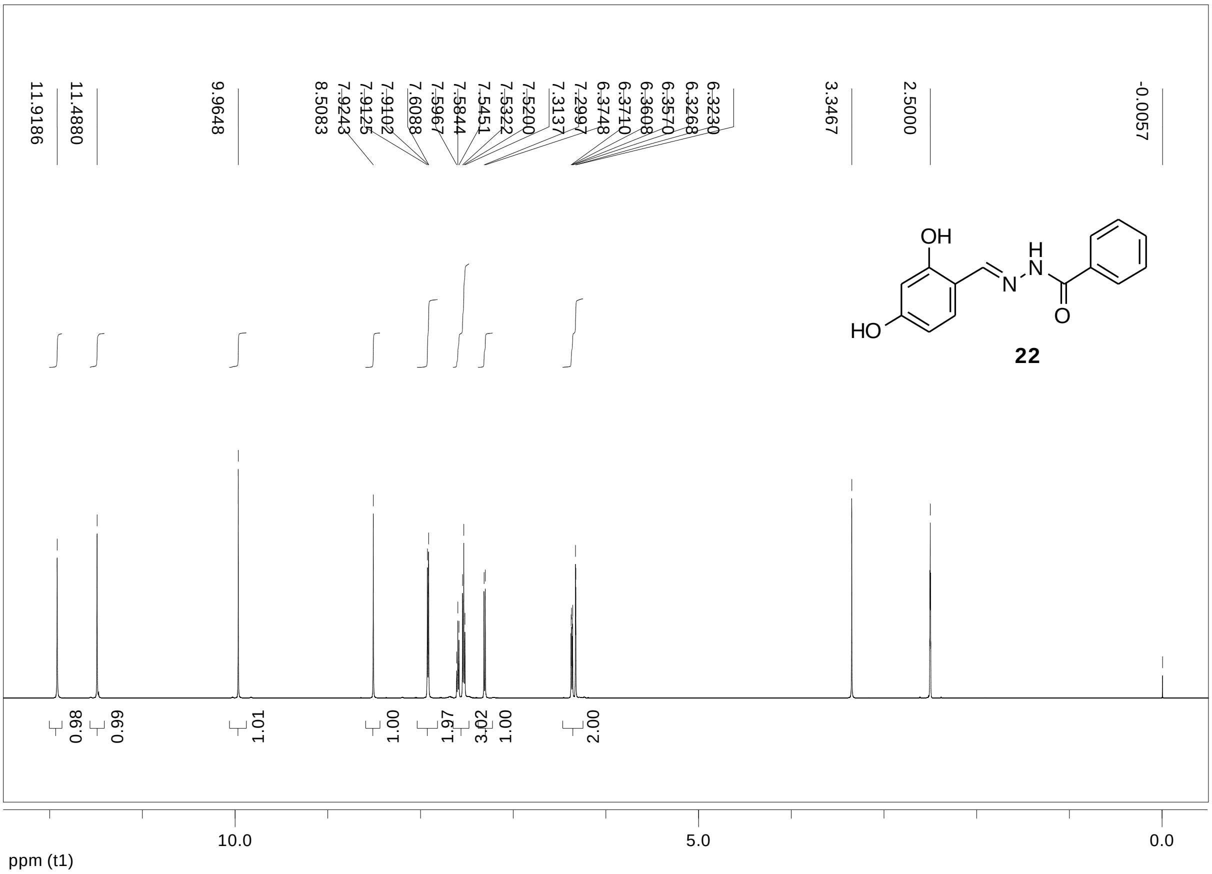

Figure S36. ^{1}H-NMR (601 MHz, DMSO-d_6) spectrum of hydrazide-hydrazone **22**.

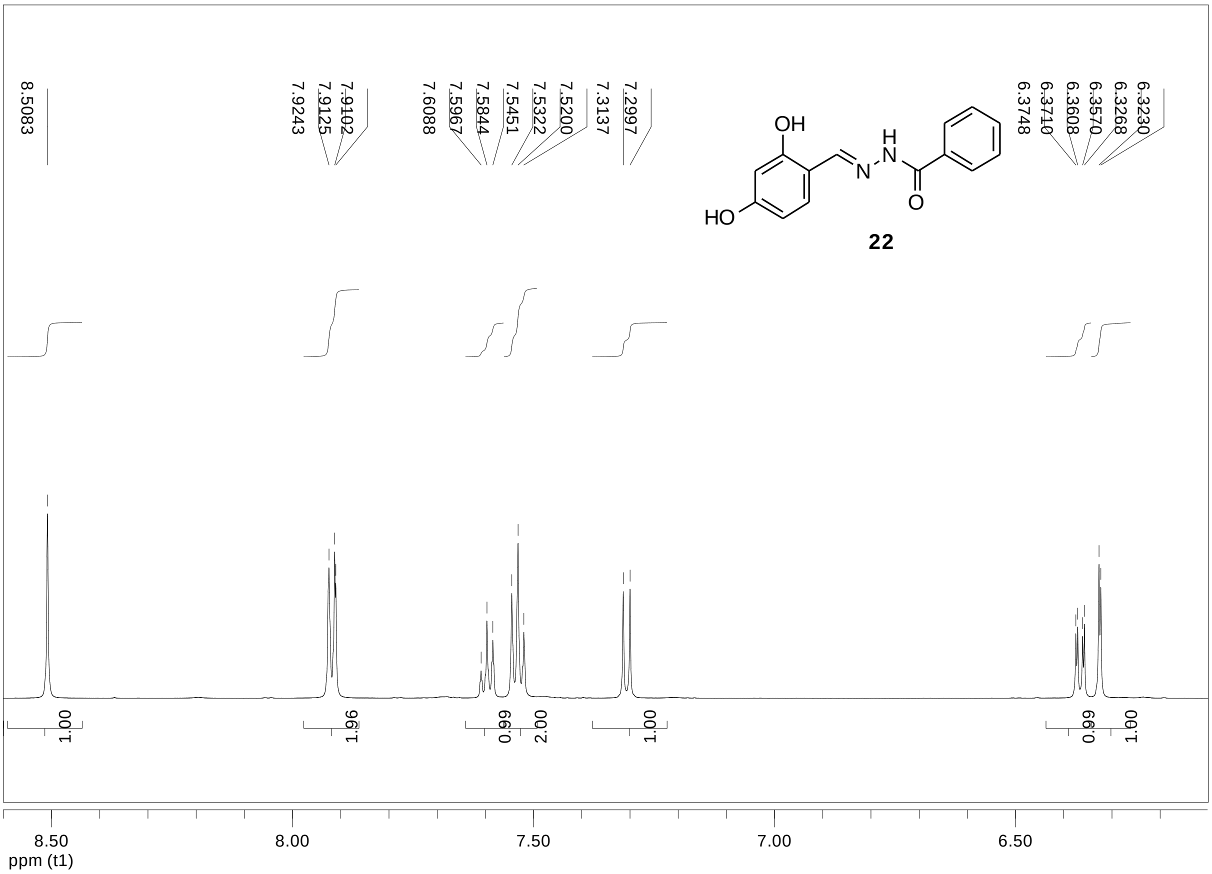

Figure S37. Expansion of ^{1}H-NMR (601 MHz, DMSO-d_6) spectrum of hydrazide-hydrazone **22**.

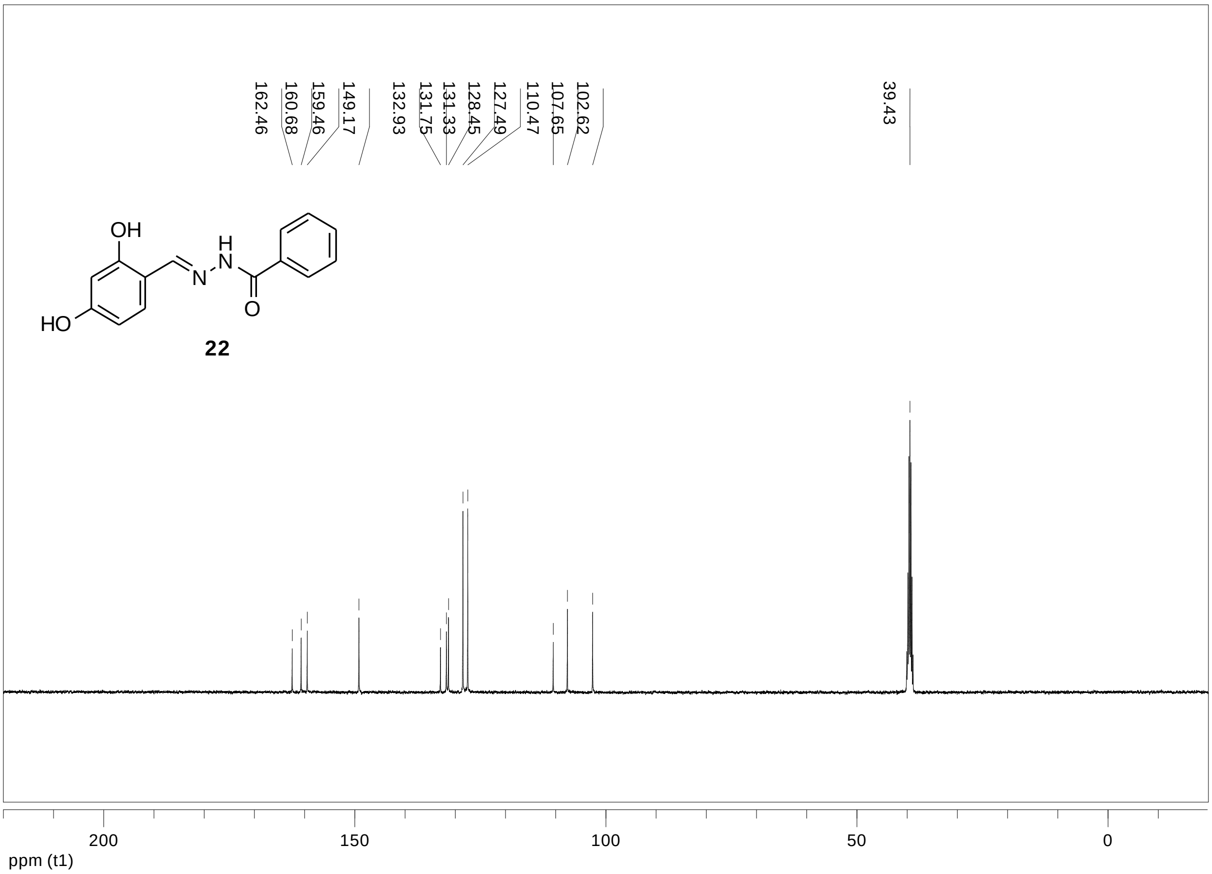

Figure S38. ^{13}C-NMR (101 MHz, DMSO-d_6) spectrum of hydrazide-hydrazone **22**.

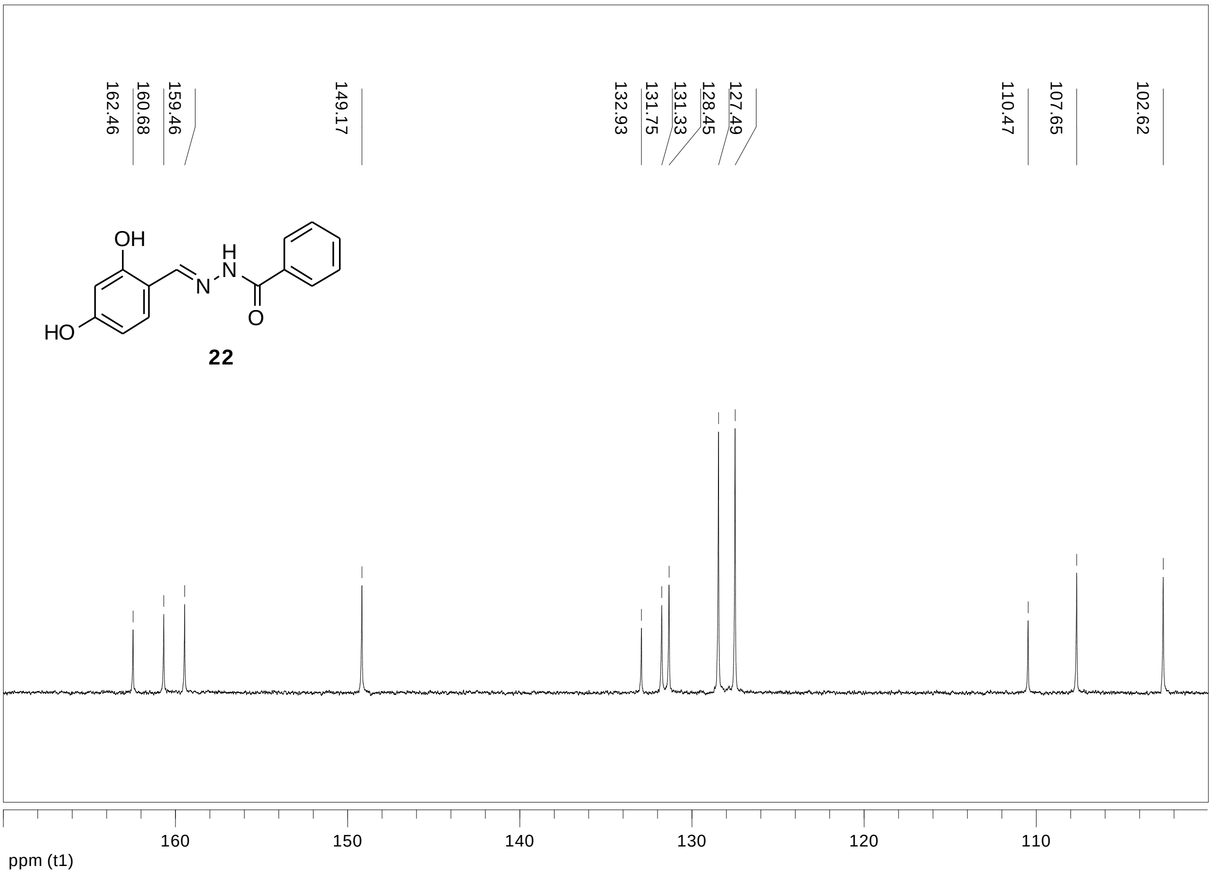

Figure S39. Expansion of ^{13}C-NMR (101 MHz, DMSO-d_6) spectrum of hydrazide-hydrazone **22**.

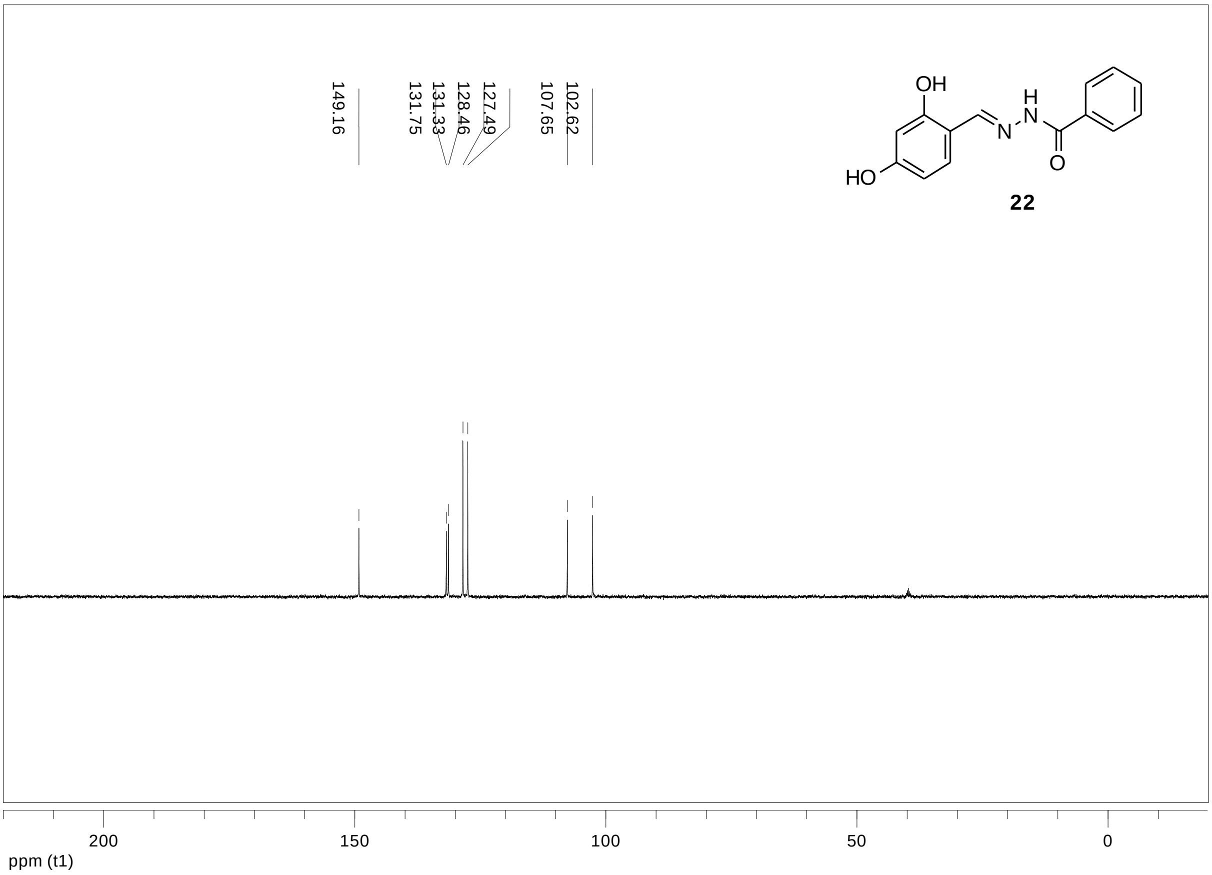

Figure S40. ^{13}C-NMR (101 MHz, DMSO-d_6) dept-135 experiment of hydrazide-hydrazone **22**.

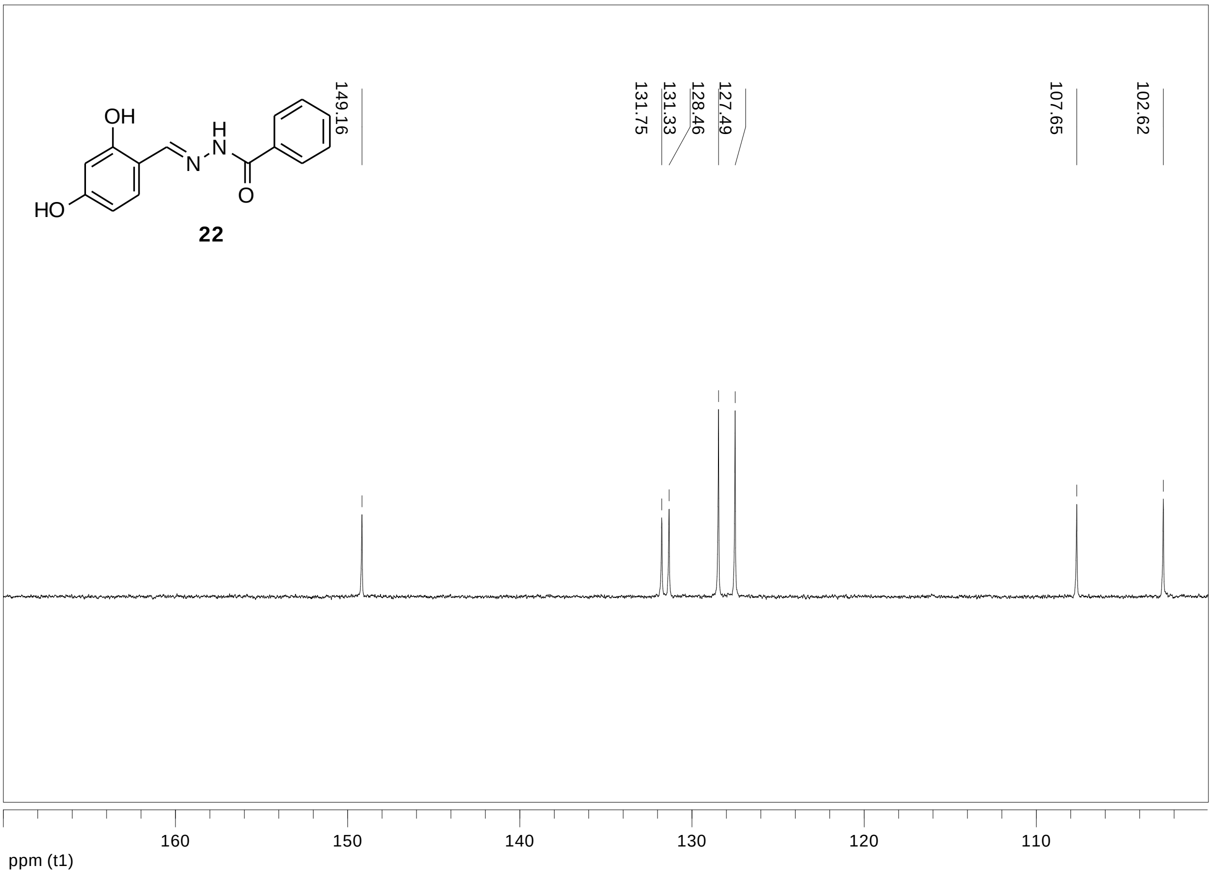

Figure S41. Expansion of ^{13}C-NMR (101 MHz, DMSO-d_6) dept-135 experiment of hydrazone **22**.

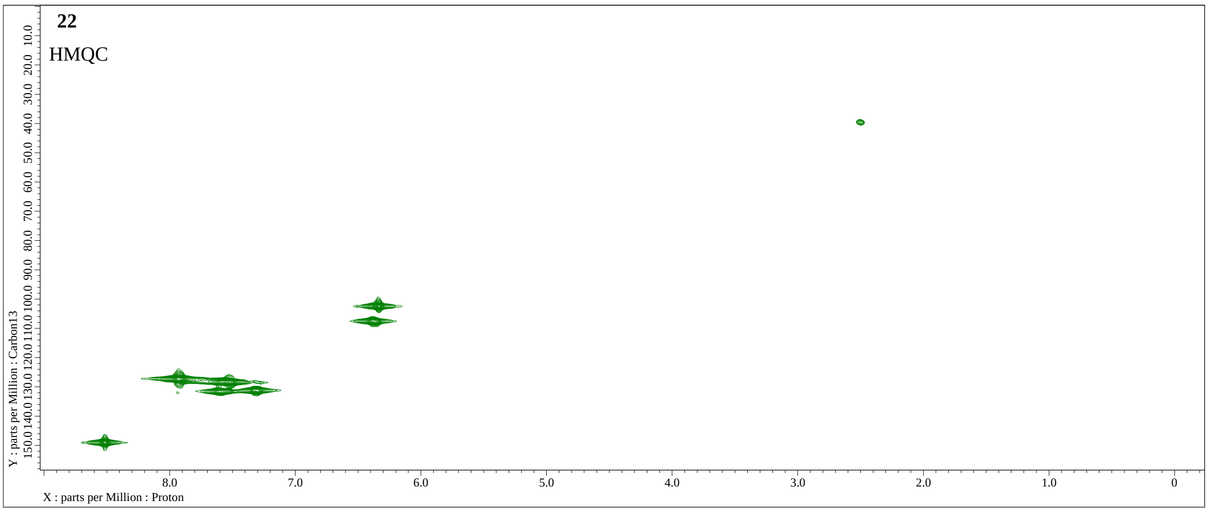

Figure S42. 2D-NMR (400 MHz, DMSO-d_6) HMQC experiment of *N*'-[(*E*)-(2,4-dihydroxyphenyl)methylidene]benzohydrazide (**22**).

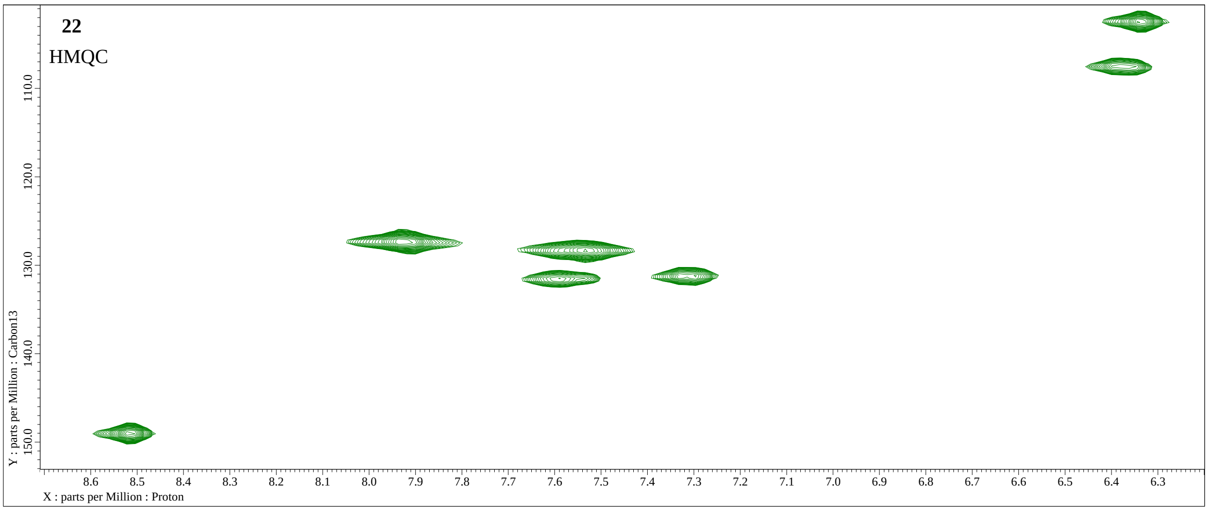

Figure S43. Expansion of 2D-NMR (400 MHz, DMSO-d_6) HMQC experiment of *N*'-[(*E*)-(2,4-dihydroxyphenyl)methylidene]benzohydrazide (**22**).

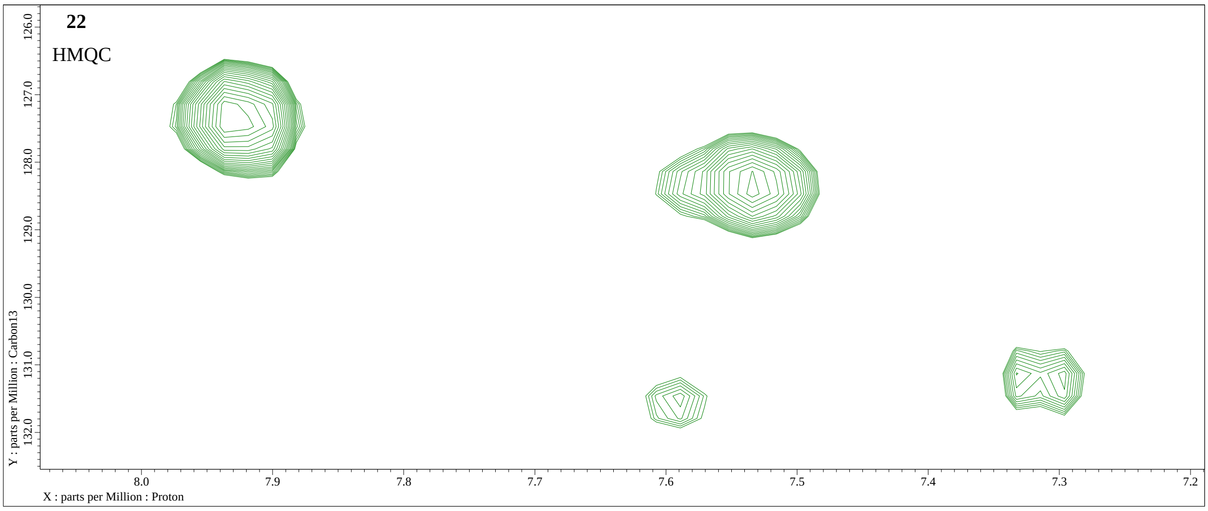

Figure S44. Expansion of 2D-NMR (400 MHz, DMSO-d_6) HMQC experiment of *N*'-[(*E*)-(2,4-dihydroxyphenyl)methylidene]benzohydrazide (**22**).

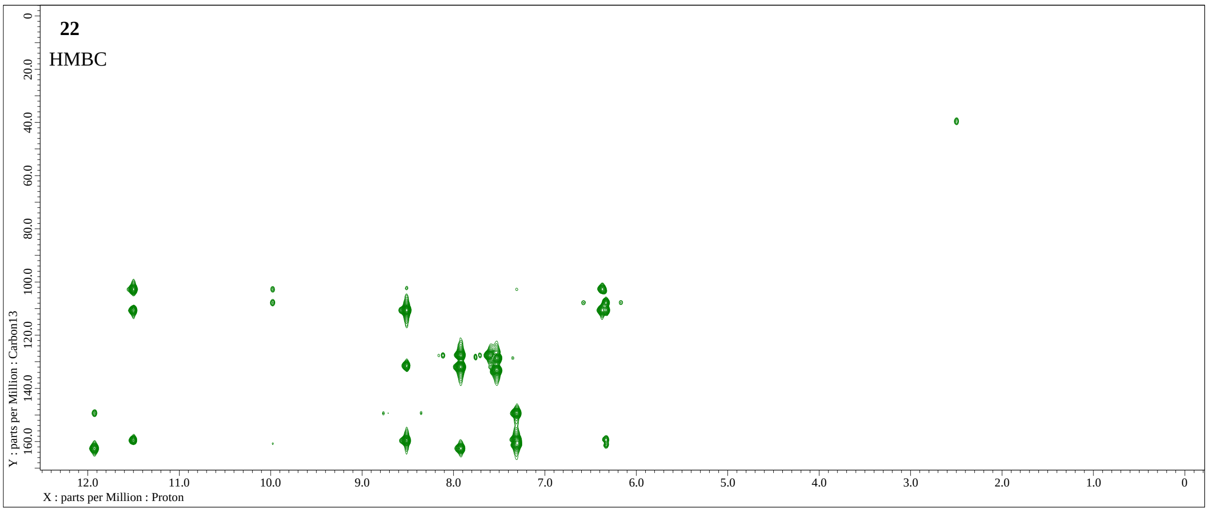

Figure S45. 2D-NMR (400MHz, DMSO-d_6) HMBC experiment of *N*'-[(*E*)-(2,4-dihydroxyphenyl)methylidene]benzohydrazide (**22**).

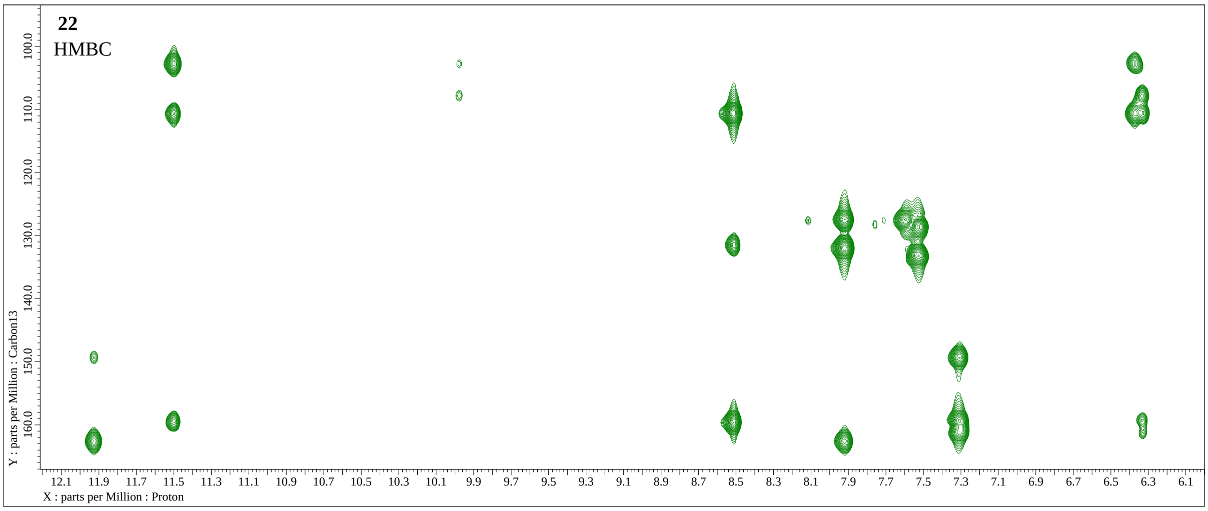

Figure S46. Expansion of 2D-NMR (400 MHz, DMSO-d_6) HMBC experiment of*N*'-[(*E*)-(2,4-dihydroxyphenyl)methylidene]benzohydrazide (**22**).

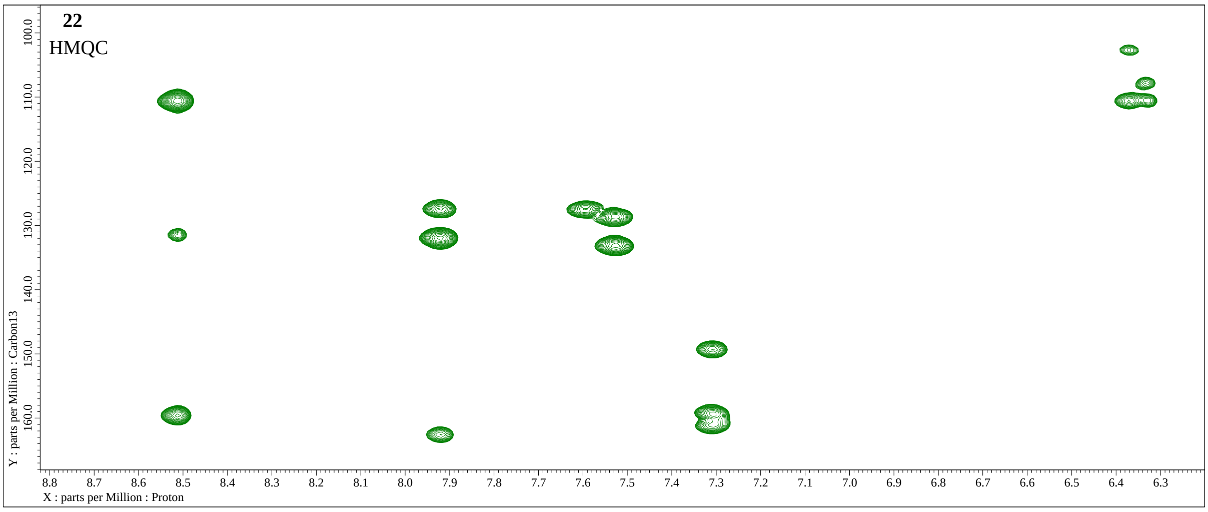

Figure S47. Expansion of 2D-NMR (400 MHz, DMSO-d_6) HMBC experiment of *N*'-[(*E*)-(2,4-dihydroxyphenyl)methylidene]benzohydrazide (**22**).

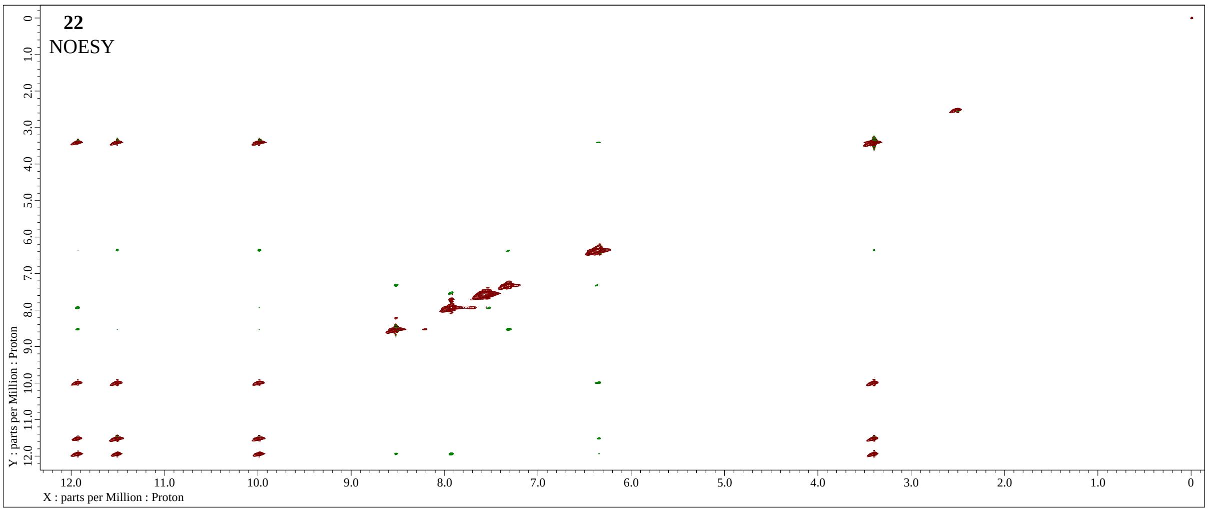

Figure S48. ^{1}H-^{1}H NMR (400 MHz, DMSO-d_6) NOESY experiment of *N*'-[(*E*)-(2,4-dihydroxyphenyl)methylidene]benzohydrazide (**22**).

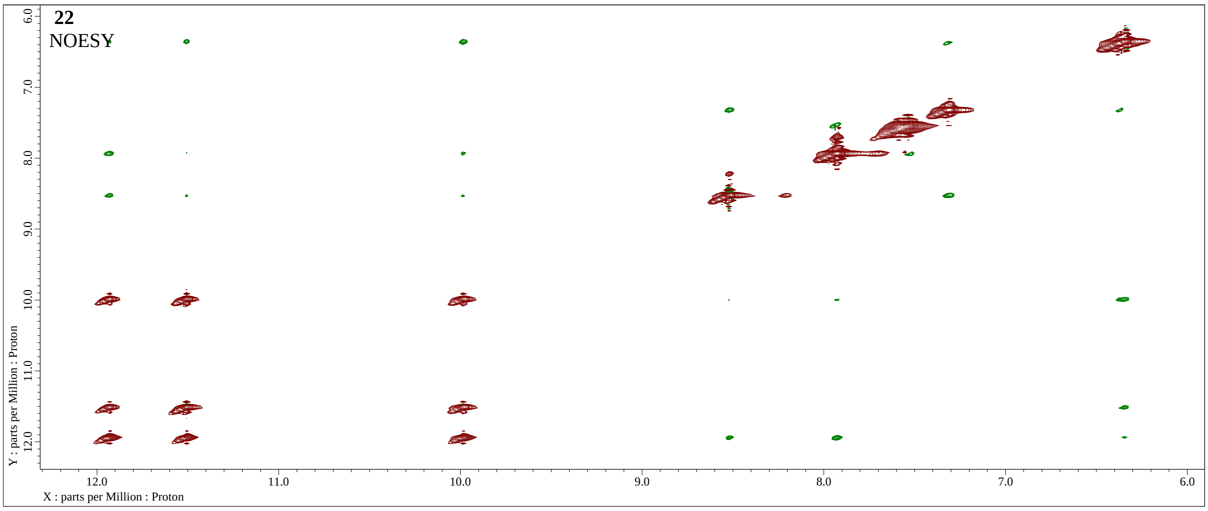

Figure S49. Expansion of ^{1}H-^{1}H NMR (400 MHz, DMSO-d_6) NOESY experiment of *N*'-[(*E*)-(2,4-dihydroxyphenyl)methylidene]benzohydrazide (**22**).

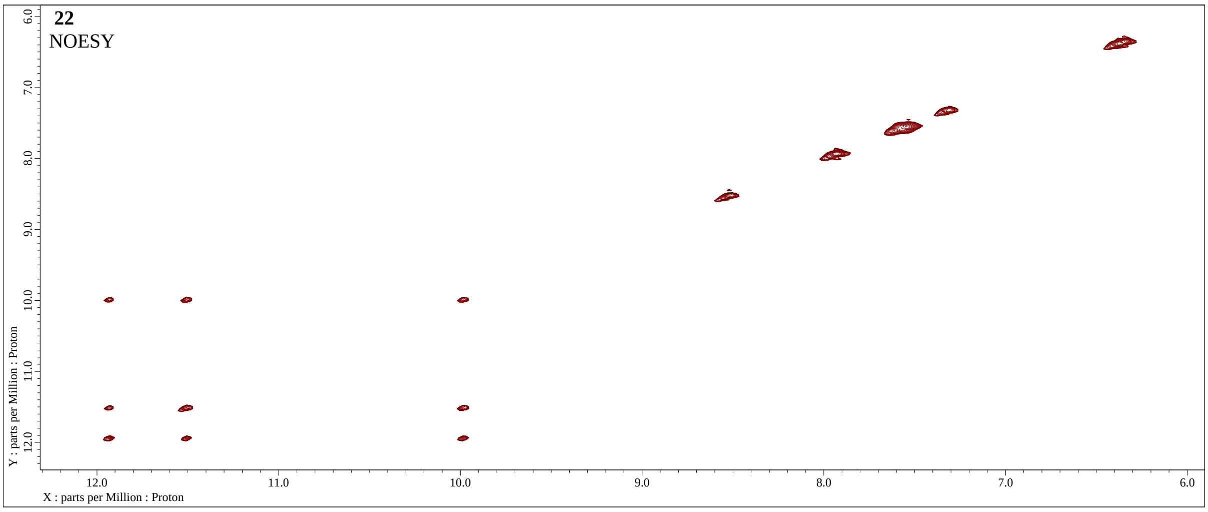

Figure S50. Expansion of ^{1}H-^{1}H NMR (400 MHz, DMSO-d_6) NOESY experiment of *N*'-[(*E*)-(2,4-dihydroxyphenyl)methylidene]benzohydrazide (**22**).

4-Hydroxy-*N*'-[(*E*)-(2,4,5-trihydroxyphenyl)methylidene]benzohydrazide (23) [20]**.**

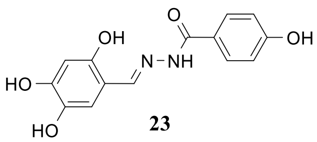

The general procedure starting from 2,4,5-trihydroxybenzaldehyde (**60**) (308 mg, 2.0 mmol) [6], 4-hydroxybenzohydrazide (**36**) (304 mg, 2.0 mmol), CH_3OH (8.5 mL), and AcOH (0.10 mL) was employed with a 5 h reaction time to obtain the 4-hydroxy-*N*'-[(*E*)-(2,4,5-trihydroxyphenyl)methylidene]benzohydrazide (**23**). A yellow powder; 532 mg, 1.86 mmol, 92% yield, which turns brown at 230 °C; m.p. 253 °C with decomposition (CH_3OH); selected FT-IR (ATR): ν_{max} 3286 (N-H, O-H), 3164 (br, O-H), 1621 (C=O), 1611 (C=C), 1591 (N-H), 1575 (C=N), 1506, 1445, 1294, 1262 (C_{Ar}-O), 1238 (C_{Ar}-O), 1183 (C_{Ar}-O), 1161 (C_{Ar}-O), 1136, 1101, 960 (N-N), 911, 842, 742, 619, 543, 495, 442 cm^{-1}; ^{1}H-NMR (400 MHz, DMSO-d_6): δ 11.60 (s, 1H, NH), 10.75 (s, 1H, Ar-2-OH), 10.12 (s, 1H, C-4-OH), 9.53 (s, 1H, Ar-4-OH), 8.54 (s, 1H, Ar-5-OH), 8.40 (s, 1H, CH=N), 7.79 (d, ^3J = 8.7 Hz, 2H, H-2,6), 6.85 (d, ^3J = 8.7 Hz, 2H, H-3,5), 6.84 (s, 1H, ArH-6), 6.33 (s, 1H, ArH-3) ppm; ^{13}C-NMR (101 MHz, DMSO-d_6): δ 162.02 (C=O), 160.60 (C-4), 151.80 (ArC-2), 149.00 (ArC-5), 147.84 (CH=N), 138.37 (ArC-4), 129.50 (C-2,6), 123.54 (C-1), 115.00 (C-3,5), 114.82 (ArC-6), 109.44 (ArC-1), 103.41 (ArC-3) ppm; HRMS (TOF, MS, ESI): *m*/*z* for $C_{14}H_{12}N_2O_5 + Na^+$ calculated: 311.0638; found: 311.0641.

The ^{1}H-NMR spectrum is consistent with the literature value [20].

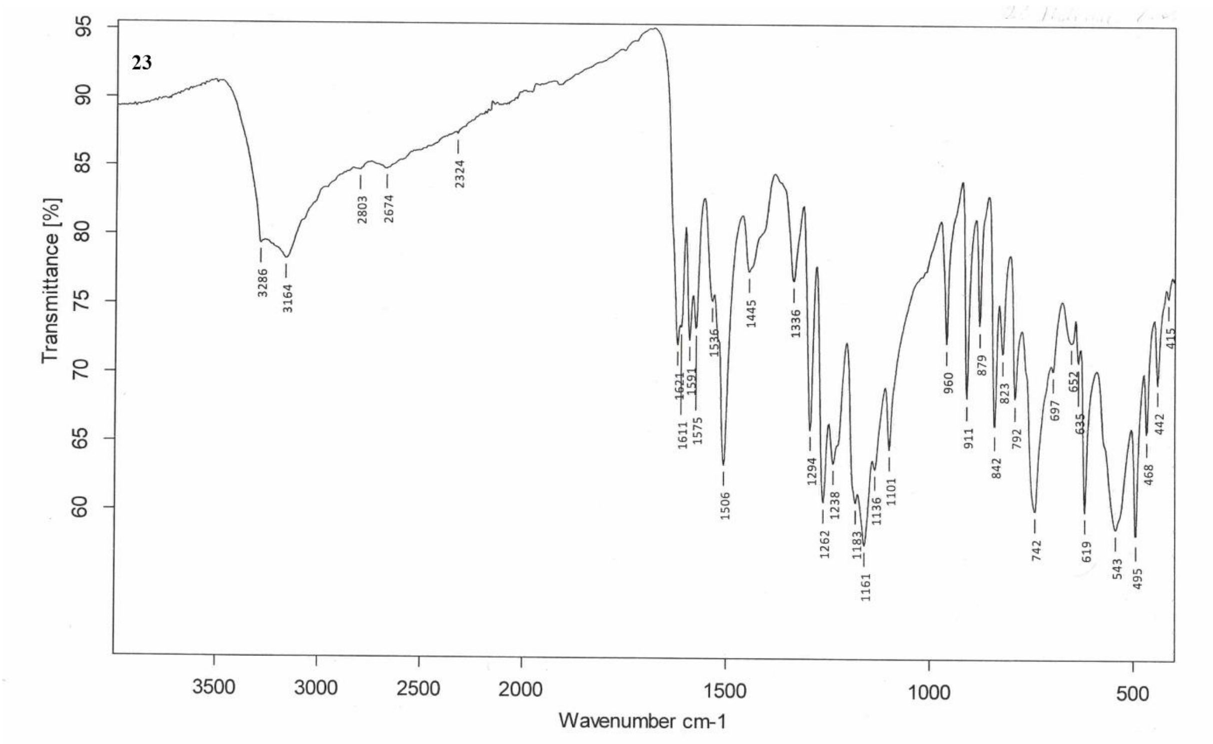

Figure S51. FT-IR (ATR) (4000–400 cm^{-1}) spectrum of 4-hydroxy-*N*'-[(*E*)-(2,4,5-trihydroxy-phenyl)methylidene]benzohydrazide (**23**).

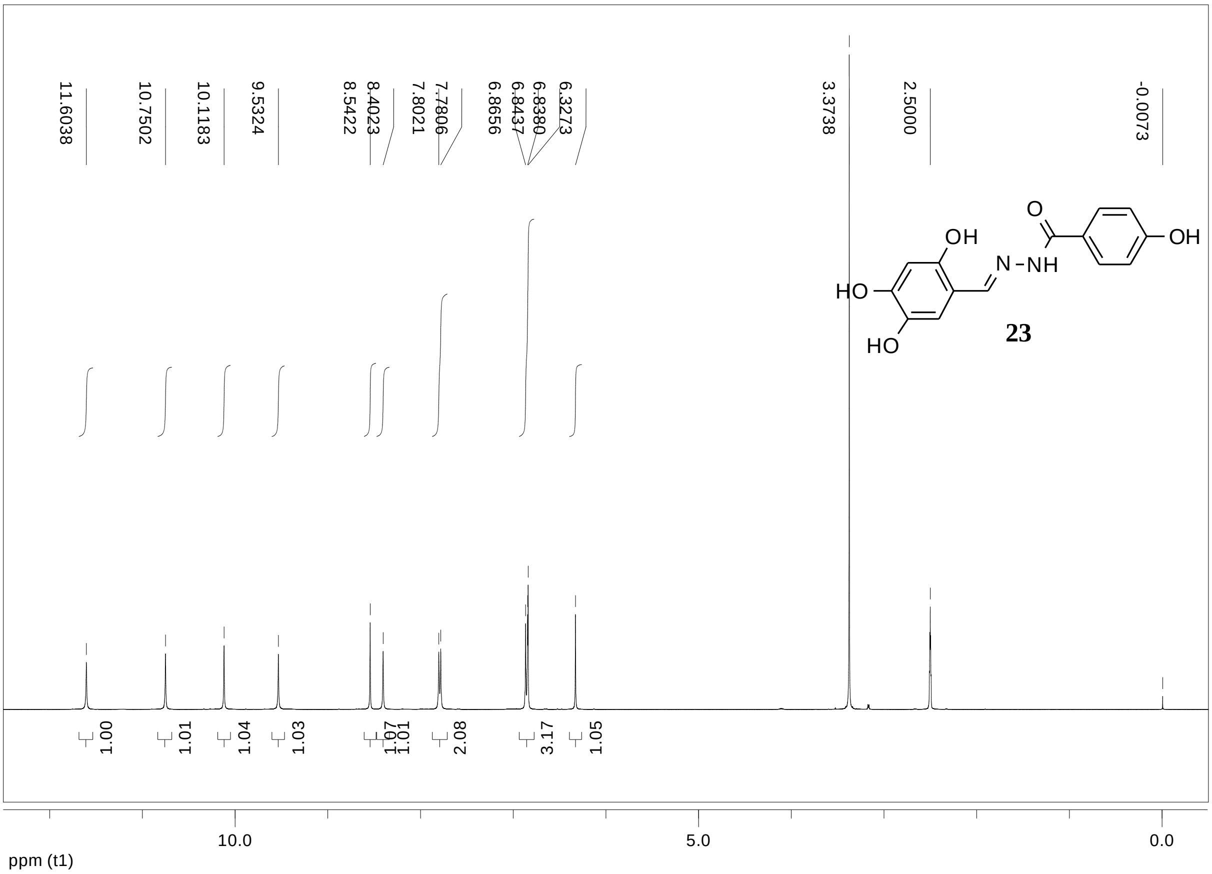

Figure S52. ^{1}H-NMR (400 MHz, DMSO-d_6) spectrum of hydrazide-hydrazone **23**.

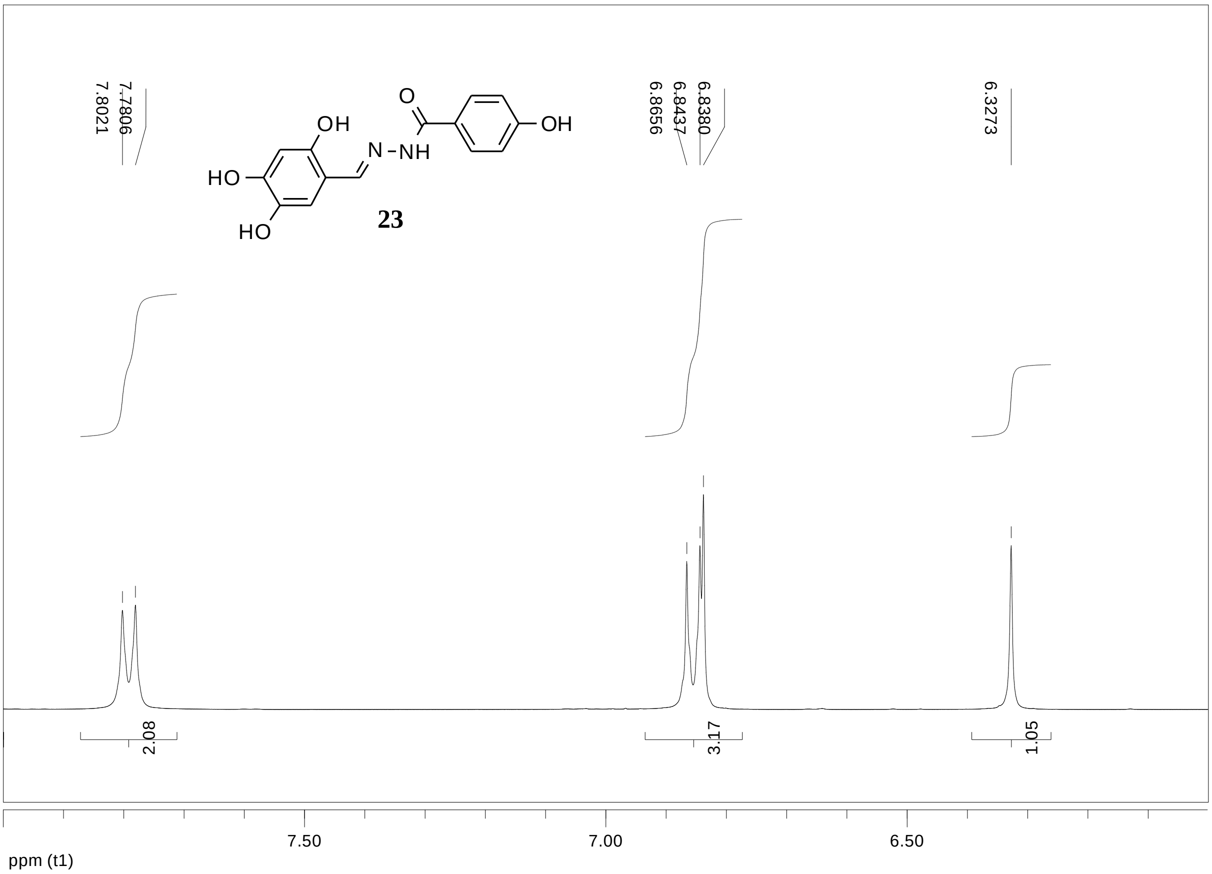

Figure S53. Expansion of ^{1}H-NMR (400 MHz, DMSO-d_6) spectrum of hydrazide-hydrazone **23**.

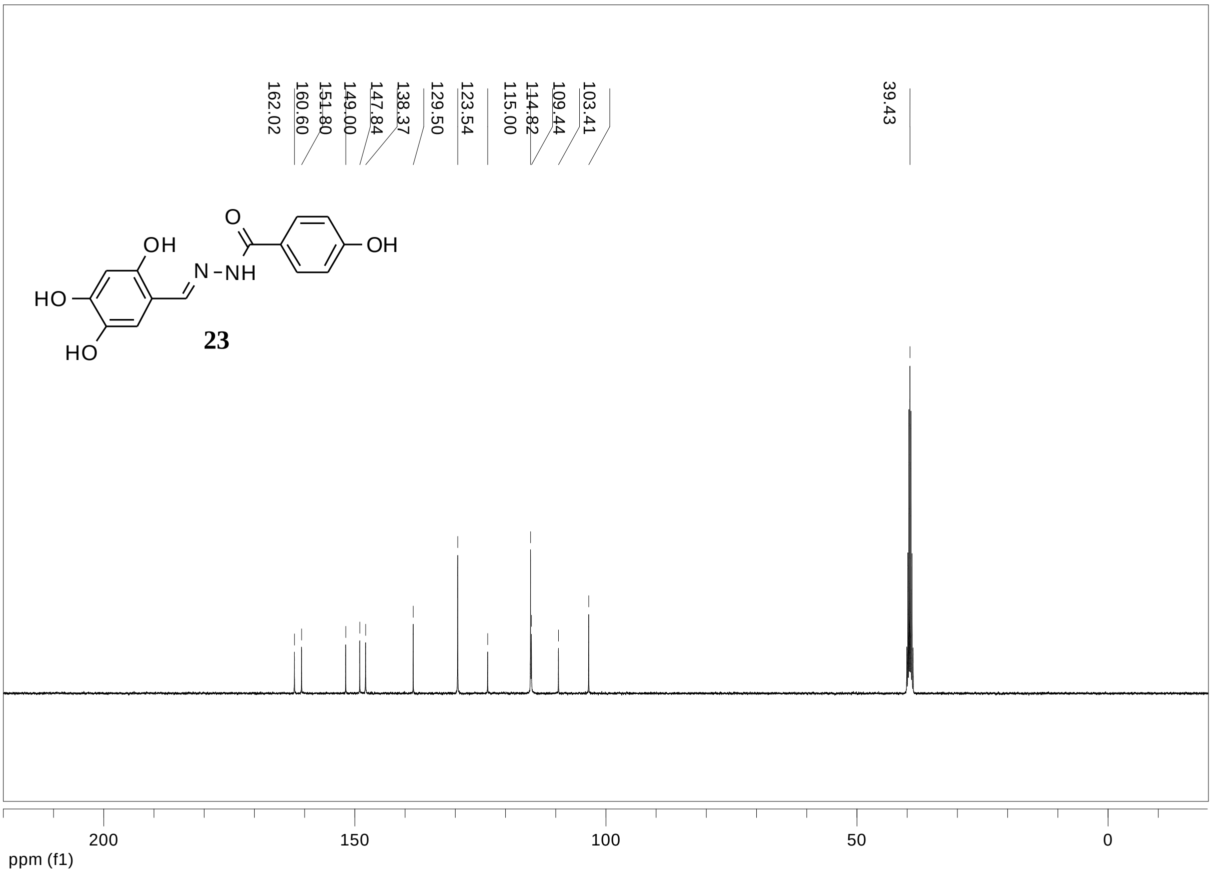

Figure S54. ^{13}C-NMR (101 MHz, DMSO-d_6) spectrum of hydrazide-hydrazone **23**.

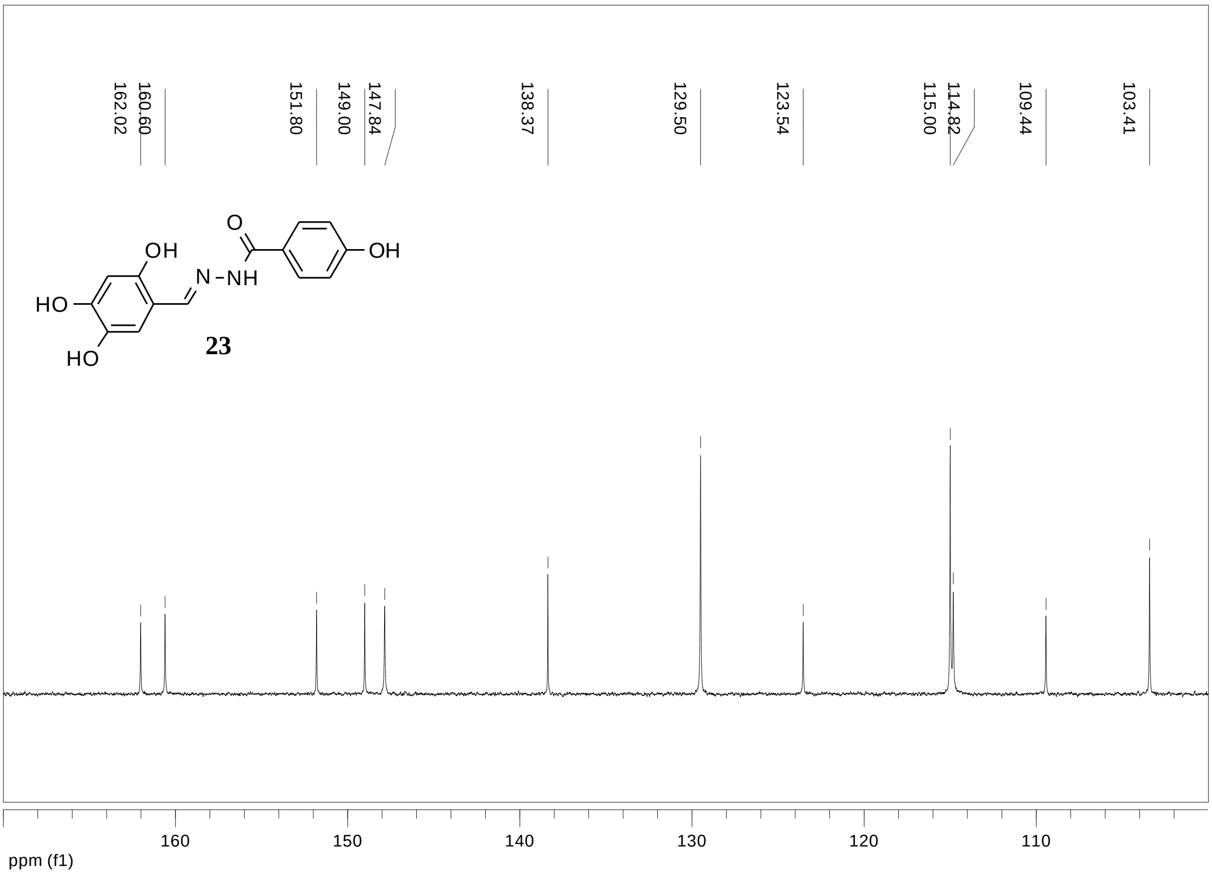

Figure S55. Expansion of ^{13}C-NMR (101 MHz, DMSO-d_6) spectrum of hydrazide-hydrazone **23**.

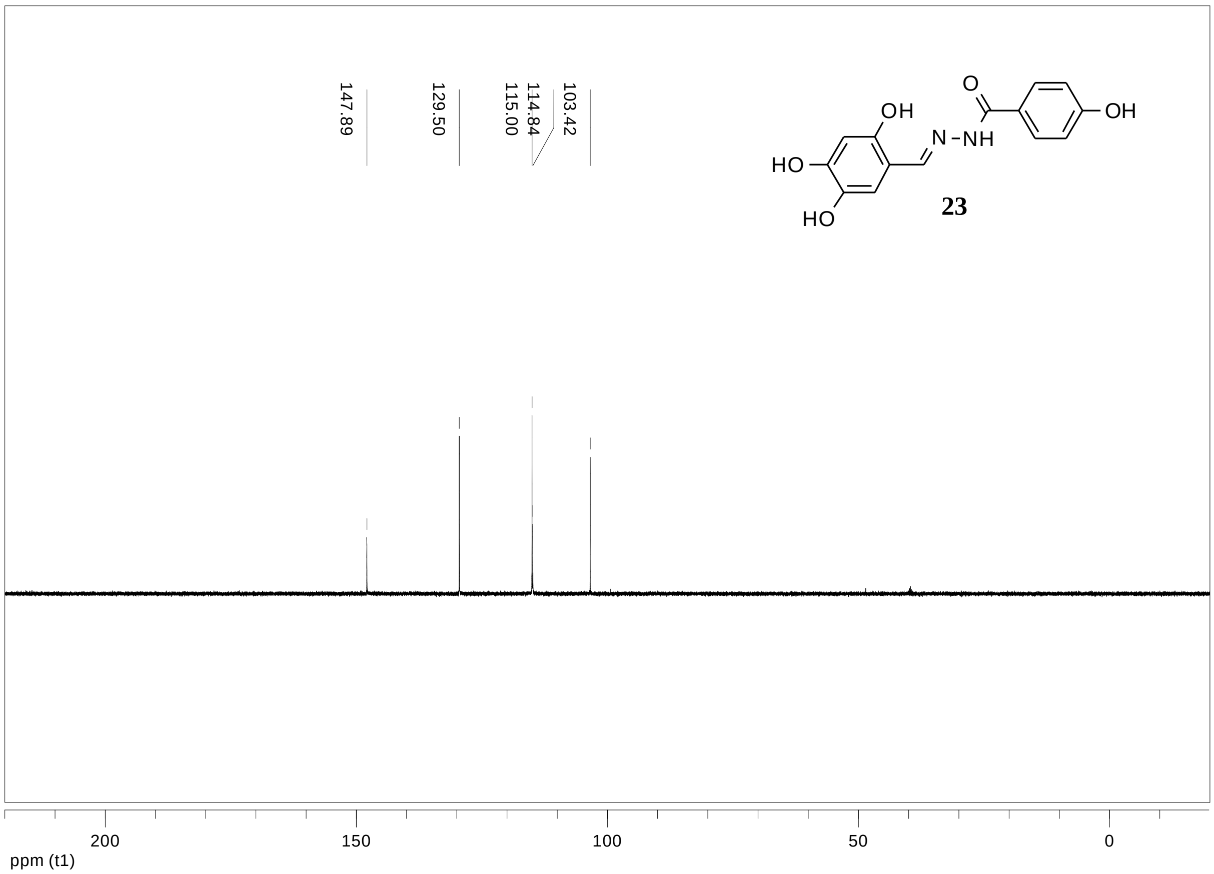

Figure S56. ^{13}C-NMR (101 MHz, DMSO-d_6) dept-135 experiment of hydrazide-hydrazone **23**.

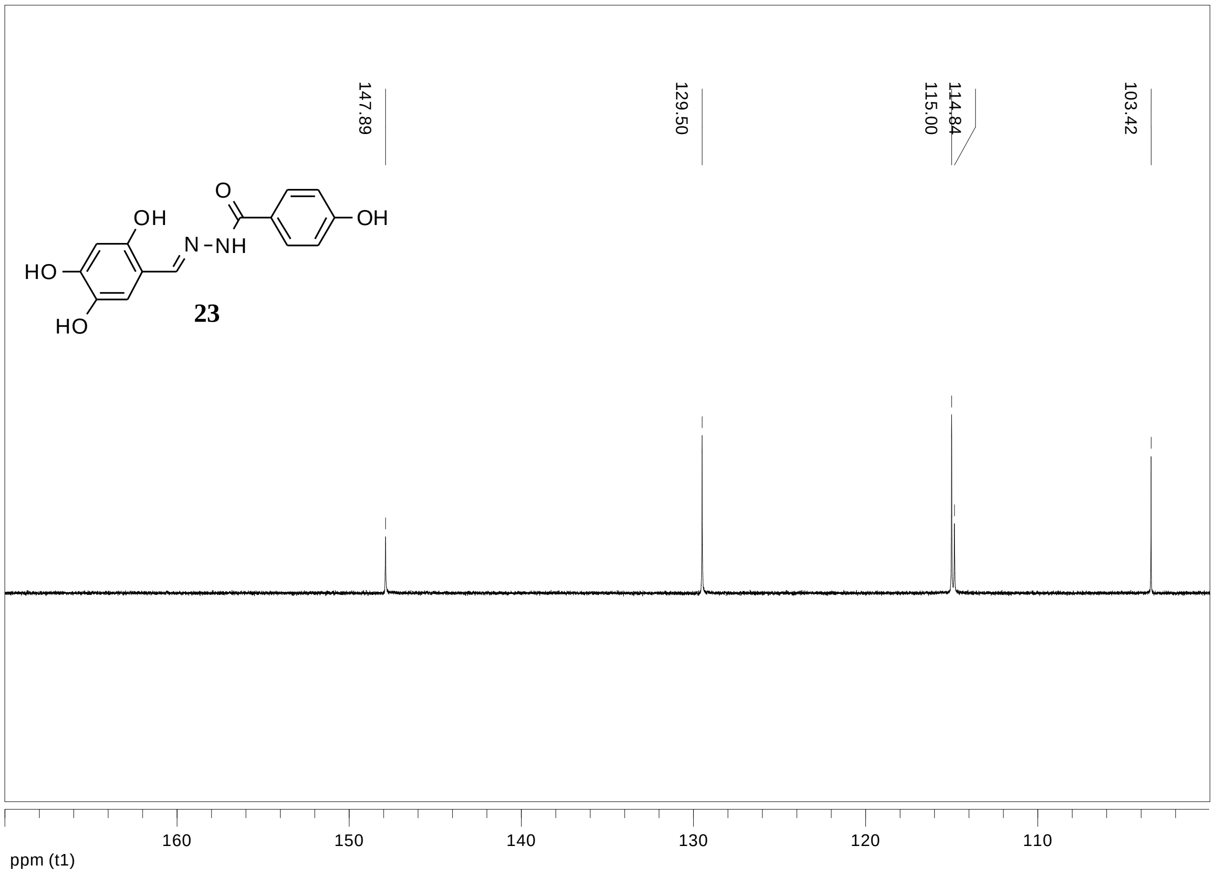

Figure S57. Expansion of ^{13}C-NMR (101 MHz, DMSO-d_6) dept-135 experiment of hydrazone **23**.

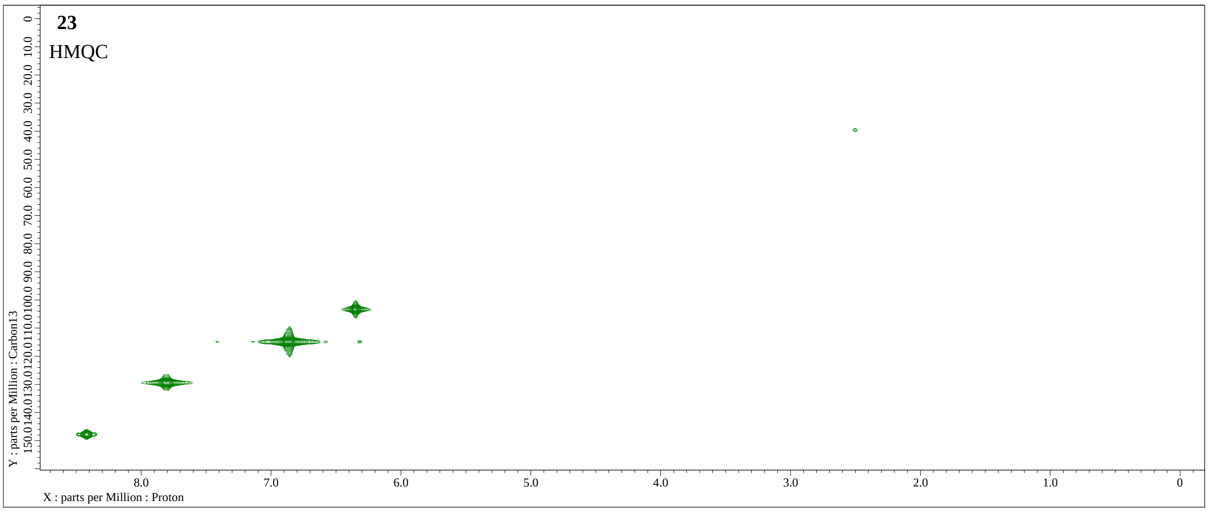

Figure S58. 2D-NMR (400 MHz,DMSO-d_6) HMQC experiment of 4-hydroxy-*N'*-[(*E*)-(2,4,5-trihydroxyphenyl)methylidene]benzohydrazide (**23**).

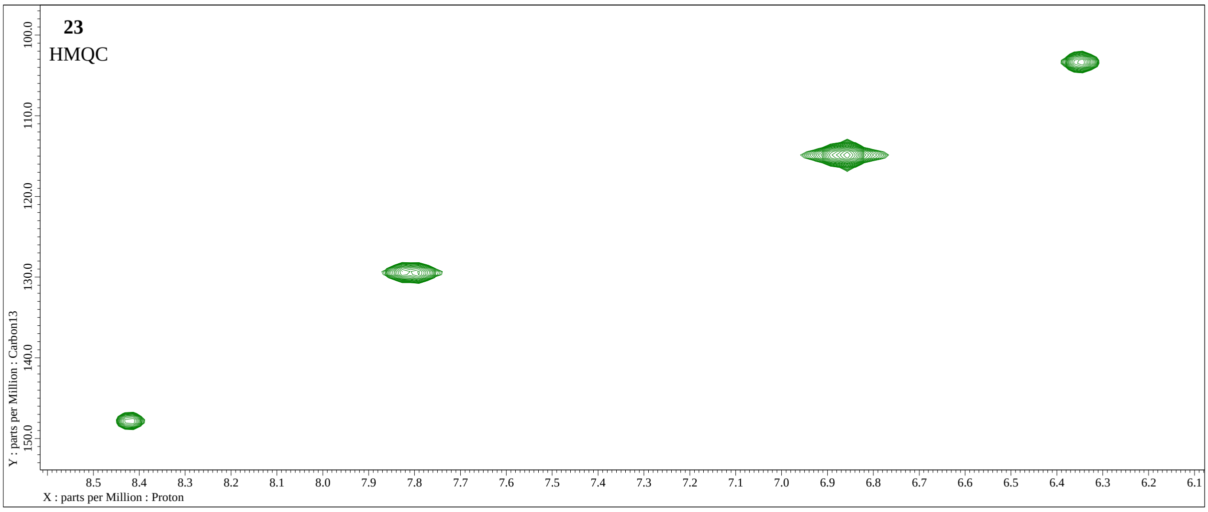

Figure S59. Expansion of 2D-NMR (400 MHz, DMSO-d_6) HMQC experiment of 4-hydroxy-*N'*-[(*E*)-(2,4,5-trihydroxyphenyl)methylidene]benzohydrazide (**23**).

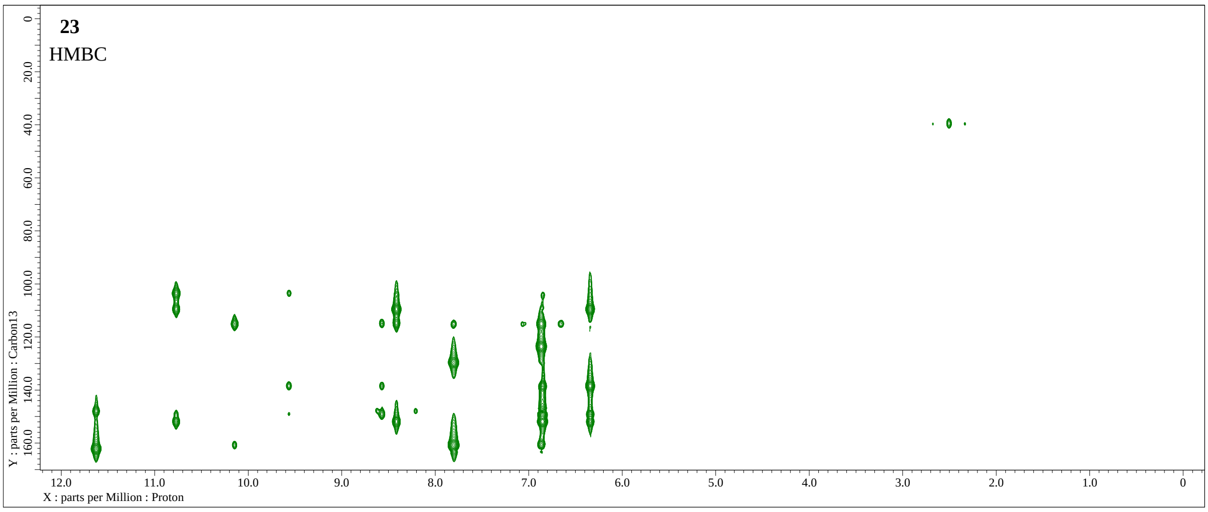

Figure S60. 2D-NMR (400 MHz, DMSO-d_6) HMBC experiment of 4-hydroxy-*N'*-[(*E*)-(2,4,5-trihydroxyphenyl)methylidene]benzohydrazide (**23**).

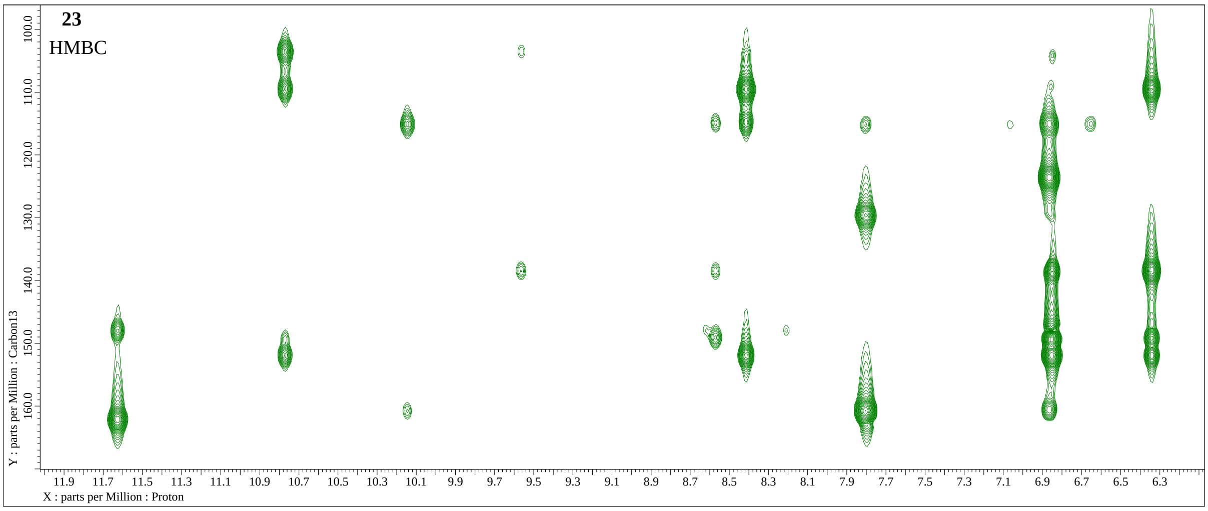

Figure S61. Expansion of 2D-NMR (400 MHz, DMSO-d_6) HMBC experiment of 4-hydroxy-*N'*-[(*E*)-(2,4,5-trihydroxyphenyl)methylidene]benzohydrazide (**23**).

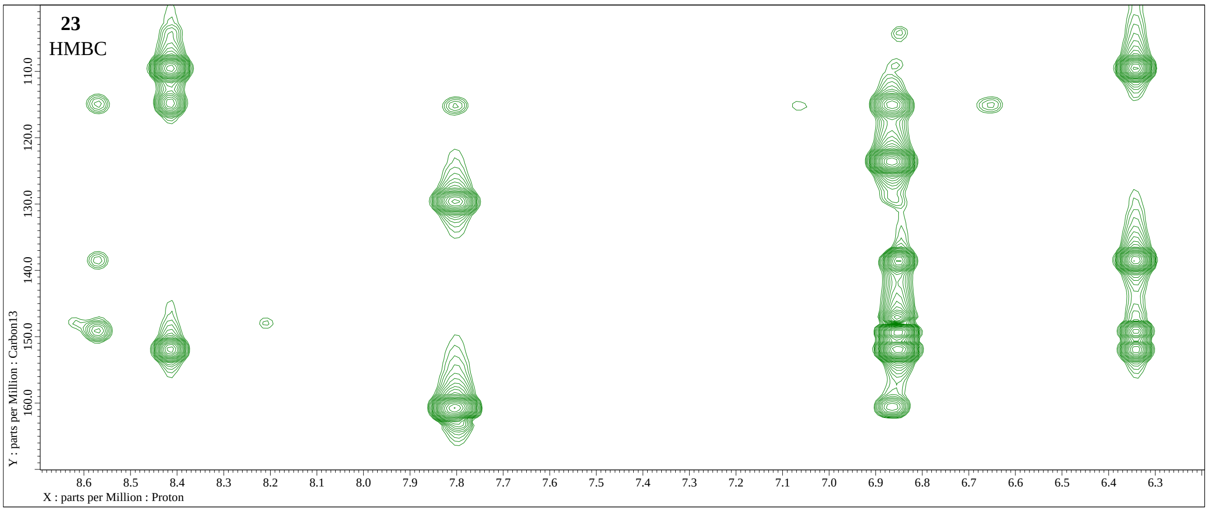

Figure S62. Expansion of 2D-NMR (400 MHz, DMSO-d_6) HMBC experiment of 4-hydroxy-*N'*-[(*E*)-(2,4,5-trihydroxyphenyl)methylidene]benzohydrazide (**23**).

4-Hydroxy-*N*'-[(*E*)-(2-hydroxy-3-hydroxymethyl-5-methylphenyl)methylidene]benzohydrazide (25) [1].

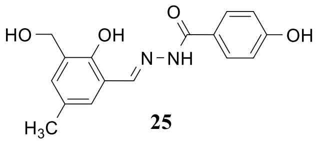

25

The general procedure starting from equimolar amounts of 2-hydroxy-3-hydroxymethyl-5-methylbenzaldehyde (**62**) [1], 4-hydroxybenzohydrazide (**36**), in methanol, in the presence of acetic acid additive was employed with 3 h reaction time to obtain the 4-hydroxy-*N*'-[(*E*)-(2-hydroxy-3-hydroxymethyl-5-methylphenyl)methylidene]benzohydrazide (**25**) with 80% yield, which melt at 228–229 °C (m.p. 229–230 °C [1]).

HRMS (TOF, MS, ESI): *m*/*z* for $C_{16}H_{16}N_2O_4 - H_2O + H^+$ calculated: 283.1077; found: 283.1072; *m*/*z* for $C_{16}H_{16}N_2O_4 + H^+$ calculated: 301.1183; found: 301.1178; *m*/*z* for $C_{16}H_{16}N_2O_4 - H_2O + Na^+$ calculated: 305.0897; found: 305.0970; *m*/*z* for $C_{16}H_{16}N_2O_4 + Na^+$ calculated: 323.1002; found: 323.0998.

The FT-IR, ^{1}H-NMR and ^{13}C-NMR spectra and HRMS analysis is content with literature value [1].

4-Hydroxy-*N*'-[(*E*)-(2-hydroxy-3-hydroxymethyl-5-methoxyphenyl)methylidene]benzohydrazide (26).

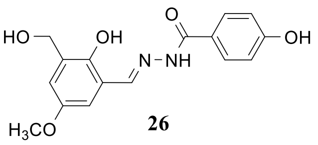

26

The general procedure starting from 2-hydroxy-3-hydroxymethyl-5-methoxy-benzaldehyde (**63**) (128 mg, 0.70 mmol) [7], 4-hydroxybenzohydrazide (**36**) (106 mg, 0.70 mmol), CH_3OH (3.5 mL), and AcOH (70 µL) was employed with a 5 hours reaction time, and left to crystallization in an open vessel to obtain the 4-hydroxy-*N*'-[(*E*)-(2-hydroxy-3-hydroxymethyl-5-methoxyphenyl)methylidene]benzohydrazide (**26**). A pale yellow powder; 215 mg, 0.68 mmol, 97% yield; m.p. 219–220 °C (from CH_3OH); selected FT-IR (ATR): ν_{max} 3540 (O-H), 3223 (br, N-H, O-H), 3045 (C_{Ar}-H), 2987 (C-H), 2834 (C-H), 1626 (C=O), 1602 (N-H), 1582 (CH=N), 1556 (C_{Ar}-H), 1509, 1457 (C_{Ar}-H), 1305, 1254 (C_{Ar}-O), 1176, 1144 (CH_2-O), 1060, 1035 (CH_3-O), 955 (N-N), 890, 847, 763, 660, 621, 526 cm^{-1}; ^{1}H-NMR (400 MHz, DMSO-d_6): δ 12.02 (s, 1H, NH), 11.53 (s, 1H, Ar-2-OH), 10.21 (s, 1H, 4-OH), 8.51 (s, 1H, CH=N), 7.83 (d, ^3J = 8.6 Hz, 2H, H-2,6), 7.04 (d, ^4J = 2.9 Hz, 1H, ArH-4), 6.89 (d, ^4J = 2.9 Hz, 1H, ArH-6), 6.89 (d, ^3J = 8.6 Hz, 2H, H-3,5), 5.15 (t, ^3J = 5.5 Hz, 1H, CH_2<u>OH</u>), 4.55 (d, ^3J = 5.5 Hz, 2H, CH_2), 3.74 (s, 3H, OCH_3) ppm; ^{13}C-NMR (101 MHz, DMSO-d_6): δ 162.41 (C=O), 160.97 (C-4), 151.83 (ArC-5), 148.66 (CH=N), 148.48 (ArC-2), 131.06 (ArC-3), 129.77 (C-2,6), 122.98 (C-1), 117.32 (ArC-1), 115.48 (ArC-4), 115.13 (C-3,5), 112.09 (ArC-6), 57.60 (CH_2), 55.46 (OCH_3) ppm; HRMS (TOF, MS, ESI): *m/z* for $C_{16}H_{16}N_2O_5 - H_2O + H^+$ calculated: 299.1026; found: 299.1037; *m/z* for $C_{16}H_{16}N_2O_5 + H^+$ calculated: 317.1132; found: 317.1141; for $C_{16}H_{16}N_2O_5 - H_2O + Na^+$ calculated: 321.0846; found: 321.0847; *m/z* for $C_{16}H_{16}N_2O_5 + Na^+$ calculated: 339.0951; found: 339.0951.

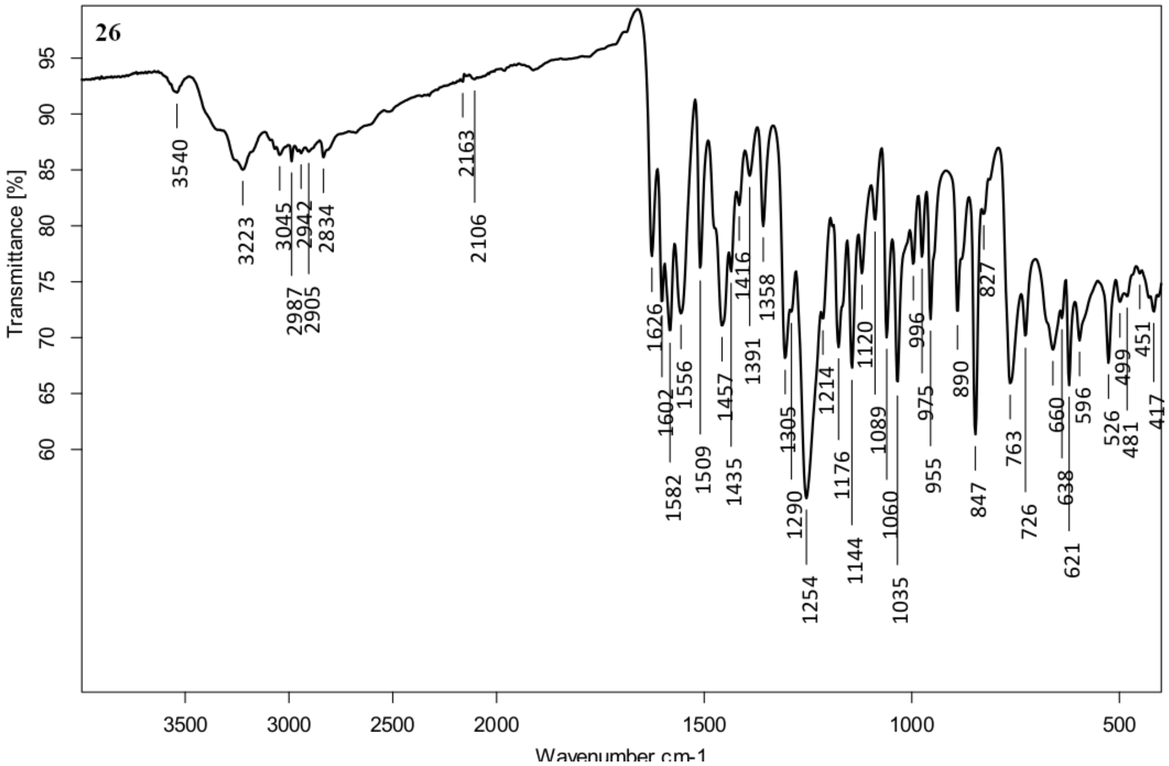

Figure S63. FT-IR (ATR) (4000–400 cm^{-1}) spectrum of 4-hydroxy-*N*'-[(*E*)-(2-hydroxy-3-hydroxymethyl-5-methoxyphenyl)methylidene]benzohydrazide (**26**).

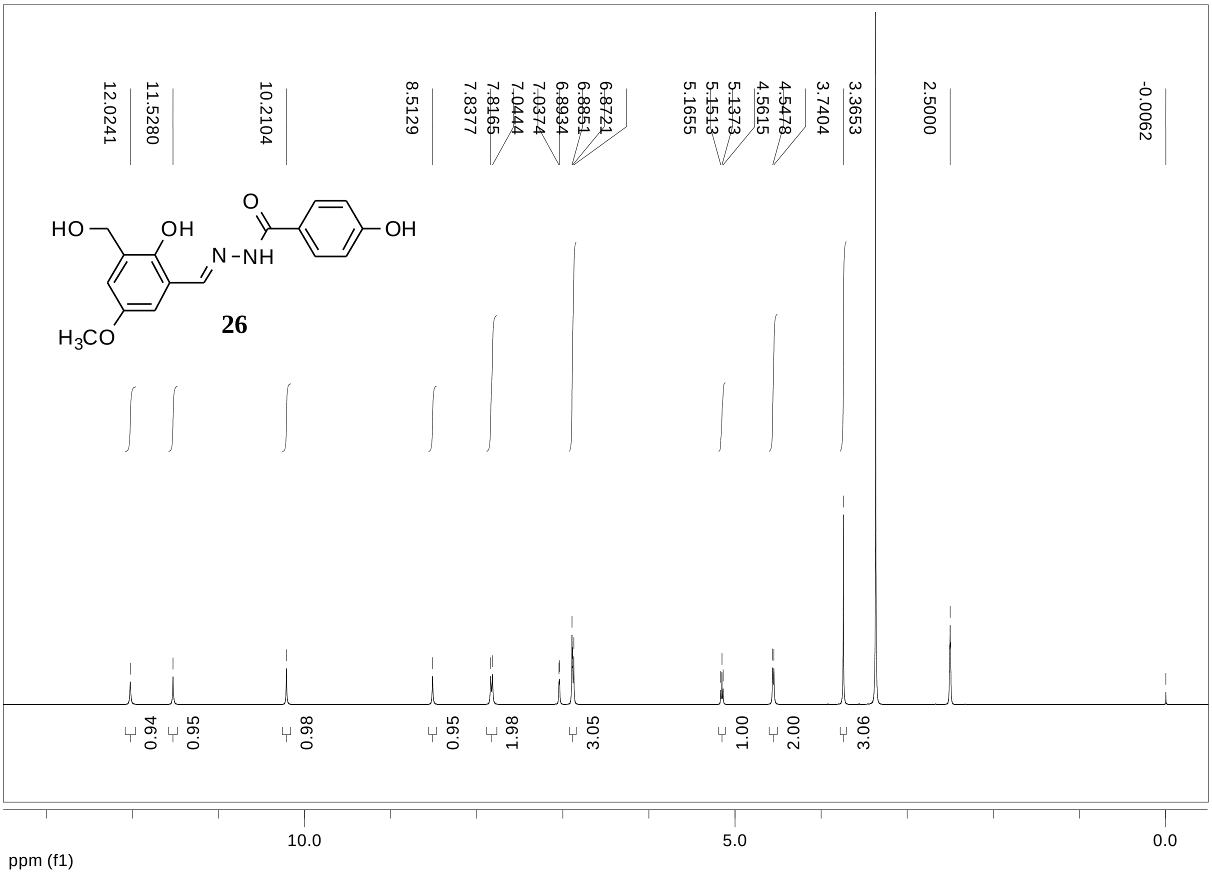

Figure S64. ^{1}H-NMR (400 MHz, DMSO-d_6) spectrum of hydrazide-hydrazone **26**.

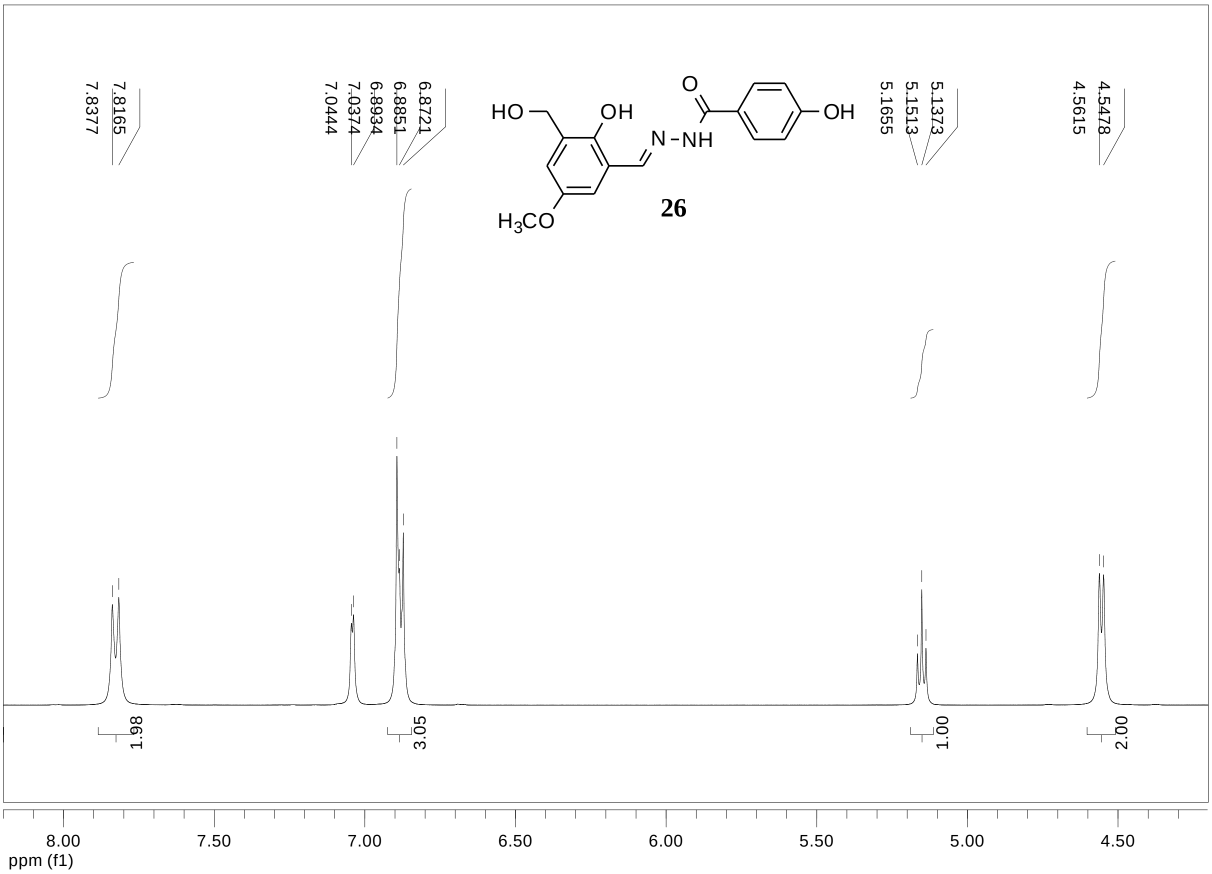

Figure S65. Expansion of ^{1}H-NMR (400 MHz, DMSO-d_6) spectrum of hydrazide-hydrazone **26**.

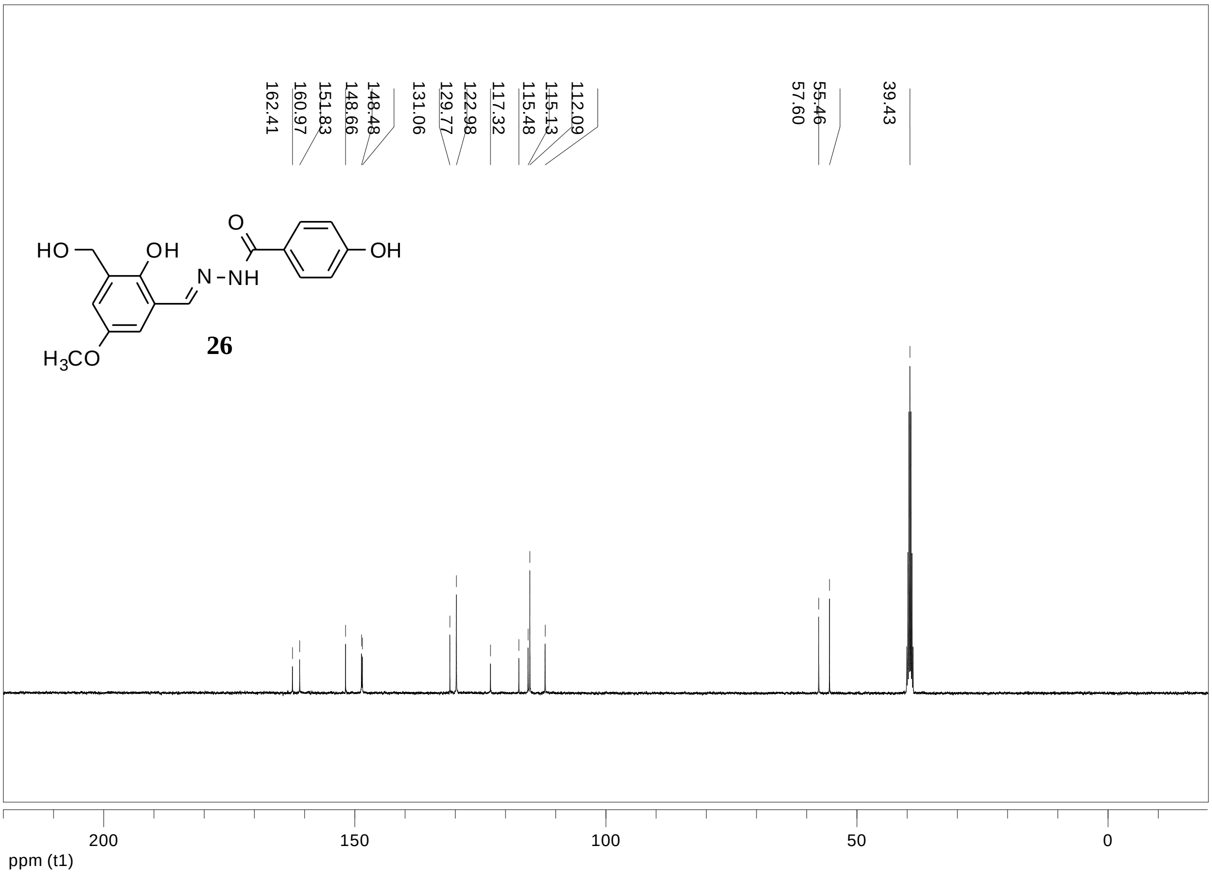

Figure S66. ^{13}C-NMR (101 MHz, DMSO-d_6) spectrum of hydrazide-hydrazone **26**.

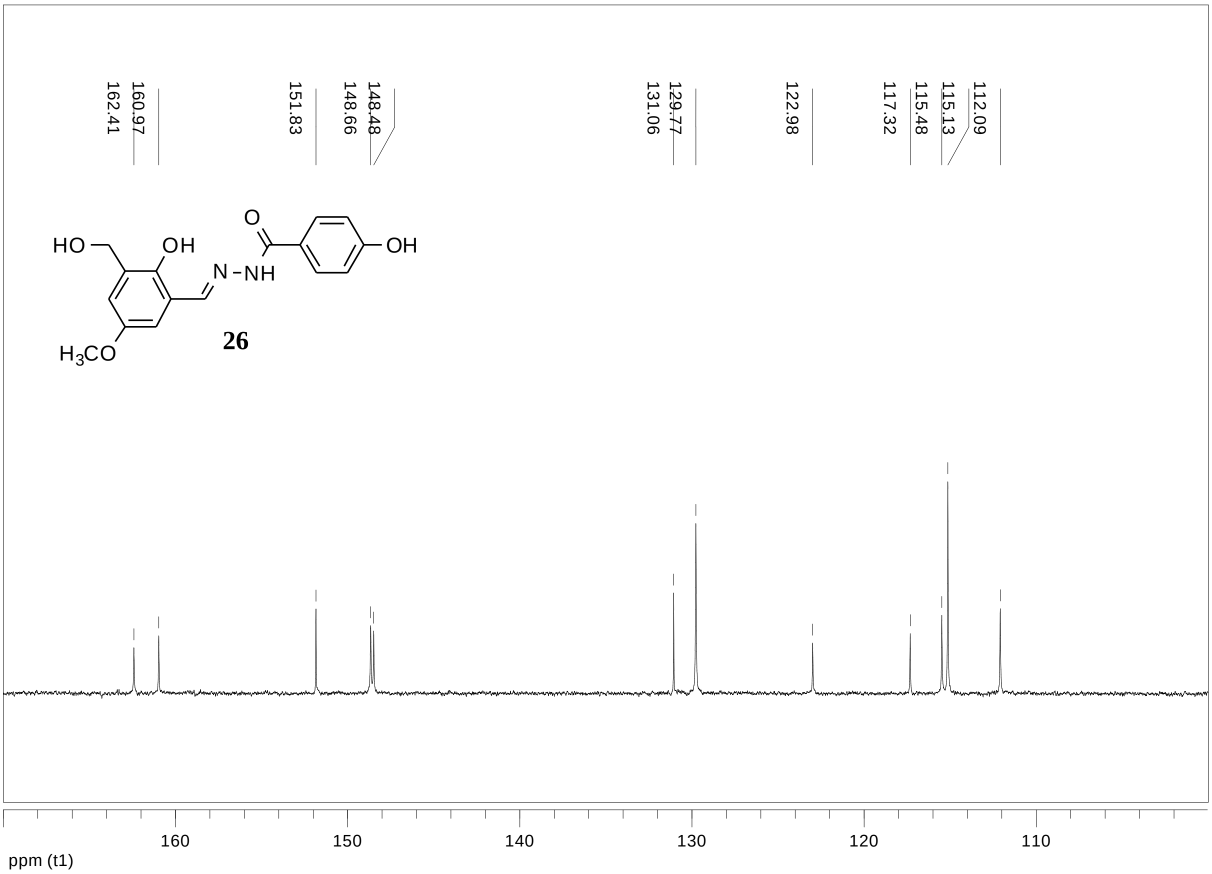

Figure S67. Expansion of ^{13}C-NMR (101 MHz, DMSO-d_6) spectrum of hydrazide-hydrazone **26**.

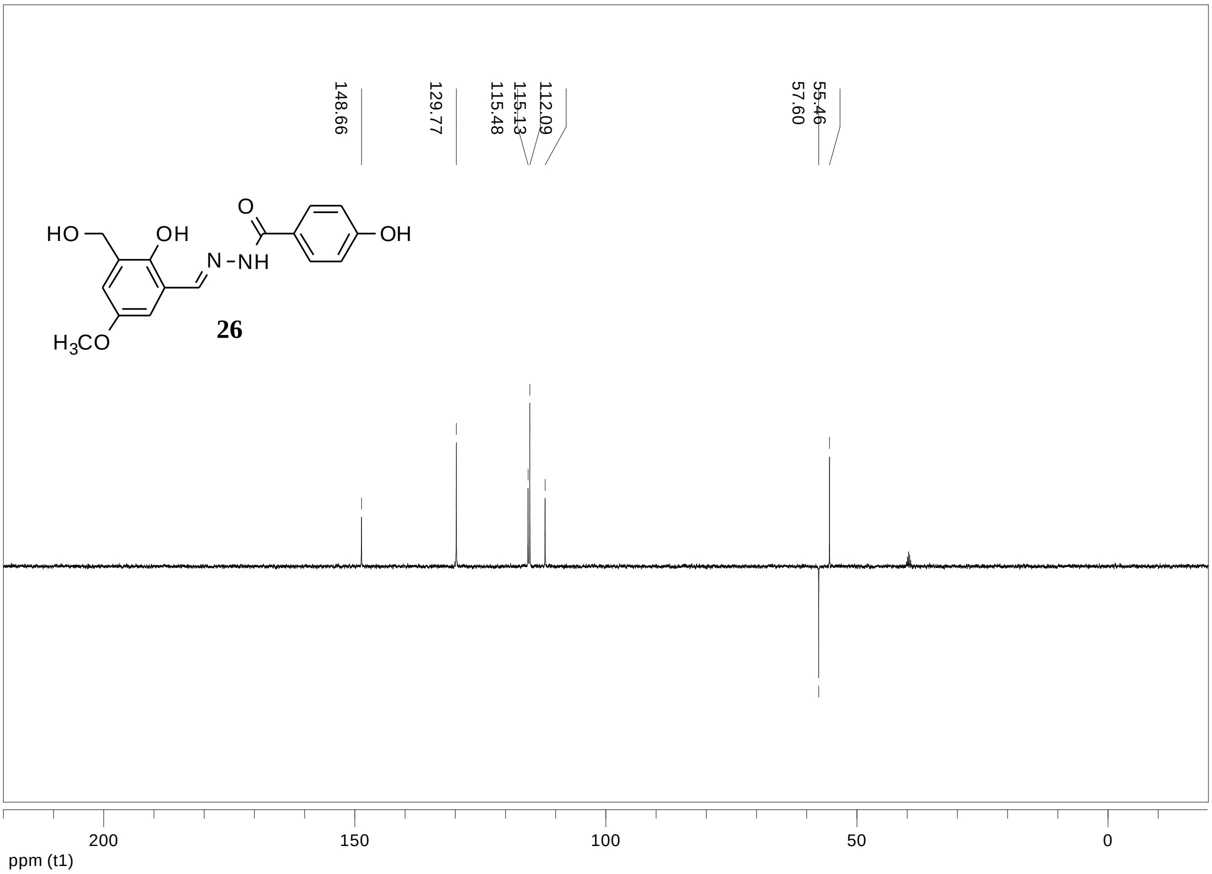

Figure S68. ^{13}C-NMR (101 MHz, DMSO-d_6) dept-135 experiment of hydrazide-hydrazone **26**.

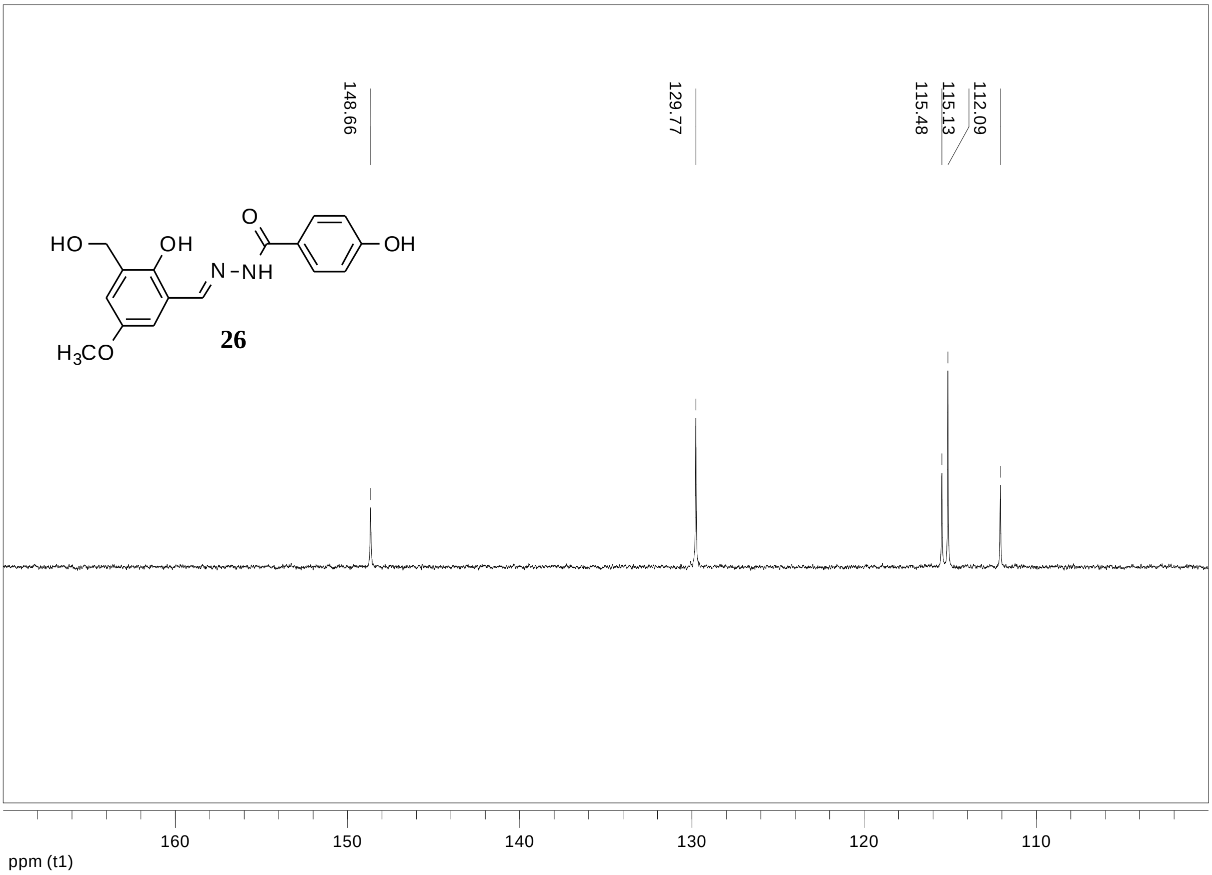

Figure S69. Expansion of ^{13}C-NMR (101 MHz, DMSO-d_6) dept-135 experiment of hydrazone **26**.

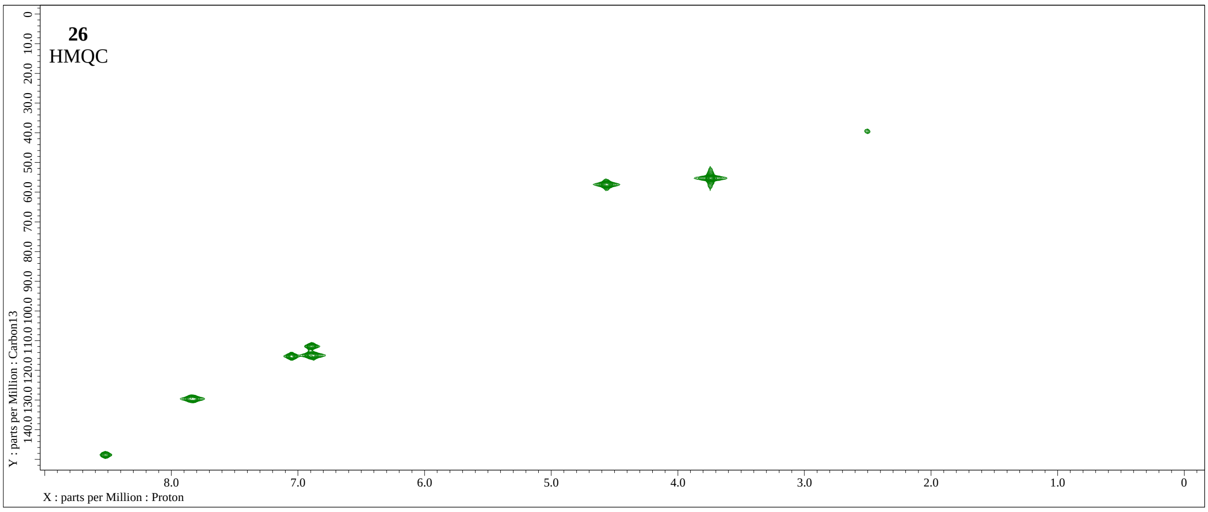

Figure S70. 2D-NMR (400 MHz, DMSO-d_6) HMQC experiment of 4-hydroxy-*N*'-[(*E*)-(2-hydroxy-3-hydroxymethyl-5-methoxyphenyl)methylidene]benzohydrazide (**26**).

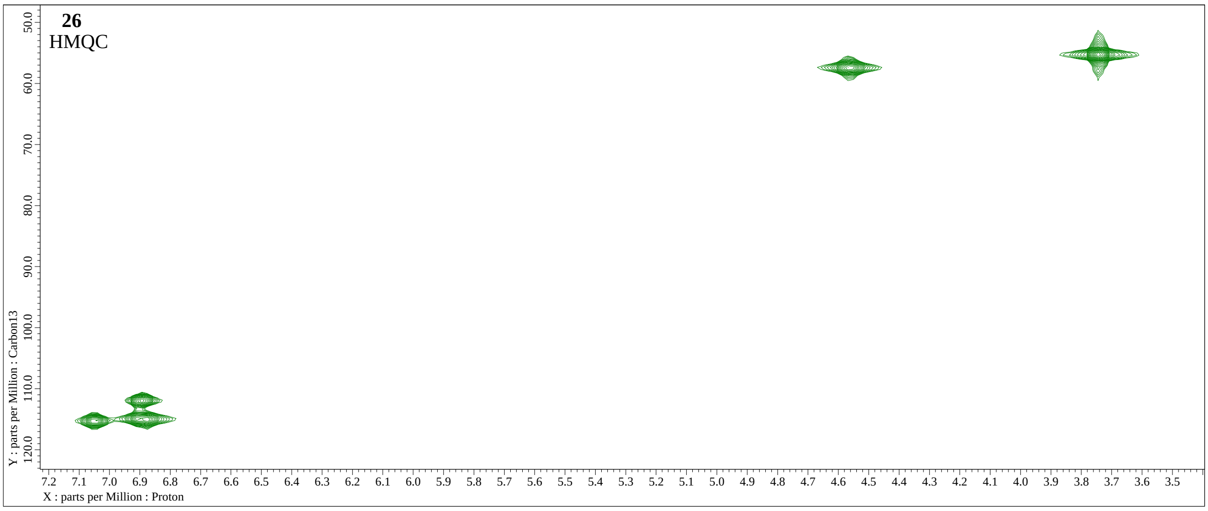

Figure S71. Expansion of 2D-NMR (400 MHz, DMSO-d_6) HMQC experiment of 4-hydroxy-*N*'-[(*E*)-(2-hydroxy-3-hydroxymethyl-5-methoxyphenyl)methylidene]benzohydrazide (**26**).

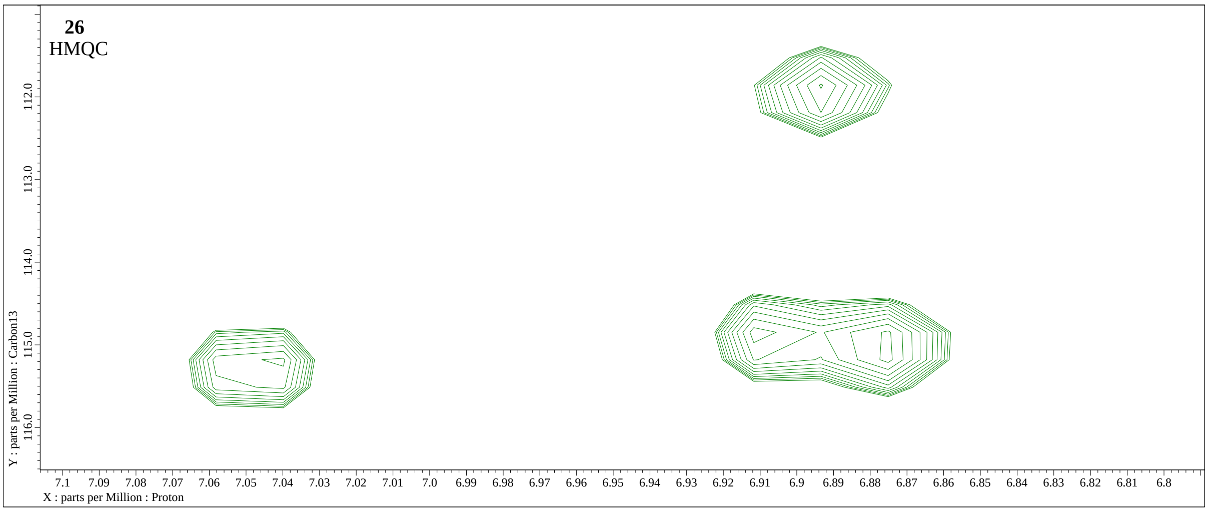

Figure S72. Expansion of 2D-NMR (400 MHz, DMSO-d_6) HMQC experiment of 4-hydroxy-*N*'-[(*E*)-(2-hydroxy-3-hydroxymethyl-5-methoxyphenyl)methylidene]benzohydrazide (**26**).

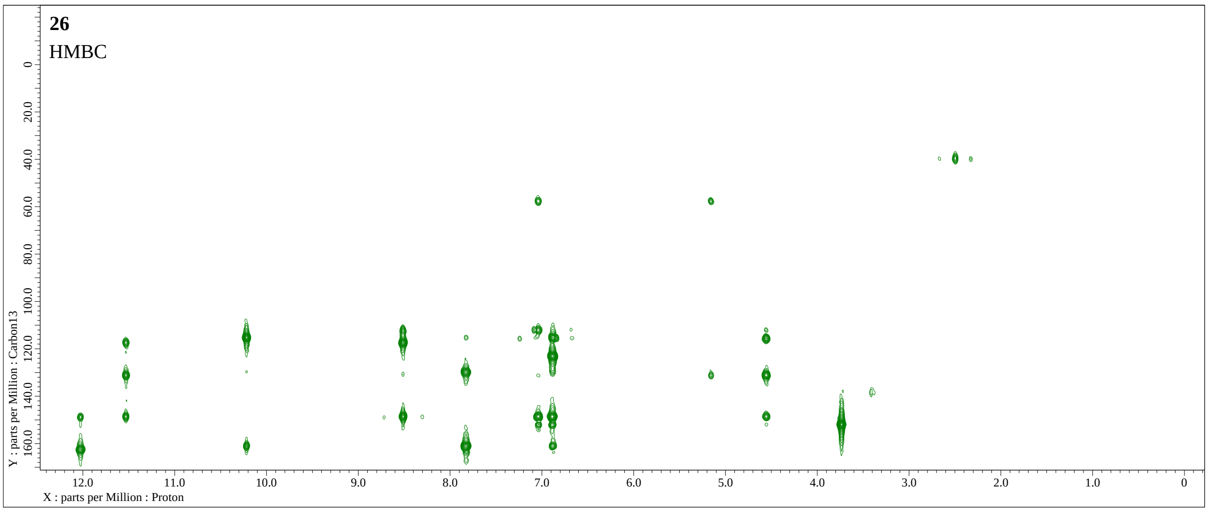

Figure S73. 2D-NMR (400 MHz, DMSO-d_6) HMBC experiment of 4-hydroxy-*N*'-[(*E*)-(2-hydroxy-3-hydroxymethyl-5-methoxyphenyl)methylidene]benzohydrazide (**26**).

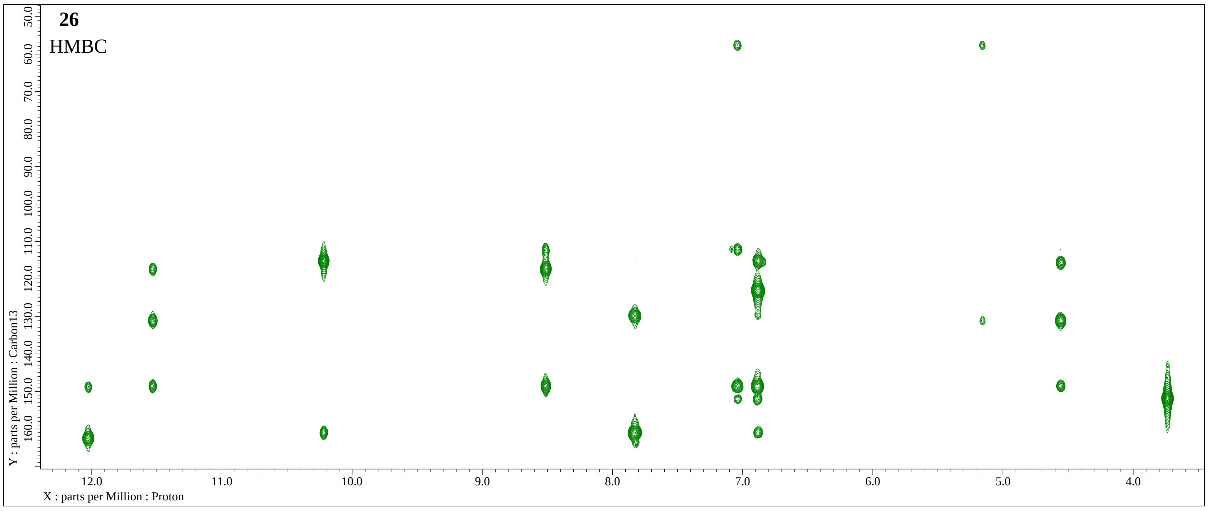

Figure S74. Expansion of 2D-NMR (400 MHz, DMSO-d_6) HMBC experiment of 4-hydroxy-*N*'-[(*E*)-(2-hydroxy-3-hydroxymethyl-5-methoxyphenyl)methylidene]benzohydrazide (**26**).

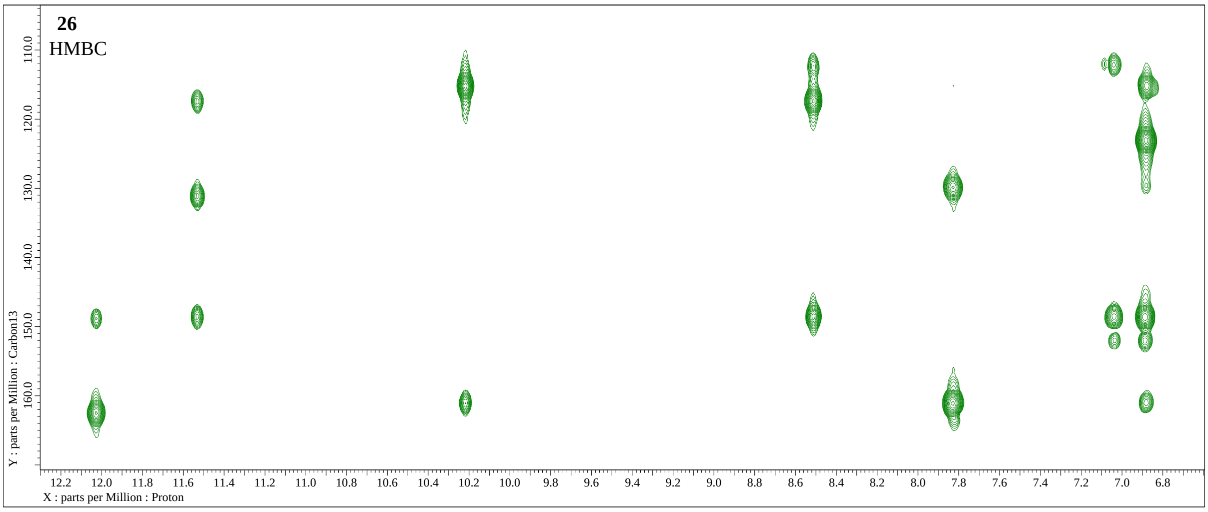

Figure S75. Expansion of 2D-NMR (400 MHz, DMSO-d_6) HMBC experiment of 4-hydroxy-*N*'-[(*E*)-(2-hydroxy-3-hydroxymethyl-5-methoxyphenyl)methylidene]benzohydrazide (**26**).

4-Hydroxy-*N*'-[(*E*)-(2-hydroxy-3-isopropyl-6-methylphenyl)methylidene]benzohydrazide (28) [1]**.**

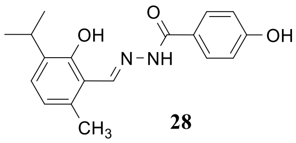

28

The general procedure starting from 2-hydroxy-3-isopropyl-6-methylbenzaldehyde (**65**) (356 mg, 2.0 mmol) [1], 4-hydroxybenzohydrazide (**36**) (304 mg, 2.0 mmol), CH_3OH (6.0 mL), and AcOH (0.10 mL) was employed with a 6 hours reaction time to obtain a pale white fine crystalline of 4-hydroxy-*N*'-[(*E*)-(2-hydroxy-3-isopropyl-6-methylphenyl)methylidene]benzohydrazide (**28**) (593 mg, 1.9 mmol) with 95% yield, which melt at 258–260 °C (from CH_3OH) (m.p. 258.5–260.5 °C [1]); selected FT-IR (ATR): ν_{max} 3416 (O-H), 3200 (br, N-H, O-H), 3019 (C_{Ar}-H), 2958 (C-H), 1610 (C=O, CH=N), 1532 (C_{Ar}-H), 1505, 1432 (C_{Ar}-H), 1332, 1261 (C_{Ar}-O), 1152, 1107, 1056, 993 (N-N), 959, 849, 759, 715, 612 (br), 519 cm^{-1}; HRMS (TOF, MS, ESI): *m/z* for $C_{18}H_{20}N_2O_3 + H^+$ calculated: 313.1547; found: 313.1561. The ^{1}H-NMR and ^{13}C-NMR are consistent with literature values [1].

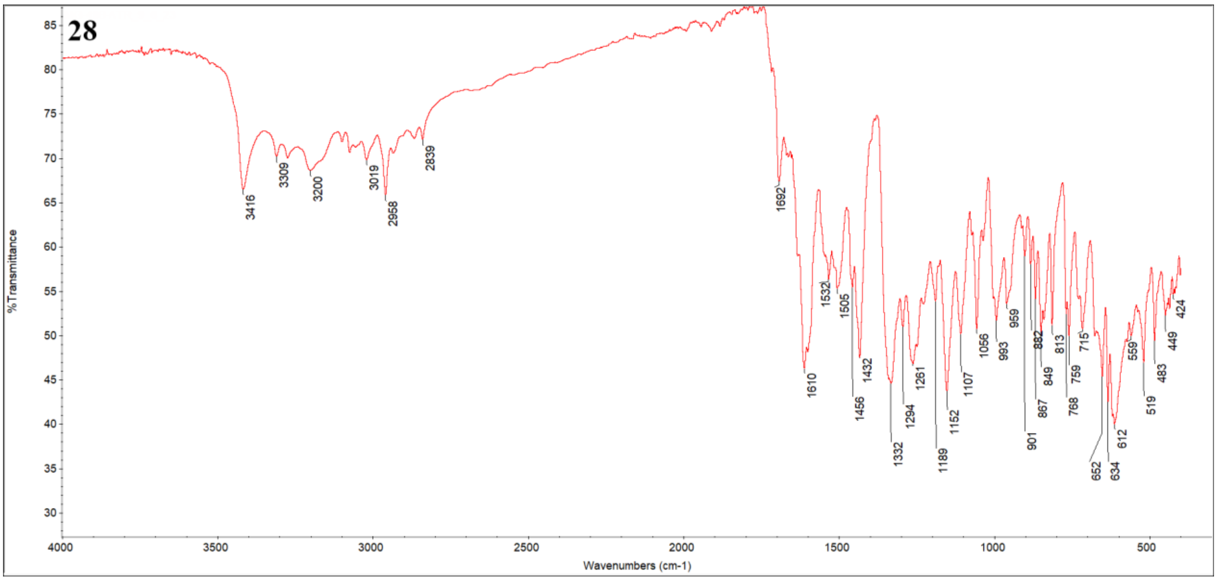

Figure S76. FT-IR (ATR) (4000–400 cm^{-1}) spectrum of thymol derivative – 4-hydroxy-*N*'-[(*E*)-(2-hydroxy-6-methyl-3-isopropylphenyl)methylidene]benzohydrazide (**28**).

***N'*,*N''*-[(2,4,6-trihydroxy-1,3-phenylene)di-(*E*)-methanylylidene]bis(4-hydroxybenzohydrazide) (32)** [2].

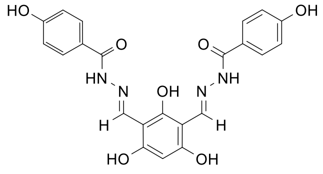

32

The hydrazide-hydrazone **32** was prepared with modified literature procedure [2]. The general procedure starting from 1,3-diformyl-2,4,6-trihydroxybenzene (**68**) (0.18g, 1.0 mmol) [8], 4-hydroxybenzohydrazide (**36**) (304 mg, 2.0 mmol), CH_3OH (10 mL), and AcOH (0.10 mL) was employed with a 20 hours reaction time, with formation carmine red mixture to obtain the *N*',*N''*-[(2,4,6-trihydroxy-1,3-phenylene)di-(*E*)-methanylylidene]bis(4-hydroxybenzohydrazide) (**32**). A brown red powder; 428 mg, 0.95 mmol, 95% yield; m.p. 223–226 °C (from CH_3OH) with decomposition (m.p. 223–226 °C with decomposition [2]); selected FT-IR (ATR): ν_{max} 3173 (br, OH, NH), 3074 (C_{Ar}-H), 3024 (C_{Ar}-H), 1604 (br, C=O, C=N), 1502, 1443, 1325, 1238 (br, C-O), 1171, 1056, 843, 756, 611, 515, 473 cm^{-1}; ^{1}H-NMR (400 MHz, DMSO-d_6): δ 13.33 (s, 1H, ArC-2-OH), 11.87 (s, 4H, ArC-4,6-OH, 2 × CONH), 10.17 (s, 2H, C-4,4'-OH), 8.84 (s, 2H, CH=N), 7.82 (d, ^3J = 8.7 Hz, 4H, H-2,2',6,6'), 6.88 (d, ^3J = 8.7 Hz, 4H, H-3,3',5,5'), 5.99 (s, 1H, ArH-5) ppm; ^{13}C-NMR (101 MHz, DMSO-d_6): δ 162.03 (2 × C=O), 161.25 (2 × C – ArC-4,6), 160.86 (2 × C – C-4,4'), 159.89 (C – ArC-2), 145.01 (2 × CH=N), 129.63 (4 × CH – C-2,2',6,6'), 123.12 (2 × C – C-1,1'), 115.16 (4 × CH – C-3,3',5,5'), 99.21 (2 × C – ArC-1,3), 94.46 (CH) ppm; HRMS (TOF, MS, ESI) *m/z* for $C_{22}H_{18}N_4O_7 + H^+$ calculated: 451.1248; found: 451.1261.

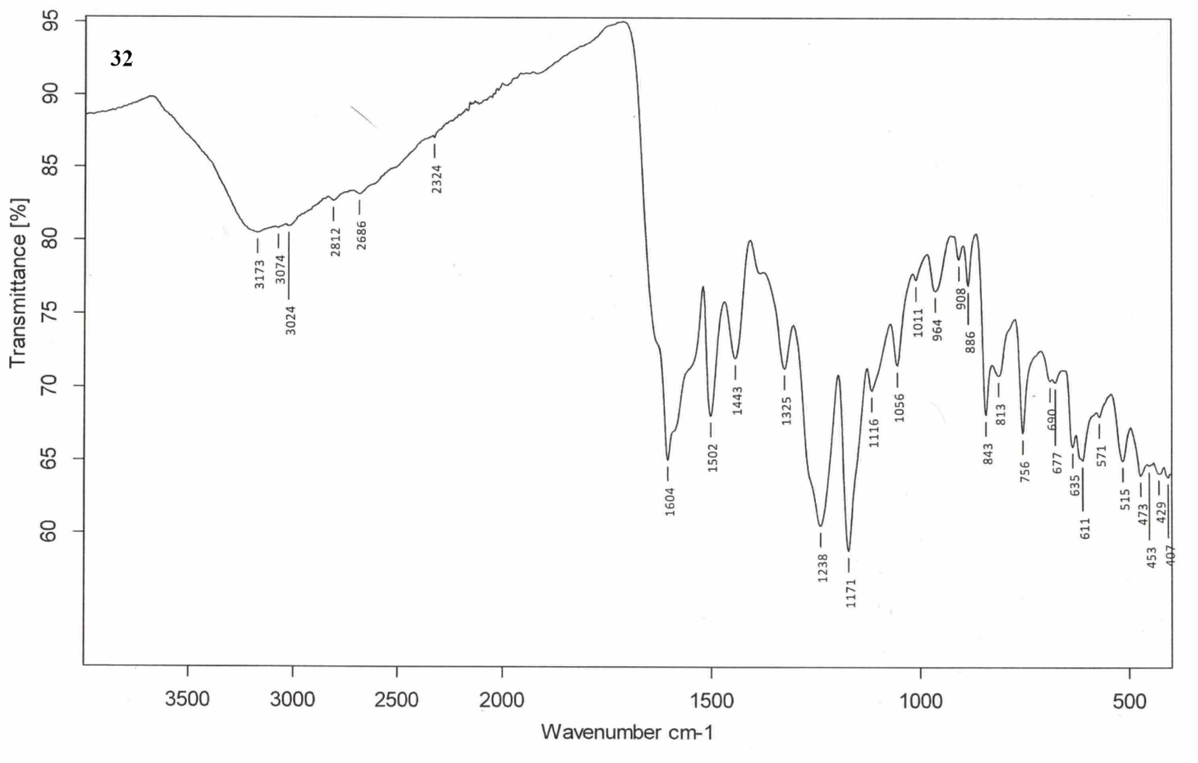

Figure S77. FT-IR (ATR) (4000–400 cm^{-1}) spectrum of *N*',*N''*-[(2,4,6-trihydroxy-1,3-phenylene)di-(*E*)-methanylylidene]bis(4-hydroxybenzohydrazide) (**32**).

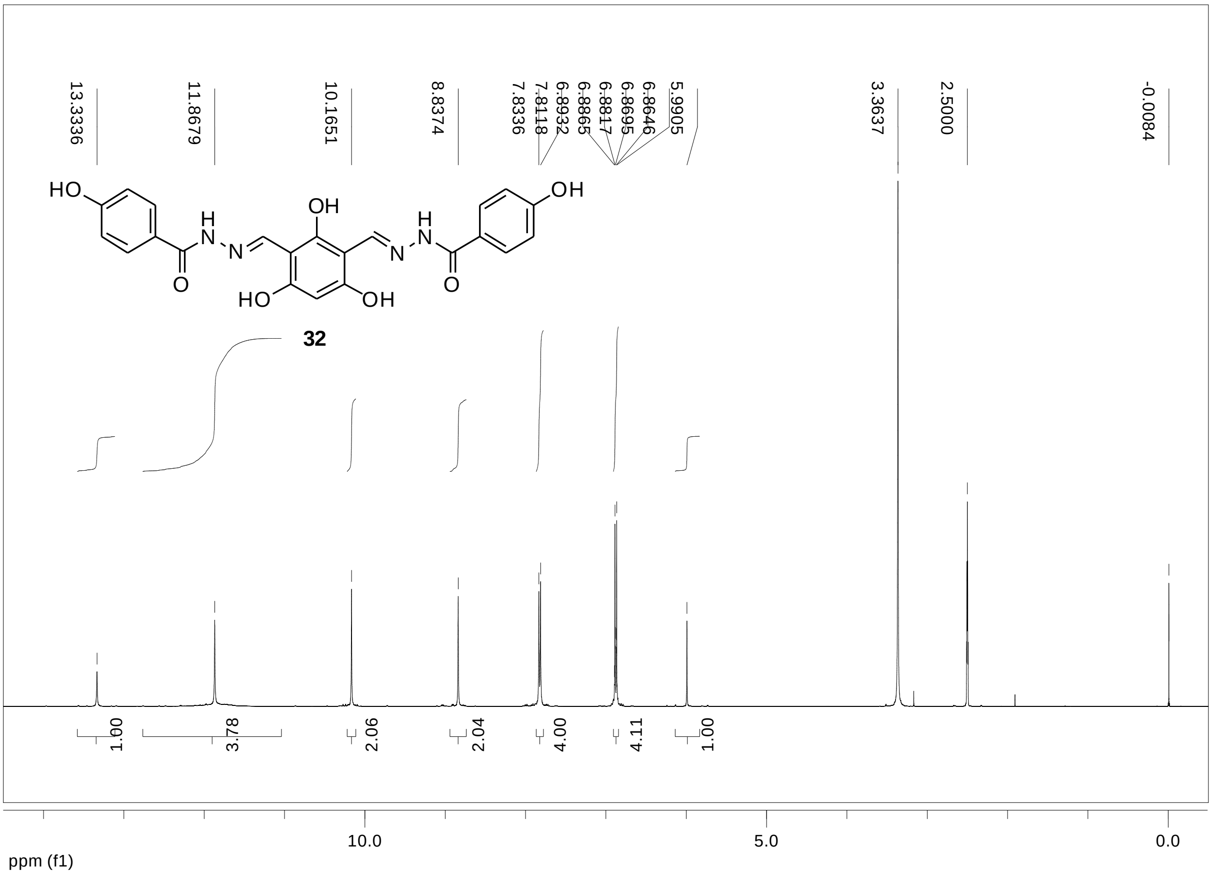

Figure S78.^{1}H-NMR (400 MHz, DMSO-d_6) spectrum of hydrazide-hydrazone **32**.

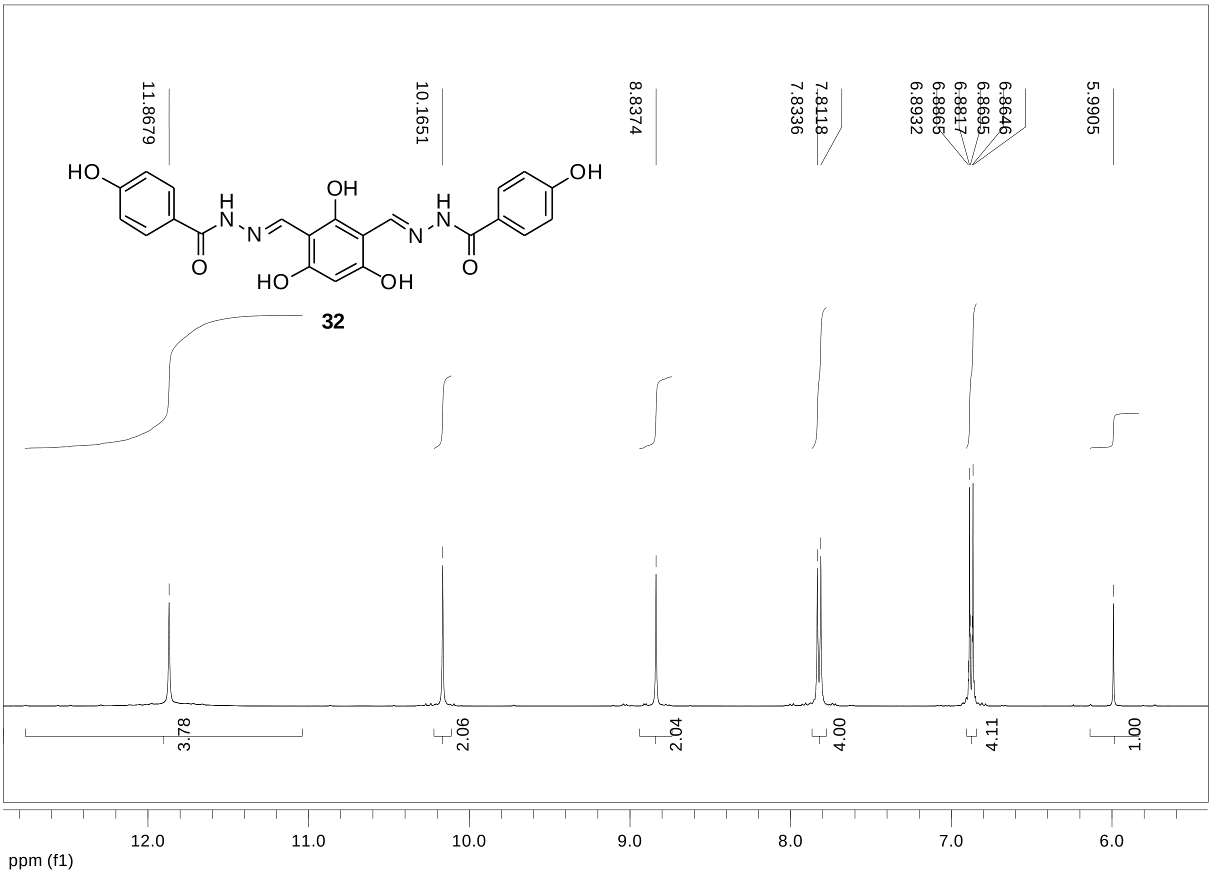

Figure S79. Expansion of ^{1}H-NMR (400 MHz, DMSO-d_6) spectrum of hydrazide-hydrazone **32**.

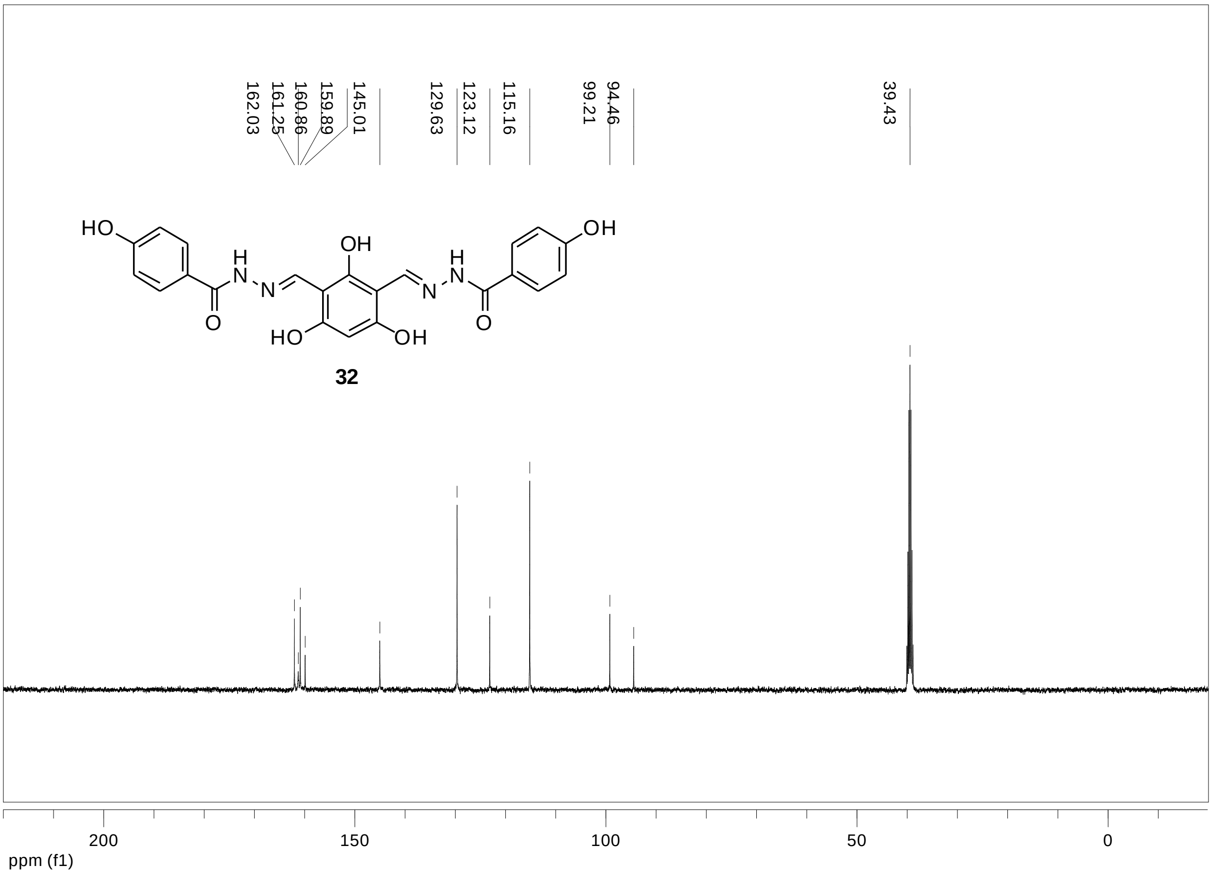

Figure S80. ^{13}C-NMR (101 MHz, DMSO-d_6) spectrum of hydrazide-hydrazone **32**.

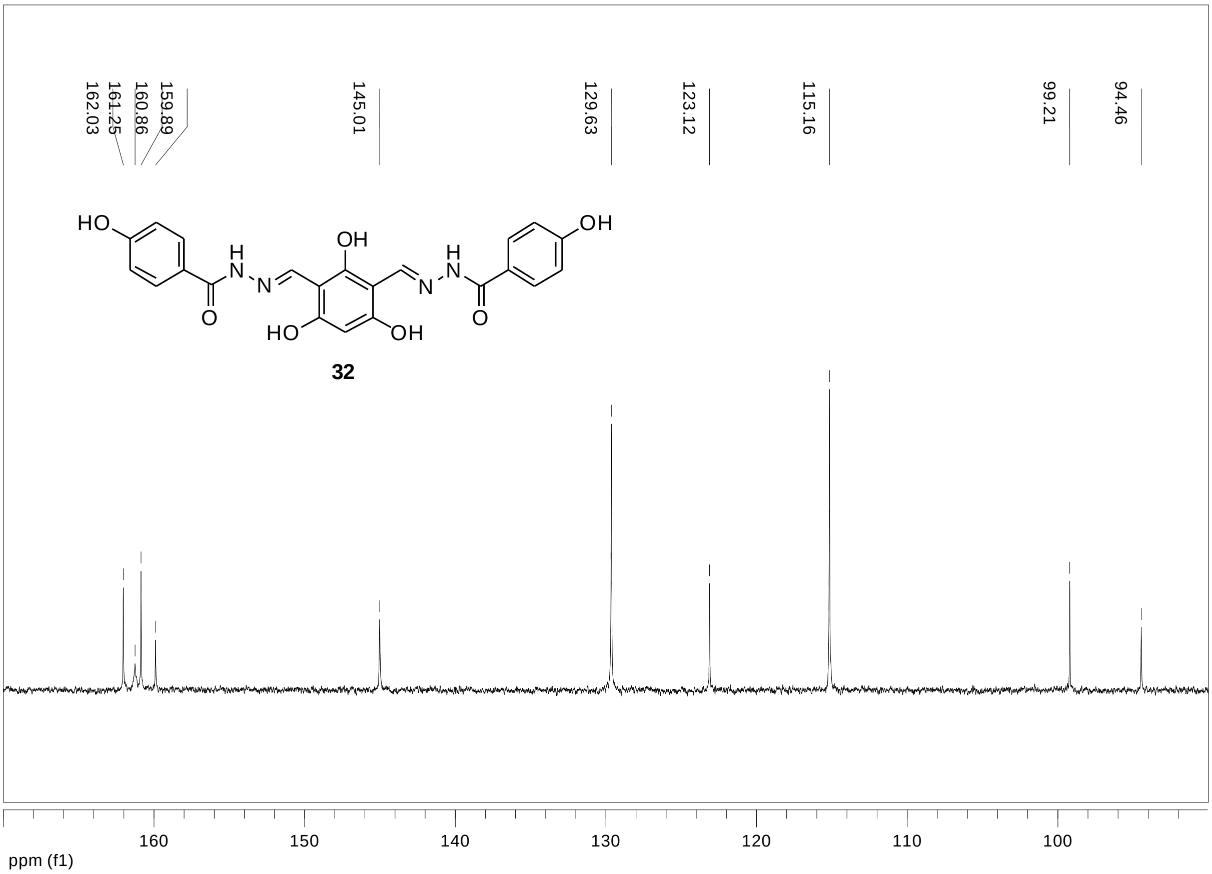

Figure S81. Expansion of ^{13}C-NMR (101 MHz, DMSO-d_6) spectrum of hydrazide-hydrazone **32**.

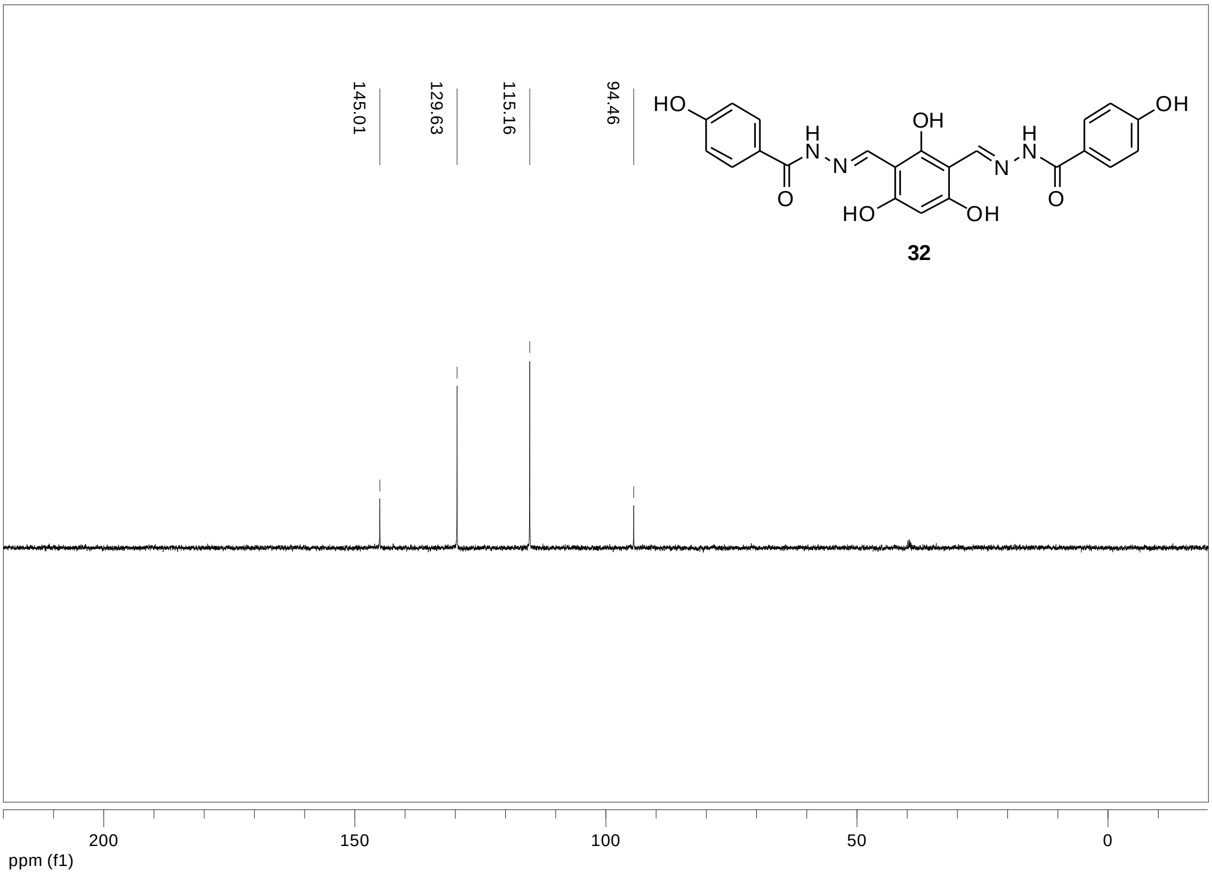

Figure S82. ^{13}C-NMR (101 MHz, DMSO-d_6) dept-135 experiment of hydrazide-hydrazone **32**.

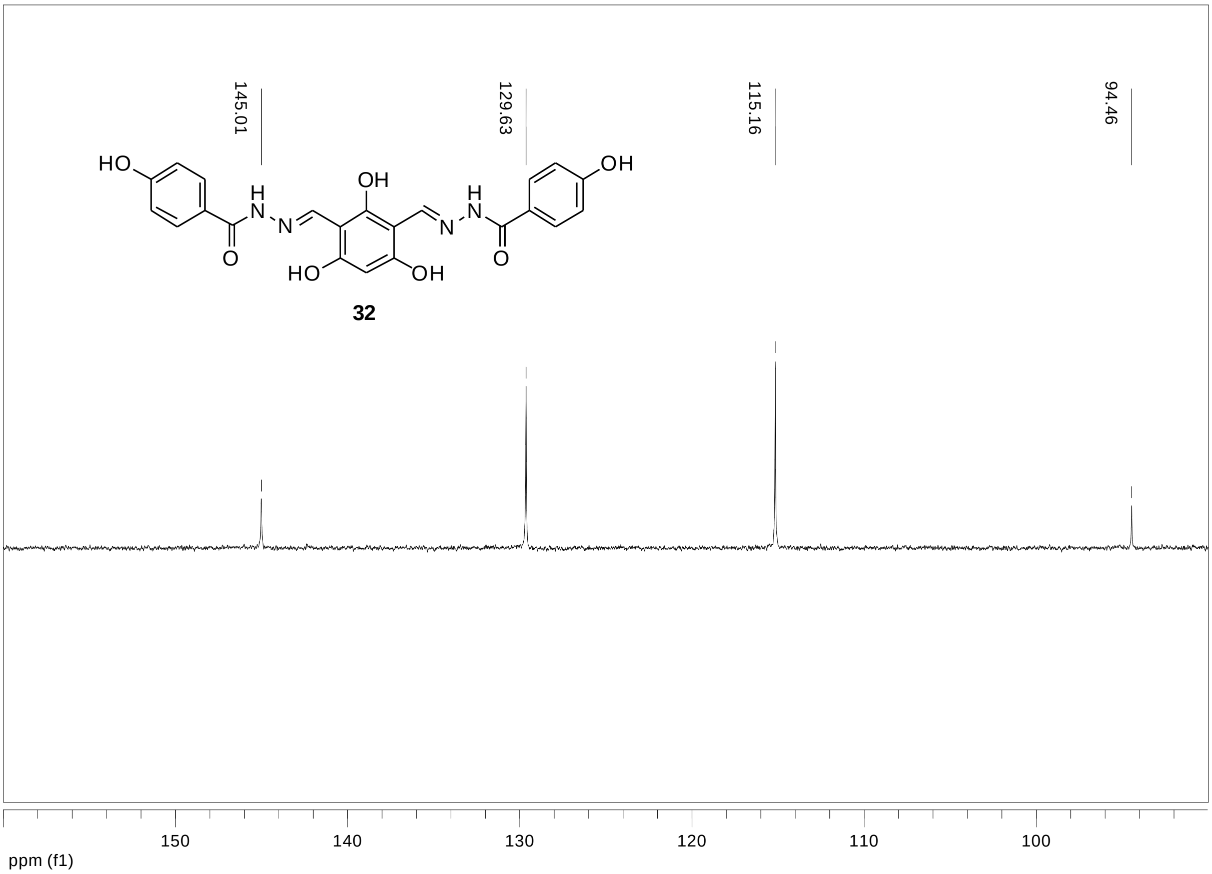

Figure S83. Expansion of ^{13}C-NMR (101 MHz, DMSO-d_6) dept-135 experiment of hydrazone **32**.

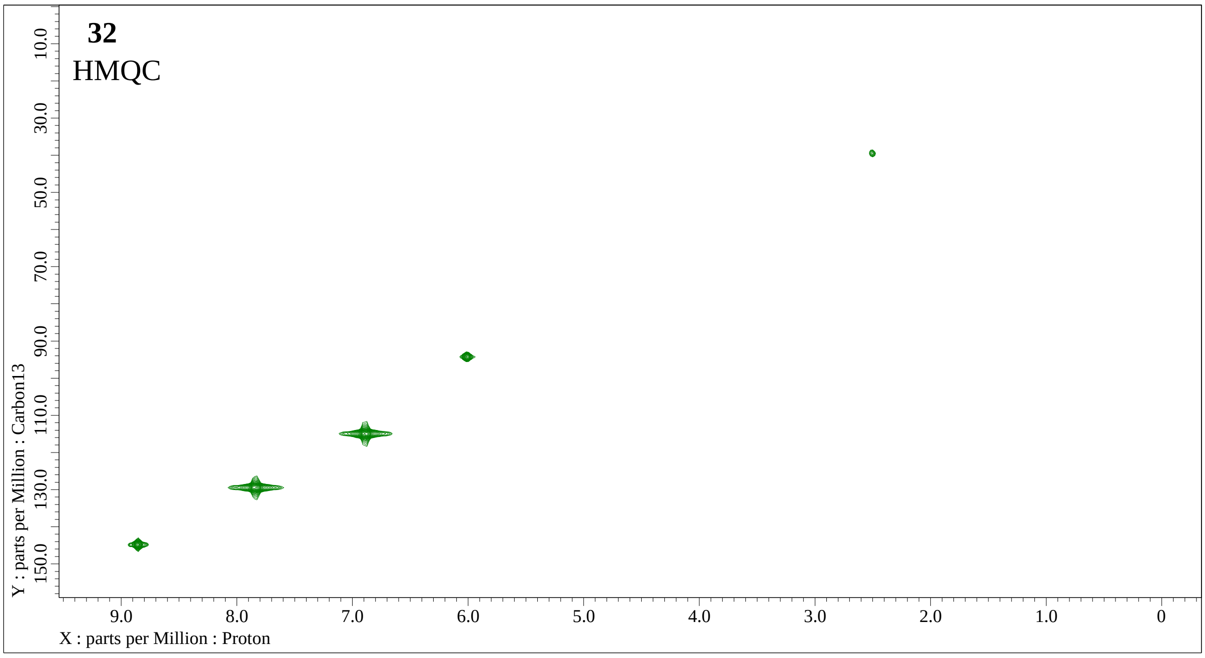

Figure S84. 2D-NMR (400 MHz, DMSO-d_6) HMQC experiment of *N*',*N*''-[(2,4,6-trihydroxy-1,3-phenylene)di-(*E*)-methanylylidene]bis(4-hydroxybenzohydrazide) (**32**).

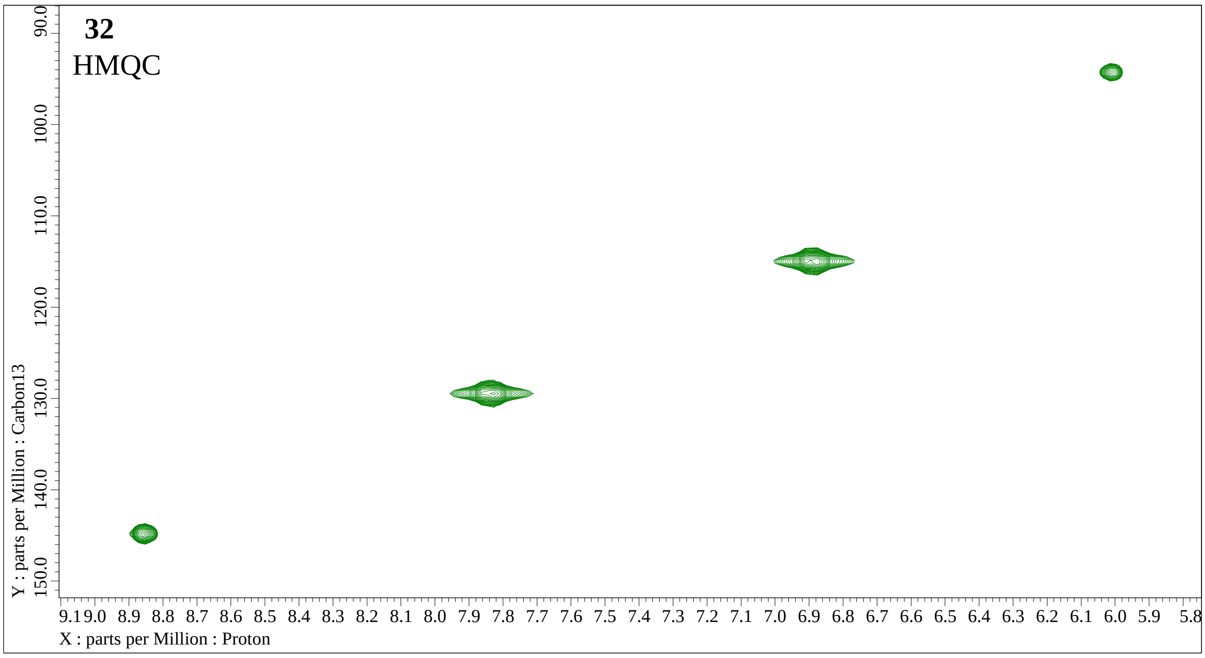

Figure S85. Expansion of 2D-NMR (400 MHz, DMSO-d_6) HMQC experiment of *N*',*N*''-[(2,4,6-trihydroxy-1,3-phenylene)di-(*E*)-methanylylidene]bis(4-hydroxybenzohydrazide) (**32**).

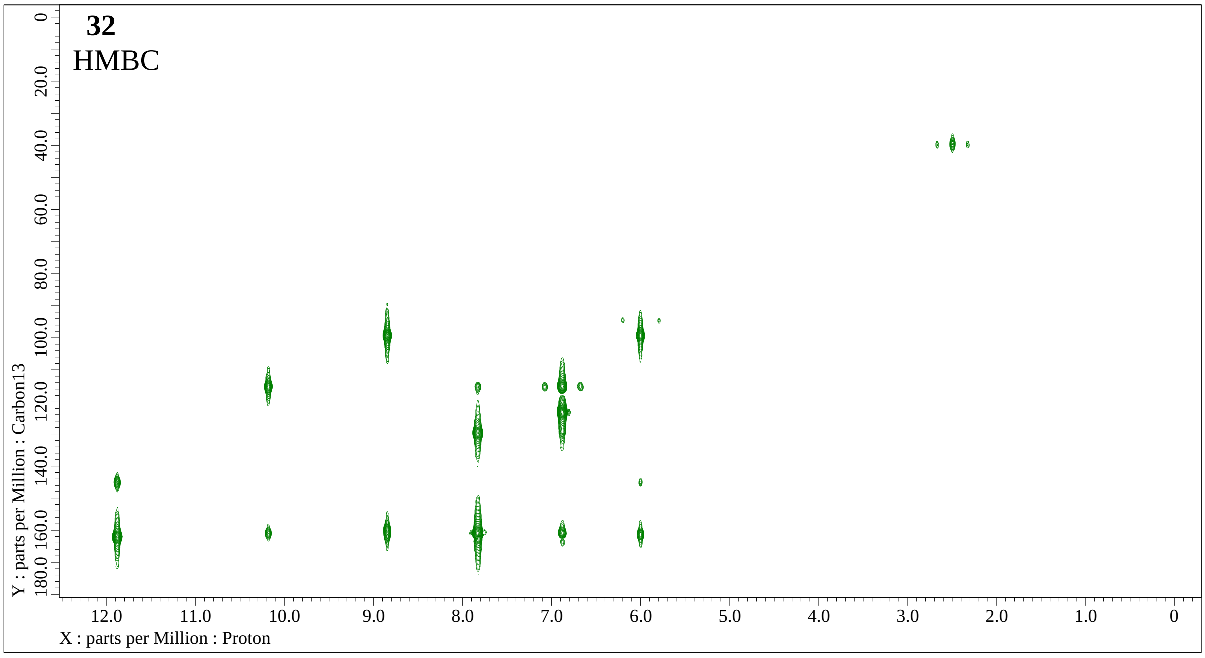

Figure S86. 2D-NMR (400MHz, DMSO-d_6) HMBC experiment of *N*’,*N*’’-[(2,4,6-trihydroxy-1,3-phenylene)di-(*E*)-methanylylidene]bis(4-hydroxybenzohydrazide) (**32**).

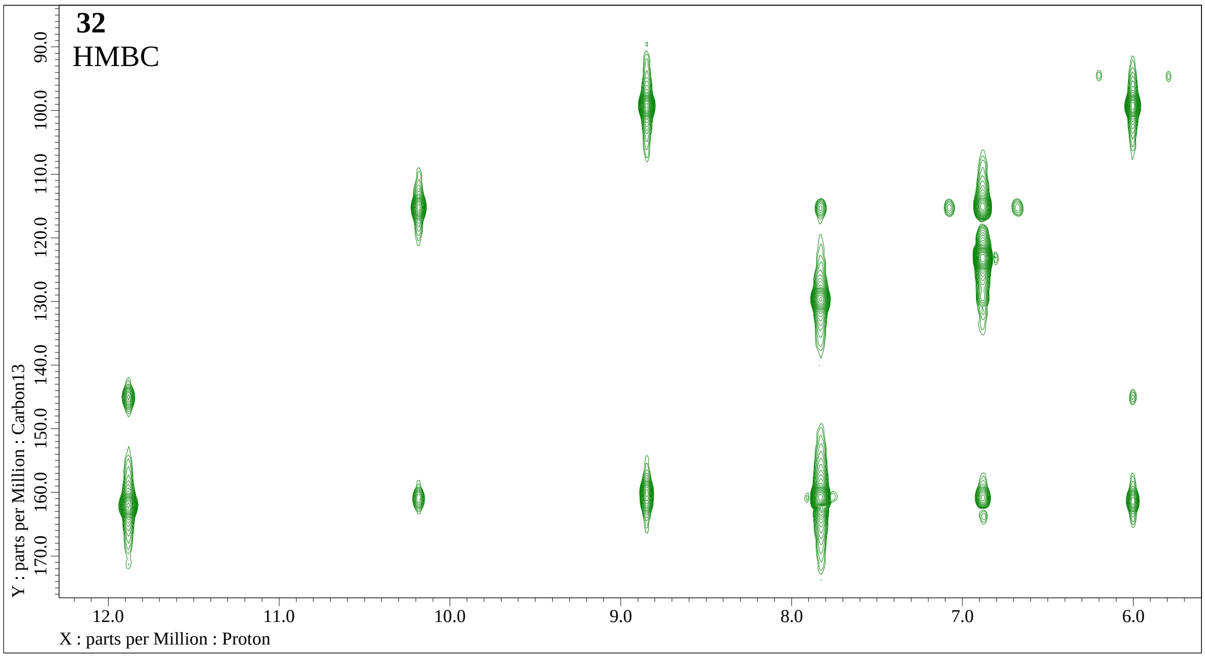

Figure S87. Expansion of 2D-NMR (400 MHz, DMSO-d_6) HMBC experiment of *N*’,*N*’’-[(2,4,6-trihydroxy-1,3-phenylene)di-(*E*)-methanylylidene]bis(4-hydroxybenzohydrazide) (**32**).

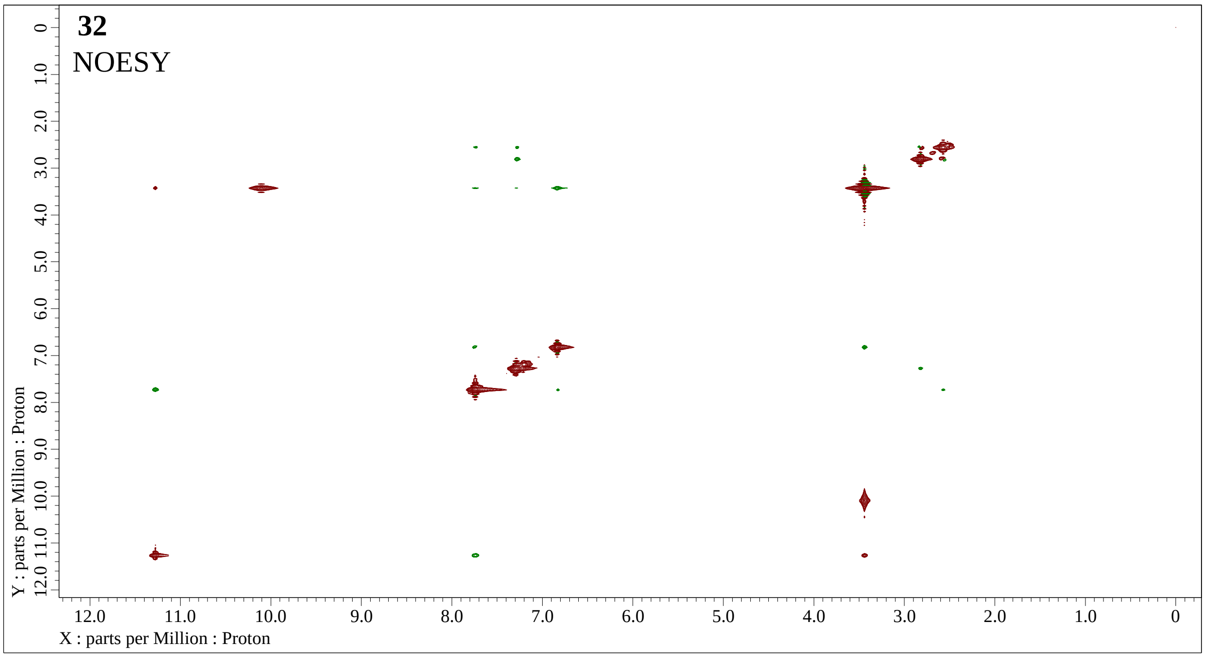

Figure S88. ^{1}H-^{1}H-NMR (400MHz, DMSO-d_6) NOESI experiment of *N*',*N*''-[(2,4,6-trihydroxy-1,3-phenylene)di-(*E*)-methanylylidene]bis(4-hydroxybenzohydrazide) (**32**).

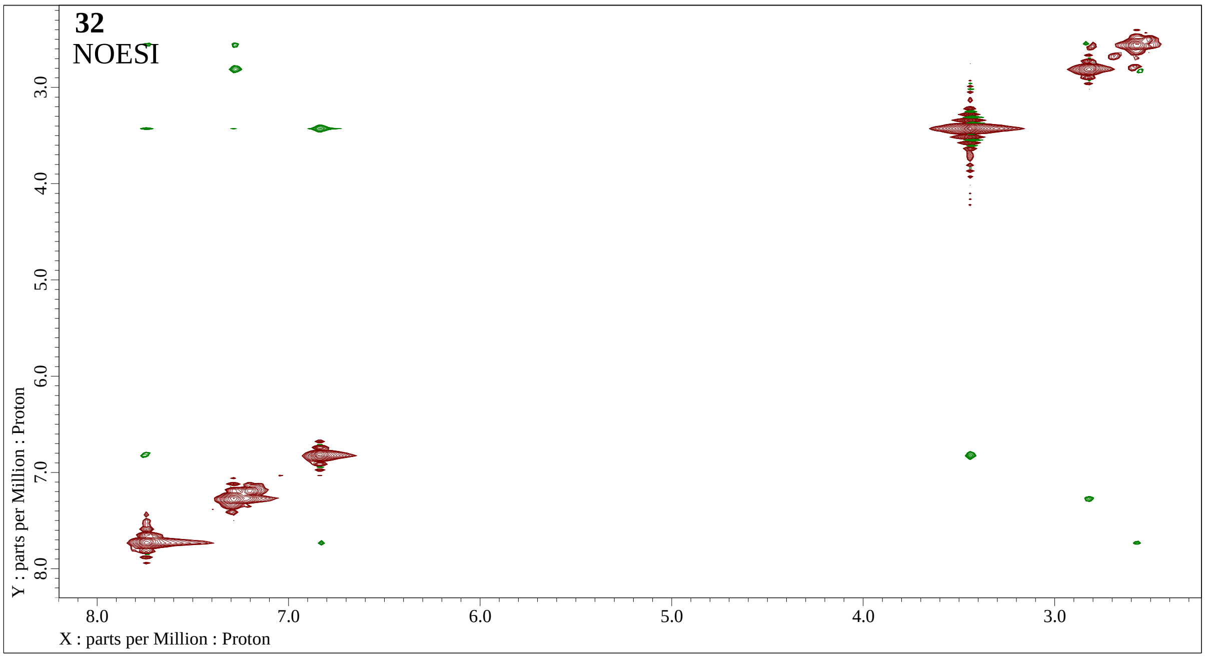

Figure S89. Expansion of ^{1}H-^{1}H NMR (400MHz, DMSO-d_6) NOESI experiment of *N*',*N*''-[(2,4,6-trihydroxy-1,3-phenylene)di-(*E*)-methanylylidene]bis(4-hydroxybenzohydrazide) (**32**).

4-Hydroxygentisaldehyde (60) preparation [6]**.**

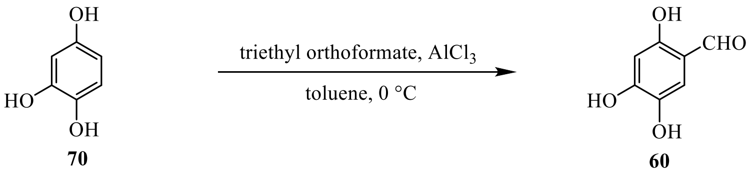

The 4-hydroxygentisaldehyde (**60**) was prepared according to literature procedure [6] by formylation of 1,2,4-trihydroxybenzene (**70**) with triethyl orthoformate in the presence of anhydrous aluminum trichloride ($AlCl_3$) in toluene at 0 °C to obtain yellow brown 4-hydroxy-gentisaldehyde (**60**) which melts at 228.5 °C with decomposition (m.p. 227–229 °C [13]); selected FT-IR (ATR): ν_{max} 3376 (OH), 3142 (br, OH), 2882 (CHO), 1635 (C=O), 1588 (C_{Ar}-H), 1520, 1376, 1283 (br, C_{Ar}-O), 1192 (br, C_{Ar}-O), 1136, 867, 800, 731, 683, 626 (br), 540, 452 cm^{-1}; ^{1}H-NMR (400 MHz, DMSO-d_6) [6]: δ 10.34 (s, 1H, 2-OH), 10.27 (s, 1H, OH), 9.88 (s, 1H, CHO), 8.88 (s, 1H, OH), 6.97 (s, 1H, ArH-6), 6.35 (s, 1H, ArH-3) ppm; ^{13}C-NMR (101 MHz, DMSO-d_6): δ 190.06 (CHO), 156.58 (ArC-2), 154.57 (ArC-4), 139.09 (ArC-5), 114.10 (ArC-6), 113.95 (ArC-1), 102.98 (ArC-3) ppm; HRMS (TOF, MS, ESI) *m*/*z* for $C_7H_6O_4 + H^+$ calculated: 155.0339; found: 155.0345.

The ^{1}H-NMR data appeared in DMSO-d_6 generally is consistent with literature [8] but signals reported in 10.27 ppm as formyl group, and in 9.89 ppm as hydroxy group in our spectrum are detected as hydroxy group (at 10.27 ppm) and formyl group (at 9.88 ppm), respectively. Both protons was correlated by ^{1}H-^{13}C 2D HMBC experiment (Fig S99-S100).

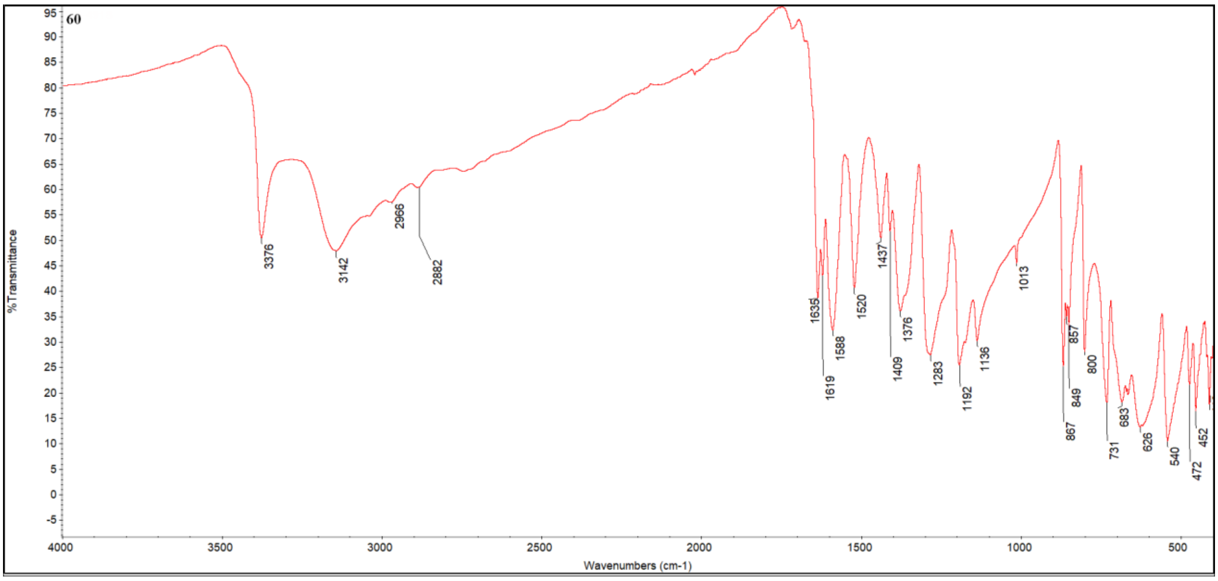

Figure S90. FT-IR (ATR) (4000–400 cm^{-1}) spectrum of 4-hydroxygentisaldehyde (**60**).

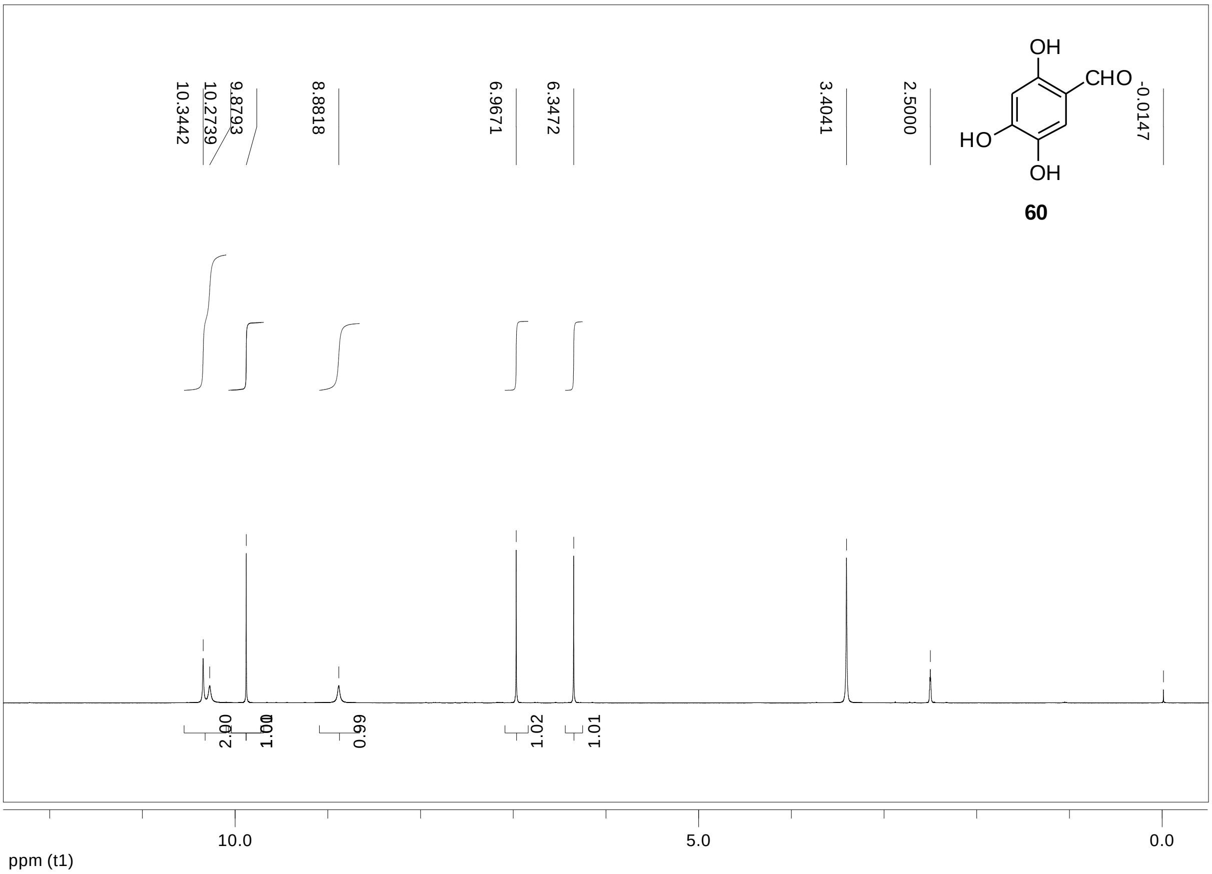

Figure S91. ^{1}H-NMR (400 MHz, DMSO-d_6) spectrum of 4-hydroxygentisaldehyde (**60**).

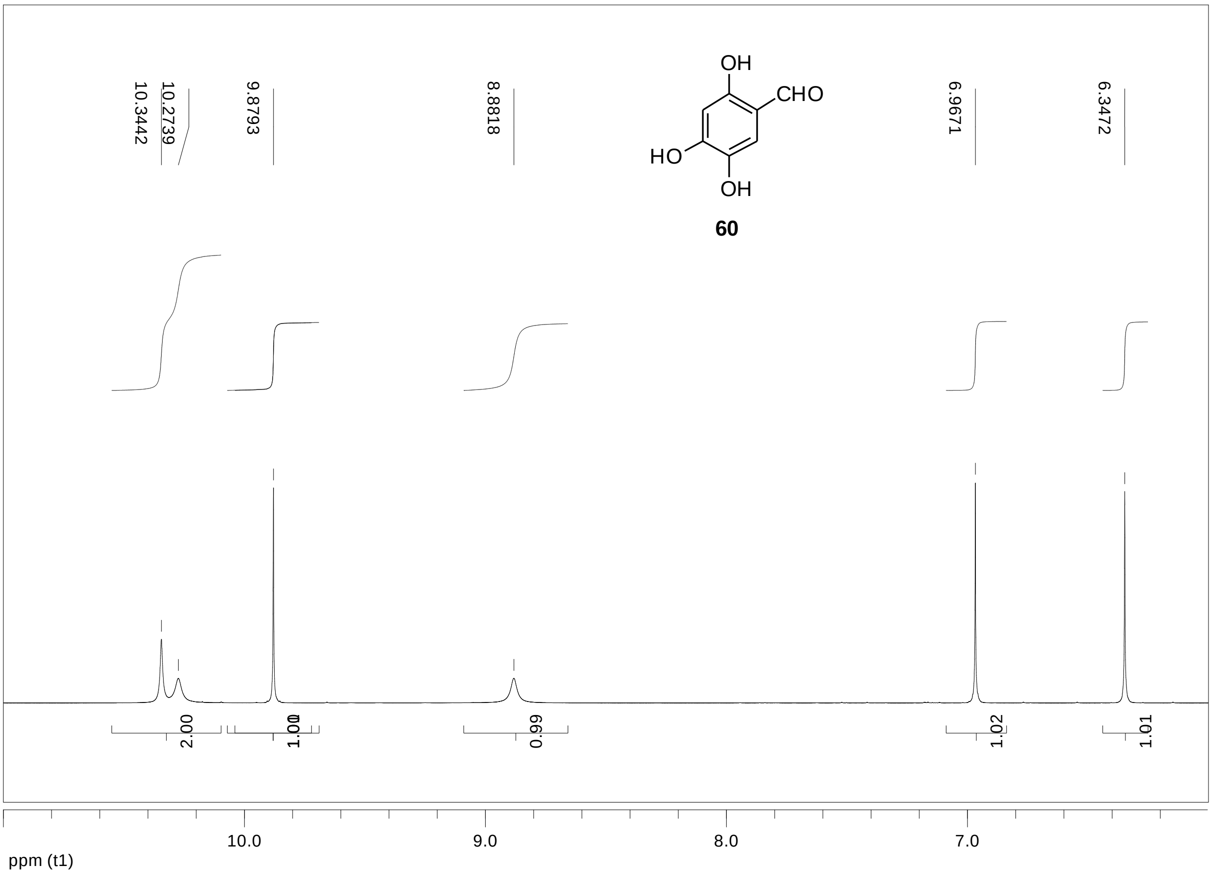

Figure S92. Expansion of ^{1}H-NMR (400 MHz, DMSO-d_6) spectrum of 4-hydroxygentisaldehyde (**60**).

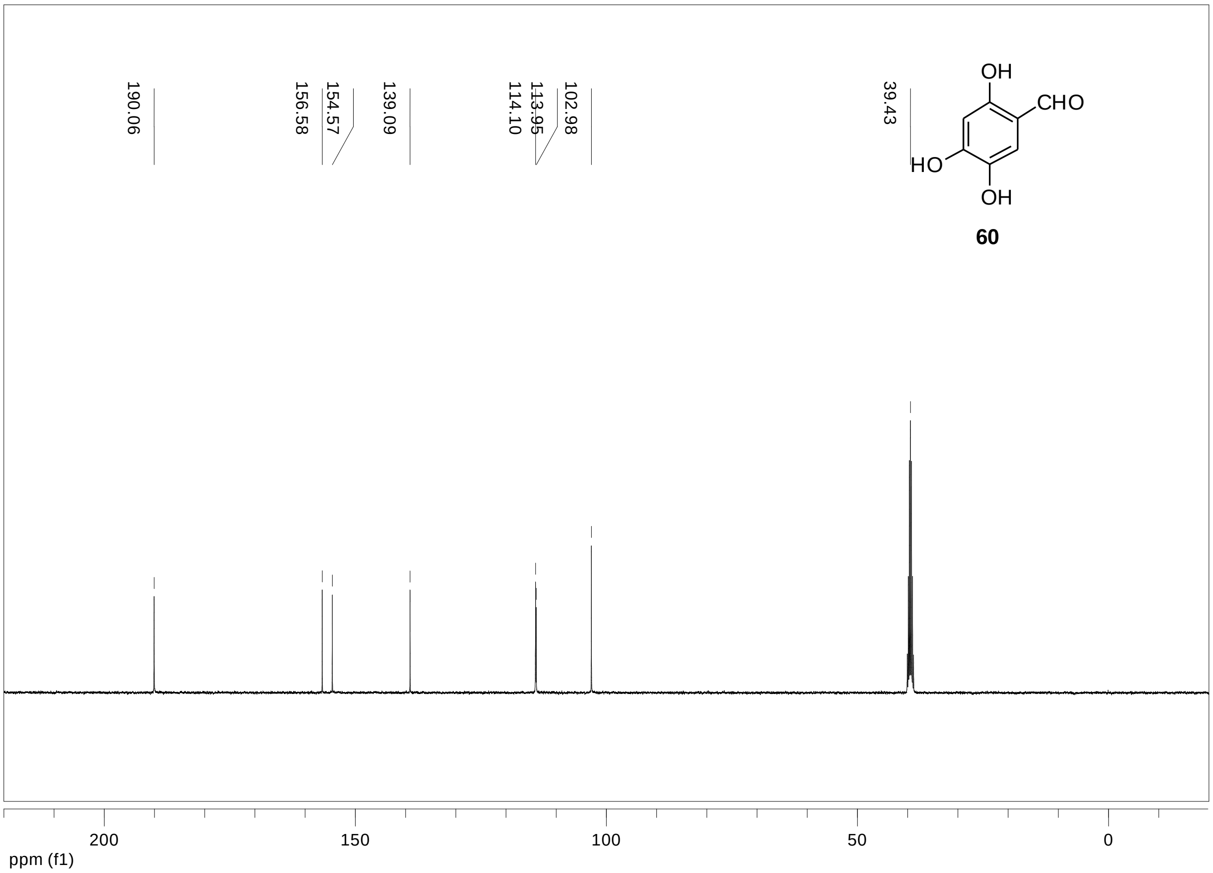

Figure S93. ^{13}C-NMR (101 MHz, DMSO-d_6) spectrum of 4-hydroxygentisaldehyde (**60**).

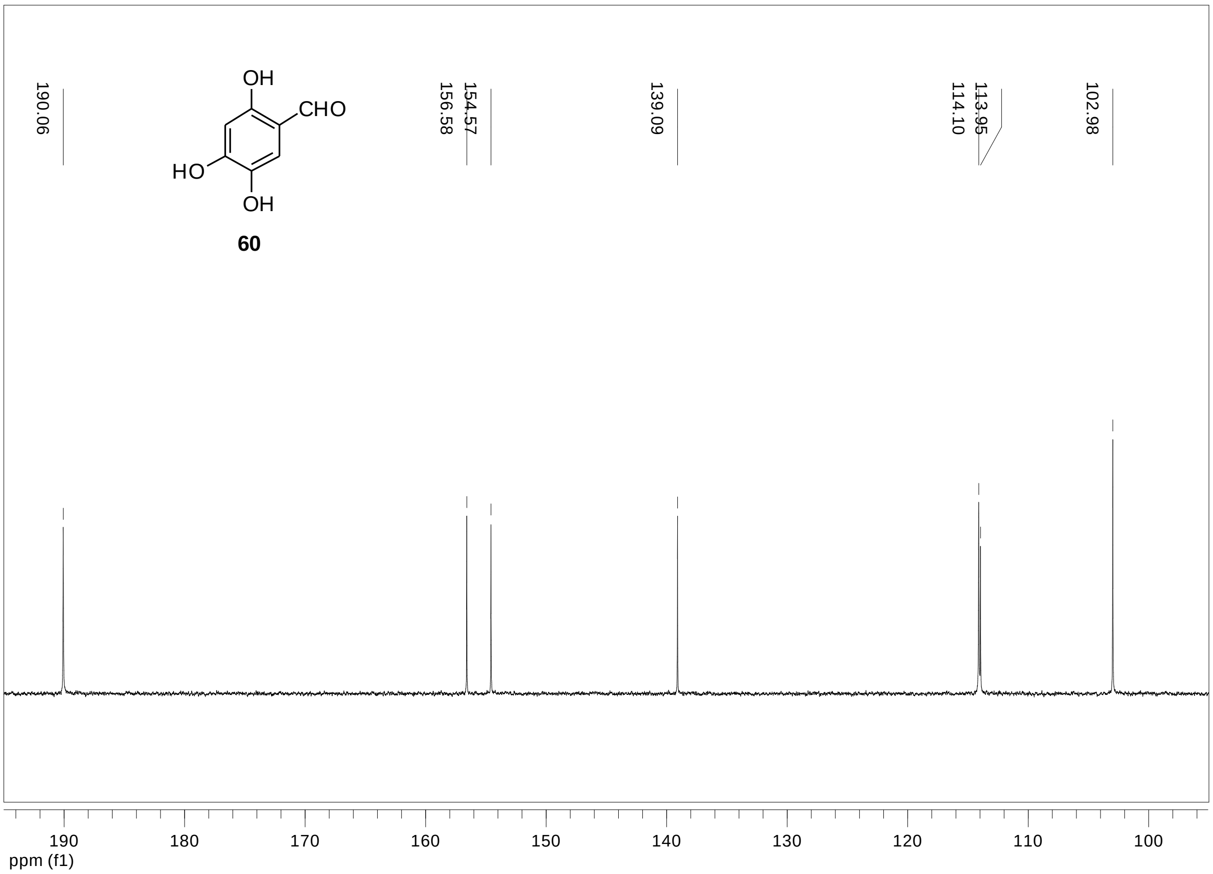

Figure S94. Expansion of ^{13}C-NMR (101 MHz, DMSO-d_6) spectrum of benzaldehyde **60**.

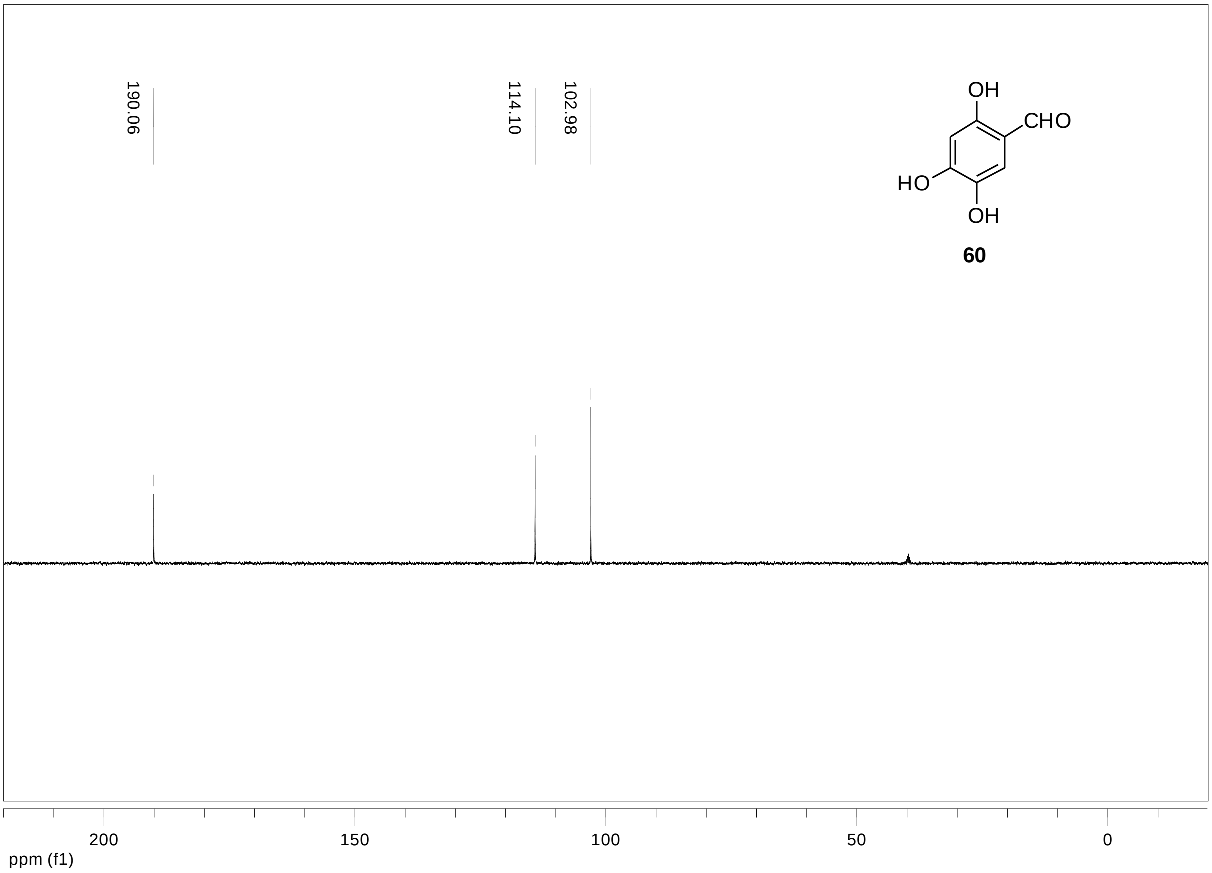

Figure S95. ^{13}C-NMR (101 MHz, DMSO-d_6) dept-135 experiment of 4-hydroxygentisaldehyde (**60**).

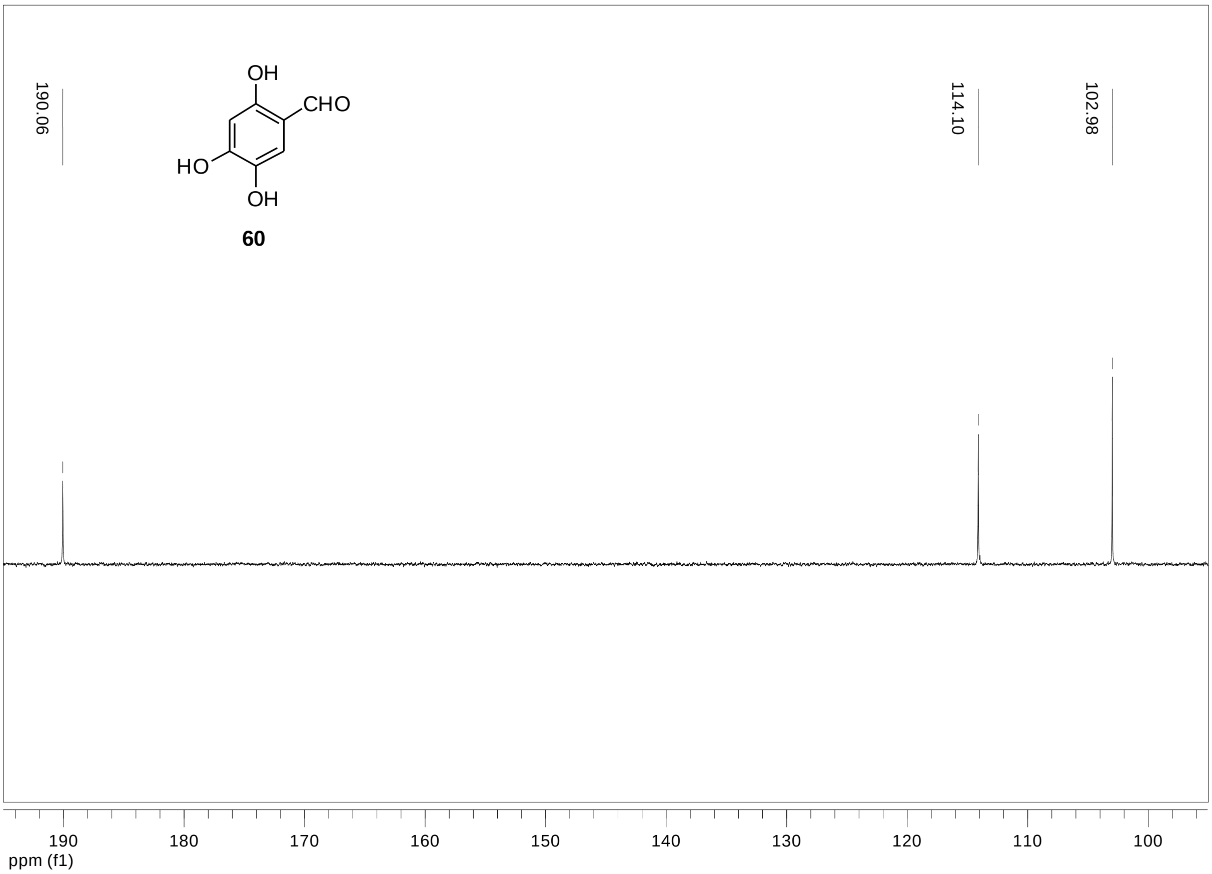

Figure S96. Expansion of ^{13}C-NMR (101 MHz, DMSO-d_6) dept-135 experiment of aldehyde **60**.

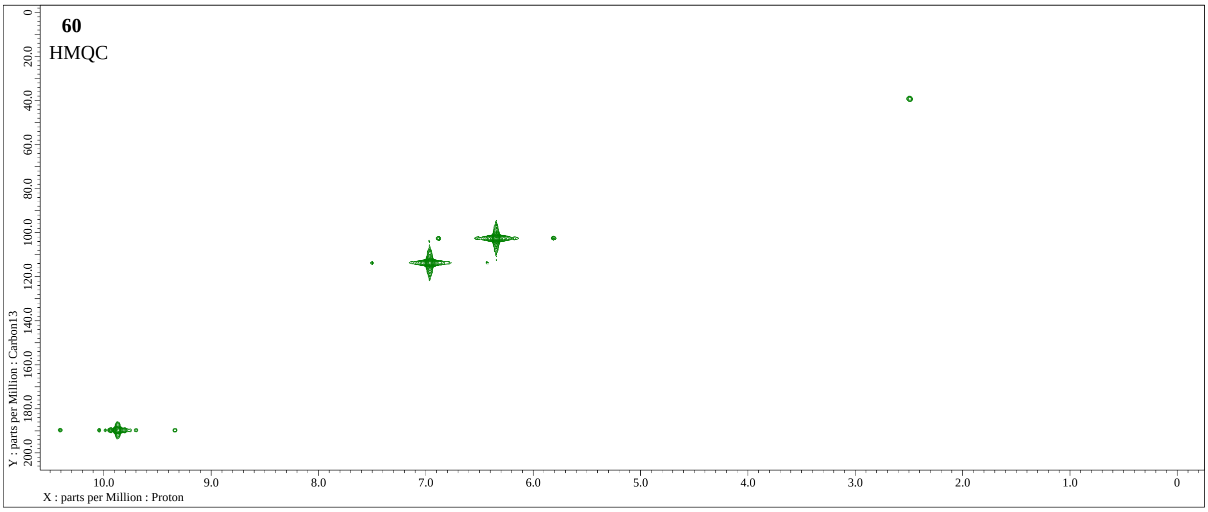

Figure S97. 2D-NMR (400 MHz, DMSO-d_6) HMQC experiment of 4-hydroxygentisaldehyde (**60**).

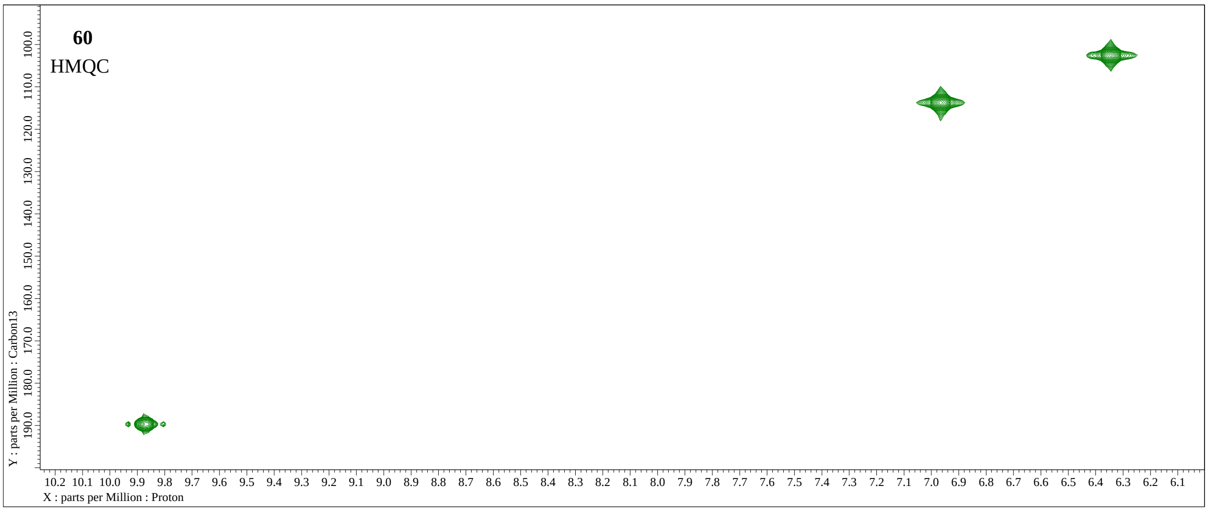

Figure S98. Expansion of 2D-NMR (400 MHz, DMSO-d_6) HMQC experiment of 4-hydroxygentisaldehyde (**60**).

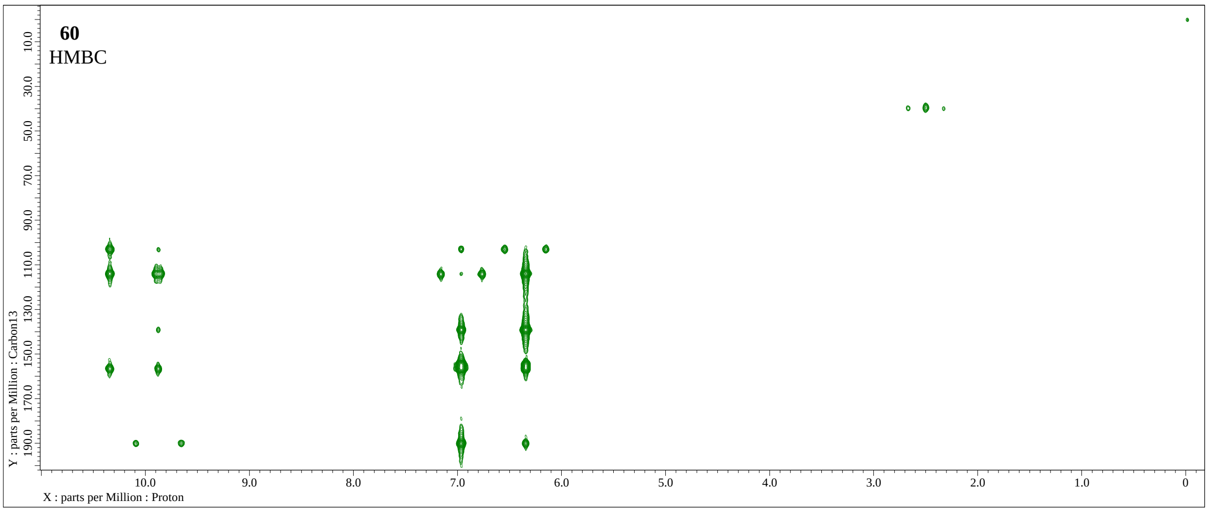

Figure S99. 2D-NMR (400MHz, DMSO-d_6) HMBC experiment of 4-hydroxygentisaldehyde (**60**).

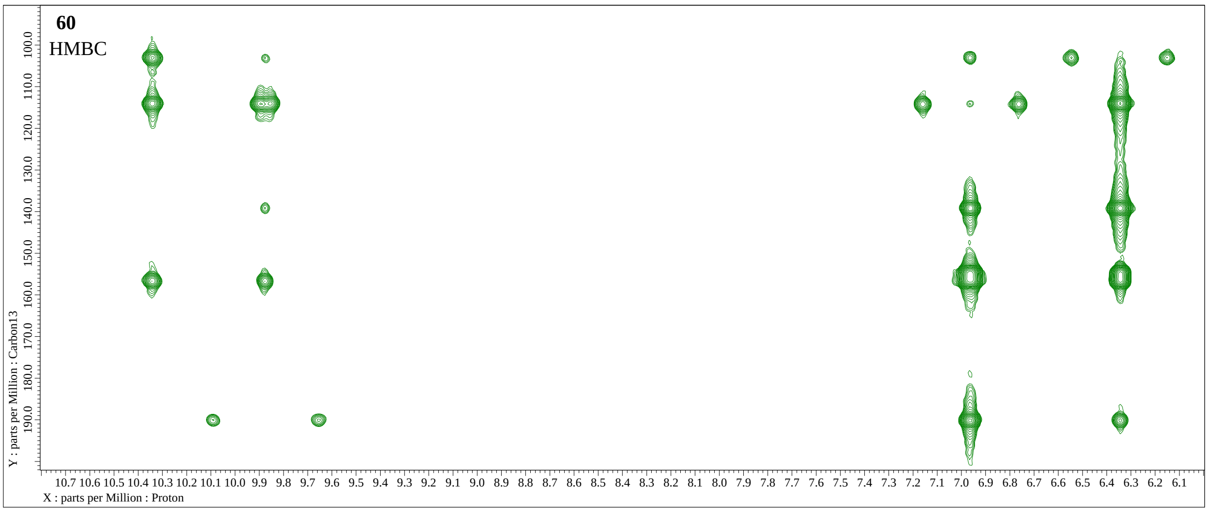

Figure S100. Expansion of 2D-NMR (400 MHz, DMSO-d_6) HMBC experiment of 4-hydroxy-gentisaldehyde (**60**).

Preparation of 2-hydroxy-3-(hydroxymethyl)-5-methylbenzaldehyde (62) [1].

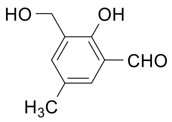

62

The 2-hydroxy-3-hydroxymethyl-5-methylbenzaldehyde (**62**) was prepared with literature procedure [4] from 4-methyl-2,6-bis(hydroxymethyl)phenol by oxidation with active MnO_2 in acetone at 25 °C to obtain the title compound as colorless crystals, which melt at 71–72 °C (m.p. 72–73 °C [1]); HRMS (TOF, MS, ESI): *m*/*z* for $C_9H_{10}O_3 - H_2O + H^+$ calculated: 149.0597; found: 149.0598.

4-Methyl-2,6-bis(hydroxymethyl)phenol substrate; HRMS (TOF, MS, ESI): *m*/*z* for $C_9H_{12}O_3 - H_2O + Na^+$ calculated: 173.0573; found: 173.0555; $C_9H_{12}O_3 + Na^+$ calculated: 191.0679; found: 191.0668.

Preparation of 2-hydroxy-3-(hydroxymethyl)-5-methoxybenzaldehyde (63).

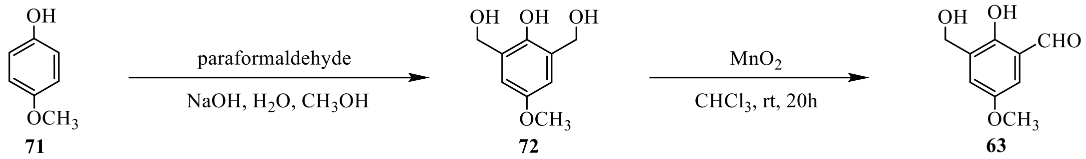

The 2-hydroxy-3-hydroxymethyl-5-methoxybenzaldehyde (**63**) was prepared with literature procedure [7] from 4-methoxy-2,6-bis(hydroxymethyl)phenol (**72**) [9] by oxidation with active MnO_2 in $CHCl_3$ at 25 °C with 20 hours to obtain the 2-hydroxy-3-hydroxymethyl-5-methoxybenzaldehyde (**63**) as yellow needles, which melt at 89.5–90.5 °C (m.p. 89–91 °C [7]); selected FT-IR (ATR): ν_{max} 3527 (O-H), 3453 (O-H), 3184 (br, O-H), 2971 (C-H), 1636 (C=O), 1589 (C=C), 1570 (C_{Ar}-H), 1510, 1429 (C_{Ar}-H), 1284, 1244 (C_{Ar}-O), 1197 (C_{Ar}-O), 1162 (CH_2-O), 1066, 1030 (CH_3-O), 964, 861, 809, 748, 688, 619, 460 cm^{-1}; ^{1}H-NMR (400 MHz, $CDCl_3$): δ 10.96 (s, 1H, CHO), 9.86 (s, 1H, OH), 7.22 (d, ^4J = 3.1 Hz, 1H, ArH-4), 6.94 (d, ^4J = 3.1 Hz, 1H, ArH-6), 4.73 (s, 2H, CH_2), 3.82 (s, 3H, OCH_3), 2.44 (s, br, 1H, CH_2<u>OH</u>) ppm; ^{13}C-NMR (101 MHz, $CDCl_3$): δ 196.29 (CH=O), 153.67 (ArC-2), 152.57 (ArC-5), 130.70 (ArC-2), 123.66 (ArC-4), 119.82 (ArC-1), 114.28 (ArC-6), 60.49 (CH_2), 55.91 (OCH_3) ppm; HRMS (TOF, MS, ESI): *m*/*z* for $C_9H_{10}O_4 - H_2O + H^+$ calculated: 165.0546; found: 165.0544.

The 4-methoxy-2,6-bis(hydroxymethyl)phenol (**72**) substrate, was prepared by diformylation of 4-methoxyphenol (**71**) with formaldehyde on alkaline water solution [9] and the crude product was used directly in the oxidation reaction toward the 2-hydroxy-3-hydroxymethyl-5-methoxybenzaldehyde (**63**).

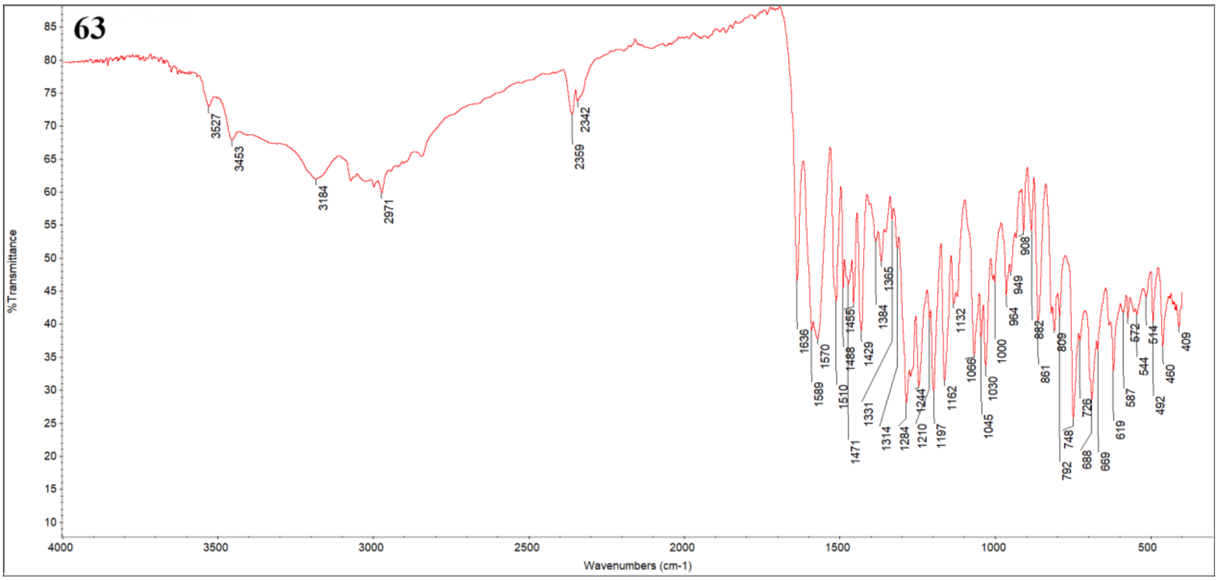

Figure S101. FT-IR (ATR) (4000–400 cm^{-1}) spectrum of 2-hydroxy-3-(hydroxymethyl)-5-methoxy-benzaldehyde (**63**).

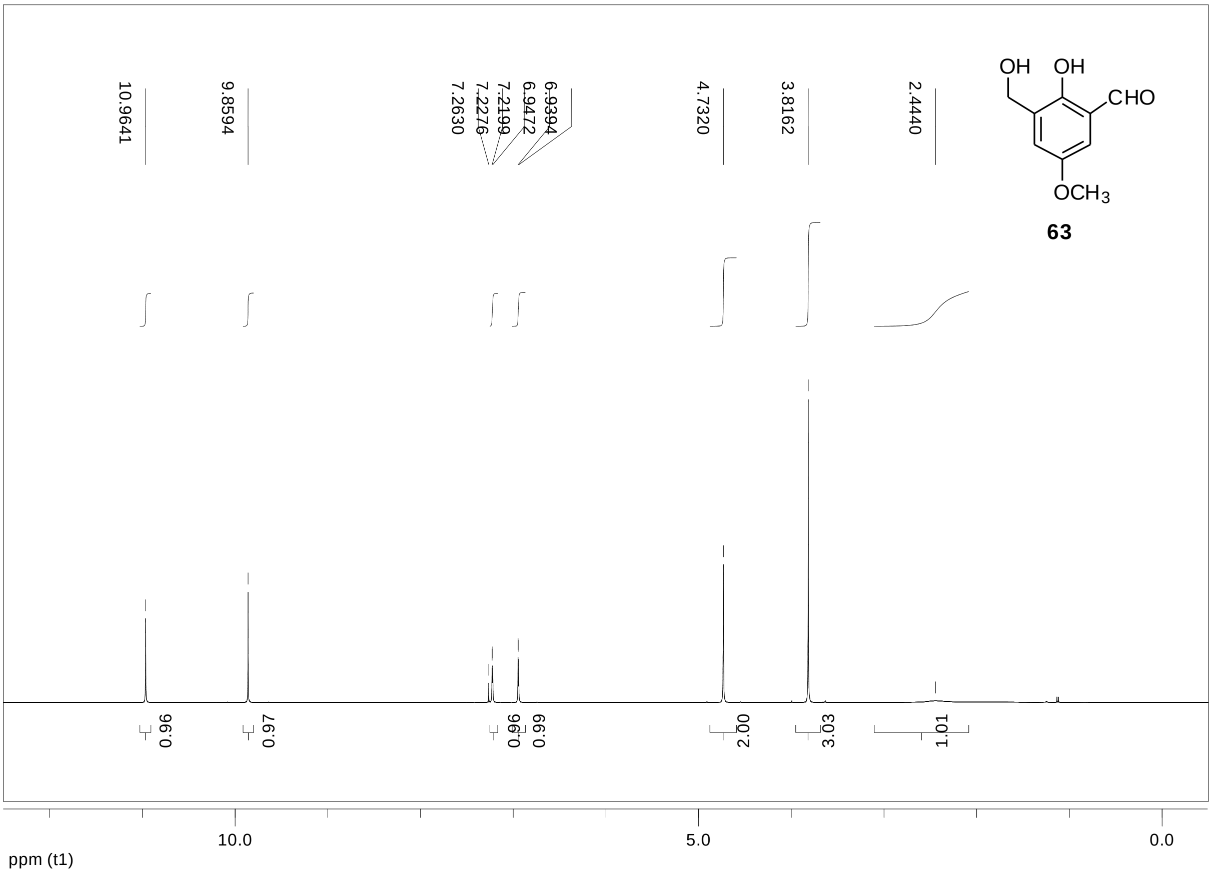

Figure S102. ^{1}H-NMR (400 MHz, $CDCl_3$) spectrum of salicylic aldehyde **63**.

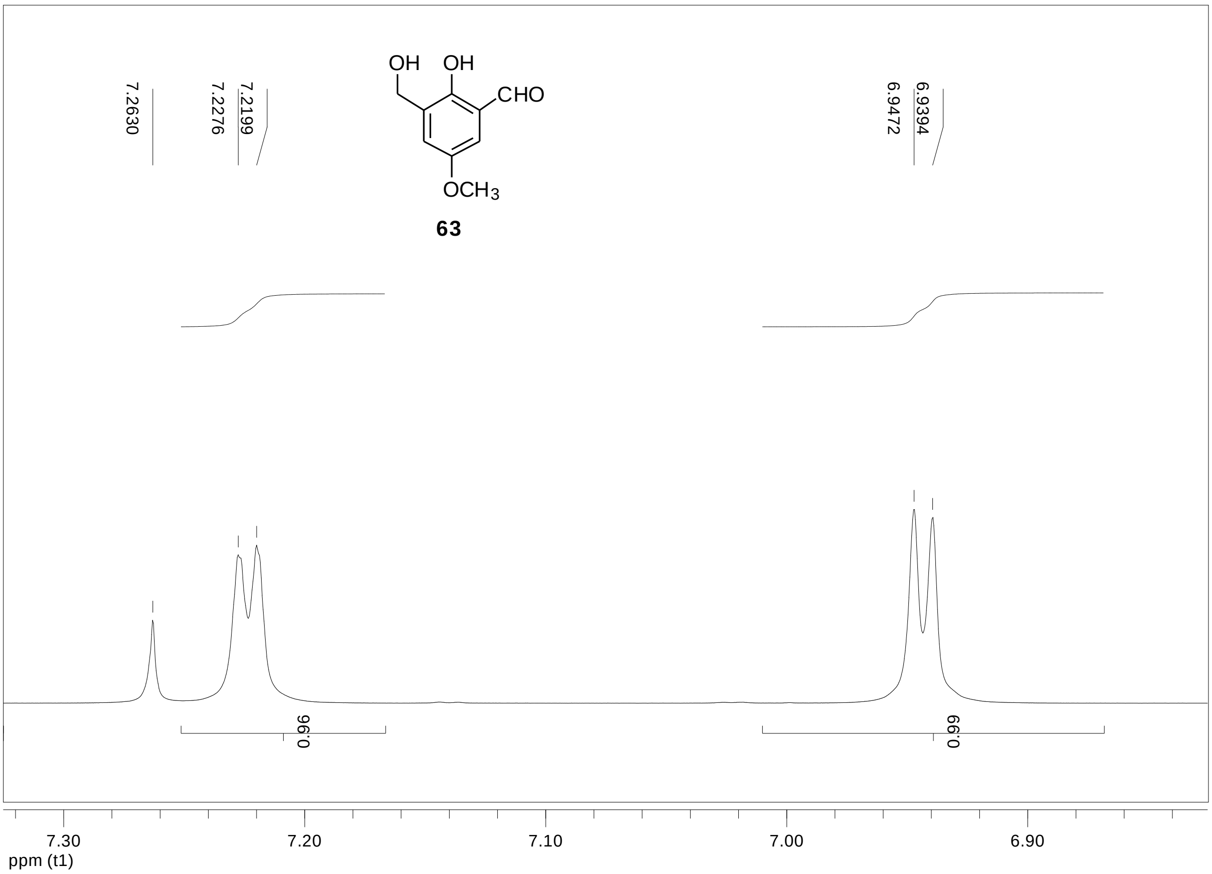

Figure S103. Expansion of ^{1}H-NMR (400 MHz, $CDCl_3$) spectrum of salicylic aldehyde **63**.

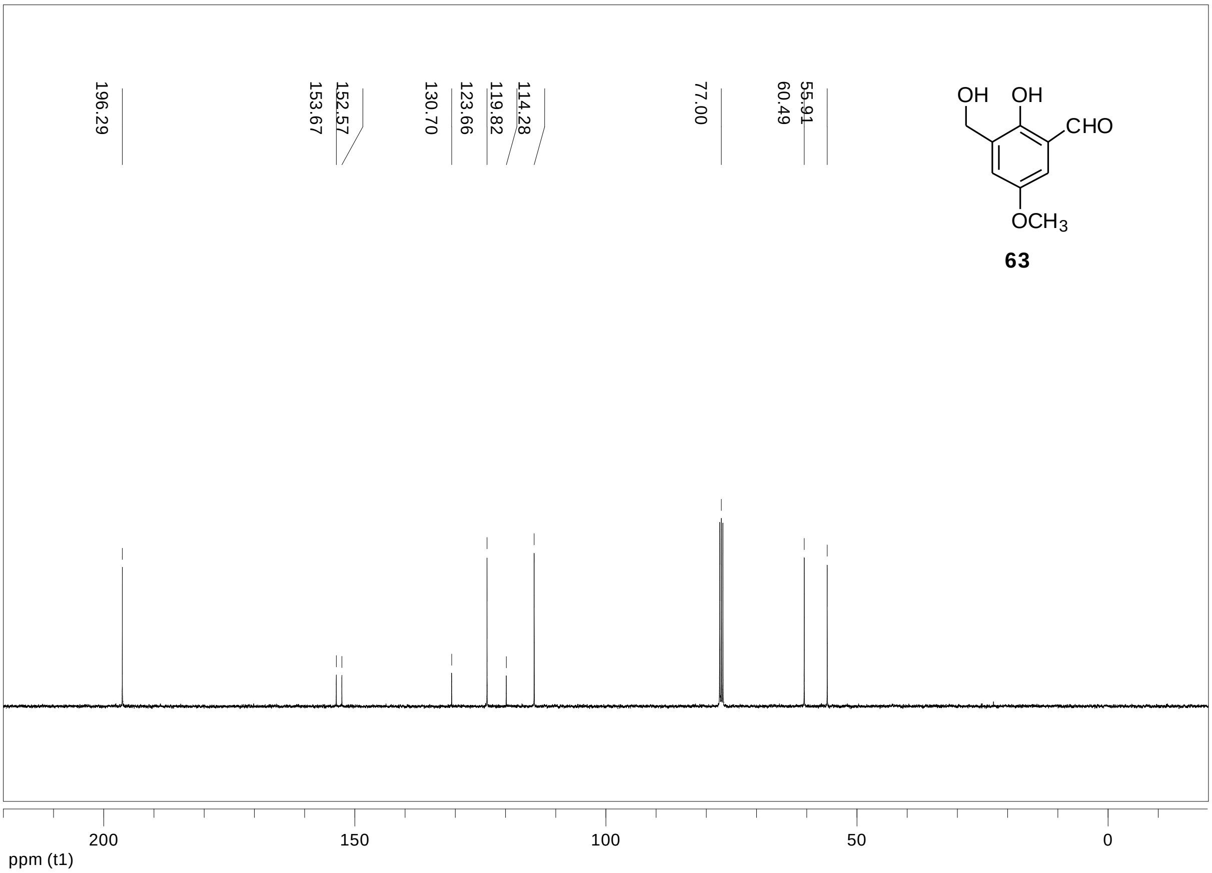

Figure S104. ^{13}C-NMR (101 MHz, $CDCl_3$) spectrum of salicylic aldehyde **63**.

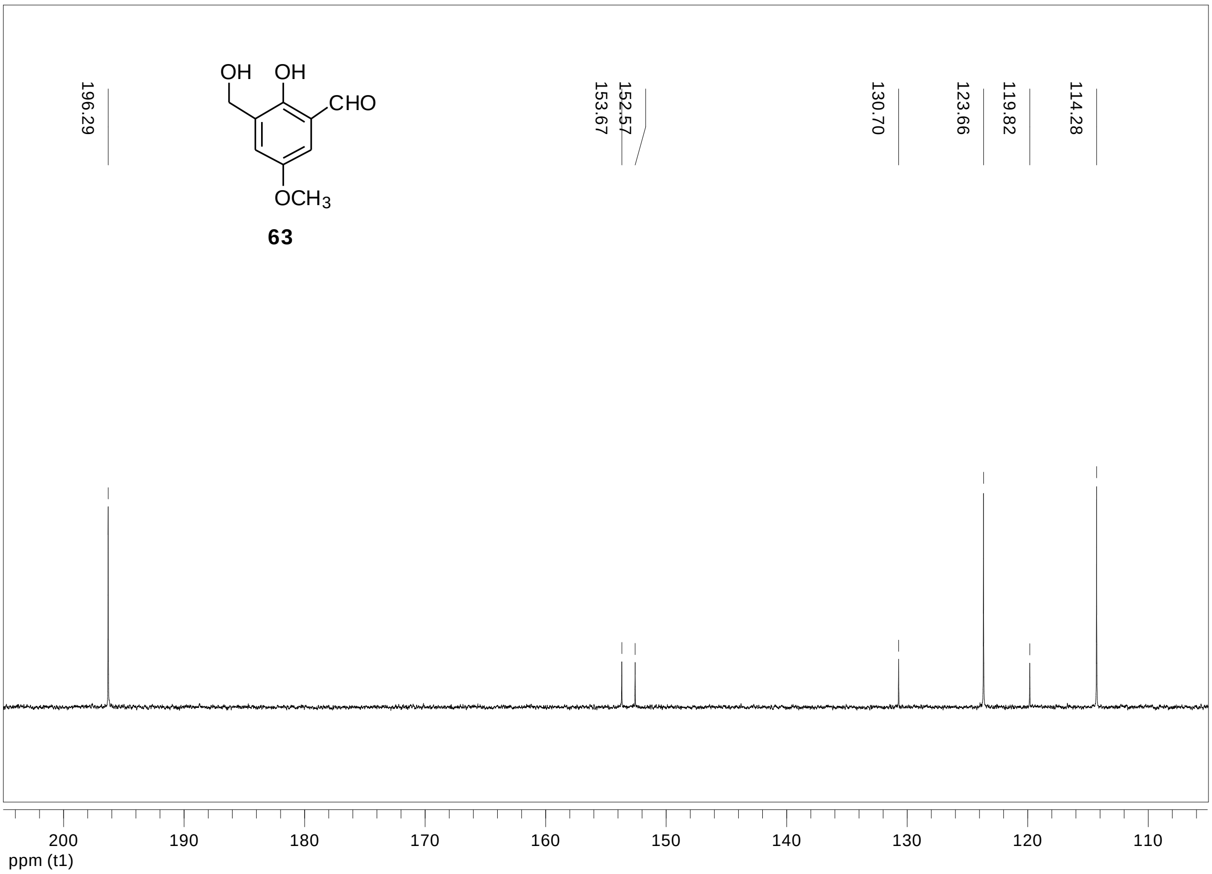

Figure S105. Expansion of ^{13}C-NMR (101 MHz, $CDCl_3$) spectrum of salicylic aldehyde **63**.

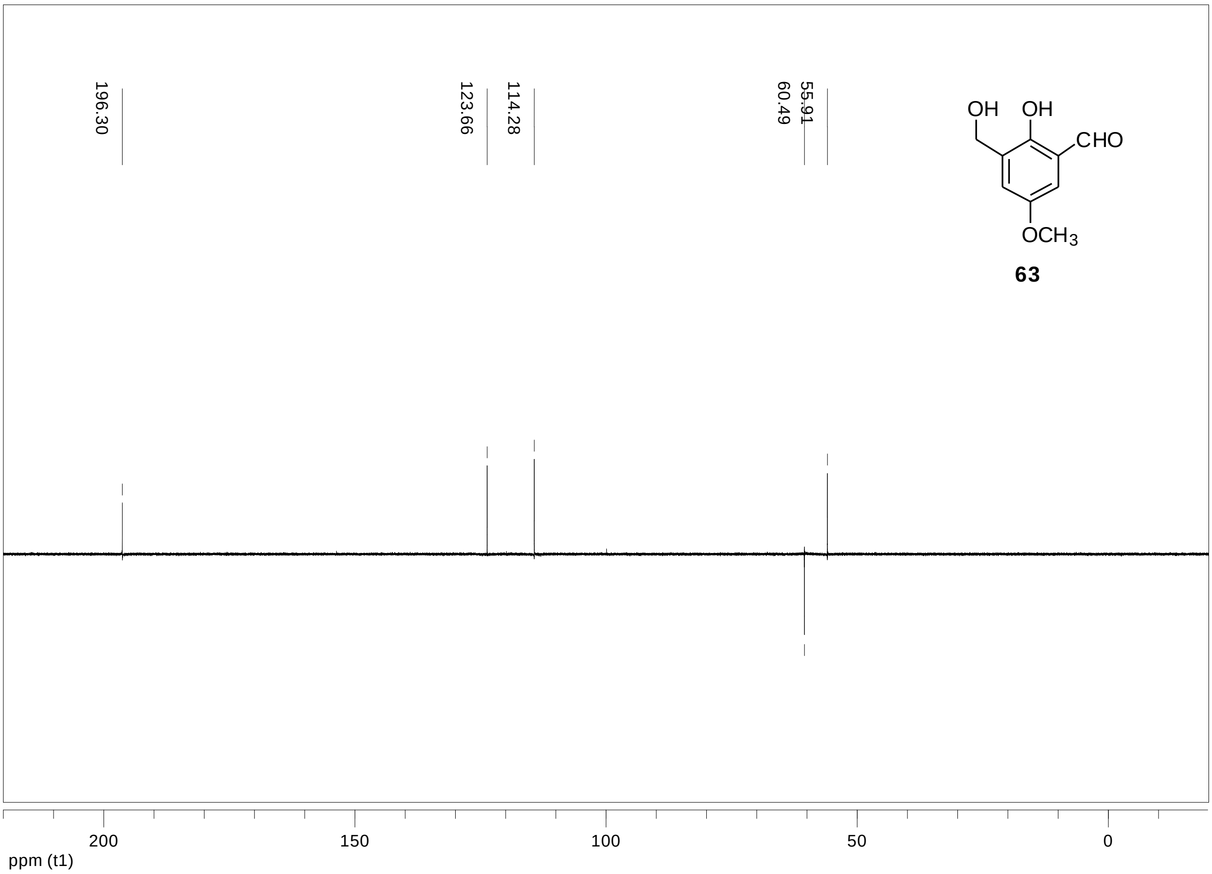

Figure S106. ^{13}C-NMR (101 MHz, $CDCl_3$) dept-135 experiment of salicylic aldehyde **63**.

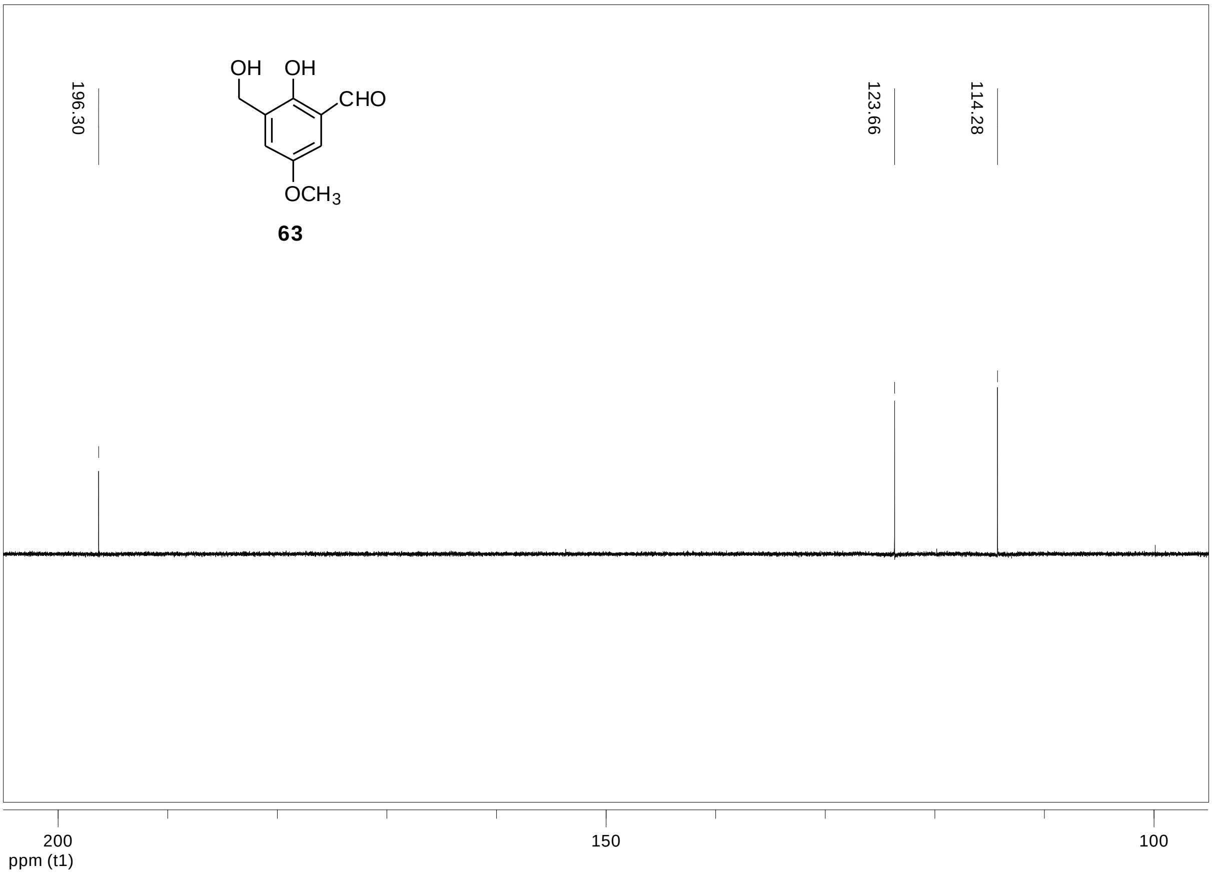

Figure S107. Expansion of ^{13}C-NMR (101 MHz, $CDCl_3$) dept-135 experiment of aldehyde **63**.

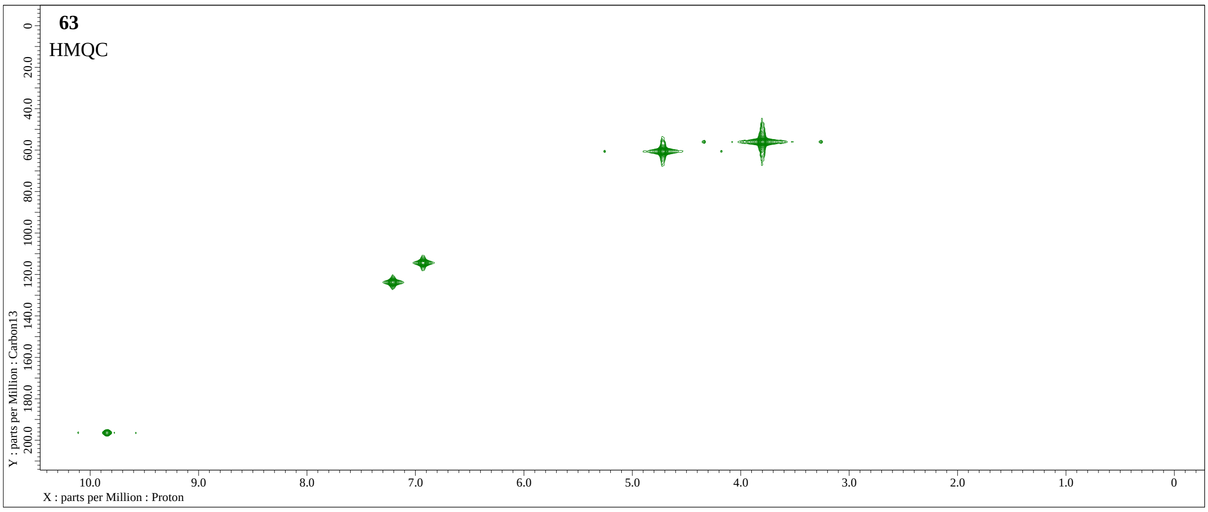

Figure S108. 2D-NMR (400 MHz, $CDCl_3$) HMQC experiment of 2-hydroxy-3-(hydroxymethyl)-5-methoxybenzaldehyde (**63**).

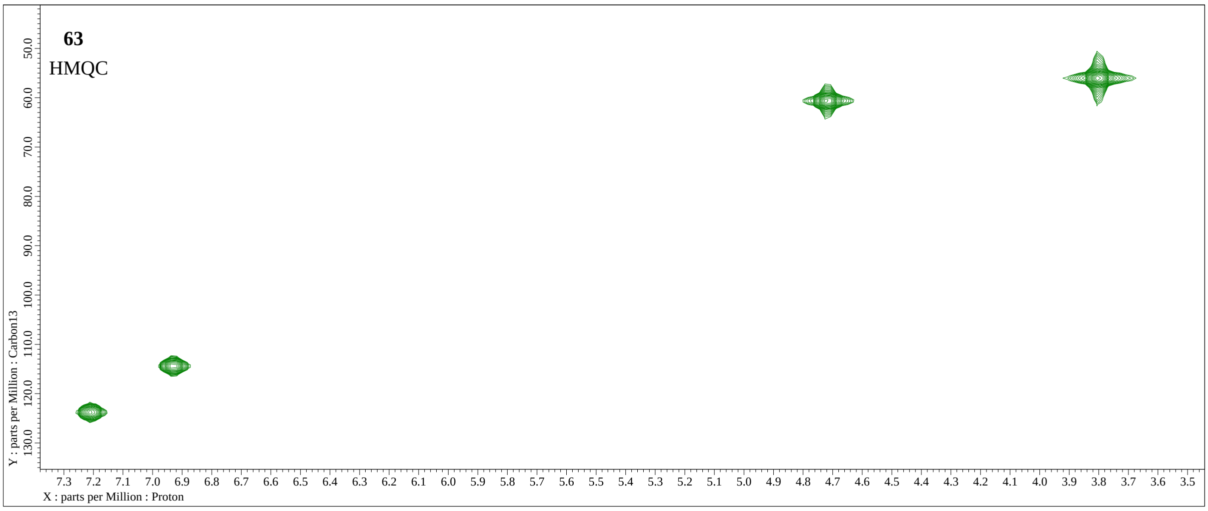

Figure S109. Expansion of 2D-NMR (400 MHz, $CDCl_3$) HMQC experiment of 2-hydroxy-3-(hydroxymethyl)-5-methoxybenzaldehyde (**63**).

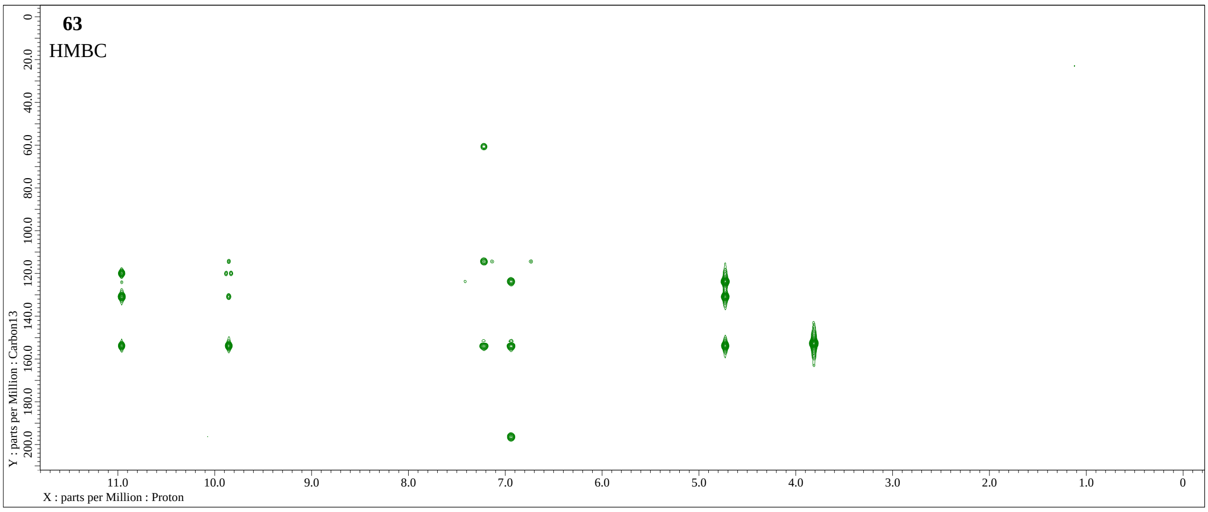

Figure S110. 2D-NMR (400 MHz, $CDCl_3$) HMBC experiment of 2-hydroxy-3-(hydroxymethyl)-5-methoxybenzaldehyde (**63**).

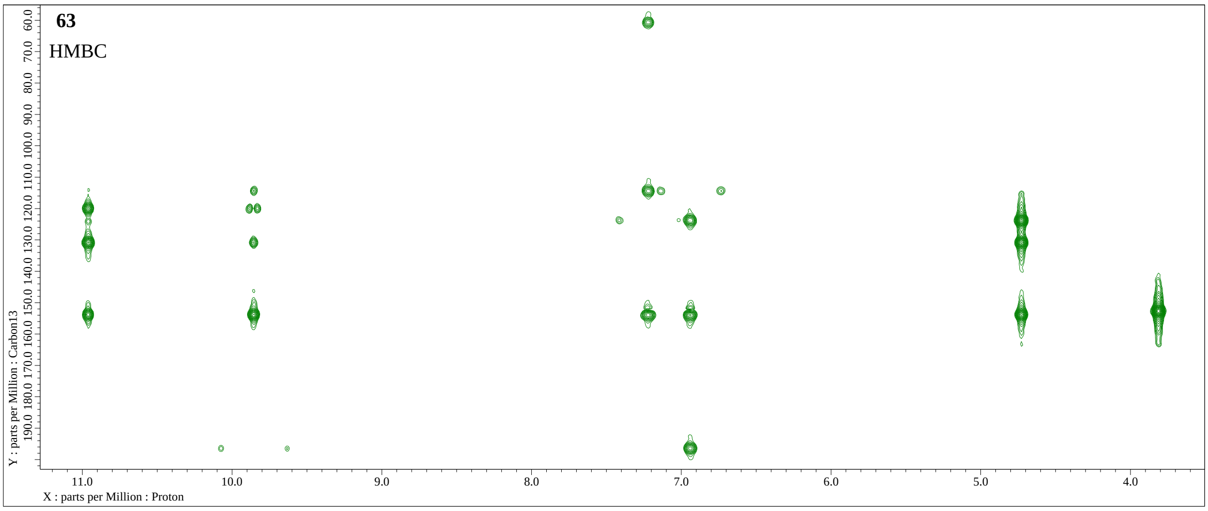

Figure S111. Expansion of 2D-NMR (400 MHz, $CDCl_3$) HMBC experiment of 2-hydroxy-3-(hydroxymethyl)-5-methoxybenzaldehyde (**63**).

Preparation of 3-isopropyl-6-methylsalicylic aldehyde (65) [1].

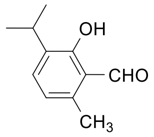

65

A literature protocol of thymol *ortho* formylation was applied [5] to obtain the 3-isopropyl-6-methylsalicylic aldehyde (**65**) as a colorless transparent oil with 67% yield which boil at 260–261 °C (b.p. 261 °C at 760 mmHg [1]); selected FT-IR (ATR): ν_{max} 3040 (C_{Ar}-H), 2961 (C-H), 2873 (CHO), 1633 (C=O), 1617 (C=C), 1423 (C_{Ar}-H), 1325, 1300, 1224 (C_{Ar}-O), 1150, 1067, 965, 722, 733, 594, 548 cm^{-1}; ^{1}H-NMR (400 MHz, DMSO-d_6): δ 12.32 (s, 1H, OH), 10.27 (s, 1H, CHO), 7.38 (d, ^{3}J = 7.7 Hz, 1H, ArH-4), 6.75 (d, ^{3}J = 7.7 Hz, 1H, ArH-5), 3.31 (m, ^{3}J = 6.9 Hz, 1H, CH), 2.55 (s, 3H, CH_3), 1.15 (d, ^{3}J = 6.9 Hz, 6H, CH(<u>CH_3</u>)$_2$) ppm; ^{13}C-NMR (101 MHz, DMSO-d_6): δ 197.42 (CHO), 159.58 (C-2), 139.99 (C-6), 133.84 (C-3), 133.69 (C-4), 121.44 (C-5), 117.76 (C-1), 25.40 (<u>CH</u>(CH_3)$_2$), 22.02 (CH(<u>CH_3</u>)$_2$), 17.47 (CH_3) ppm; HRMS (TOF, MS, ESI) *m/z* for $C_{11}H_{14}O_2 + H^+$ calculated: 179.1067; found: 179.1065.

^{1}H-NMR and ^{13}C-NMR data are consistent with our literature values [1].

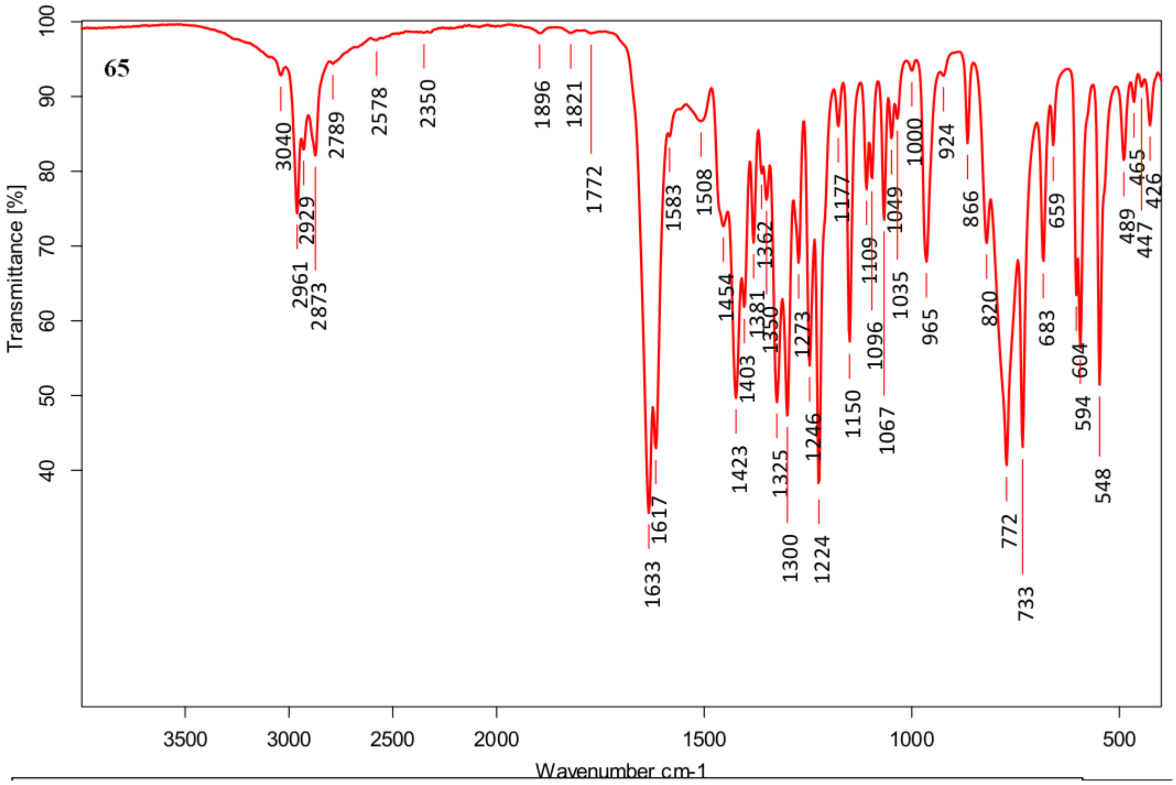

Figure S112. FT-IR (ATR) (4000–400 cm^{-1}) spectrum of 3-*iso*-propyl-6-methylsalicylic aldehyde (**65**).

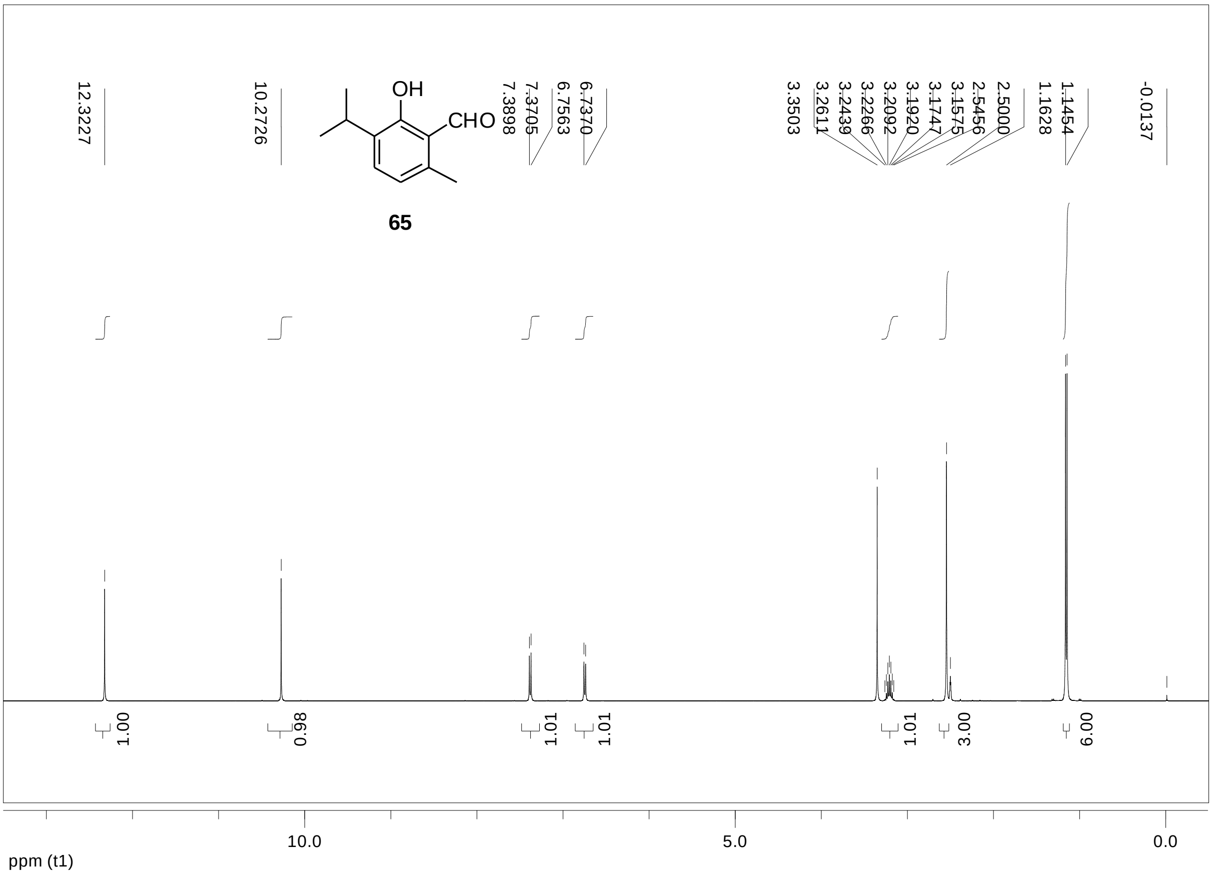

Figure S113. ^{1}H-NMR (400 MHz, DMSO-d_6) spectrum of salicylic aldehyde **65**.

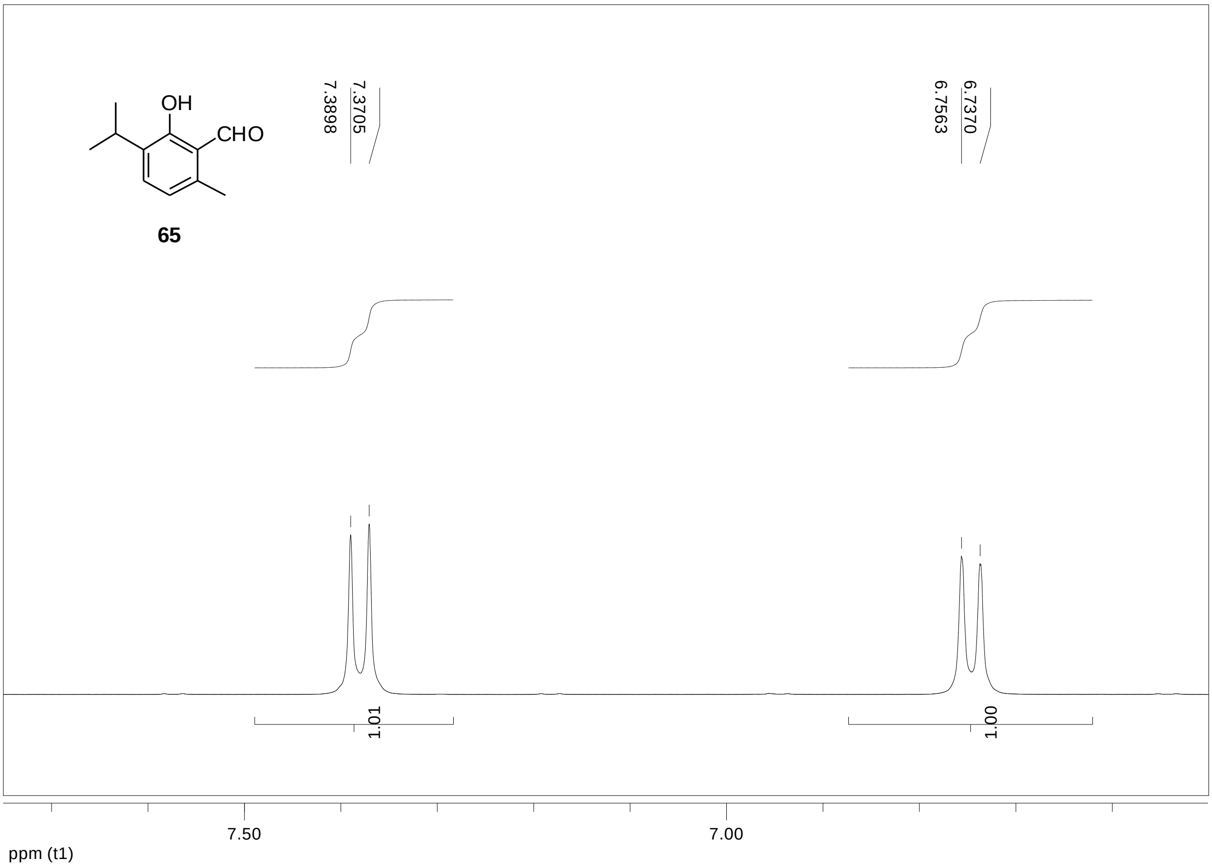

Figure S114. Expansion of ^{1}H-NMR (400 MHz, DMSO-d_6) spectrum of salicylic aldehyde **65**.

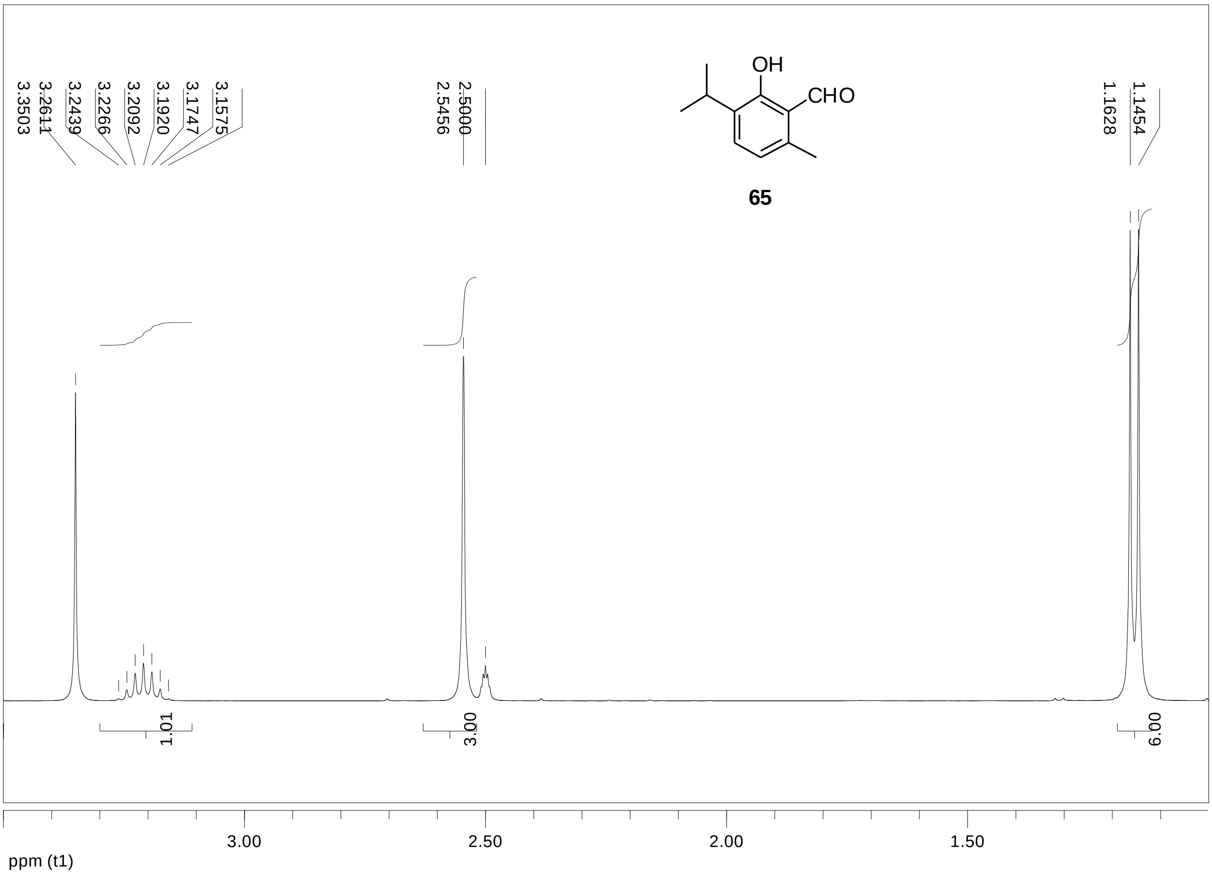

Figure S115. Expansion of ^{1}H-NMR (400 MHz, DMSO-d_6) spectrum of salicylic aldehyde **65**.

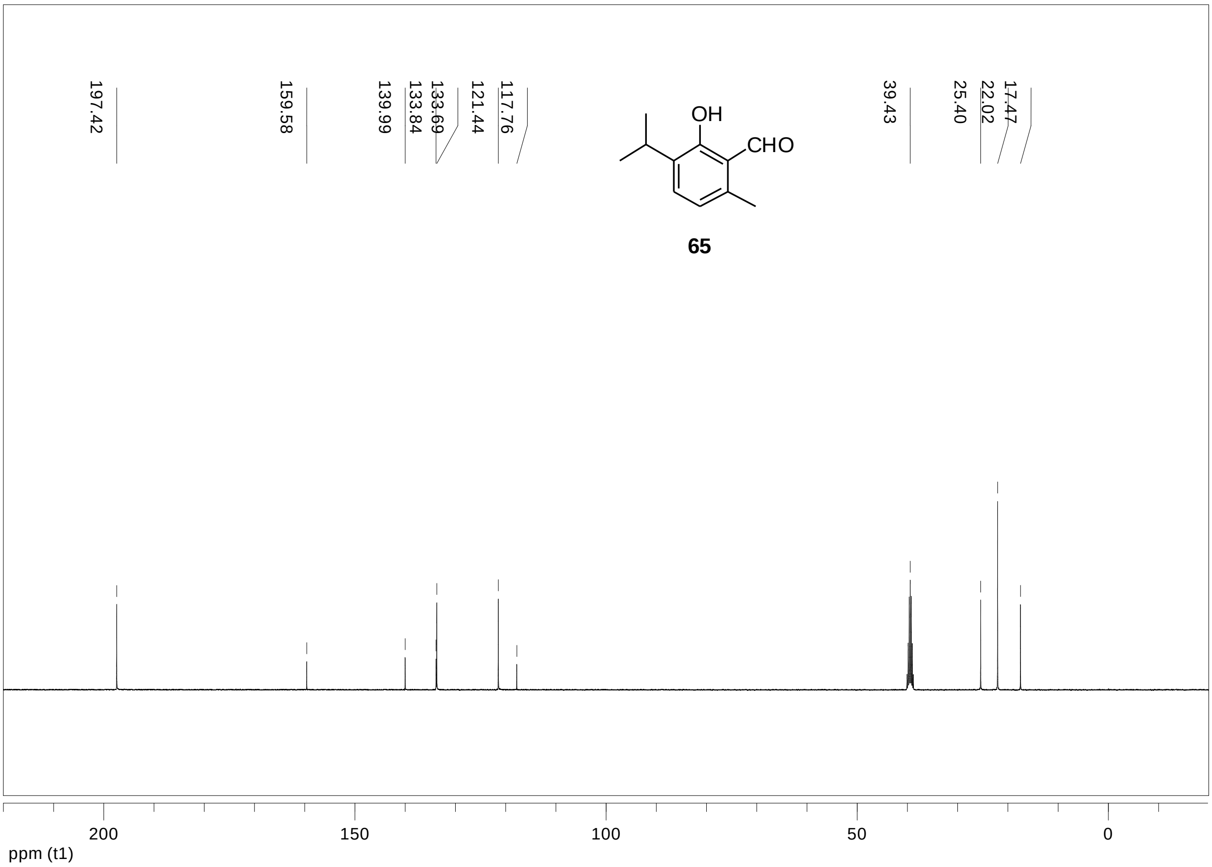

Figure S116. ^{13}C-NMR (101 MHz, DMSO-d_6) spectrum of salicylic aldehyde **65**.

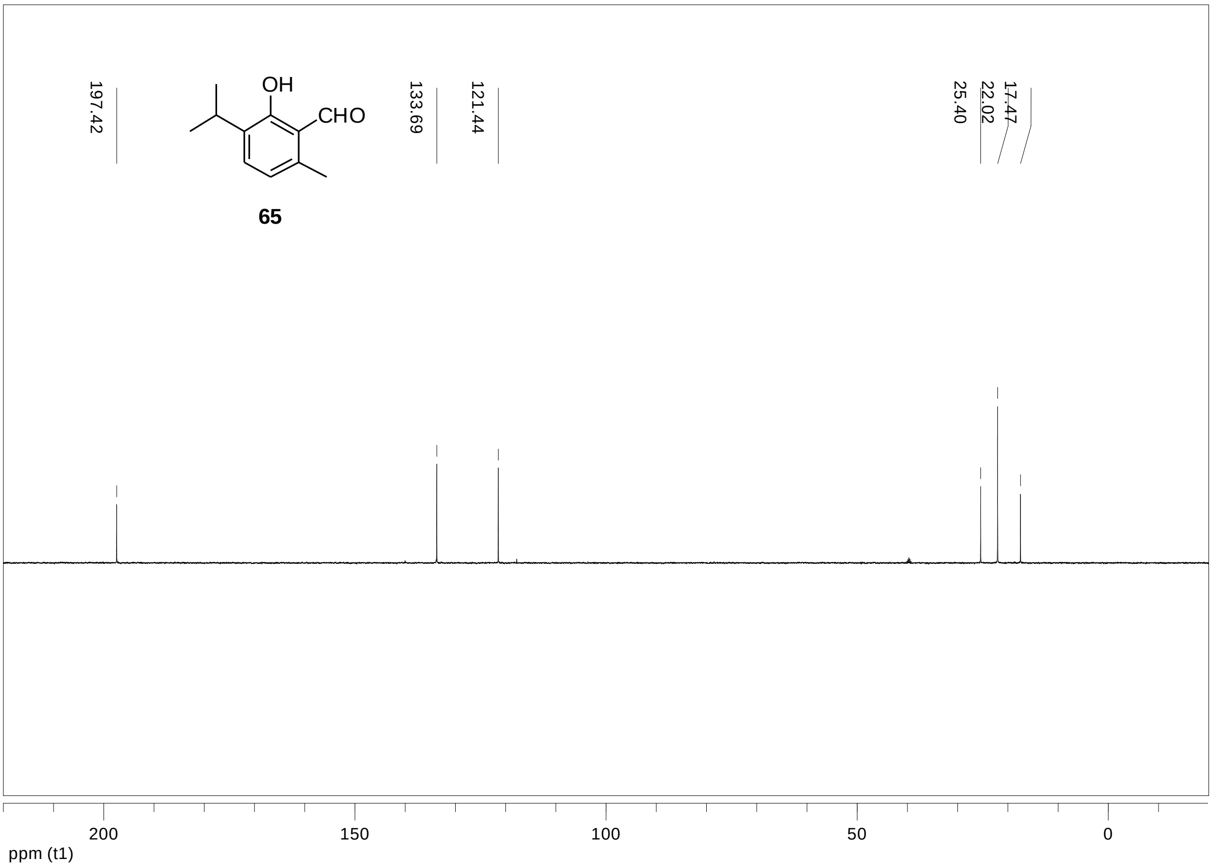

Figure S117. ^{13}C-NMR (101 MHz, DMSO-d_6) dept-135 experiment of salicylic aldehyde **65**.

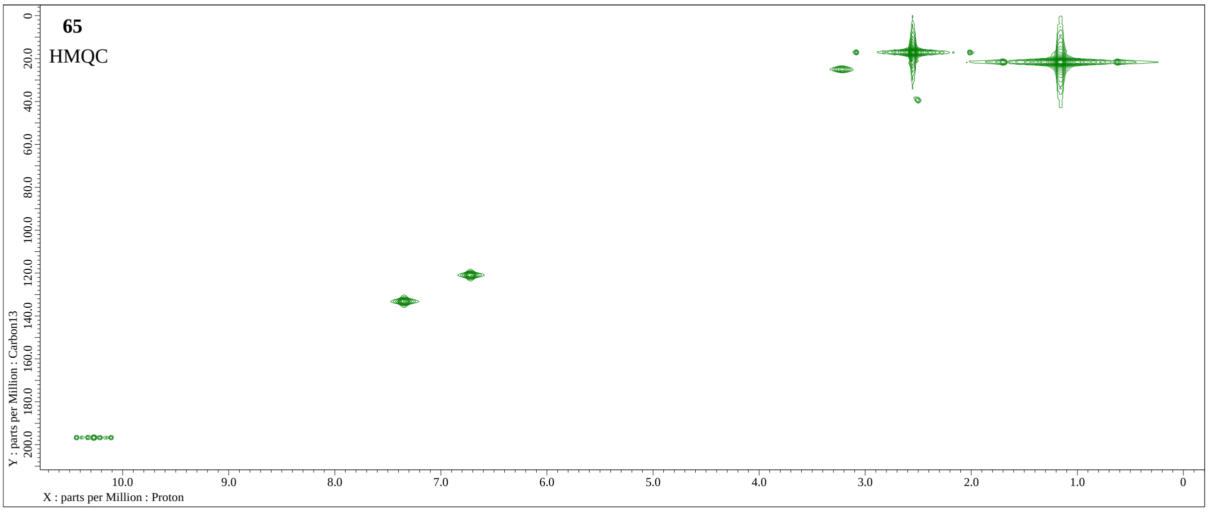

Figure S118. 2D-NMR (400 MHz, DMSO-d_6) HMQC experiment of 3-*iso*-propyl-6-methylsalicylic aldehyde (**65**).

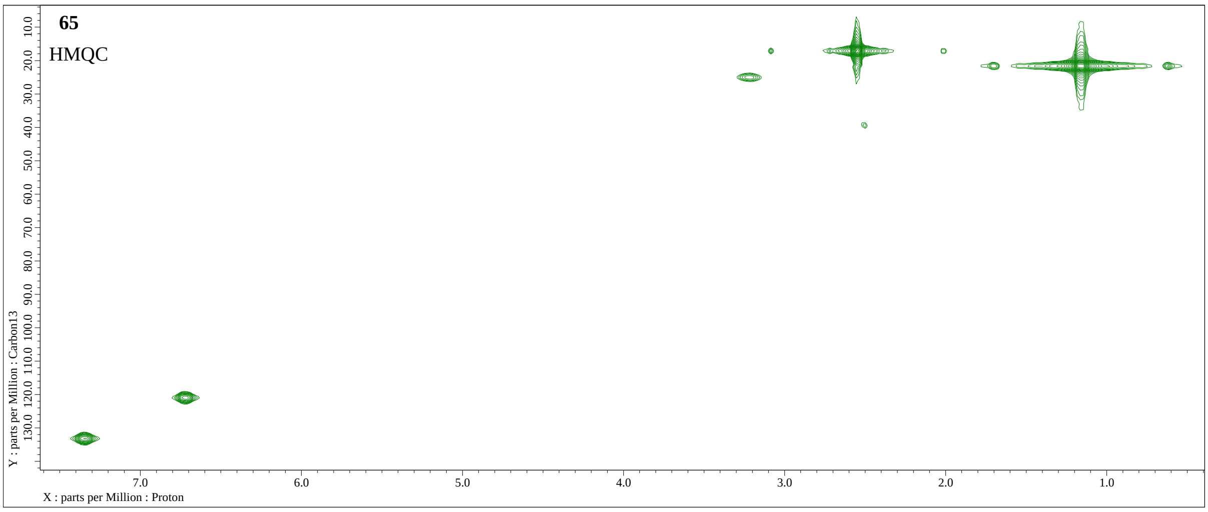

Figure S119. Expansion of 2D-NMR (400 MHz, DMSO-d_6) HMQC experiment of 3-*iso*-propyl-6-methylsalicylic aldehyde (**65**).

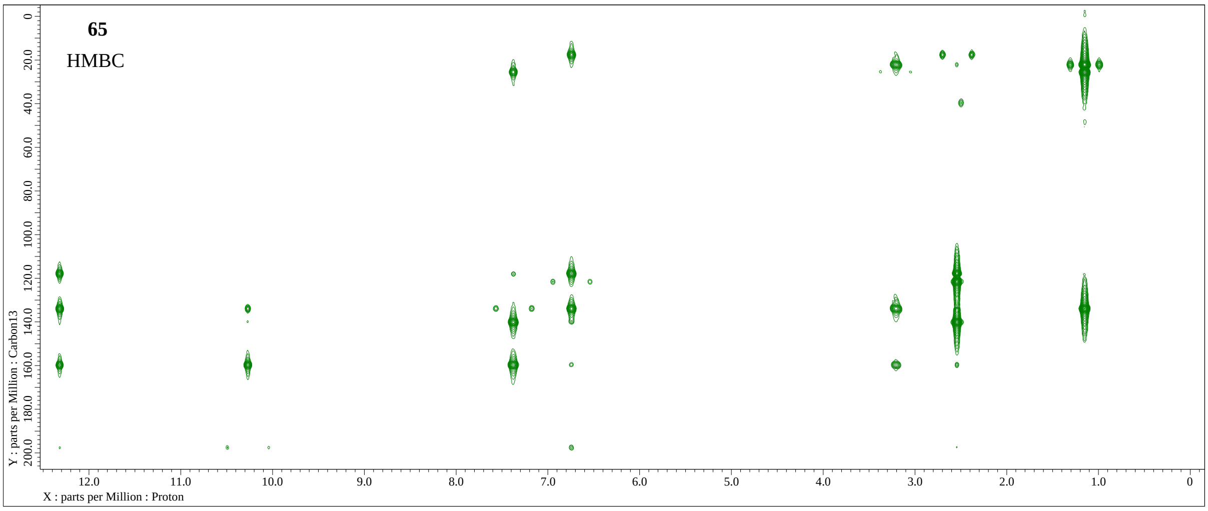

Figure S120. 2D-NMR (400 MHz, DMSO-d_6) HMBC experiment of 3-*iso*-propyl-6-methylsalicylic aldehyde (**65**).

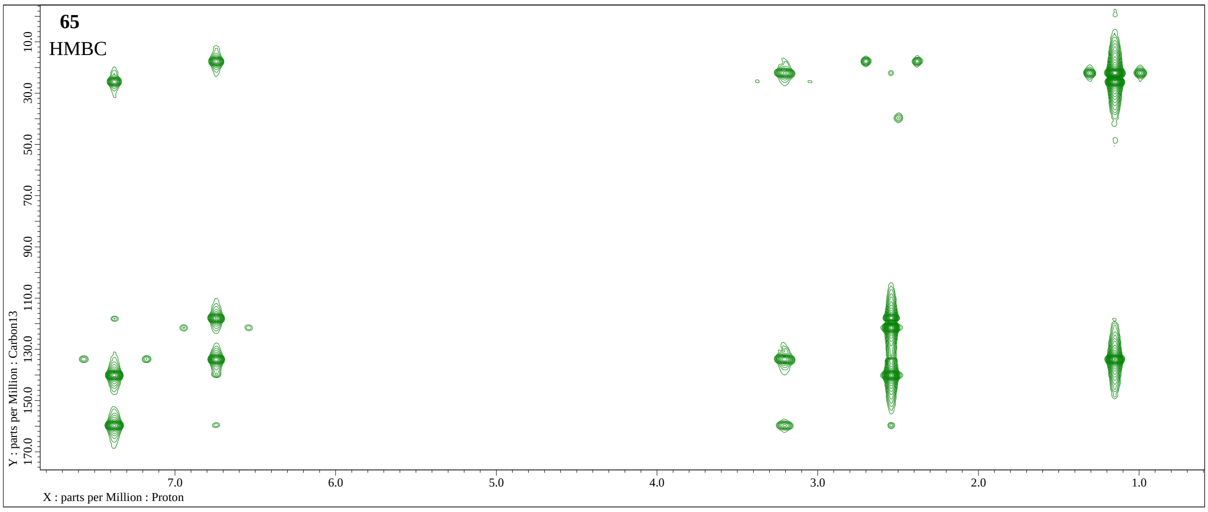

Figure S121. Expansion of 2D-NMR (400 MHz, DMSO-d_6) HMBC experiment of 3-*iso*-propyl-6-methylsalicylic aldehyde (**65**).

Preparation of 2,4-diformylphloroglucinol (68) [8]**.**

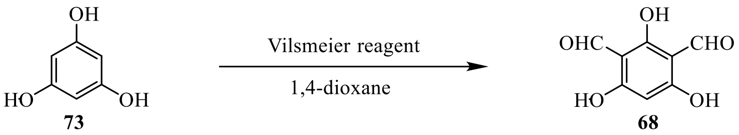

The 2,4-diformylphloroglucinol (**68**) was prepared with literature procedure [8] by diformylation of phloroglucinol (**73**) in 1,4-dioxane with Vilsmeier reagent prepared from a mixture of $POCl_3$ and DMF at 0 °C to obtain the 2,4-diformylphloroglucinol (**68**) as an orange powder which melt at 220–223 °C (m.p. 221–224 °C [8]); selected FT-IR (ATR): ν_{max} 3250–2250 (vbr, OH), 1593 (br, CHO), 1502, 1435, 1392, 1251 (C_{Ar}-O), 1182 (C_{Ar}-O), 1095, 947, 799, 711, 607, 534, 419 cm^{-1}; ^{1}H-NMR (400 MHz, DMSO-d_6): δ 13.50 (s, br, 1H, OH), 12.48 (s, br, 2H, OH), 9.99 (s, 2H, CHO), 5.88 (s, 1H, ArH-5) ppm; ^{13}C-NMR (101 MHz, DMSO-d_6): δ 191.33 (2 × CHO), 169.36 (ArC-4,6), 168.96 (ArC-2), 103.68 (ArC-1,3), 94.02 (ArC-5) ppm; HRMS (TOF, MS, ESI) *m*/*z* for $C_8H_6O_5$ + H^+ calculated: 183.0288; found: 183.0289.

The FT-IR and ^{13}C-NMR spectra are in agreement with the literature value [8]. The ^{1}H-NMR spectrum is partially consistent with the literature [8]. In our spectrum is present a broad singlet of hydroxy group at δ = 13.50 ppm.

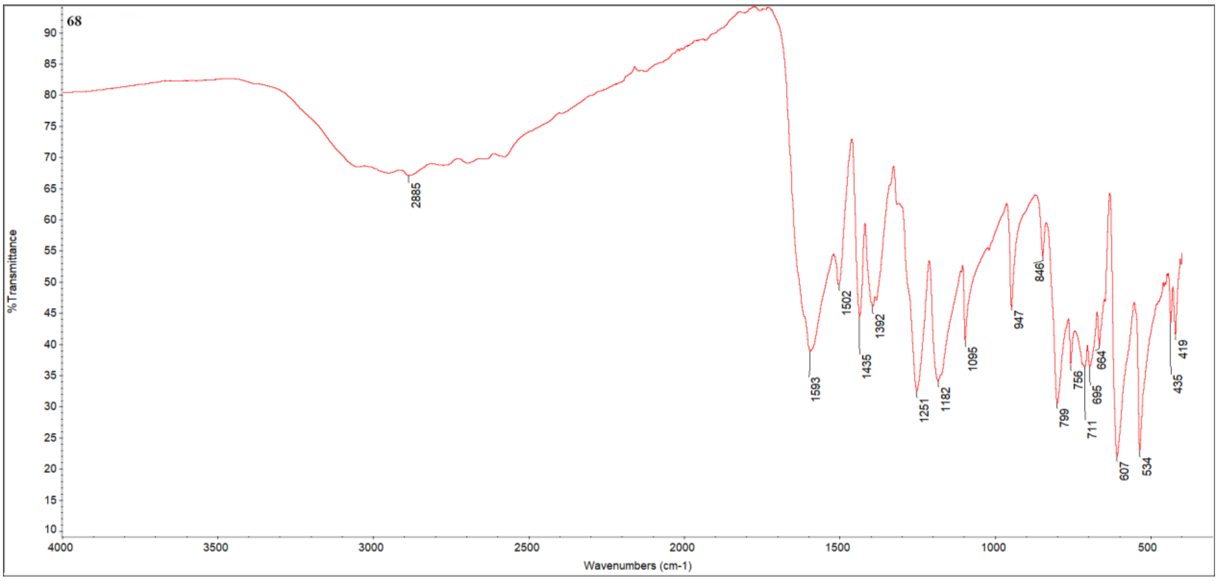

Figure S122. FT-IR (ATR) (4000–400 cm^{-1}) spectrum of 2,4-diformylphloroglucinol (**68**).

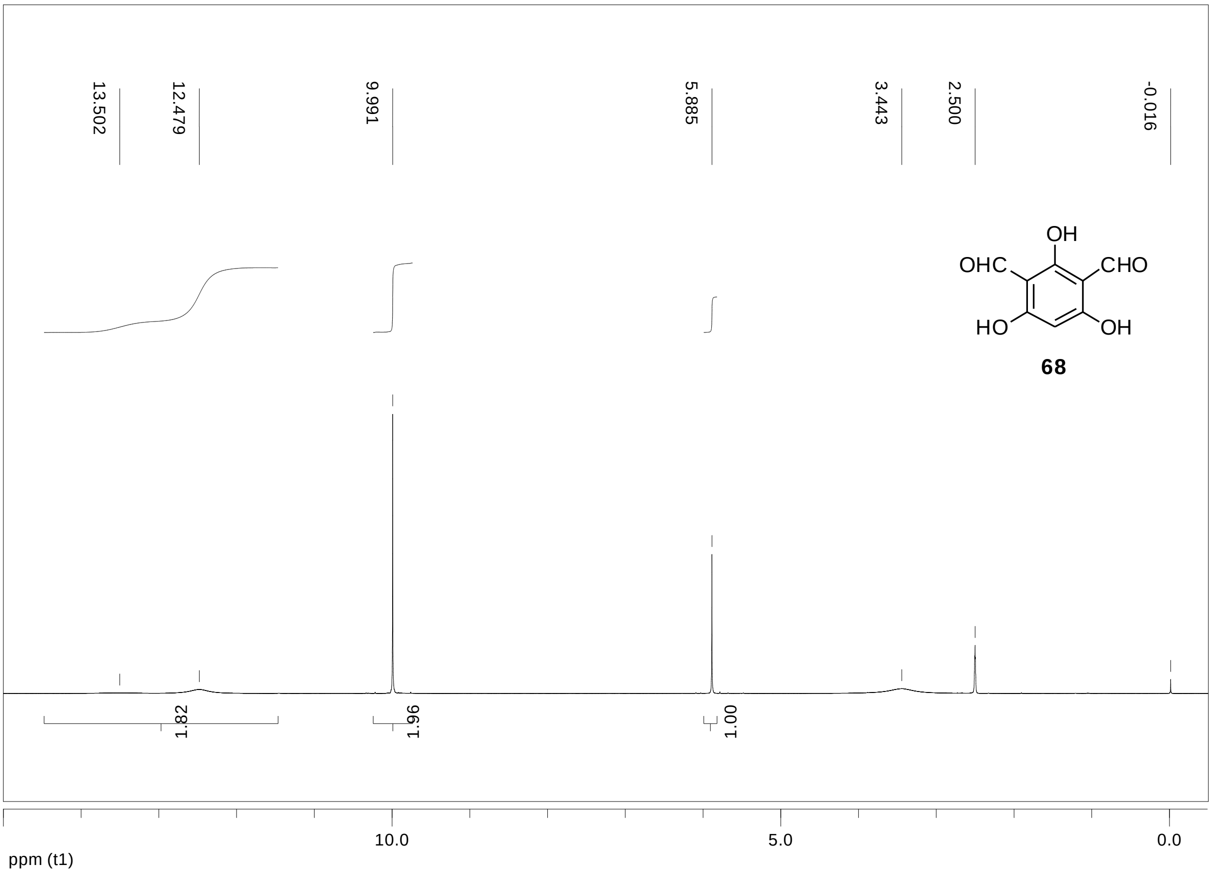

Figure S123. ^{1}H-NMR (400 MHz, DMSO-d_6) spectrum of 2,4-diformylphloroglucinol (**68**).

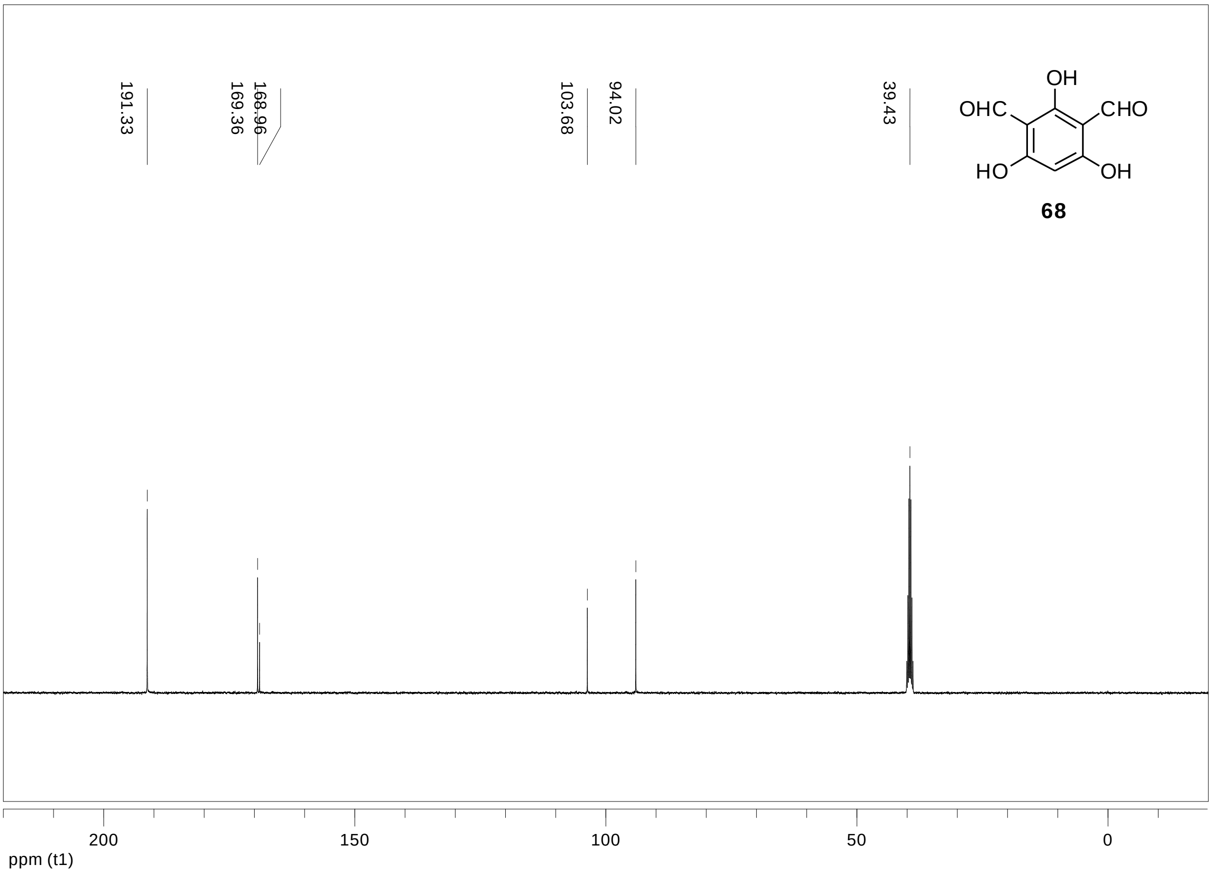

Figure S124. ^{13}C-NMR (101 MHz, DMSO-d_6) spectrum of 2,4-diformylphloroglucinol (**68**).

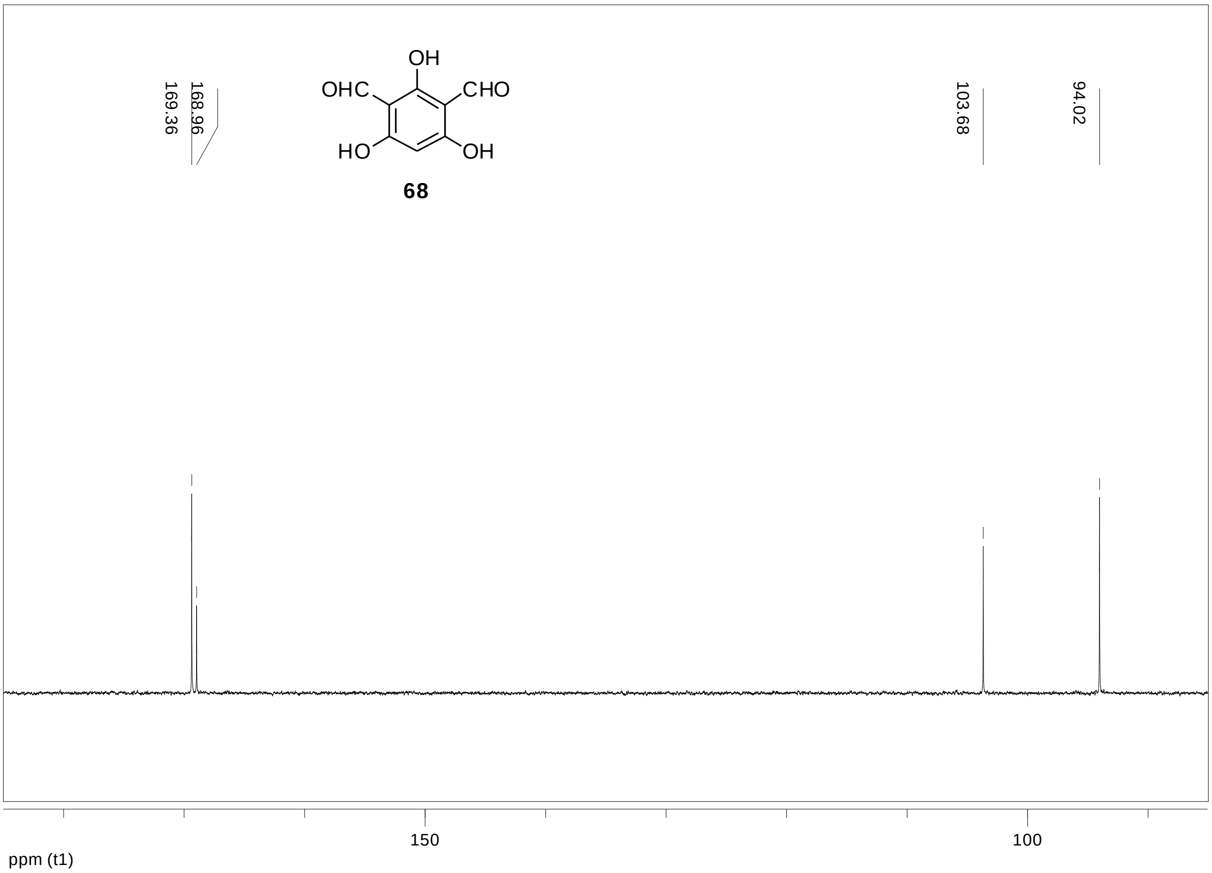

Figure S125. Expansion ^{13}C-NMR (101 MHz, DMSO-d_6) spectrum of diformylphloroglucinol (**68**).

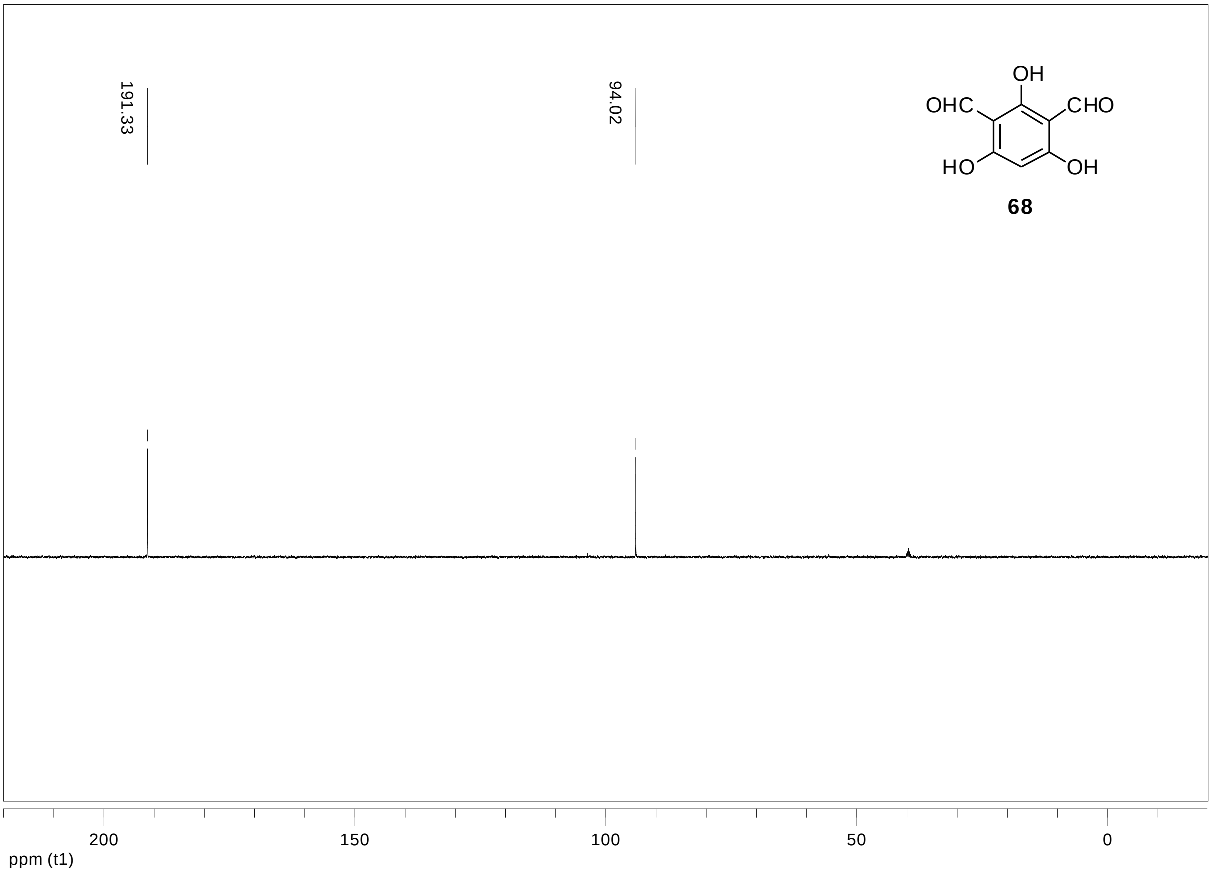

Figure S126. ^{13}C-NMR (101 MHz, DMSO-d_6) dept-135 experiment of diformylphloroglucinol (**68**).

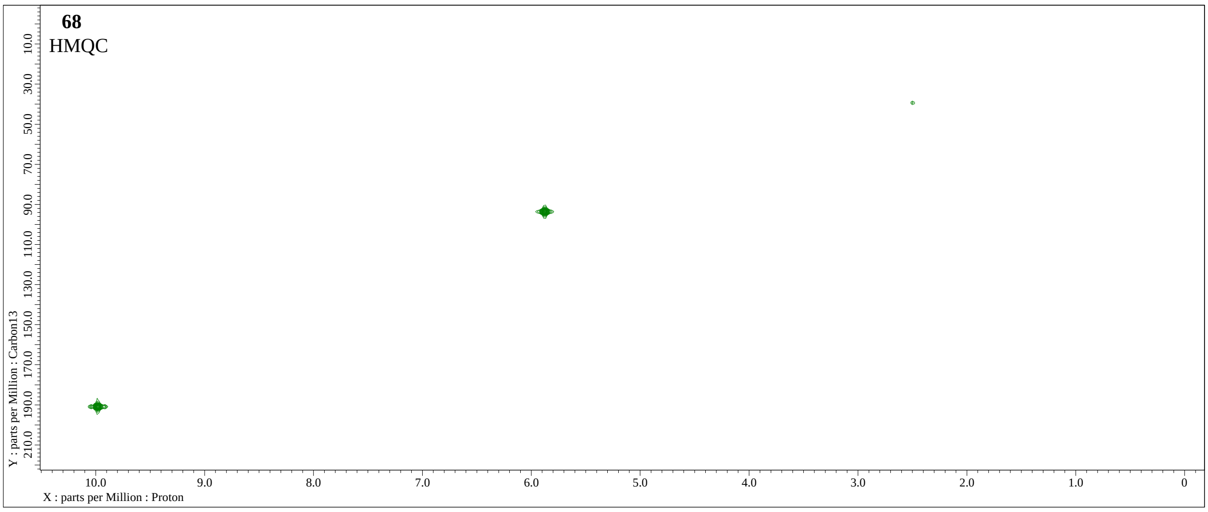

Figure S127. 2D-NMR (400 MHz, DMSO-d_6) HMQC experiment of 2,4-diformylphloroglucinol (**68**).

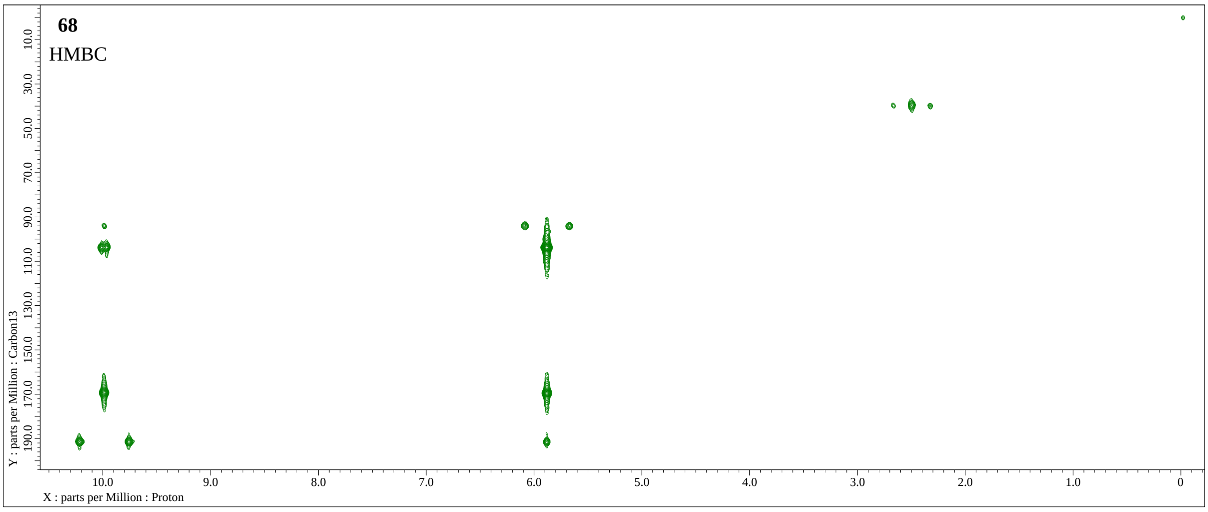

Figure S128. 2D-NMR (400 MHz, DMSO-d_6) HMBC experiment of 2,4-diformylphloroglucinol (**68**).

General Procedure for the synthesis of hydrazides 38–40 [1,3]**.**

The synthesis procedure was adapted from the literature [1,3]. To a solution of appropriate carboxylic acid methyl ester (**74–76**) (10 mmol) in dry CH_3OH (25 mL), $H_2NNH_2 \times H_2O$ (0.53 mL, 10.5 mmol) was added. The reaction mixture was stirred under gentle reflux for 36 to 48 hours. From the reaction mixture, solvent was distilled off, dry in desiccator under P_2O_5 at 20 mmHg, and recrystallized the crude product from methanol in an open vessel to obtain the hydrazides **38–40**.

***p*-Anisic acid hydrazide (38) preparation** [3]**.**

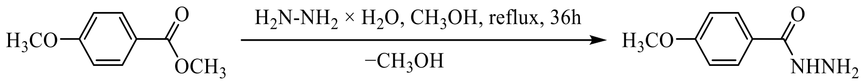

74 **38**

The general procedure starting from methyl 4-methoxybenzoate (**74**) (1.66g, 10 mmol) [1] was employed for 36 hours reaction times to obtain a colorless powder of *p*-Anisic acid hydrazide – 4-methoxybenzohydrazide (**38**) (1.42g, 8.5 mmol) with 85% yield, which melt at 137–139 °C (from CH_3OH) (m.p. 136–138 °C [1]).

The FT-IR, ^{1}H-NMR, and ^{13}C-NMR data are consistent with literature values [15].

Benzohydrazide (39) preparation [3]**.**

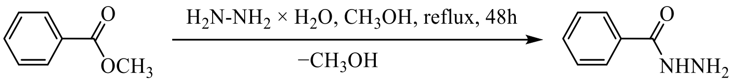

75 **39**

The general procedure starting from methyl benzoate (**75**) (1.36g, 10 mmol) [3] was employed with 48 h reaction time to obtain benzohydrazide (**39**) (1.09g, 8.0 mmol) with 80% yield, which melts at 115–116 °C (water, 7 mL/g) (m.p. 110–113 °C [11]). Colorless white prisms of analytically pure samples were obtained by recrystallization from methanol, which melts at 116–117 °C (m.p. 114–115 °C [12]).

The FT-IR, ^{1}H-NMR, and ^{13}C-NMR data are consistent with literature values [15].

Nicotinic acid hydrazide (40) preparation [3]**.**

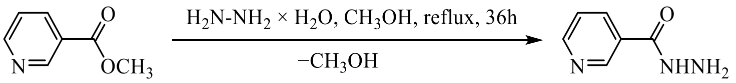

76 **40**

The general procedure starting from nicotinic acid methyl ester (**76**) (1.37g, 10 mmol) was employed at 36 hours to obtain a colorless powder of nicotinic acid hydrazide (**40**) (1.17g, 8.54 mmol) with 85% yield, which melt at 166–167 °C (from CH_3OH) (m.p. 165–167 °C [3]) (m.p. 159–160 °C [12]).
The FT-IR, ^{1}H-NMR, ^{13}C-NMR, and HRMS data are generally consistent with literature values [3].

In vitro antifungal activity

Prior to the fungicidal activity assay of hydrazide-hydrazones **1–35**, we considered ethanol (EtOH) and DMSO as potential dissolution media for tested compounds. Most of the hydrazide-hydrazones have poor solubility in water. EtOH and DMSO with a final concentration between 0.1–1.0% (*v*/*v*) in a PDA medium were applied to evaluate the inhibition growth of *B. cinerea*, *S. sclerotiorum*, and *C. unicolor* (see Table S3). The following procedure was applied to the microorganism testing with the hydrazide-hydrazones. The assays of fungi inhibition growth were performed at a basal concentration of 50 µg/mL for all compounds, and detailed experiments were extended for testing selected molecules **18**, **19**, **27**, **28**, and **30** (see Table S4), between 0–50 µg/mL (0, 6.25, 12.5, 25.0, and 50.0 µg/mL).

Table S2. *In vitro* antifungal activity for DMSO and ethanol co-solvents. *Botrytis cinerea* (**red circles**), *Sclerotinia sclerotiorum* (**black squares**), *Cerrena unicolor* (**blue triangles**). Growth inhibition, % (y-axis) in the function of compound concentration, µg/mL (x-axis).

Co-solvent	Regression lines: y=f(x),<br>y = growth inhibition (%)<br>x = concentration [µg/mL]	*B. cinerea* (**red circles**)			*S. sclerotiorum* (**black squares**)			*C. unicolor* (**blue triangles**)		
		**IC50 µg/mL**	R^2	RMSE	**IC50 µg/mL**	R^2	RMSE	**IC50 µg/mL**	R^2	RMSE
**DMSO**	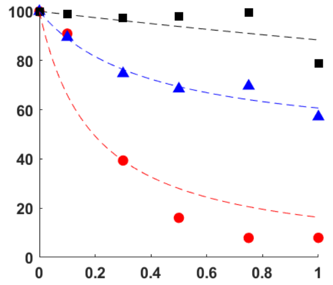	**0.19**	0.80	15.76	**7.62**	0.41	6.80	**4.03**	0.91	3.98
**EtOH**	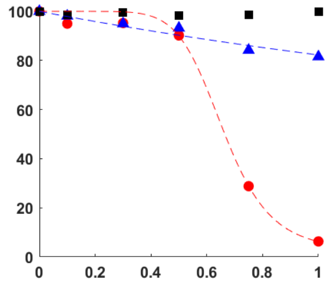	**0.66**	0.99	4.80	**n.o.**	–	–	**4.63**	0.93	1.86

Table S3. *In vitro* antifungal activity for compounds **18**, **19**, **27**, **28**, and **30**. *Botrytis cinerea* (black squares), *Sclerotinia sclerotiorum* (**red circles**), *Cerrena unicolor* (**blue triangles**). Growth inhibition, % (y-axis) in the function of compound concentration, µg/mL (x-axis).

**No.**	Regression lines: y = f(x), y = growth inhibition (%) x = concentration [µg/mL]	*B. cinerea* (**red circles**)			*S. sclerotiorum* (**black squares**)			*C. unicolor* (**blue triangles**)		
		**IC50 µg/mL**	R^2	RMSE	**IC50 µg/mL**	R^2	RMSE	**IC50 µg/mL**	R^2	RMSE
**18**	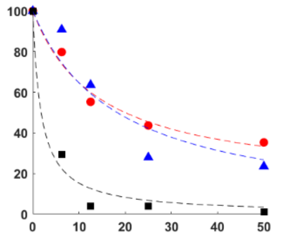	**20.0**	0.94	5.65	**1.8**	0.74	0.68	**18.3**	0.84	12.92
**19**	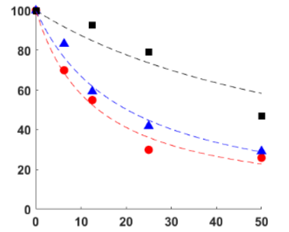	**13.9**	0.96	5.30	**69.9**	0.82	12.62	**20.3**	0.96	4.54
**27**	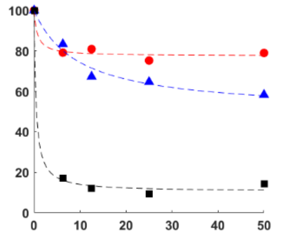	**n.d.**	0.13	2.72	**0.5**	0.43	3.13	**2194**	0.91	3.84
**28**	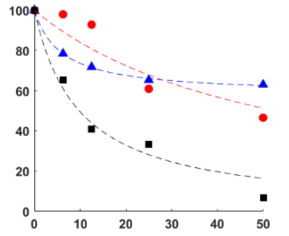	**52.7**	0.84	9.80	**9.8**	0.92	6.97	**n.d.**	0.99	0.69
**30**	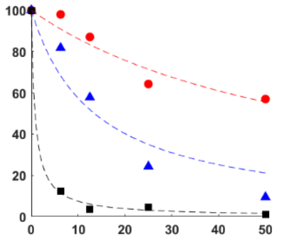	**62.5**	0.89	6.24	**0.8**	0.87	1.72	**13.5**	0.85	12.50
Fenhexamid	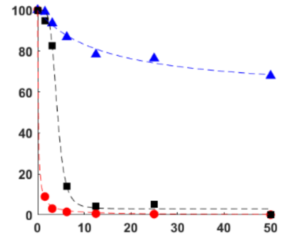	**0.1**	0.95	0.73	**4.2**	1.00	3.35	**n.d.**	0.96	2.47

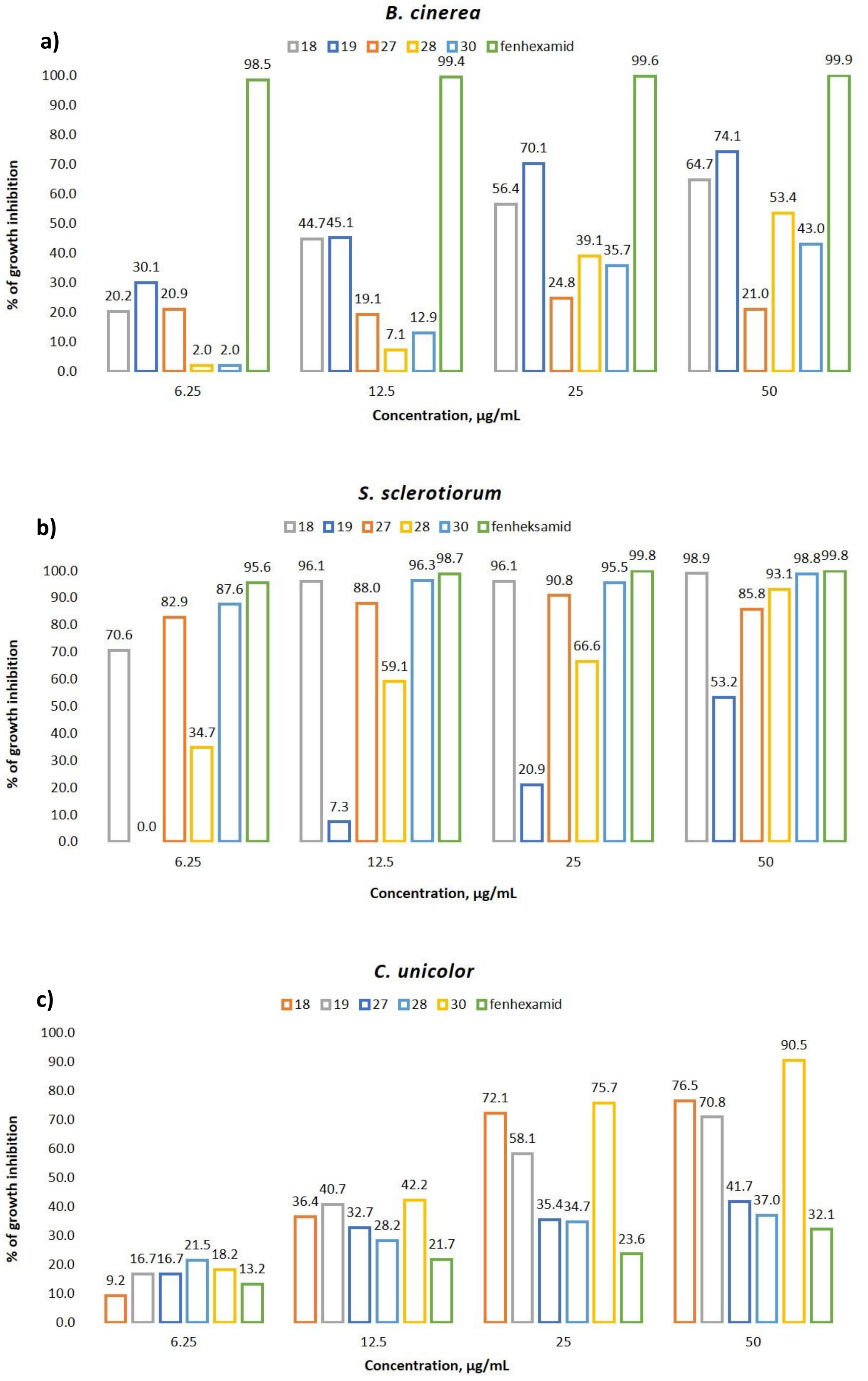

Figure S129. Fungicidal profiles of hydrazide-hydrazones **18**, **19**, **27**, **28**, **30** and reference fenhexsamid illustrated for 6.25, 12.5, 25.0, and 50.0 µg/mL concentration and a) *Botrytis cinerea*, b) *Sclerotina sclerotiorum* and c) *Cerrena unicolor*.

Cytotoxicity profiles

Cytotoxicity was performed on two normal cell lines: MCF-10A and Balb/3T3, using as controls *cis*-platin in the concentrations of 0.0332, 0.332, 3.32 and 33.2 µM and azoxystrobin fungicide in the concentrations of 0.05, 0.5, 5.0 and 50.0 µM; the remaining compounds: **13**, **17**, **18**, **19**, **25**, **27**, and **30** were tested in the concentrations of 0.5, 5.0, 50, and 500 µM. The relation of compound concentration [µM] *versus* antiproliferation activity [%] was presented in Table S4. Further, a comparison of the influence of each tested concentration in the group of tested compounds on cell lines was illustrated in Figure S130 a (MCF-10A) and b (Balb/T3T).

Table S4. Cytotoxicity results for compounds **13**, **17**, **18**, **19**, **25**, **27**, and **30** investigated on normal mammalian lines: MCF-10A and Balb/3T3 using Azoxystrobin (fungicide) and *cis*-platin as reference compounds. Cytotoxicity profile presented as proliferation inhibition, % (y-axis) in the function of compound concentration, µg/mL (x-axis).

Structure	MCF-10A	Balb/3T3
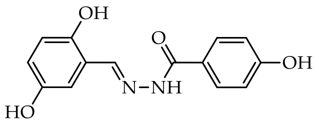 **13**	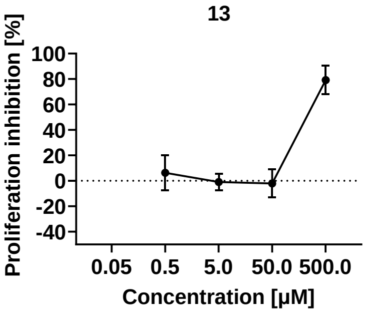	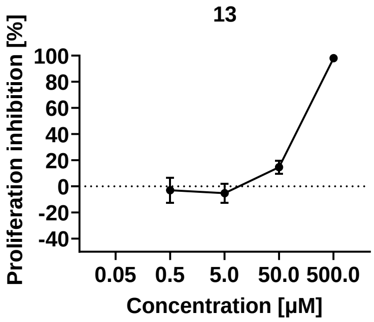
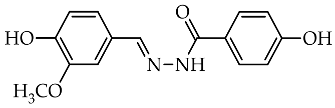 **17**	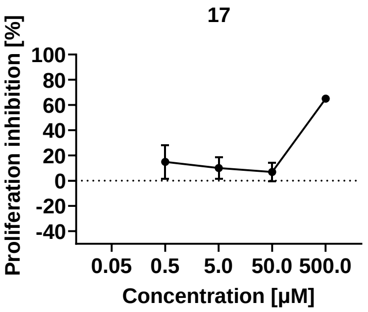	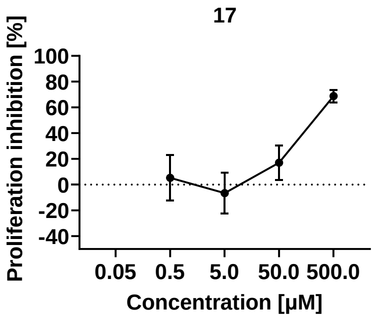
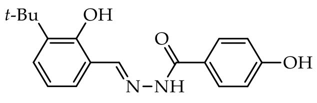 **18**	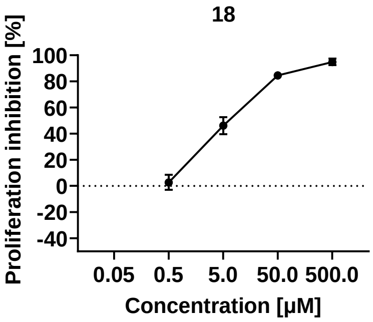	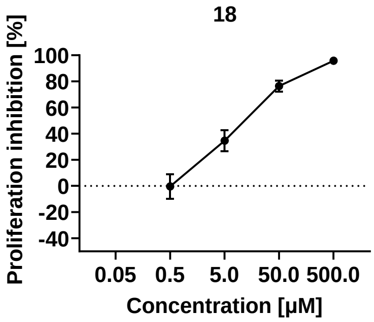

Structure	MCF-10A	Balb/3T3
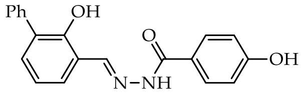<br>**19**	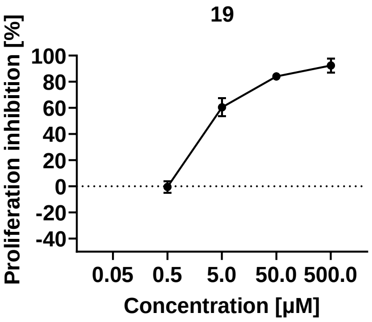	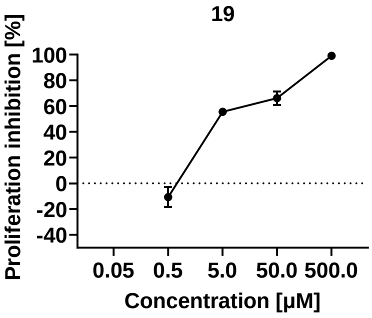
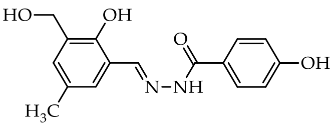<br>**25**	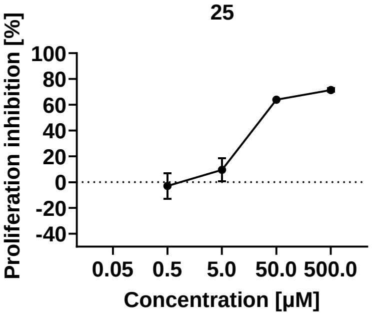	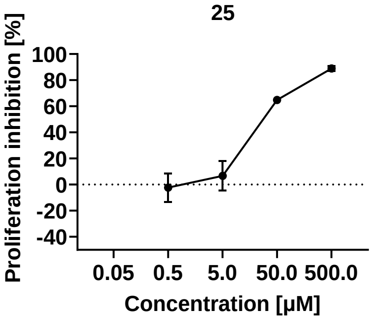
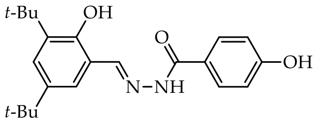<br>**27**	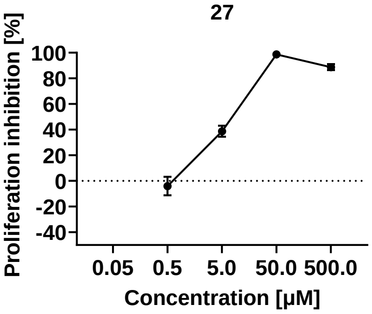	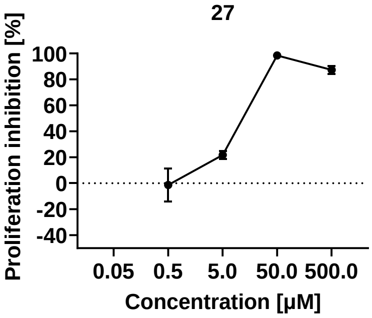
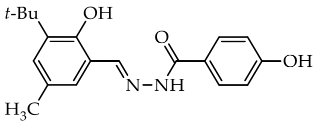<br>**30**	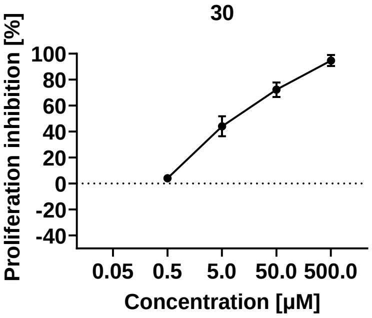	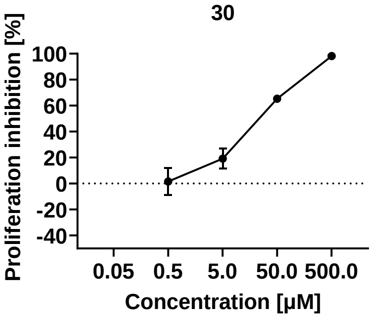
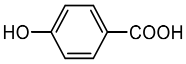<br>**4-hba**	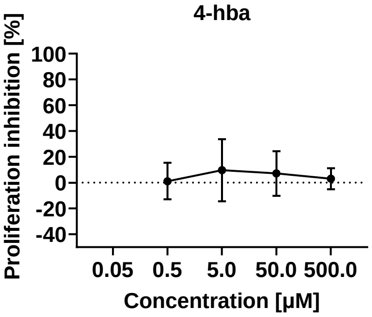	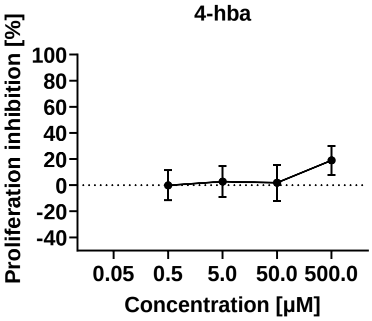

<table>
<tr><th>Structure</th><th>MCF-10A</th><th>Balb/3T3</th></tr>
<tr><td>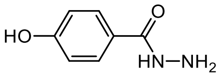

36 (4-hbah)</td><td>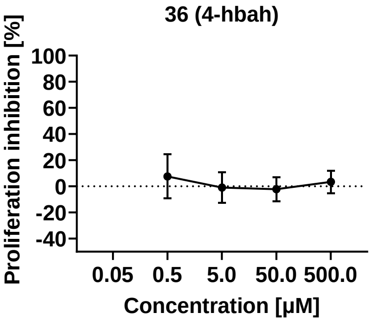
</td><td>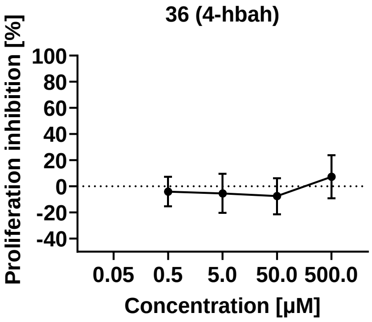
</td></tr>
<tr><td>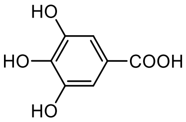

3,4,5-hba</td><td>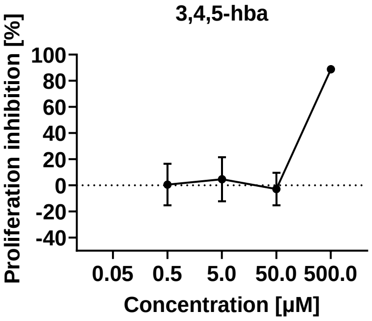
</td><td>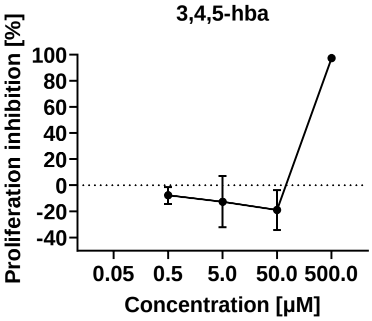
</td></tr>
<tr><td>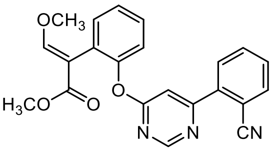

Azoxystrobin [a]</td><td>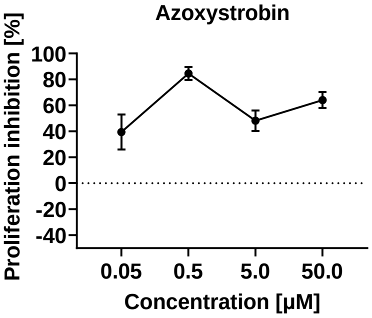
</td><td>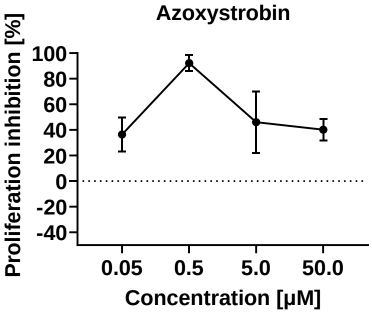
</td></tr>
<tr><td>Cis-platin</td><td>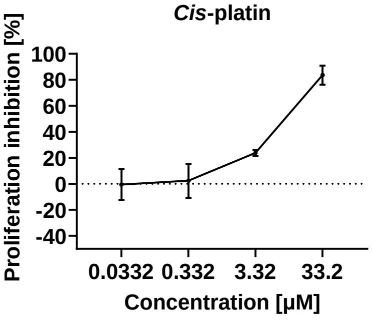
</td><td>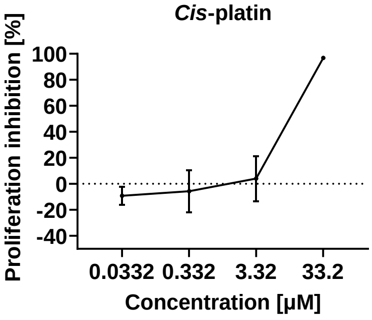
</td></tr>
</table>

[a] For the MCF-10A line, IC_{50} values were not determined due to the lack of a direct (typical) relationship of azoxystrobin concentration to inhibition of cell proliferation.

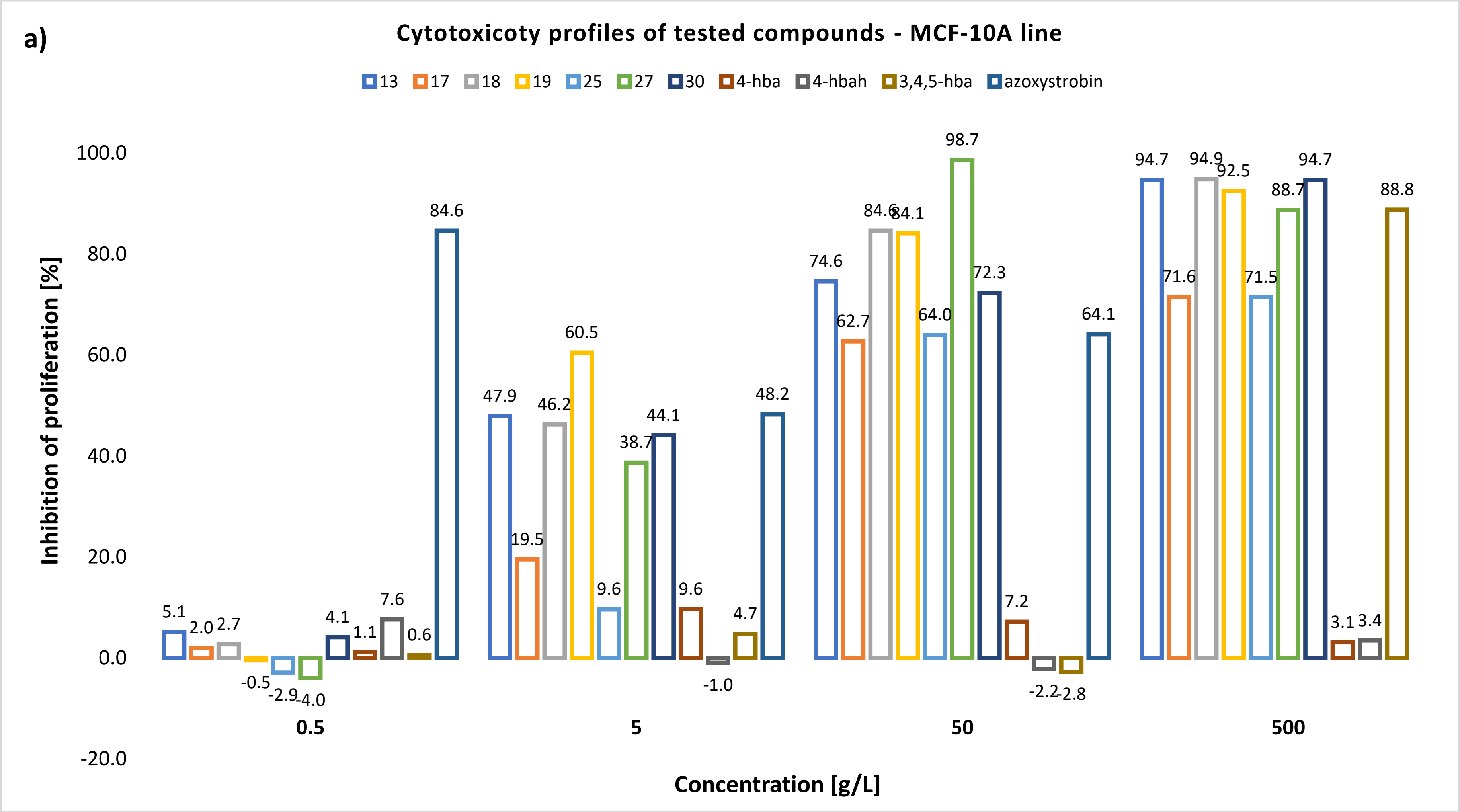
a)
Cytotoxicoty profiles of tested compounds - MCF-10A line
13
17
18
19
25
27
30
4-hba
4-hbah
3,4,5-hba
azoxystrobin
Inhibition of proliferation [%]
Concentration [g/L]
0.5
5
50
500
100.0
80.0
60.0
40.0
20.0
0.0
-20.0
5.1
2.0
2.7
-0.5
-2.9
-4.0
4.1
1.1
7.6
0.6
84.6
47.9
19.5
46.2
60.5
9.6
38.7
44.1
9.6
-1.0
4.7
48.2
74.6
62.7
84.6
84.1
64.0
98.7
72.3
7.2
-2.2
-2.8
64.1
94.7
71.6
94.9
92.5
71.5
88.7
94.7
3.1
3.4
88.8

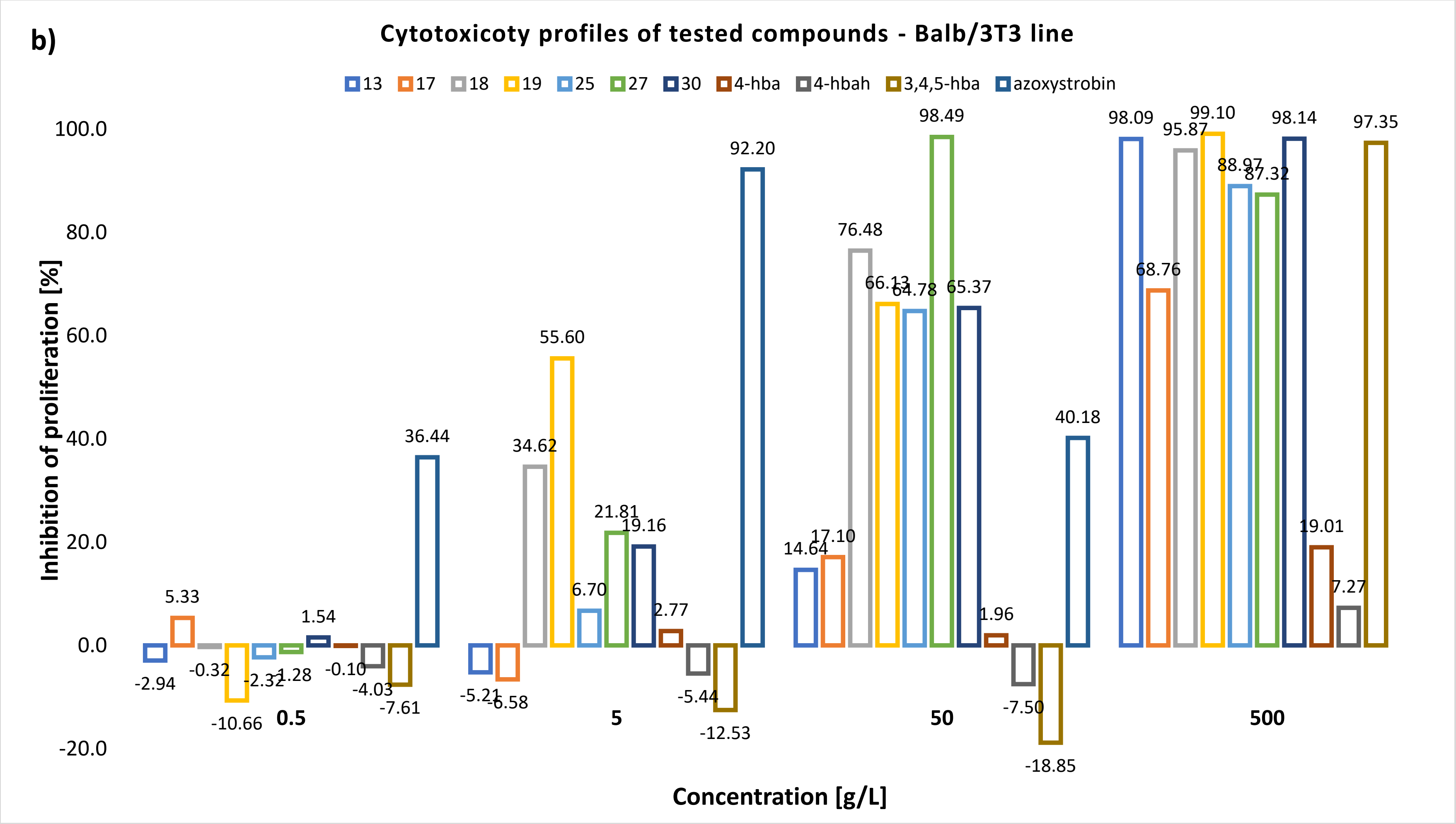

Figure S130. Cytotoxicity profile comparison between tested concentrations: 0.5, 5.0, 50.0 and 500 μM of the compounds: hydrazide-hydrazone **13**, **17**, **18**, **19**, **25**, **27**, 30; **4-hba** (4-hydroxybenzoic acid); **4-hbah** (4-hydroxybenzoic acid hydrazide); **3,4,5-hba** (gallic acid). **Azoxystrobin** (fungicide, reference) was tested for 0.05, 0.5, 5.0 and 50 μM. a) Studies performed on the MCF-10A line and b) on the Balb/3T3 line.

References

1. Maniak, H.; Talma, M.; Matyja, K.; Trusek, A.; Giurg, M. Synthesis and Structure-Activity Relationship Studies of Hydrazide-Hydrazones as Inhibitors of Laccase from Trametes Versicolor. *Molecules* **2020**, *25*, 1255, doi:10.3390/molecules25051255.

2. Giurg, M.; Maniak, H. Iminowe Pochodne Aldehydów Salicylowych i Hydrazydu Kwasu 4-Hydroksybenzoesowego Oraz Sposób Ich Wytwarzania. Patent PL 233208 B1 2019, 1–8.

3. Maniak, H.; Talma, M.; Giurg, M. Inhibitory Potential of New Phenolic Hydrazide-Hydrazones with a Decoy Substrate Fragment towards Laccase from a Phytopathogenic Fungus: SAR and Molecular Docking Studies. *Int J Mol Sci* **2021**, *22*, 12307, doi:10.3390/ijms222212307.

4. Serra, S.; Alouane, A.; Le Saux, T.; Huvelle, S.; Plasson, R.; Schmidt, F.; Jullien, L.; Labruère, R. A Chemically Encoded Timer for Dual Molecular Delivery at Tailored Ranges and Concentrations. *Chemical Communications* **2018**, *54*, 6396–6399, doi:10.1039/c8cc03253j.

5. Rajput, J.D.; Bagul, S.D.; Hosamani, A.A.; Patil, M.M.; Bendre, R.S. Synthesis, Characterizations, Biological Activities and Docking Studies of Novel Dihydroxy Derivatives of Natural Phenolic Monoterpenoids Containing Azomethine Linkage. *Research on Chemical Intermediates* **2017**, *43*, 5377–5393, doi:10.1007/s11164-017-2933-4.

6. Gou, H.; Zhang, J.; Li, P.; Li, C.; Wang, H.; Hong, W. A Practical Total Synthesis of Wedelolactone. *Synth Commun* **2023**, *53*, 1126–1133, doi:10.1080/00397911.2023.2211772.

7. Hu, Y.; Hu, H. A Novel Selective Oxidation of 5-Substituted 2-Hydroxy-3-Hydroxymethylbenzaldehydes. *Synthesis (Stuttg)* **1991**, *4*, 325–326, doi:10.1055/s-1991-26458.

8. Lee, H.; Park, R.Y.; Park, K. Total Syntheses of 4′,6′-Dimethoxy-2’-Hydroxy-3′,5′-Dimethylchalcone Derivatives. *Bull Korean Chem Soc* **2021**, *42*, 66–71, doi:10.1002/bkcs.12156.

9. Fan, H.; Peng, X. Photoinduced DNA Interstrand Cross-Linking by Benzene Derivatives: Leaving Groups Determine the Efficiency of the Cross-Linker. *Journal of Organic Chemistry* **2021**, *86*, 493–506, doi:10.1021/acs.joc.0c02234.

10. Nassar, M.Y.; El-Shwiniy, W.H.; El-Desoky, S.I. Synthesis of Pd(II), Ag(I), Pt(IV), and Hg(II) Complexes with Nifuroxazide, Their Structure, DFT Modeling, and Antimicrobial and Anticancer Activity. *Russ J Gen Chem* **2018**, *88*, 573–579, doi: 10.1134/S1070363218030295.

11. Nisa, M.; Munawar, M.A.; Iqbal, A.; Ahmed, A.; Ashraf, M.; Gardener, Q.-A.A.; Khan, M.A. Synthesis of Novel 5-(Aroylhydrazinocarbonyl)Escitalopram as Cholinesterase Inhibitors. *Eur J Med Chem* **2017**, *138*, 396–406, doi:10.1016/j.ejmech.2017.06.036.

12. Freitas, R.H.C.N.; Barbosa, J.M.C.; Bernardino, P.; Sueth-Santiago, V.; Wardell, S.M.S.V.; Wardell, J.L.; Decoté-Ricardo, D.; Melo, T.G.; da Silva, E.F.; Salomão, K.; et al. Synthesis and Trypanocidal Activity of Novel Pyridinyl-1,3,4-Thiadiazole Derivatives. *Biomedicine and Pharmacotherapy* **2020**, *127*, 110162, doi:10.1016/j.biopha.2020.110162.

13. Seo, Y.H.; Damodar, K.; Kim, J.K.; Jun, J.G. Synthesis and Biological Evaluation of 2-Aroylbenzofurans, Rugchalcones A, B and Their Derivatives as Potent Anti-Inflammatory Agents. *Bioorg Med Chem Lett* **2016**, *26*, 1521–1524, doi:10.1016/j.bmcl.2016.02.023.

14. Jacq, J.; Einhorn, C.; Einhorn, J. A Versatile and Regiospecific Synthesis of Functionalized 1,3-Diarylisobenzofurans. *Org Lett* **2008**, *10*, 3757–3760, doi:10.1021/ol801550a.

15. Pereira, T.M.; Vitório, F.; Amaral, R.C.; Zanoni, K.P.S.; Murakami Iha, N.Y.; Kümmerle, A.E. Microwave-Assisted Synthesis and Photophysical Studies of Novel Fluorescent N-Acylhydrazone and Semicarbazone-7-OH-Coumarin Dyes. *New Journal of Chemistry* **2016**, *40*, 8846–8854, doi:10.1039/c6nj01532h.

16. Zuercher, W.J.; Gaillard, S.; Orband-Miller, L.A.; Chao, E.Y.H.; Shearer, B.G.; Jones, D.G.; Miller, A.B.; Collins, J.L.; McDonnell, D.P.; Willson, T.M. Identification and Structure-Activity of Phenolic Acyl as Selective Agonists

for the Estrogen-Related Orphan Nuclear Receptors ERRβ and ERRγ. *J Med Chem* **2005**, *48*, 3107–3109, doi:10.1021/jm050161j.

17. Rando, D.G.; Avery, M.A.; Tekwani, B.L.; Khan, S.I.; Ferreira, E.I. Antileishmanial Activity Screening of 5-Nitro-2-Heterocyclic Benzylidene Hydrazides. *Bioorg Med Chem* **2008**, *16*, 6724–6731, doi:10.1016/j.bmc.2008.05.076.

18. Han, S.Y.; Kim, B.K.; Lee, H.J.; Yoon, K.B. Novel Compounds Preparation Method Thereof and Pharmaceutical Composition for Use in Preventing or Treating Abnormal Cell Growth Diseases Containing the Same as an Active Ingredient. Patent KR101958107B1 2019, 1–20.

19. Xiong, H.; Liang, H.; Dai, K.; Tian, Q.; Dai, X.; Su, H.; Royal, G. Acylhydrazones as Sensitive Fluorescent Sensors for Discriminative Detection of Thorium(IV) from Uranyl and Lanthanide Ions. *Spectrochim Acta A Mol Biomol Spectrosc* **2023**, *293*, 122501, doi:10.1016/j.saa.2023.122501.

20. Javaid, S.; Saad, S.M.; Zafar, H.; Malik, R.; Khan, K.M.; Iqbal Choudhary, M.; Rahman, A. Thymidine Phosphorylase and Prostrate Cancer Cell Proliferation Inhibitory Activities of Synthetic 4-Hydroxybenzohydrazides: In Vitro, Kinetic, and in Silico Studies. *PLoS One* **2020**, *15*, e0227549, doi:10.1371/journal.pone.0227549.

21. Cedergreen, N.; Andersen, L.; Olesen, C.F.; Spliid, H.H.; Streibig, J.C. Does the Effect of Herbicide Pulse Exposure on 1083 Aquatic Plants Depend on Kow or Mode of Action? *Aquatic Toxicology* **2005**, *71*, 261–271, 1084 doi:10.1016/j.aquatox.2004.11.010. 1085

22. Ritz, C. Toward a Unified Approach to Dose-Response Modeling in Ecotoxicology. *Environ Toxicol Chem* **2010**, *29*, 220–1086 229, doi:10.1002/etc.7.
